# Dendritic cell immunotherapy followed by cART interruption during HIV-1 infection induces plasma protein markers of cellular immunity and neutrophil recruitment

**DOI:** 10.1371/journal.pone.0192278

**Published:** 2018-02-01

**Authors:** Henk-Jan van den Ham, Jason D. Cooper, Jakub Tomasik, Sabine Bahn, Joeri L. Aerts, Albert D. M. E. Osterhaus, Rob A. Gruters, Arno C. Andeweg, on behalf of the DC-TRN trial investigators

**Affiliations:** 1 Department of Viroscience, Erasmus Medical Center, Rotterdam, The Netherlands; 2 Department of Chemical Engineering and Biotechnology, University of Cambridge, Cambridge, United Kingdom; 3 Department of Neuroscience, Erasmus Medical Center, Rotterdam, The Netherlands; 4 Laboratory of Molecular and Cellular Therapy, Department of Physiology and Immunology, Vrije Universiteit Brussel, Brussels, Belgium; 5 Research Institute for Infectious Diseases and Zoonoses, Veterinary University Hannover, Hannover, Germany; Imperial College London, UNITED KINGDOM

## Abstract

**Objectives:**

To characterize the host response to dendritic cell-based immunotherapy and subsequent combined antiretroviral therapy (cART) interruption in HIV-1-infected individuals at the plasma protein level.

**Design:**

An autologous dendritic cell (DC) therapeutic vaccine was administered to HIV-infected individuals, stable on cART. The effect of vaccination was evaluated at the plasma protein level during the period preceding cART interruption, during analytical therapy interruption and at viral reactivation. Healthy controls and post-exposure prophylactically treated healthy individuals were included as controls.

**Methods:**

Plasma marker (‘analyte’) levels including cytokines, chemokines, growth factors, and hormones were measured in trial participants and control plasma samples using a multiplex immunoassay. Analyte levels were analysed using principle component analysis, cluster analysis and limma. Blood neutrophil counts were analysed using linear regression.

**Results:**

Plasma analyte levels of HIV-infected individuals are markedly different from those of healthy controls and HIV-negative individuals receiving post-exposure prophylaxis. Viral reactivation following cART interruption also affects multiple analytes, but cART interruption itself only has only a minor effect. We find that Thyroxine-Binding Globulin (TBG) levels and late-stage neutrophil numbers correlate with the time off cART after DC vaccination. Furthermore, analysis shows that cART alters several regulators of blood glucose levels, including C-peptide, chromogranin-A and leptin. HIV reactivation is associated with the upregulation of CXCR3 ligands.

**Conclusions:**

Chronic HIV infection leads to a change in multiple plasma analyte levels, as does virus reactivation after cART interruption. Furthermore, we find evidence for the involvement of TBG and neutrophils in the response to DC-vaccination in the setting of HIV-infection.

## Introduction

Chronic HIV infection is characterized by profound changes in immune function that, if left untreated, will result in AIDS. A gradual decline of CD4 T cells is a hallmark of untreated HIV infection, but other immune cells including CD8 T cells and B cells also have altered numbers and turnover dynamics [1,2]. At the functional level, a pathognomonic feature of HIV infection is generalized immune activation [3–5]. While combined antiretroviral treatment (cART) curbs viral replication and thereby reverses CD4 T cell decline, it does not completely restore immune function [2]. Indeed, it has recently been reported that cART is not distributed evenly throughout the body, resulting into low-level virus replication occurring in specific anatomical niches, such as lymph nodes and gut [6]. Alternatives to cART are therefore needed. Immune intervention strategies such as therapeutic vaccination could offer an attractive alternative for cART: with fewer side effects, no need for lifelong strict therapy adherence, better availability and cost reduction. Many therapeutic HIV vaccination studies have been conducted, some with encouraging results [7,8] as demonstrated by a reduction of plasma viral load (lower set point) or a prolonged time of therapy interruption [9]. In these therapeutic vaccination studies, a range of clinical and laboratory parameters was assessed to monitor vaccine safety, but no classical correlates of vaccine efficacy could be identified to guide next generation vaccine trials. Therefore, other readouts have been used to assess the effect of vaccination, including a decrease of the viral set point after therapy [8].

**Fig 1:**
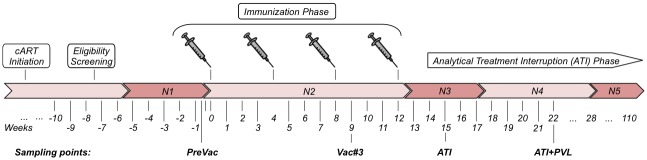
Study setup. Eligible HIV+ individuals were recruited into the study. They were given 4 autologous dendritic cell vaccinations at 4 week intervals, followed by analytical treatment interruption (ATI). Plasma analytes were measured before vaccination (‘PreVac’), after the third vaccination (Vac#3), just after commencing ATI, and when plasma virus loads become detectable again (ATI+PVL). The bar indicates the phases around the sampling points that were used for summarizing the neutrophil counts (N1-5).

In the phase I/IIa clinical trial “DC-TRN”, we vaccinated HIV-infected individuals with autologous dendritic cells expressing HIV regulatory proteins [10] prior to analytical cART interruption. DC-TRN was a non-randomized, non-blinded trial evaluating the safety and immunogenicity of DC-based immunotherapy in cART-treated HIV-infected patients. The clinical protocol of the trial has been detailed in Allard et al [10]. Briefly, 17 HIV-1 subtype B infected males, stable on cART, were vaccinated both subcutaneously and intra-dermally on four occasions with four-week intervals (Fig 1, for patient characteristics, please see Table 1). The vaccine consisted of autologous mature DCs electroporated with sig-Tat-DC-Lamp, sig-Rev-DC-Lamp or sig-Nef-DC-Lamp mRNA [11]. Two weeks after the last vaccination, patients were submitted to analytical treatment interruption (ATI). CD4 or CD8 T-cell responses as measured by *ex vivo* stimulation assays were used to assess the vaccine responses [10]. Additionally, the time off cART, which ranged from 3 months to 7 years, was used as a vaccine response variable as this approaches the ‘functional cure’ that immunotherapy aims to provide. Transcriptome analysis of peripheral blood mononuclear cells (PBMC) obtained from the DC-TRN trial participants revealed a profound vaccine induced, long lasting, transcriptome shift enriched for genes related to cellular immunity and general immune activation [12]. These results further corroborate the fact that DC-vaccination boosts the host response in HIV-infected individuals. In circulating PBMCs, however, transcriptome changes that were associated with vaccination success could not be identified.

**Table 1:** DCTRN trial participant characteristics.

Patient	Country	Gender	transmission	Age*	Year of HIV Diagnosis	Time before cART(weeks)	Nadir CD4 T cell count before ART	Time on cART (months)	current cART	Time on current cART (months)	Weeks until PVL>1000 c/ml after ATI	visit #8 days from imm #1	visit #11 days from ATI/ imm #4	visit #16 days from ATI	PVL at visit #16 c/ml	weeks off cART (ATI)
H001	BE	male	MSM	45	2001	5	404	64	3TC, AZT, ABC	41	6	ND	7/20	ND	98400	205
H002	NL	male	MSM	36	1992	224	440	121	3TC, AZT, ABC	72	6	64	7/22	53	100000	352
H003	NL	male	MSM	41	2000	32	370	76	3TC, TDF, NVP	28	3	64	7/21	54	100000	28
H004	NL	male	MSM	56	1988	442	400	127	3TC, NVP, AZT	79	4	63	7/21	63	5750	52
H005	NL	male	MSM	42	1993	151	650	133	3TC, NVP, AZT	67	3	63	7/21	56	16800	96
H006	NL	male	MSM	48	1997	2	450	119	3TC, NVP, AZT	67	4	56	7/21	56	14800	96
H007	NL	male	MSM	42	1998	26	340	108	3TC, TDF, NVP	56	8	63	7/23	56	100000	39
H008	NL	male	MSM	45	1997	124	310	91	3TC, NVP, ABC	38	2	63	7/21	54	43600	28
H009	BE	male	MSM	52	1991	248	326	142	3TC, NVP, ddI	47	4	ND	7/24	53	9300	181
H010	BE	male	MSM	45	1992	442	323	75	TDF, EFZ, ddI	43	6	ND	7/21	ND	100000	94
H011	BE	male	MSM	55	2001	25	555	69	3TC, AZT, ABC	52	6	ND	7/20	56	149	137
H012	BE	male	MSM	39	1993	423	304	73	TDF, EFZ, d4T	48	3	ND	7/22	54	27700	90
H013	BE	male	MSM	72	2000	27	660	78	3TC, NVP, AZT	65	6	ND	7/21	56	20000	179
H014	NL	male	MSM	37	1996	30	300	128	3TC, TDF, NVP	38	3	64	7/21	55	91200	29
H015	NL	male	MSM	42	2004	30	290	36	3TC, TDF, NVP	35	6	64	7/20	56	12600	50
H016	NL	male	MSM	56	1995	11	350	142	3TC, TDF, NVP	61	6	61	14/27	63	58000	117
H017	NL	male	MSM	50	1999	245	350	45	3TC, TDF, NVP	42	2	63	7/19	54	100000	50

* at immunization #1

MSM: men having sex with men

Plasma concentrations of analytes, including cytokines, chemokines, and hormones, reflect regulatory processes of inflammatory events and may also be affected by the host response to HIV infection. Previous studies have utilized plasma analyte levels to study inflammatory processes in a variety of infectious diseases, including dengue [13], hepatitis B [14] and acute and chronic HIV infection [5,15–17]. The advantage of measuring analytes is that they represent the primary regulatory mediators in the blood of patients and are therefore well suited for investigating immune-regulatory changes in response to HIV infection and DC-vaccination.

In the present study, we investigate the effect of therapeutic DC-vaccination in the setting of chronic HIV-1 infection [10,12] on plasma analyte levels. We use ‘time off cART’ as a surrogate marker of vaccine success, and relate it to changes in plasma analyte levels. The protein analyte profiling data demonstrate induction of cellular immunity and the upregulation neutrophil-related factors in HIV infection and DC vaccination. Therefore, we also investigate neutrophil counts and we show that they also associate with time off cART. Furthermore, we study plasma analyte level changes induced by HIV infection on cART, and the effect of cART treatment by itself and viral rebound on these analyte levels.

## Materials and methods

### Patients and controls

Seventeen patients chronically infected with HIV-1 subtype B on stable cART and with undetectable plasma viral load (PVL) were recruited in the DC-TRN trial [10] (Netherlands trial registry NTR2198; Table 1). All patients were Caucasian men who have sex with men (MSM). Patients co-infected with HBV, HCV were excluded from the study. Before inclusion, all patients provided written informed consent, in accordance with the regulations of the institutional ethical committees at the University Hospital Brussels and Erasmus Medical Centre Rotterdam (VUB 05–001 and MEC 2005–227). As controls, peripheral blood samples of ten healthy adults (anonymized laboratory personnel) from the same region and of the same age range were included in the study. In addition, three healthy individuals receiving cART in the context of post-exposure prophylaxis (PEP) treatment administered after a possible transmission event, were collected. They were all treated with Combivir (3TC, AZT) and Kaletra (LPV/r). They were in the same age range as controls and vaccinees. No CD4 counts have been determined and no seroconversion has been documented after PEP. Plasma samples were isolated from EDTA tubes (Becton Dickinson, Breda, The Netherlands) by centrifugation and processed the same day for storage at -80°C.

### Analyte levels measurements and processing

Plasma samples were sent frozen to Myriad RBM and multiplex immunoassay panel Human DiscoveryMAP^®^ 250+ v2.0 (Myriad RBM, Texas, USA) was used at the Clinical Laboratory Improvement Amendments (CLIA)-certified laboratory for measuring the levels of multiple analytes in plasma of trial subjects and controls. The multiplex assay comprised measurement of cytokines, chemokines, growth factors, hormones, metabolic markers, angiogenesis markers, tissue remodelling proteins, acute phase reactants, cancer markers, kidney damage markers, CNS biomarkers and other circulating proteins. In total, 243 analytes were quantified (see supporting information) using duplicate 8-point standard curves and 3 control samples at low, medium and high concentrations run in duplicate for each assay. Analytes with similar concentrations were combined into specific multiplexes and measured simultaneously. Concentrations were interpolated from four or five parameter logistic curves. Analytes showing no changes in any sample were excluded, leaving 207 analytes for analysis (S1 Fig, S1 Table). Plots for all analytes are provided in the supplementary material as a resource accompanying this paper (S1 File).

### Data processing

Analyte analysis was performed in R, an environment for statistical computing [18]. All analyte concentrations were log-transformed before analysis. Initial analysis indicated an effect of the clinical center, separating the Belgian and Dutch samples. This center (batch) effect was removed with ComBat [19]. Differential analyte expression analysis was performed using limma [20]. Heatmaps of analyte expression were constructed by hierarchical clustering using correlation distance and Ward’s linkage [21]. For cluster analysis, several sample selections from the primary dataset were studied: a dataset including, respectively, all samples; samples from trial participants only; and a trial participants-only set from which the intra-individual analyte patterns have been removed with the removeBatchEffect function from the limma package [20].

### Statistical analysis

Statistically significant analyte level differences between groups of patients and at the indicated time points (*i*.*e*., before, during and after DC vaccination and at treatment interruption) were performed using limma [20]. Analytes were considered differentially expressed whenever a false discovery rate (FDR) of < 0.05 and at least a 1.5-fold up- or down-regulation between groups was observed.

A Cox proportional hazards model was used to assess whether any analytes are associated with the long-term outcome of DC vaccination. The outcome variable was ‘time off cART’ and analyte measurements at PreVac (week 0; Fig 1), ATI (week 15) and ATI+PVL (week 22) were time dependent predictors. Note that because of the correlation between these three time dependent predictors, we tested the combined association of the predictors with time off cART by performing a Chi-square test to compare the prediction models with and without the analyte measurements at the three time points. The analyte measurements at Vac#3 were excluded from the analysis, as no plasma sample was available for six patients. In addition, the analysis was restricted to 15 of the 17 patients who had analytes measured at these three time points. We conducted multiple imputation for missing analyte measures under the null hypothesis [22] for 20 analytes (11 with less than 5% missing, 6 with 5–10% missing and 3 with 10–13.3% missing, S2 Table) and used ComBat to remove batch effects.

### Neutrophil count analysis

Blood absolute neutrophil counts (ANC) were routinely performed at multiple time points for all patients. To obtain a robust estimate of ANC, the counts were stratified into the five relevant study phases of the trial (N1 –N5, Fig 1) and summarized using the median value. Regression analysis was performed to assess the association between ANC and time off cART. Removal of any single sample did not affect the outcome of this analysis, nor did the addition of single factor covariates including ANC at other time points and origin of patients.

## Results

### HIV infection but not cART treatment affects global analyte levels

Blood plasma was collected from HIV patients at various time points before, during and after DC immunotherapy (see Fig 1), as well as from healthy controls (HC) and individuals treated with cART as post-exposure prophylaxis (PEP). Samples were annotated by trial stage, *i*.*e*., cART-treated HIV-infected individuals with pVL below the lower limit of detection (<50 copies/ml) sampled before vaccination (‘PreVac’); one week after the third administration of the DC-vaccine (while patients are still under cART, ‘Vac.3’); one week after analytical treatment interruption (ATI); and 10 weeks after ATI, once patients had become viraemic (‘ATI.PVL’, Fig 1). Following ATI, the median time to viral rebound was 3.1 weeks and pVL increased to >3 log_10_ copies/ml after a median of 4.7 weeks [10]. For DC-TRN trial participant details, see Table 1.

**Fig 2:**
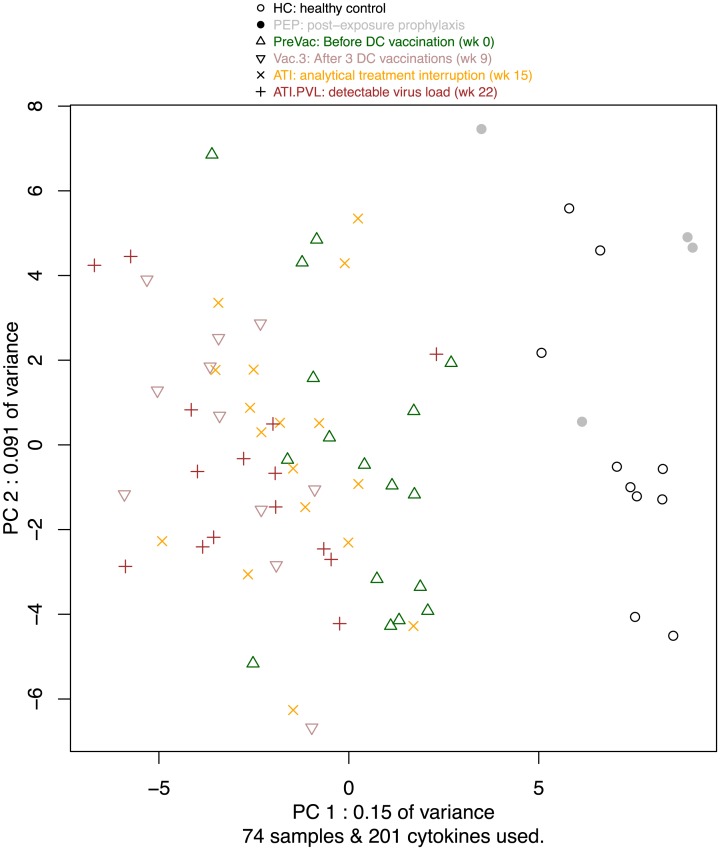
PCA of all samples. Missing values were removed by either excluding the analytes that contain missing values, or patients that have missing values.

To obtain a bird’s eye view of the analyte expression data, we perform principle component analysis. The first principal component identifies HIV infection as the main source of variability, separating the samples into two groups representing respectively HC/PEP and HIV patients (Fig 2). This analysis suggests small effects, if any, of cART on the analyte levels, since the samples from the HIV-infected study participants do not aggregate by cART treatment, and since the PEP samples are located firmly in the HC group. PCA indicates that PreVac tends to be more similar to HC than samples from the other phases of the trial. Samples from the Vaccination and ATI groups are intermingled, whereas samples taken when the viral load rebounded tend to be located on the left of PC1. Taken together, this analysis indicates that there is a difference in analyte expression between HIV-positive and HIV-negative individuals, regardless of vaccination, therapy or virus reactivation.

### Samples cluster by trial stage after correcting for intra-individual effects on analyte profiles

**Fig 3:**
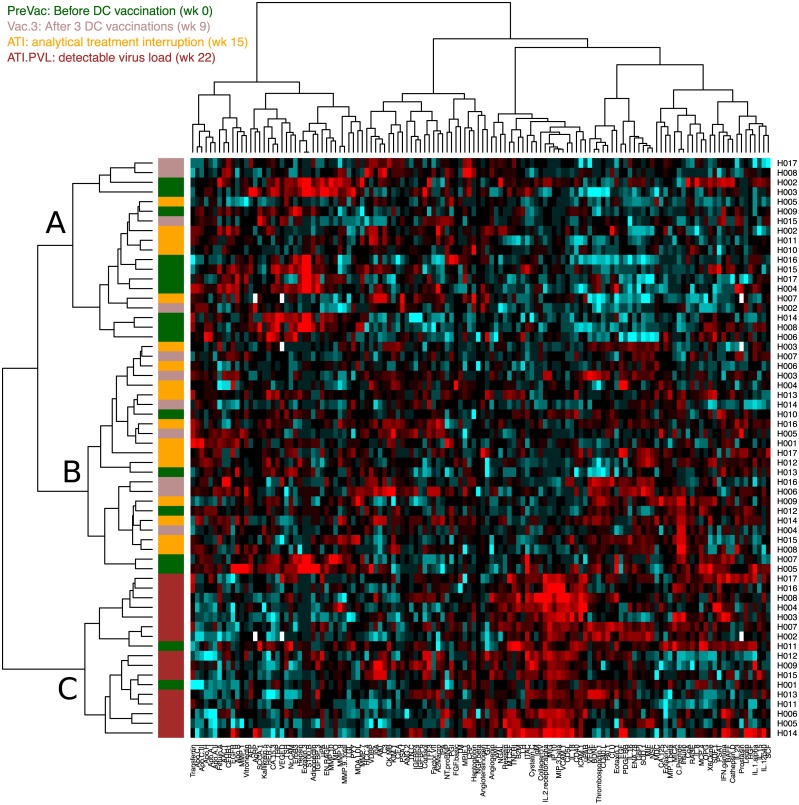
Heatmap of analyte measurements in DC-TRN trial participants. All measurements were corrected for patient effect. Three sample clusters are observed, which have been labelled A-C. The DCTRN trial stage at which the sample was taken in indicated by the left-most colour bar.

In order to evaluate the effect, if any, of the DC-TRN trial stages on the analyte expression pattern, we applied cluster analysis to the HIV+ (i.e., DCTRN trial) samples only [21,23]. A consistent clustering of samples originating from the same patient is noticeable (S2a Fig). In the initial cluster analysis, no clustering by vaccination or viraemic status (S3 Fig) is observed. However, upon mathematical correction for the individual specific effect on the analyte profile, cluster analysis reveals a clustering that relates to the phase and status in the vaccination trial (Fig 3). Clusters A, B, and C contain primarily PreVac samples, samples taken after vaccination or after ATI, and samples collected during viral rebound, respectively. The data reveal little effect of cART interruption as we see no segregation based on cART status (see Table 1). In summary, this analysis shows that virus reactivation induces the largest change in analyte expression within the DC-TRN trial participants.

### Markers of long-term outcome

Previous analyses of data obtained from this trial, including B and T cell responses and PBMC transcriptome analysis [10,12,24], did not reveal a biomarker for the time off cART. Time off cART may not be the optimal correlate of protection as therapy can be biased by physician or patient appraisal. However, some study participants control the virus and maintain CD4+ T cell numbers for a considerable period of time without cART. Therefore, we use “time off cART” as an outcome in the identification of biomarkers among the plasma analytes. To this end (see Table 1), we used a Cox proportional hazards model with time off cART as outcome variable and analyte measurements at PreVac (week 0; Fig 1), ATI (week 15 from the first vaccination) and ATI+PVL (week 22) as predictor variables. A longer time off cART is significantly associated with Thyroxine-Binding Globulin (TBG; *P* = 2.66x10^-4^ and when corrected for multiple testing *P* = 0.0423, see S3 Table). To better understand the relationship between TBG and long-term outcome, we examined the levels of TBG as a function of time off cART (S4 Fig). Although the levels of TBG in the DCTRN trial participants are in the same range as observed in healthy controls, we notice that participants that have a long time off cART period tend to exhibit a different *change* in TBG levels that the other participants. In participants that resumed cART early, TBG levels are highest at virus reactivation (i.e., study week 22); in participants that remain off cART for a longer time, TBG levels tend to be highest at the ATI stage (i.e., study week 15). A further 30 analytes have uncorrected *P* < 0.05 and are considered to be worth further study (S3 Table).

### Differential expression of HIV-specific factors in patients stable on cART

**Fig 4:**
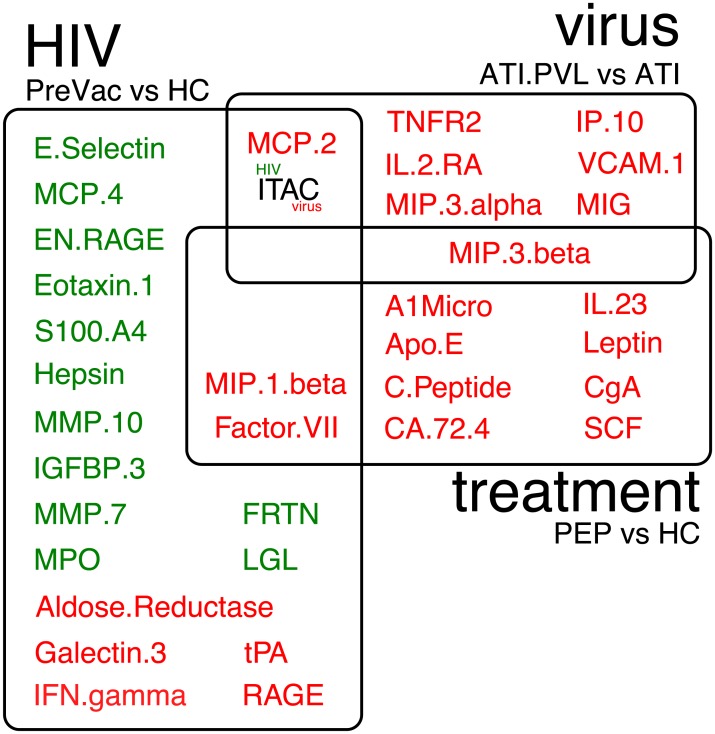
Differential expression analysis of analytes. The names of analytes that are differentially expressed are depicted in circles for HIV infection stable on treatment vs healthy controls (‘HIV+’), cART treatment being treated HIV negative vs health control (‘treatment’) and virus reactivation as seen after stop cART, i.e., ATI.PVL—ATI (called ‘reactivation’). Up- and down-regulated analytes are depicted in red or green, respectively. ITAC is up-regulated during virus reactivation and down-regulated in during HIV infection.

In addition to identifying biomarkers associated with a longer time off cART, we also examine associations between changes in analyte concentration during the trial and other independent clinical variables. By statistical modelling using limma, we are able to identify analytes that specifically and significantly associate with each of the trial stages. The largest number of significant differentially expressed analytes is found for the HIV-treated—HC difference. Additionally, we identify analytes that were differentially expressed as a result of cART treatment and virus reactivation (Fig 4).

HIV infection is associated with an increase in IFNγ levels, which is a marker of an increased cellular immune response. In addition, HIV infection is associated with a decrease in S100 protein family members S100A12 (EN-RAGE), S100A4, and a small decrease in S100B. RAGE, which is a receptor for S100B and is expressed on neutrophils [25] is up-regulated. Overall, more analytes are down-regulated than are up-regulated. The differences between HIV-on-cART individuals and healthy controls can mostly be attributed to HIV-induced changes, because the effect of cART in HIV-negative individuals affects other analytes (Fig 4), although these results should be interpreted with caution given the small number of PEP subjects.

### cART up-regulates markers of insulin resistance

Enrolment of both HIV-infected and non-HIV-infected individuals receiving cART allows the examination of the effect of cART independent of HIV infection. cART is associated with an increase in both the T-cell associated chemotactic cytokines MIP1β (CCL4) and MIP3β (CCL19) (Fig 4). CgG (Chromogranin-A) promotes the secretion of insulin from beta-islets and is also increased in samples taken under cART-treatment. Furthermore, levels of C-peptide, a cleavage product of insulin, and leptin were increased in cART-treated individuals. This effect on blood glucose regulation that is indicative of HIV metabolic syndrome that may lead to diabetes mellitus and cardio-vascular diseases [26]. cART treatment is also associated with an increase in MIP1β that is produced by CD8+ T cells and has strong anti-HIV activity [27].

### Active HIV replication induces CXCR3 ligands

Viral rebound as a result of productive HIV replication after cART interruption induced several changes in analyte levels. We observe an increase in MCP-2 (CCL8) (Fig 4), which is a potent HIV inhibitor by virtue of its strong affinity for the HIV co-receptor CCR5 [28]. MIP3β (CCL19), which attracts DCs, B and T cells towards lymph nodes via binding of its receptor CCR7, is up-regulated. Virus replication is also associated with an increase in MIG (CXCL9), IP-10 (CXCL10), and I-TAC (CXCL11). These chemokines are all induced by IFNγ, and are all ligands for chemokine receptor CXCR3, which mediates T-cell trafficking to lymphoid and mucosal tissues. Both IP-10 and I-TAC are known to be up-regulated by HIV replication [29,30]. Furthermore, there is evidence for a role of IP-10 and CXCR3 in the integration of HIV in resting CD4+ T cells that promotes virus persistence within the host [31]. Our results show that MIG expression is higher in samples taken during active virus replication.

### Late-stage granulocyte counts associate with duration of cART-free period after DC vaccination

No analytes are significantly differentially expressed in plasma upon DC vaccination. However, five analytes, MPO, RANTES (CCL5), GRO-α (CXCL1), NSE, and Matrix Metalloproteininase (MMP)-10, of which three are associated with granulocytes, display a borderline increase (FDR < 0.1) upon vaccination. This prompted us to evaluate the association between time off cART and the number of neutrophils in circulation by analysing the absolute neutrophil counts (ANC) of our subjects during the trial.

**Fig 5:**
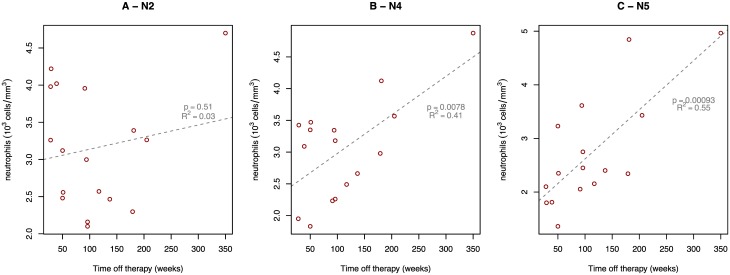
Neutrophil counts over the course of the trial. All data available for all patients is shown. The DC-TRN trail phases are indicated in Fig 1. The regression lines are annotated with significance levels and R-squared values.

To perform a global trend analysis of the variable ANC, counts are summarized by trial period using median averaging. By evaluating the neutrophil counts at several study phases, we can attribute the change in circulating neutrophil levels to different factors. By fitting a regression model of neutrophil counts during the various phases of the trial (Fig 1, N1-N5), we observe that late-stage neutrophil counts associate best with time off cART (Fig 5). The results support the involvement of neutrophils in the response to HIV and to DC vaccination in a chronic HIV-setting.

## Discussion

In this study, we profile the response of chronically HIV-infected individuals to DC-vaccine-mediated immunotherapy with a high-throughput assay quantifying over 200 analytes in peripheral blood. With this approach, we are able to directly monitor regulatory molecules that circulate in the periphery, irrespective of the tissue source, rather than monitoring host response mediators such as mRNA expressed in circulating lymphoid cells. Using both the DC-vaccinated DC-TRN study subjects as well as healthy controls and post-exposure prophylactically treated individuals (PEP), we are able to detect changes in regulatory molecules in response to HIV-infection, DC vaccination, cART treatment and virus reactivation during cART interruption. Plasma analyte profiling revealed DC-vaccination and viral rebound associated responses characterized by immune activation and involvement of neutrophils.

Overall analyte profile changes, as visualized by principal component analysis, demonstrated that the profiles from healthy controls (HC) and HIV negative individuals on cART (PEP) clearly differ from HIV-infected individuals, even when virus cannot be detected in plasma. Interestingly, only upon adjustment for individual (study participant) specific analyte expression levels, a superimposed pattern discriminating pre-vac, early post-vac and late post-vac analyte profiles could be identified by PCA (S2B Fig) and cluster analysis (Fig 3). Viral rebound upon ATI, but not cART interruption itself, results in significant analyte profile changes. Previously, PBMC samples collected from the same study participants were subjected to mRNA profiling [12]. Principle component analysis of the resulting blood transcriptome profiles revealed a similar marked difference between healthy individuals and chronically HIV infected individuals. Therapeutic vaccination however had a dominant effect at the mRNA level. In addition, the DC-vaccination induced transcriptome shift persisted after ATI. Unlike the analyte profiling results presented here, the major transcriptome shift induced by DC-vaccination was not affected by the subsequent viral rebound. This shows that analyte- and mRNA profiling provide different and complementary information on the host response.

Among the analytes that are differentially expressed in response to HIV infection, several members of the S100 protein family are down-regulated, including EN-RAGE (S100A12), S100A4 and S100B. The S100 protein family consists of 24 members that have a wide variety of functions, including growth, development, inflammation and DAMPs. EN-RAGE is constitutively expressed in neutrophils and its expression causes grow arrest in epithelial cells. S100A4 and S100B are both known to interact with epidermal growth factor. The down-regulation of these three proteins suggests that epithelial growth is promoted, both through a release of epithelial cell growth factor by a decrease in EN-RAGE, and reduced sequestering of epithelial growth factor by down regulation of S100B and S100A4 [25]. Our results indicate that the role of S100 family proteins in HIV pathogenesis warrants further investigation.

Study stage-associated differential analyte expression analysis identified the differential expression of analytes involved in blood sugar regulation, including up-regulation of C-peptide, CaG and leptin during cART therapy, independent of HIV infection. Although this comparison is based on three versus ten individuals and should therefore be interpreted with caution, up-regulation of these analytes is consistent with earlier findings that cART induces insulin resistance. In fact, insulin resistance is one of the main complications of prolonged cART therapy, especially in HIV-positive individuals over the age of 50 [32]. Both these findings illustrate the added value of plasma analyte profiling in antiviral strategy evaluation studies. Since time off cART ranged widely (28–352 weeks), application of techniques such as mass spectrometry enabling the quantification of a much larger set of plasma analytes may result in the identification of additional biomarkers of favourable host responses to therapeutic vaccination, especially when applied in more extended follow-up studies.

Although this phase I/IIa study is not designed for biomarker analysis, we were nevertheless able to identify the plasma protein thyroxine-binding globulin as a potential biomarker of the efficacy of DC vaccination in a chronic HIV setting. TBG associates with a longer period off therapy; it appears that this is primarily due to a difference in how DCTRN trial participants react to virus reactivation. Most participants that remain off therapy for a prolonged period of time have a down-regulation of TBG following virus reactivation, whereas others have an up-regulation. TBG is a regulator of that, along with transthyretin and serum albumin, is responsible for carrying the thyroid hormones (thyroxine (T_4_) and 3,5,3’-triiodothyronine (T_3_)) in the bloodstream. A change in TBG levels therefore leads to a shift in T4 or T3, which suggests that patients that remain off therapy for a prolonged period of time have a different metabolic response to reactivation of virus. Since TBG is considered a marker for multiple metabolic and oncogenic abnormalities and thought to have a minor role in HIV [33], further study is needed to establish its use adand relevance. Several other markers that do not reach statistical significance after multiple testing correction are considered to be worth further study also (S3 Table). Markers that stand out include IL8, ENA78 and other neutrophil-related cytokines. Furthermore, markers of metabolic regulation are also related to time off cART, which could reflect a rebound in metabolic status due to the prolonged absence of cART treatment.

A considerable number of analytes that are differentially expressed at various stages of the trial are related to neutrophil regulation. This prompted us to enumerate the number of circulating neutrophils in our trial. Despite a considerable variation in neutrophil counts, neutrophil numbers correlate positively with a longer time off cART after DC vaccination. Our analysis does not indicate a causal relationship between higher ANC and a longer period off cART, since the ANC collected during the last phase of the trial correlated best with time off cART. However, in our study, multiple analytes attracting neutrophils are induced by DC-vaccination, suggesting that the neutrophils are indeed specifically induced. Additionally, TBG, which we found to be related to time off cART, is cleaved by elastin, a protein that is produced by neutrophils in response to inflammation. This leads to a localized release of T4 that mediates more energy expenditure around sites of inflammation [34]. The decrease of TBG at late timepoints could therefore be related to the increase of inflammation and neutrophils that we observe. However, there is no significant difference in inflammatory cytokine expression between early and late therapy resumers, suggesting that any differences in inflammation are small.

Omics profiling approaches support vaccine response monitoring [35,36], especially in difficult vaccine evaluation settings like therapeutic vaccination of a chronic infection [12]. These high-resolution approaches may reveal host response differences that are easily missed when solely applying traditional immunological and virological tools, in particular when these responses are affected by patient-specific factors. We show that combining multiple approaches provides different perspectives on the same process; for instance, the role of neutrophils has not been noticed before in earlier transcriptome profiling studies since PBMCs (*i*.*e*., blood cell fraction devoid of neutrophils) were analysed [12]. Combining all of these with integrated systems biology analysis approaches supports the identification of host correlates of sustained viral suppression and the identification of host response biomarkers to guide a booster vaccination scheme for therapeutic vaccines.

## Supporting information

S1 Fig Distribution of cytokine expressions. A—38 cytokines show little or no variance and have been removed from the dataset. B—Distribution of cytokine levels in a sample. No samples need to be removed as a result of insufficient quality.
(EPS)

S2 Fig Patients have strong individual cytokine profiles that can be partially corrected. Principle component analysis of samples from HIV-infected individuals only before (A) and after (B) removal of the patient effect.
(EPS)

S3 Fig Heatmap of analytes from all samples. Samples from the same patient tend to cluster together.
(EPS)

S4 Fig Association of TBG expression with time off therapy. Vertical grey lines connect samples from a single patient. The dotted diagonal lines represent regression lines that represent time off treatment vs TBG expression at specific trial stages.
(EPS)

S1 Table Analyse measurements for all samples. This table contains the primary analyte data that this study is based on.
(XLSX)

S2 Table Analyte quality control. Number and proportion of missing values in the analytes that passed quality control.
(XLSX)

S3 Table Details of Cox proportional hazards model. List of analytes that have borderline significance and are considered to be worth further study.
(XLSX)

S1 File Group-wise expression of all analytes. Boxplots of analyte expression per group for every analyte that passed quality control.
(PDF)
