# Supplementary material for: Dendritic cell immunotherapy followed by cART interruption during HIV-1 infection induces plasma protein markers of cellular immunity and neutrophil recruitment
Source: PLoS One. 2018 Feb 1;13(2):e0192278. doi: 10.1371/journal.pone.0192278 (PMC5794189; doi:10.1371/journal.pone.0192278)
Supplement: S1 File — Boxplots of analyte expression per group for every analyte that passed quality control. (PDF) [file pone.0192278.s008.pdf]

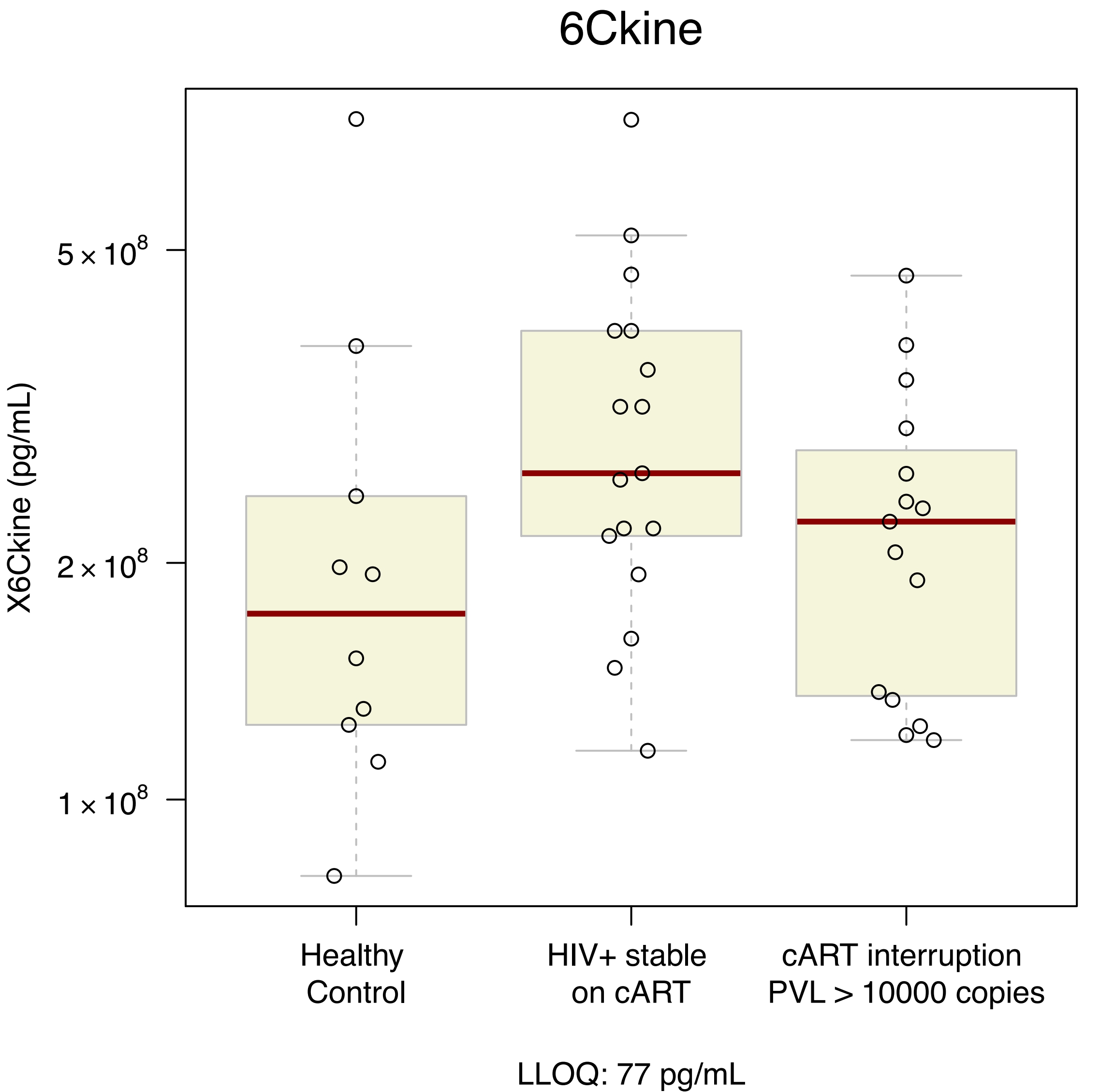
6Ckine
X6Ckine (pg/mL)
5×10^8
2×10^8
1×10^8
Healthy Control
HIV+ stable on cART
cART interruption PVL > 10000 copies
LLOQ: 77 pg/mL

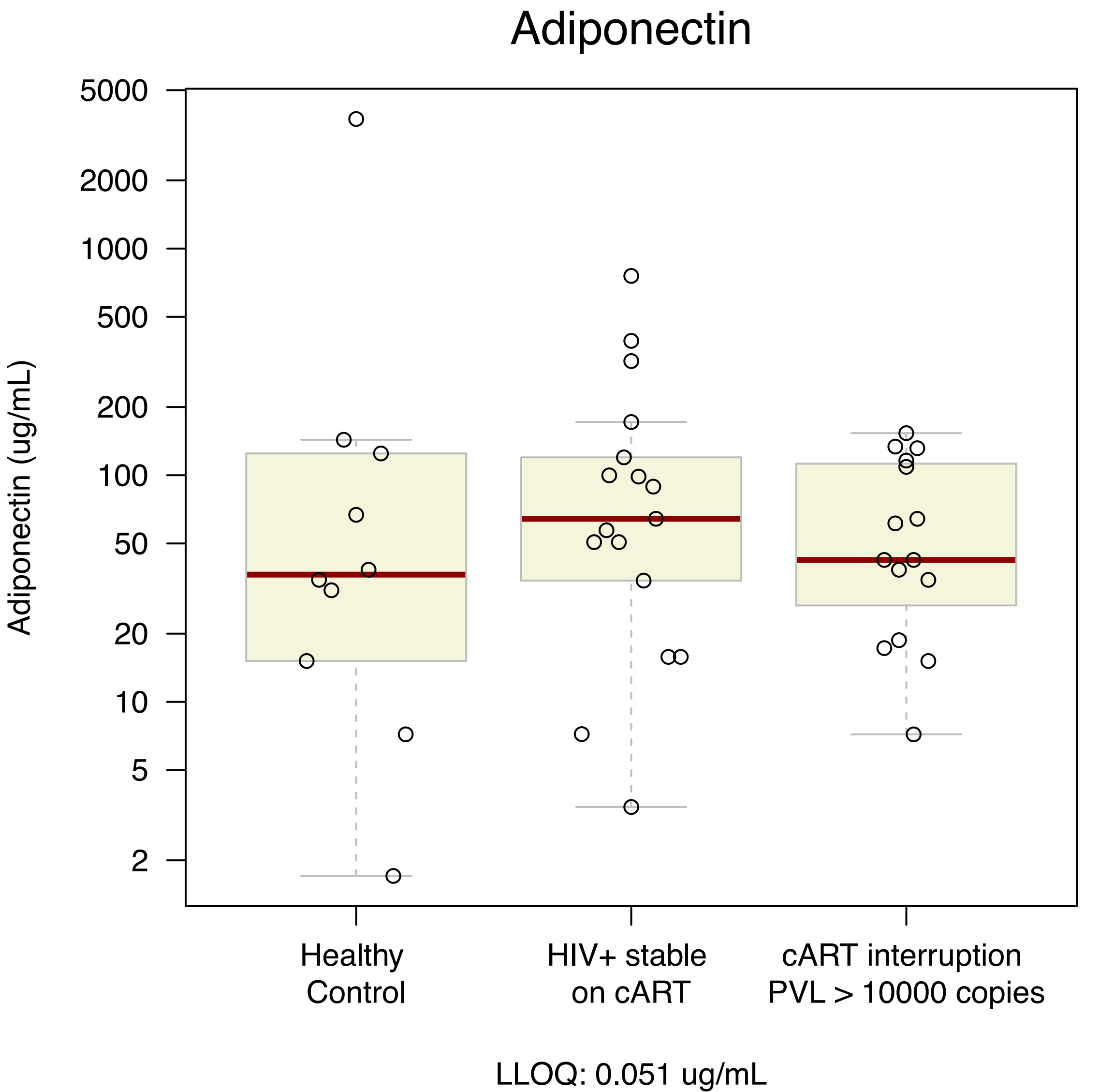
Adiponectin
Adiponectin (ug/mL)
5000
2000
1000
500
200
100
50
20
10
5
2
Healthy Control
HIV+ stable on cART
cART interruption PVL > 10000 copies
LLOQ: 0.051 ug/mL

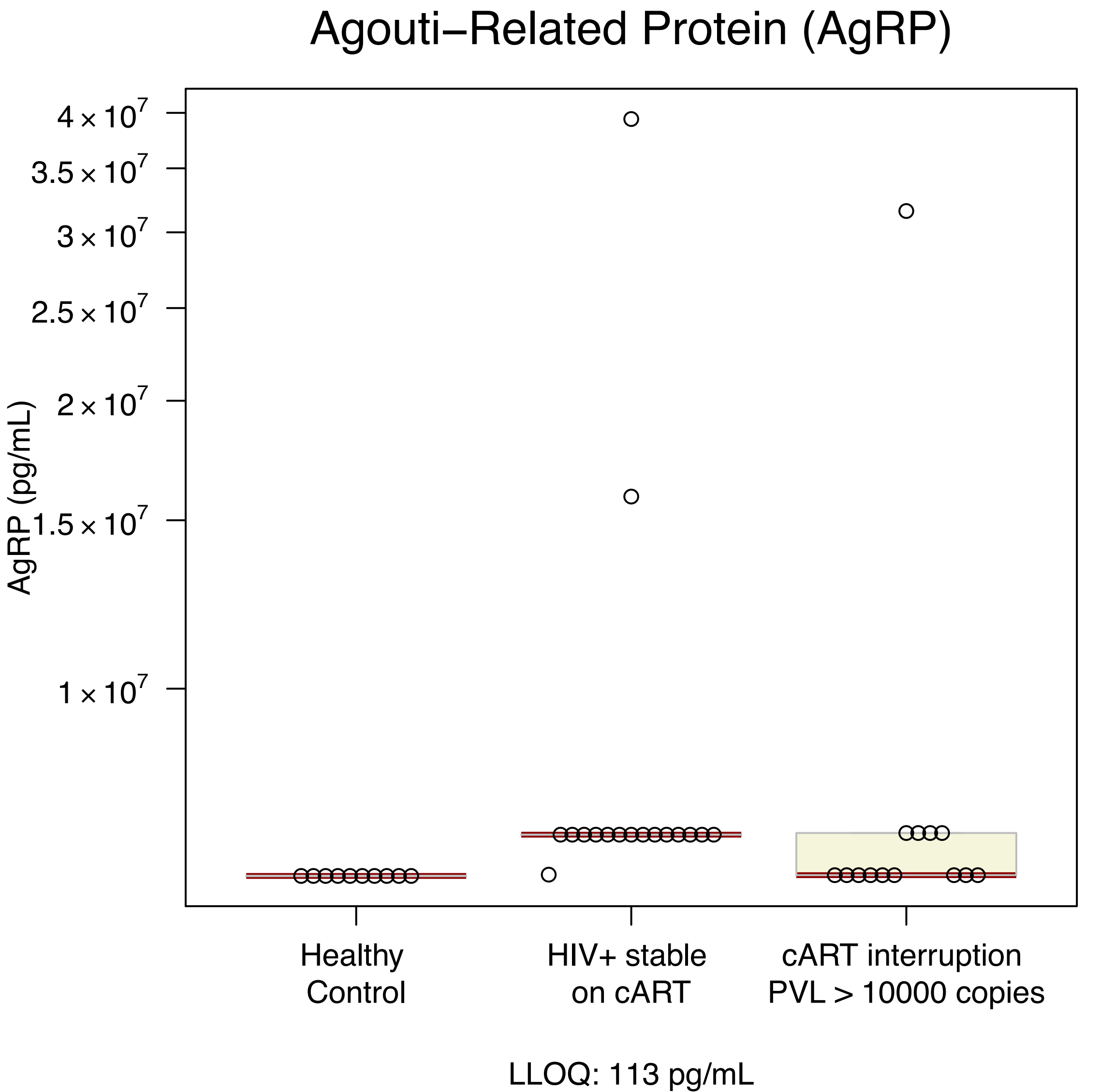

Agouti–Related Protein (AgRP)
AgRP (pg/mL)
4 × 10^7
3.5 × 10^7
3 × 10^7
2.5 × 10^7
2 × 10^7
1.5 × 10^7
1 × 10^7
Healthy Control
HIV+ stable on cART
cART interruption PVL > 10000 copies
LLOQ: 113 pg/mL

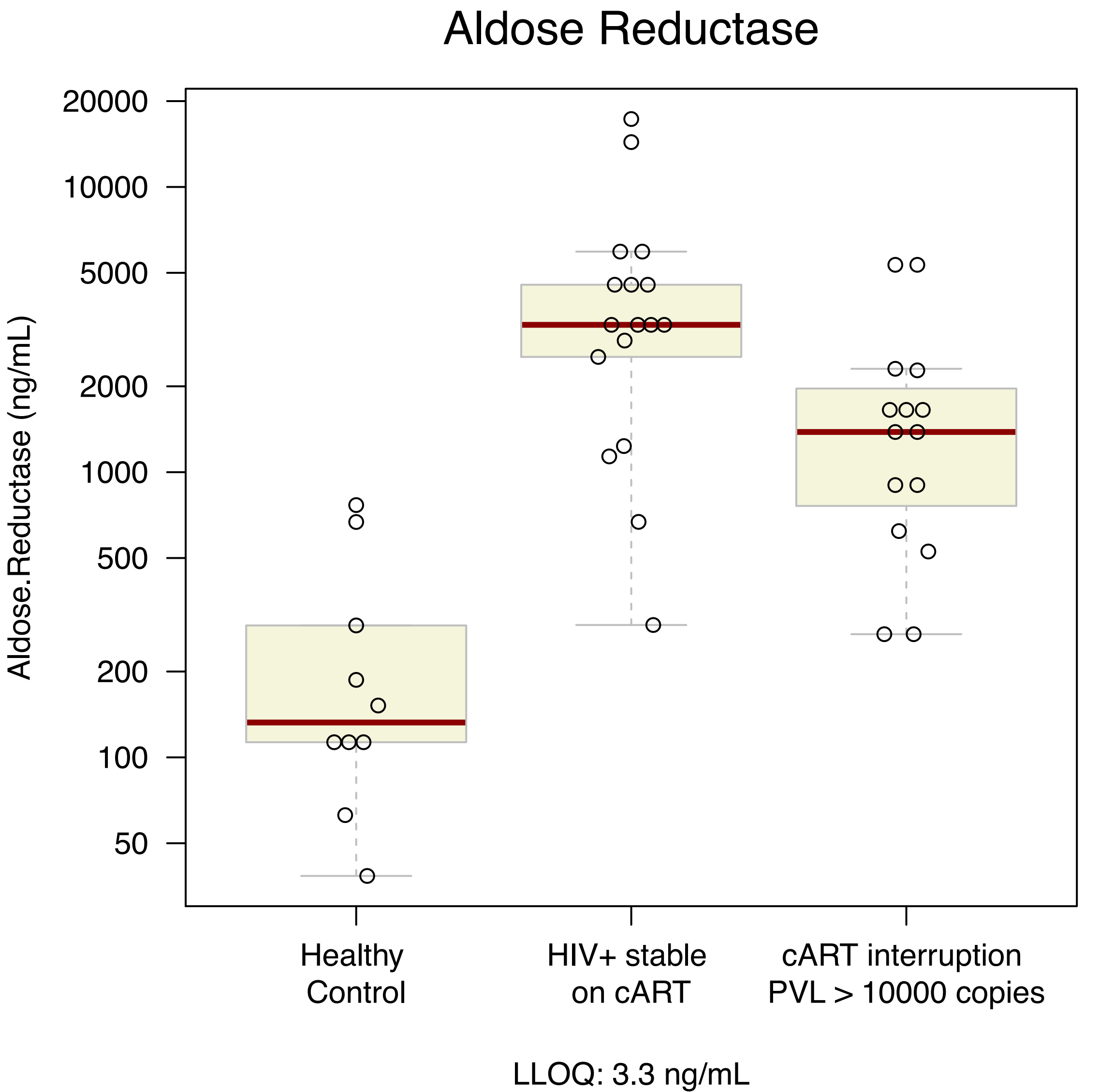

Aldose Reductase
20000
10000
5000
2000
1000
500
200
100
50
Aldose.Reductase (ng/mL)
Healthy Control
HIV+ stable on cART
cART interruption PVL > 10000 copies
LLOQ: 3.3 ng/mL

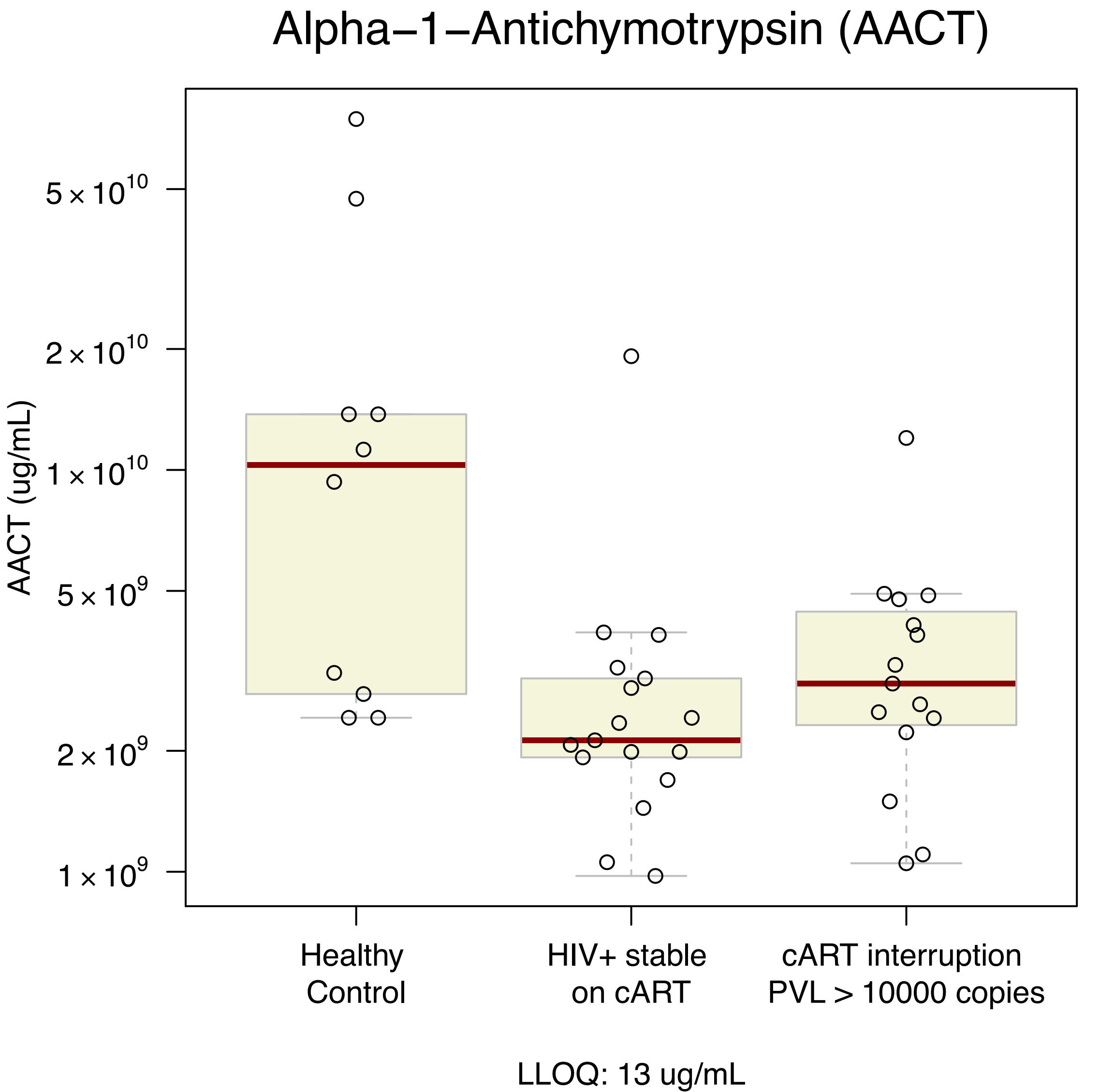
Alpha−1−Antichymotrypsin (AACT)
AACT (ug/mL)
5 × 10^10
2 × 10^10
1 × 10^10
5 × 10^9
2 × 10^9
1 × 10^9
Healthy Control
HIV+ stable on cART
cART interruption PVL > 10000 copies
LLOQ: 13 ug/mL

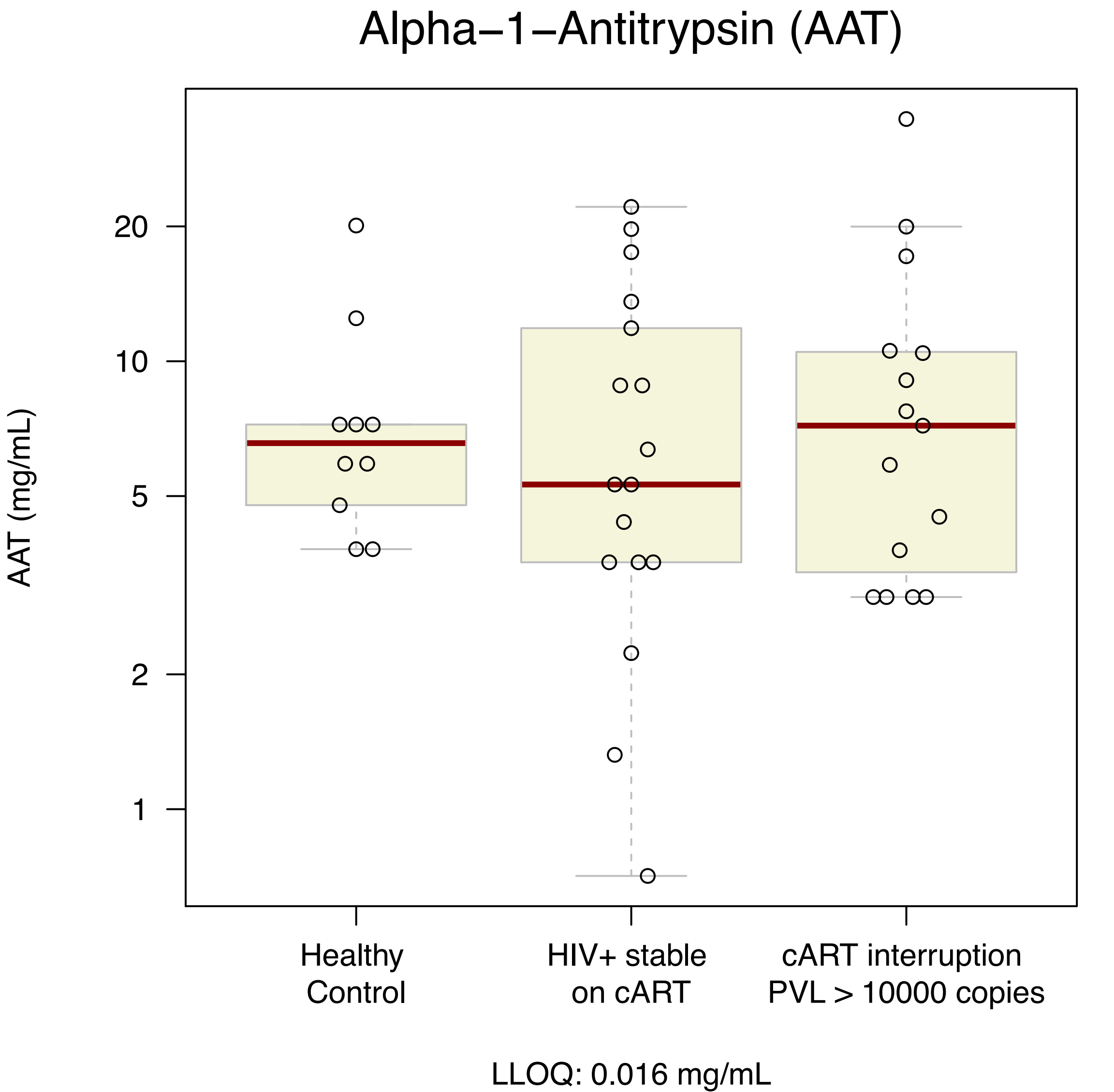
Alpha–1–Antitrypsin (AAT)
AAT (mg/mL)
20
10
5
2
1
Healthy Control
HIV+ stable on cART
cART interruption PVL > 10000 copies
LLOQ: 0.016 mg/mL

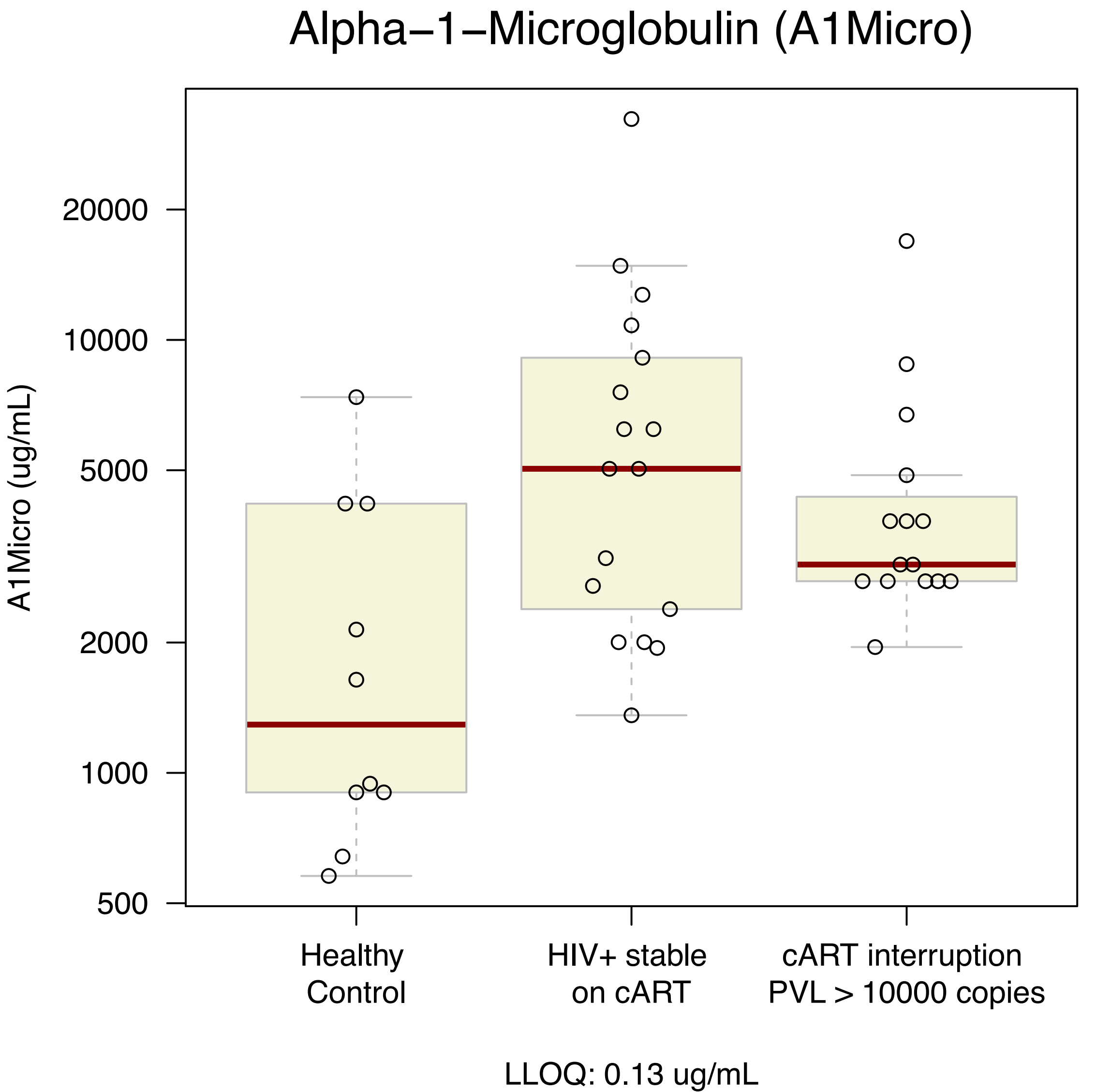
Alpha−1−Microglobulin (A1Micro)
A1Micro (ug/mL)
20000
10000
5000
2000
1000
500
Healthy Control
HIV+ stable on cART
cART interruption PVL > 10000 copies
LLOQ: 0.13 ug/mL

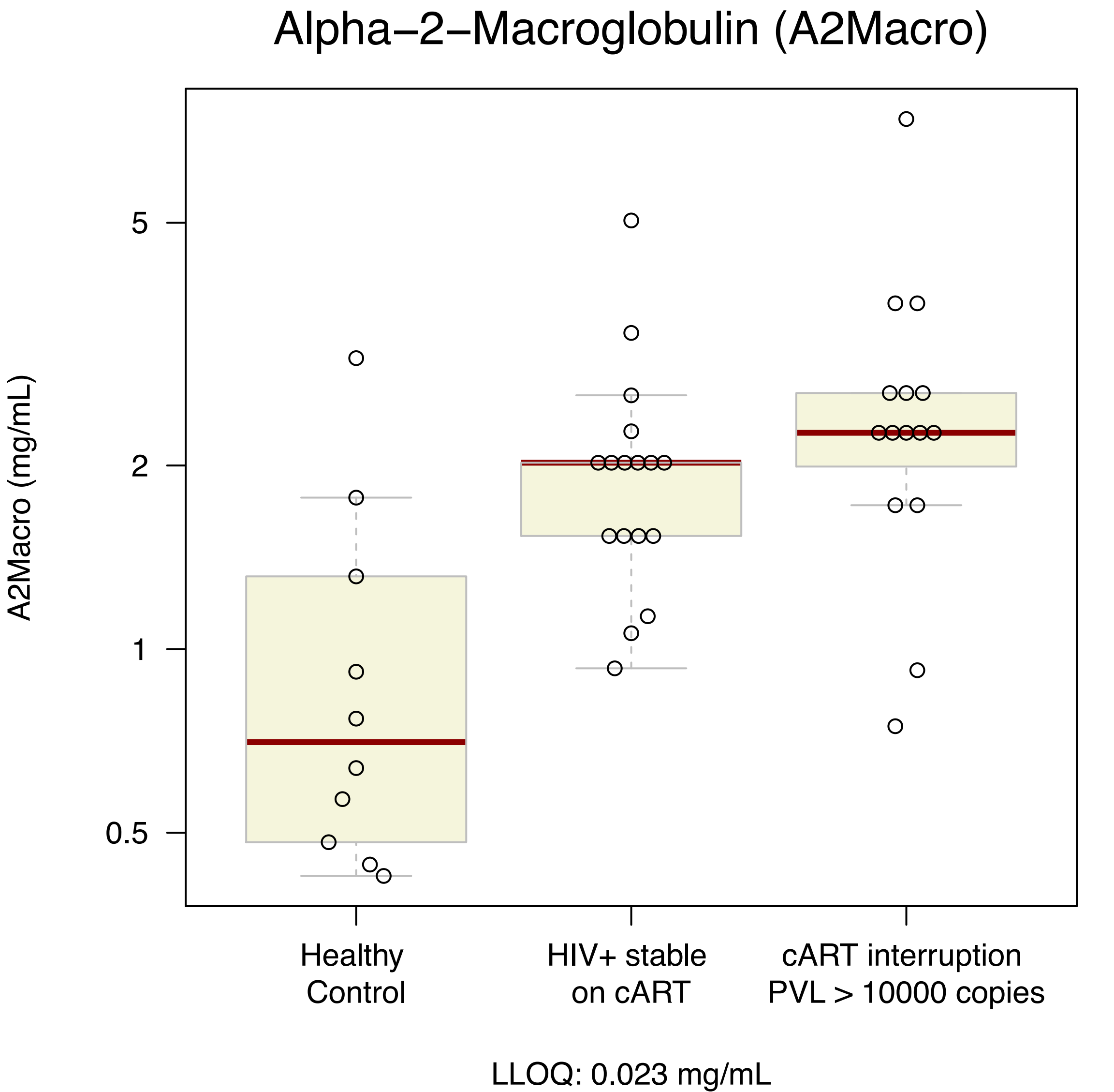
Alpha−2−Macroglobulin (A2Macro)
A2Macro (mg/mL)
5
2
1
0.5
Healthy Control
HIV+ stable on cART
cART interruption PVL > 10000 copies
LLOQ: 0.023 mg/mL

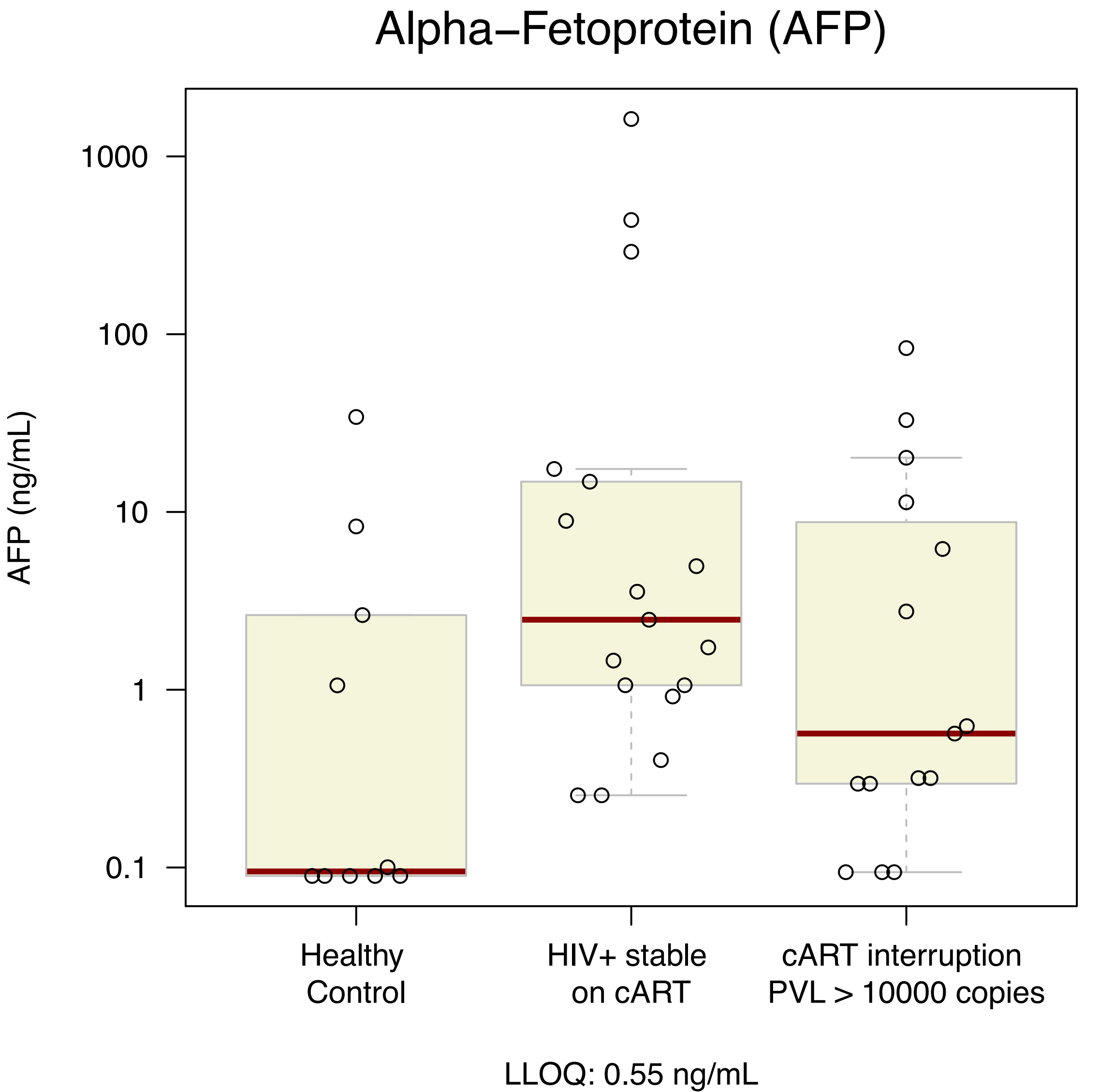

Alpha–Fetoprotein (AFP)
AFP (ng/mL)
1000
100
10
1
0.1
Healthy
Control
HIV+ stable
on cART
cART interruption
PVL > 10000 copies
LLOQ: 0.55 ng/mL

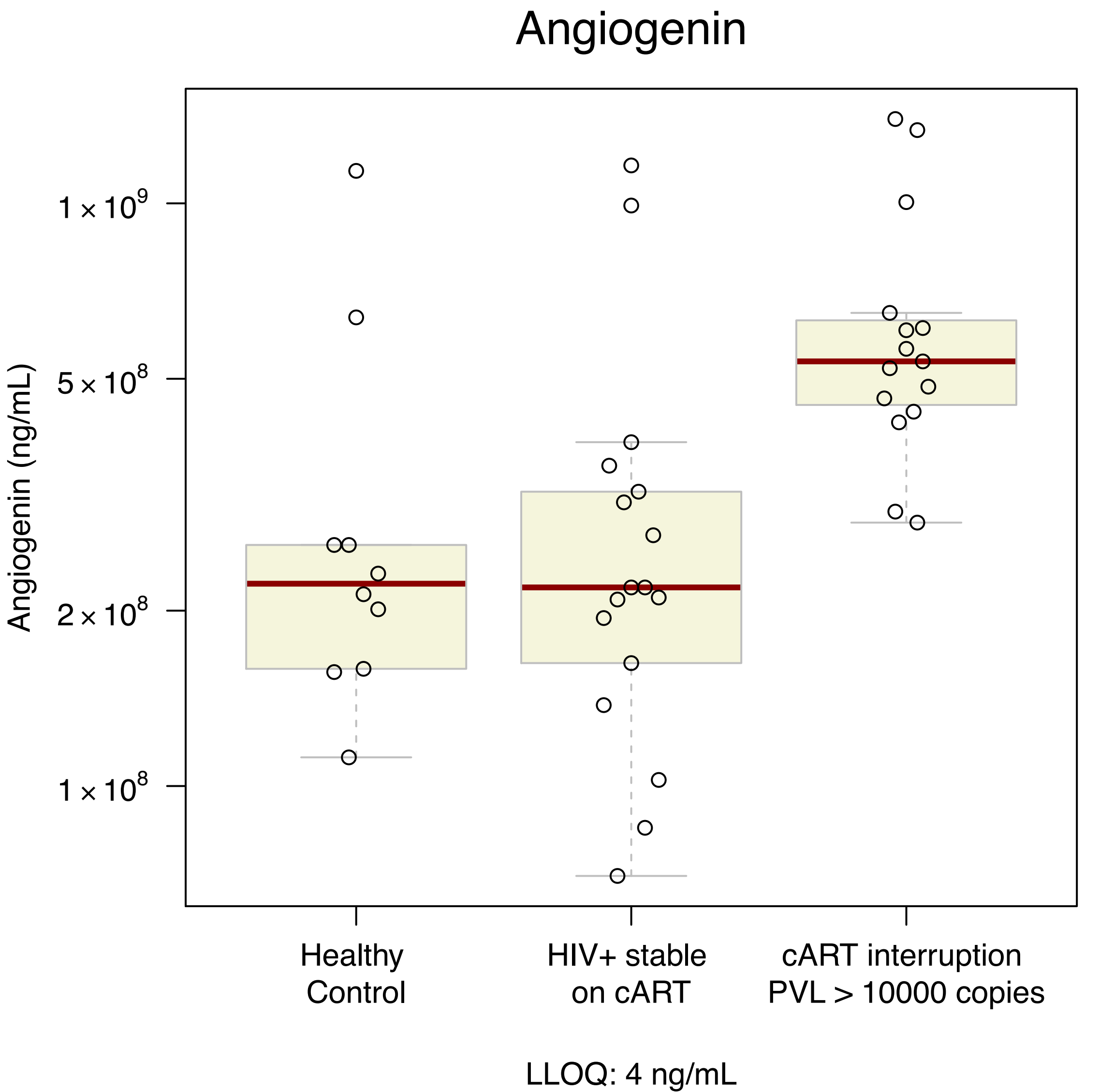
Angiogenin
Angiogenin (ng/mL)
1×10^9
5×10^8
2×10^8
1×10^8
Healthy Control
HIV+ stable on cART
cART interruption PVL > 10000 copies
LLOQ: 4 ng/mL

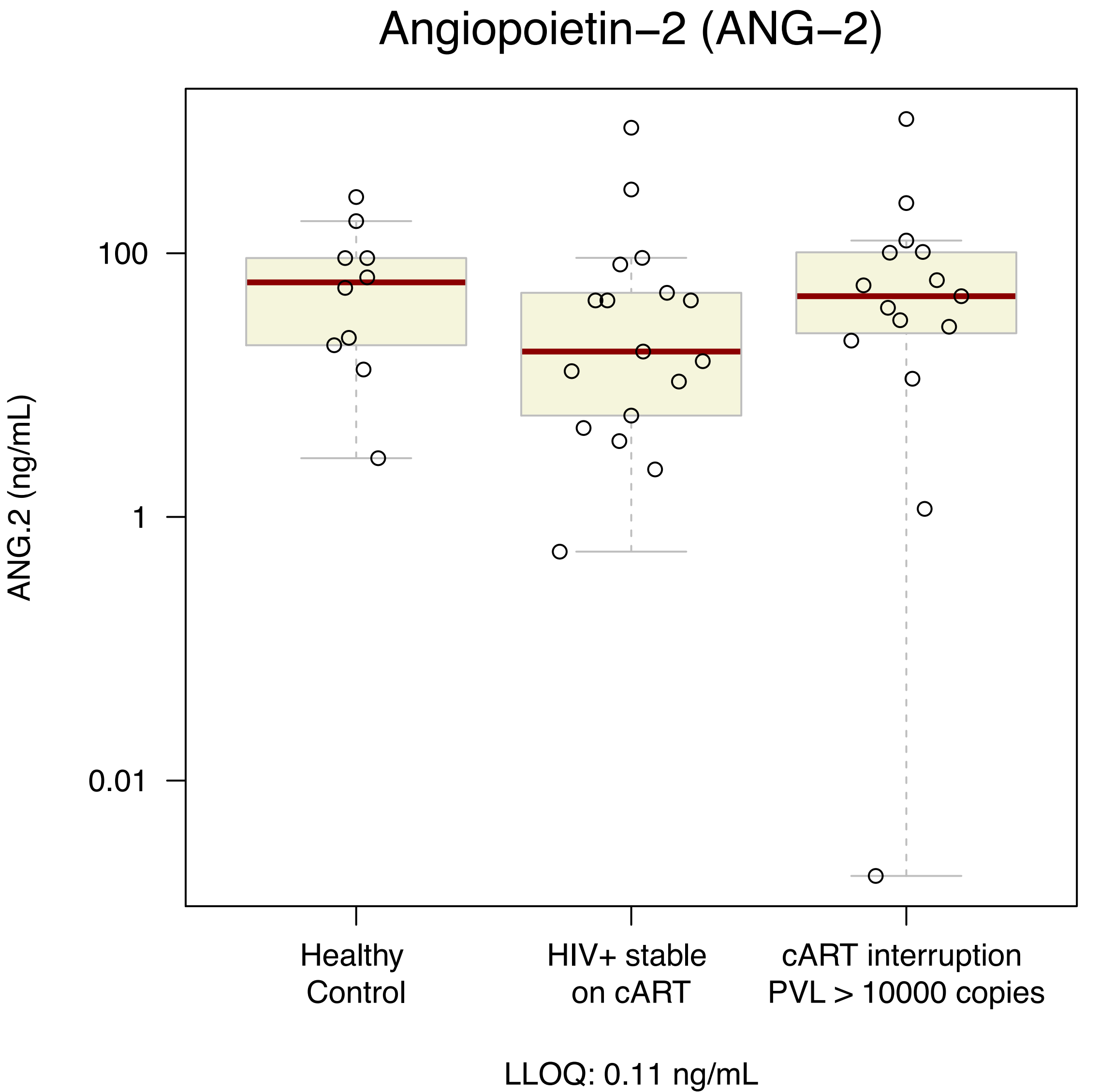
Angiopoietin−2 (ANG−2)
ANG.2 (ng/mL)
100
1
0.01
Healthy Control
HIV+ stable on cART
cART interruption PVL > 10000 copies
LLOQ: 0.11 ng/mL

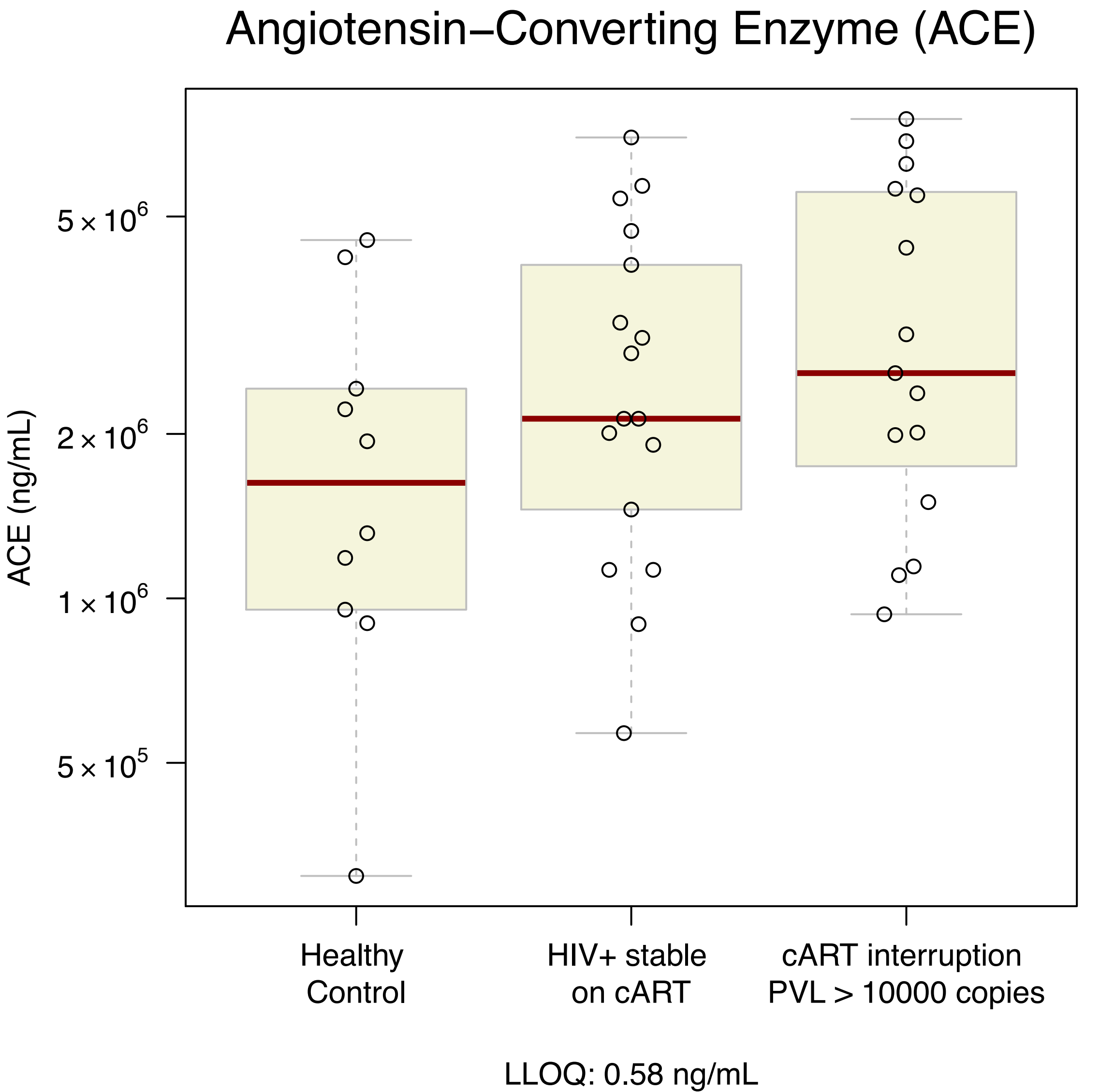
Angiotensin−Converting Enzyme (ACE)
ACE (ng/mL)
5 × 10^6
2 × 10^6
1 × 10^6
5 × 10^5
Healthy Control
HIV+ stable on cART
cART interruption PVL > 10000 copies
LLOQ: 0.58 ng/mL

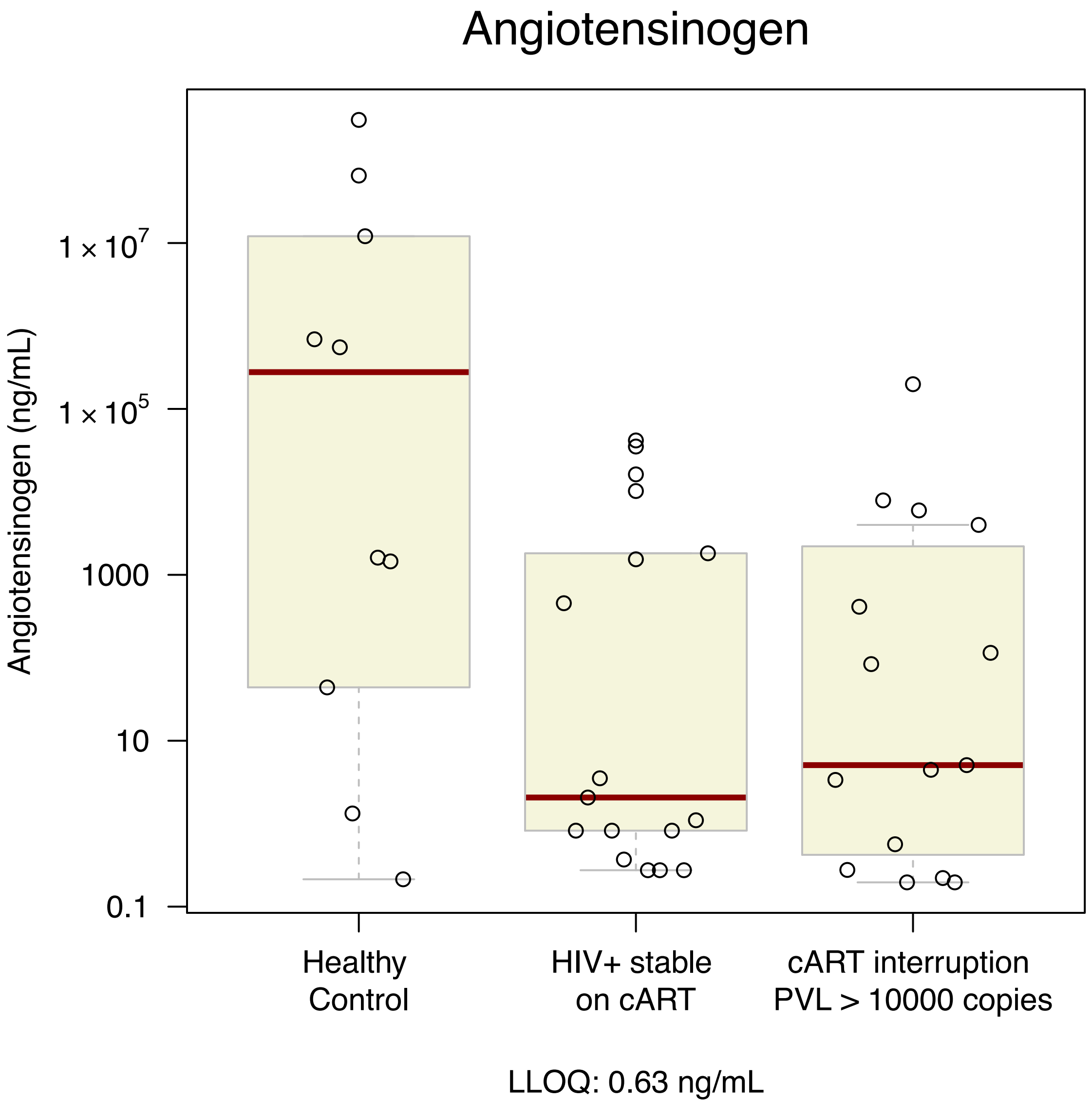

Angiotensinogen
Angiotensinogen (ng/mL)
1 × 10^7
1 × 10^5
1000
10
0.1
Healthy Control
HIV+ stable on cART
cART interruption PVL > 10000 copies
LLOQ: 0.63 ng/mL

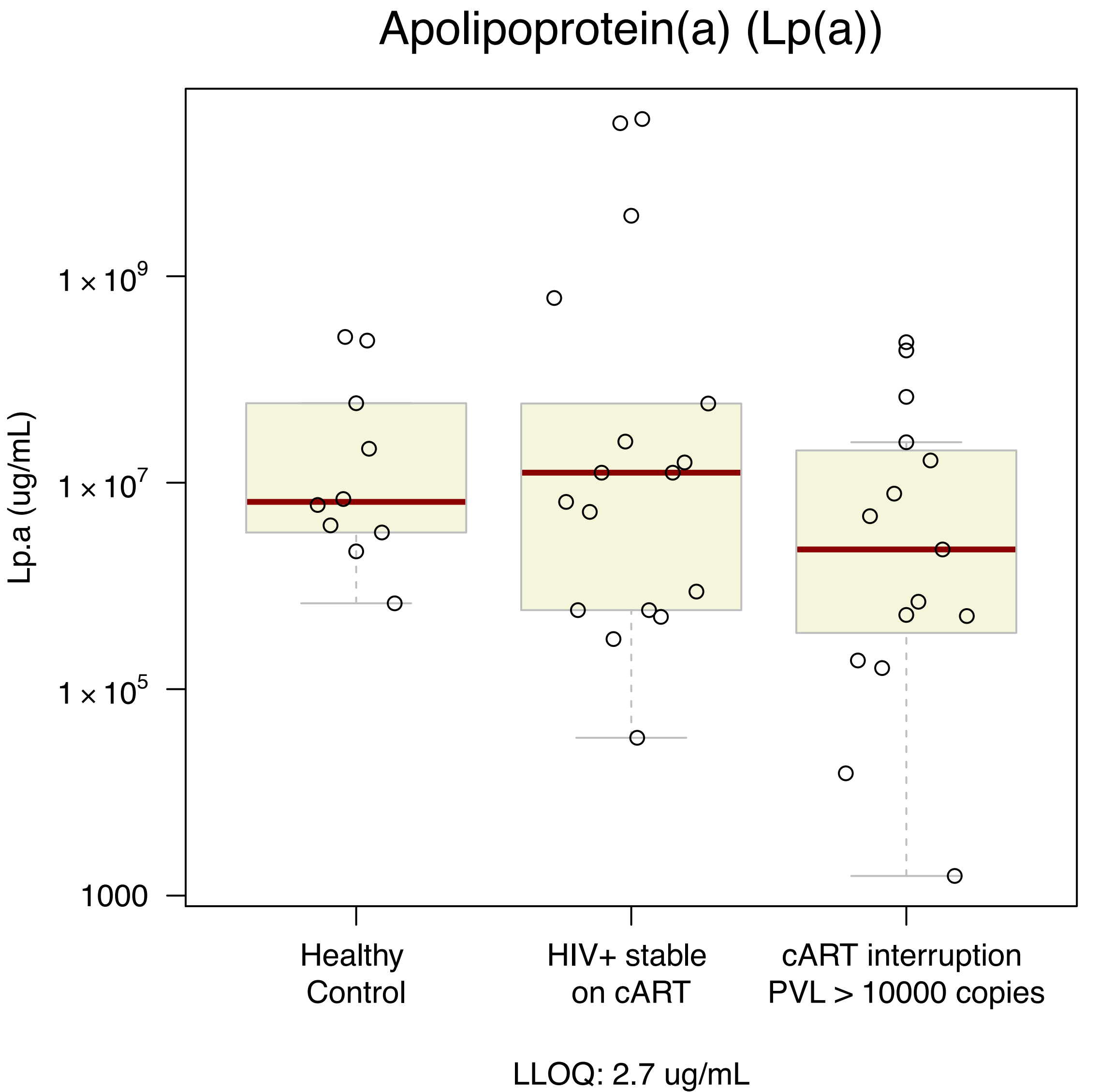

Apolipoprotein(a) (Lp(a))
Lp.a (ug/mL)
1×10^9
1×10^7
1×10^5
1000
Healthy Control
HIV+ stable on cART
cART interruption PVL > 10000 copies
LLOQ: 2.7 ug/mL

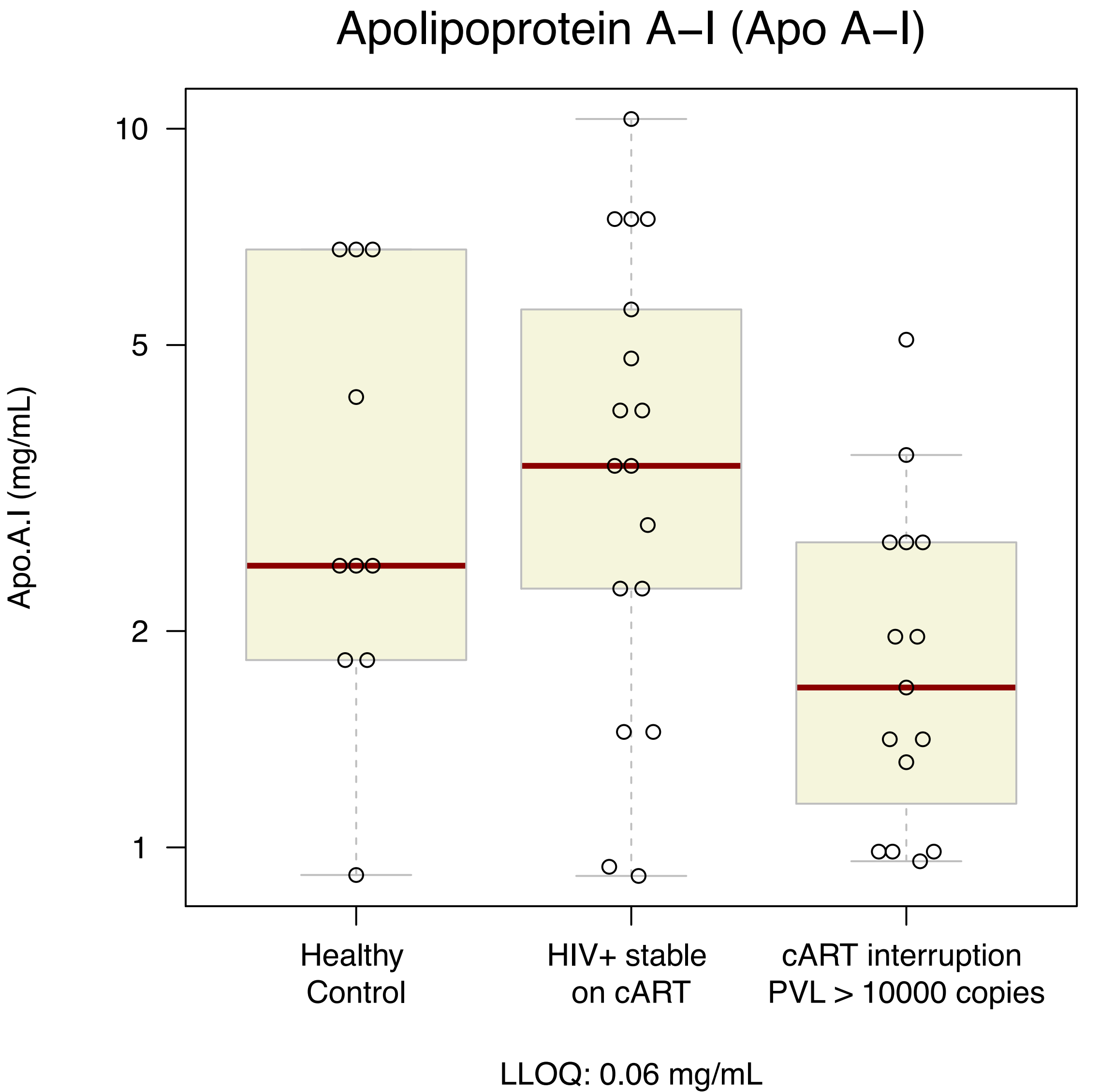

Apolipoprotein A−I (Apo A−I)
10
5
2
1
Apo.A.I (mg/mL)
Healthy Control
HIV+ stable on cART
cART interruption PVL > 10000 copies
LLOQ: 0.06 mg/mL

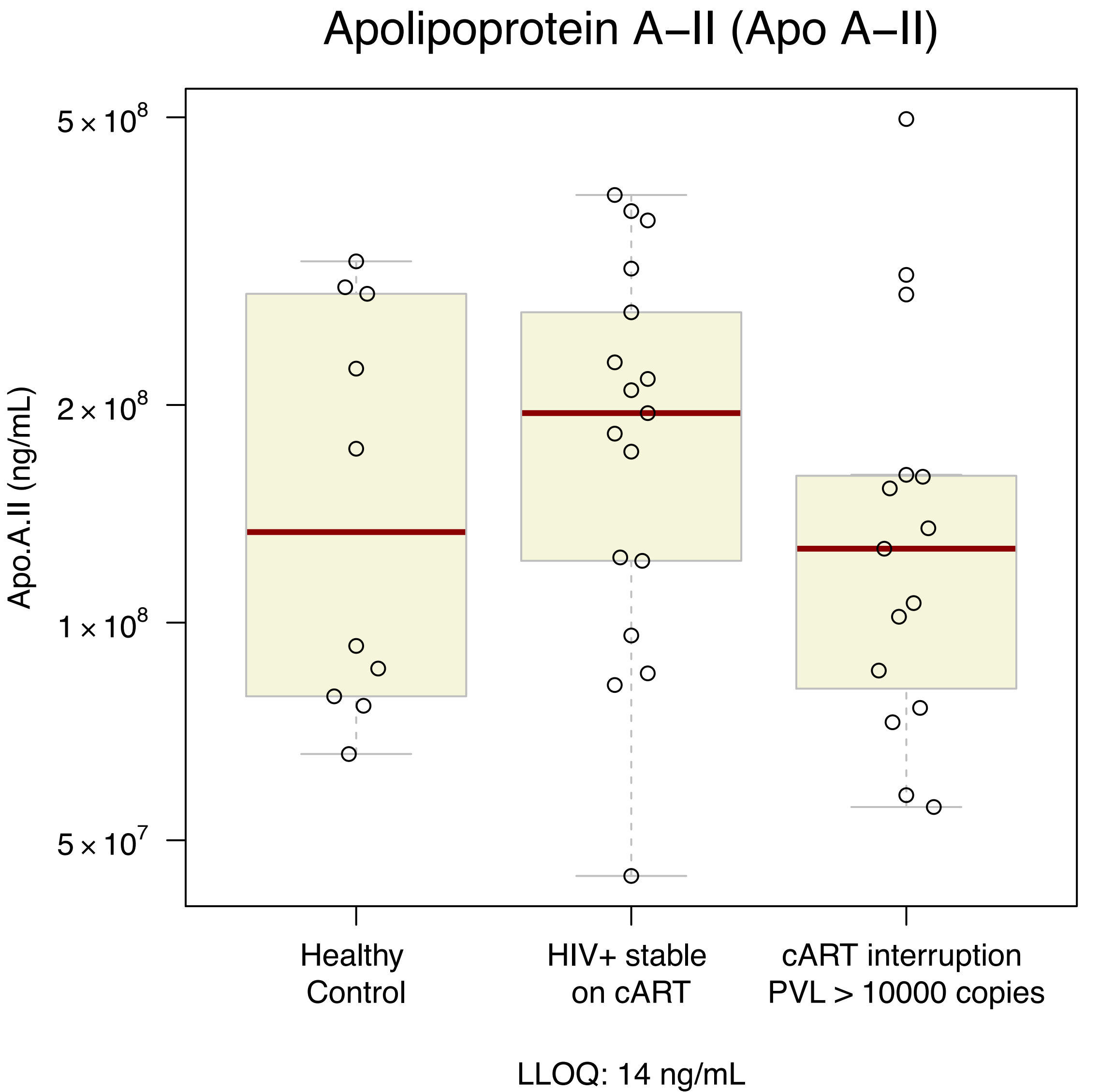

Apolipoprotein A−II (Apo A−II)
Apo.A.II (ng/mL)
5 × 10^8
2 × 10^8
1 × 10^8
5 × 10^7
Healthy Control
HIV+ stable on cART
cART interruption PVL > 10000 copies
LLOQ: 14 ng/mL

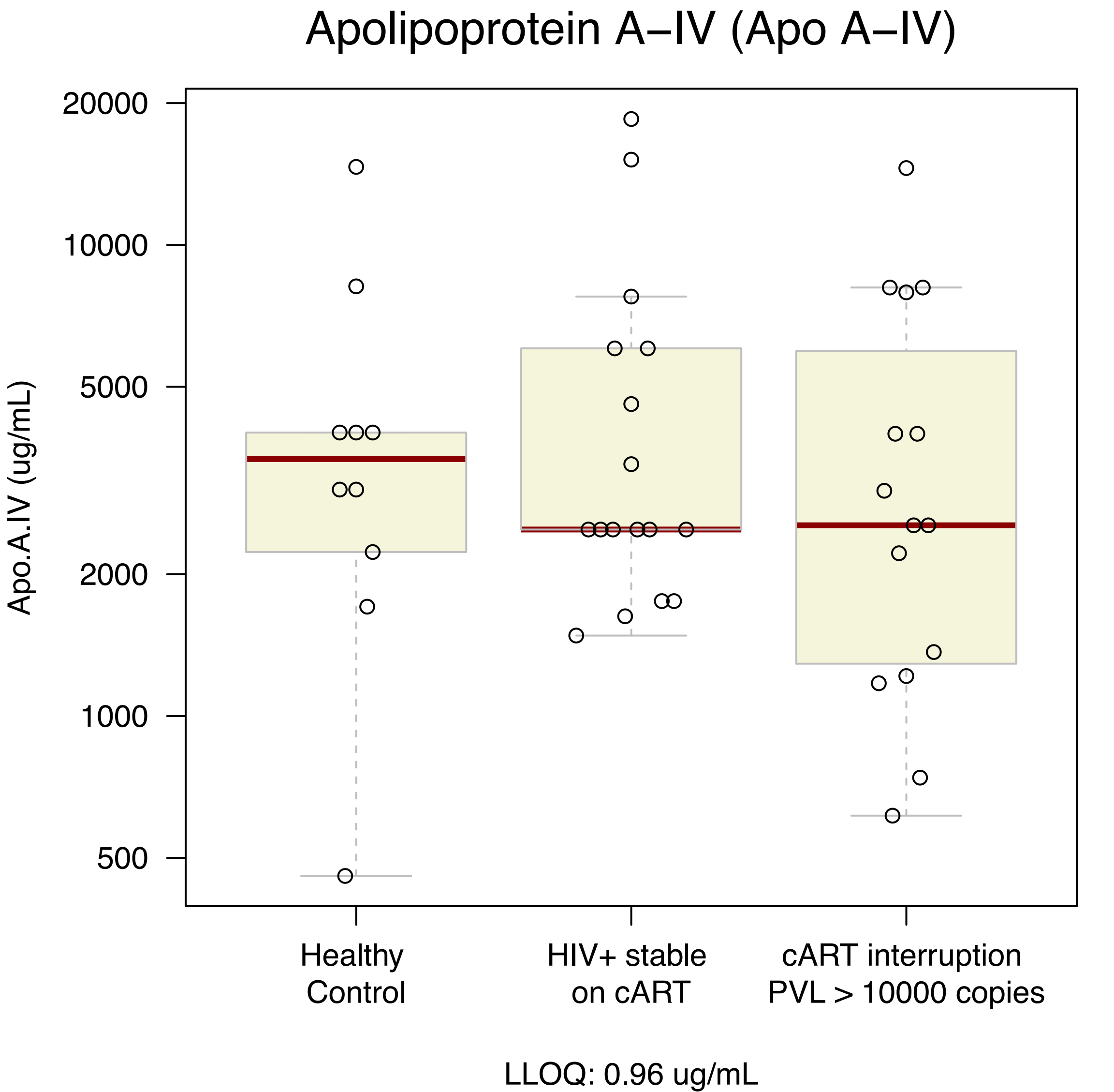

Apolipoprotein A−IV (Apo A−IV)
Apo.A.IV (ug/mL)
20000
10000
5000
2000
1000
500
Healthy Control
HIV+ stable on cART
cART interruption PVL > 10000 copies
LLOQ: 0.96 ug/mL

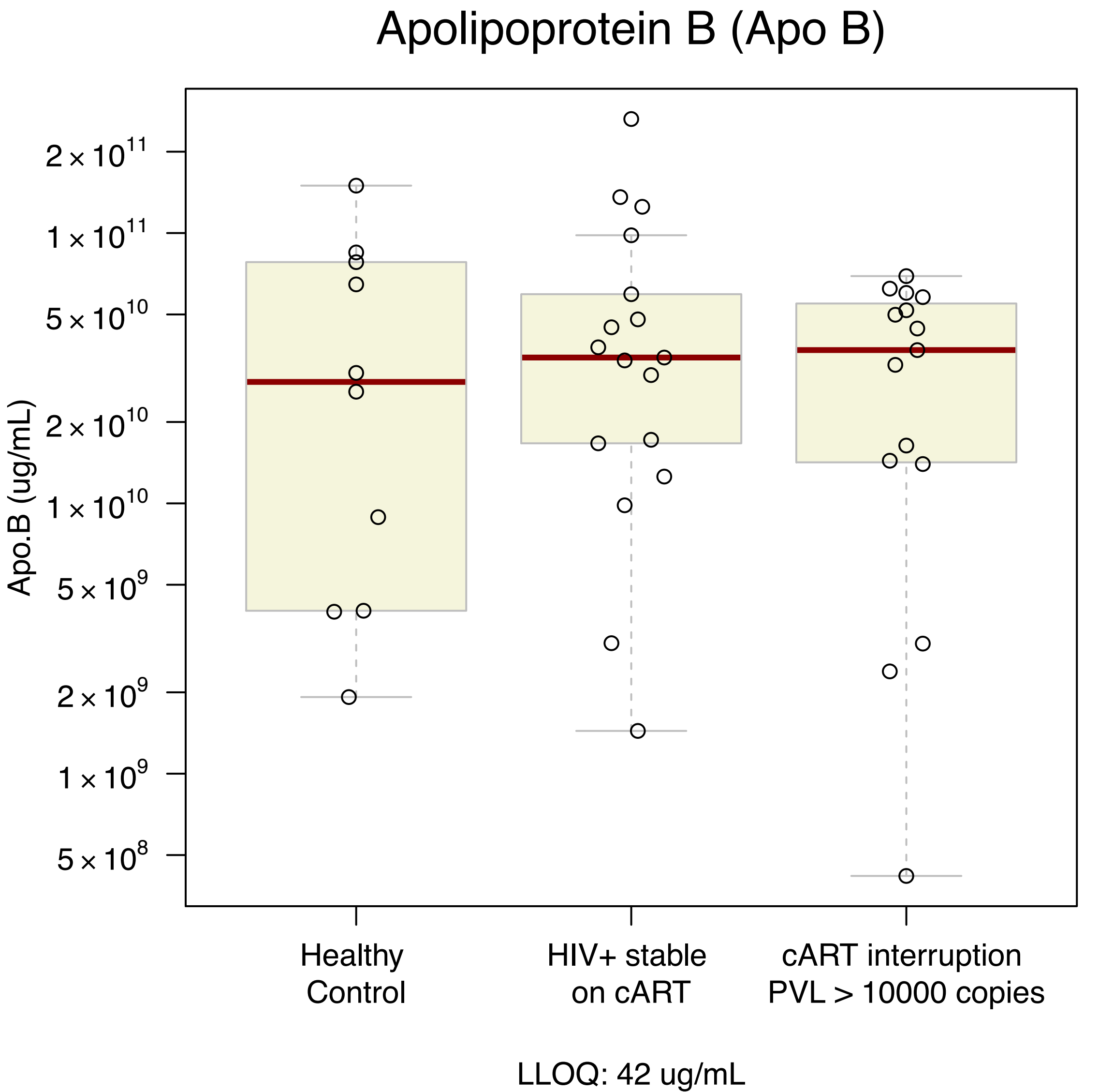
Apolipoprotein B (Apo B)
Apo.B (ug/mL)
2 × 10^11
1 × 10^11
5 × 10^10
2 × 10^10
1 × 10^10
5 × 10^9
2 × 10^9
1 × 10^9
5 × 10^8
Healthy Control
HIV+ stable on cART
cART interruption PVL > 10000 copies
LLOQ: 42 ug/mL

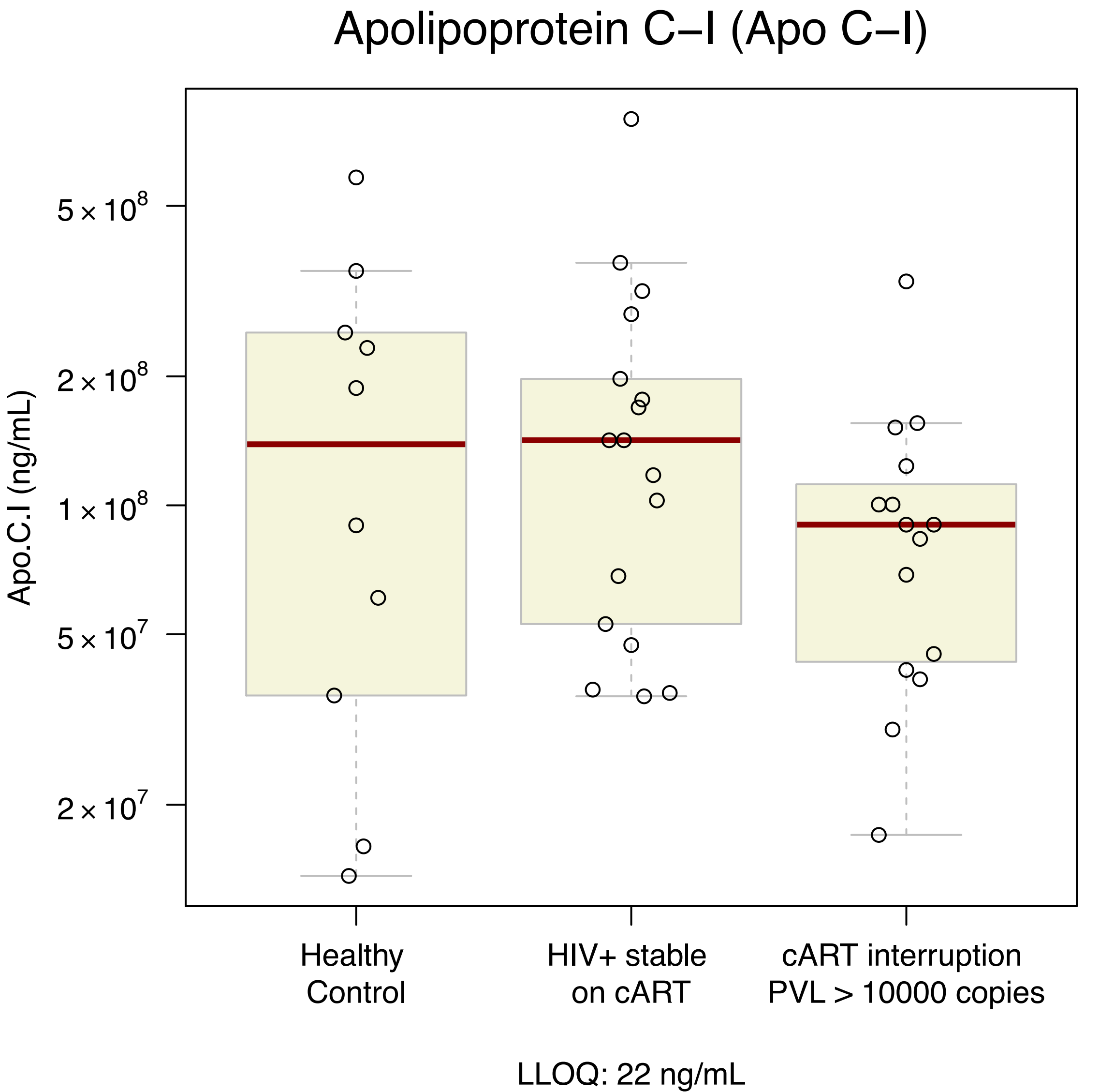

Apolipoprotein C−I (Apo C−I)
Apo.C.I (ng/mL)
5 × 10^8
2 × 10^8
1 × 10^8
5 × 10^7
2 × 10^7
Healthy Control
HIV+ stable on cART
cART interruption PVL > 10000 copies
LLOQ: 22 ng/mL

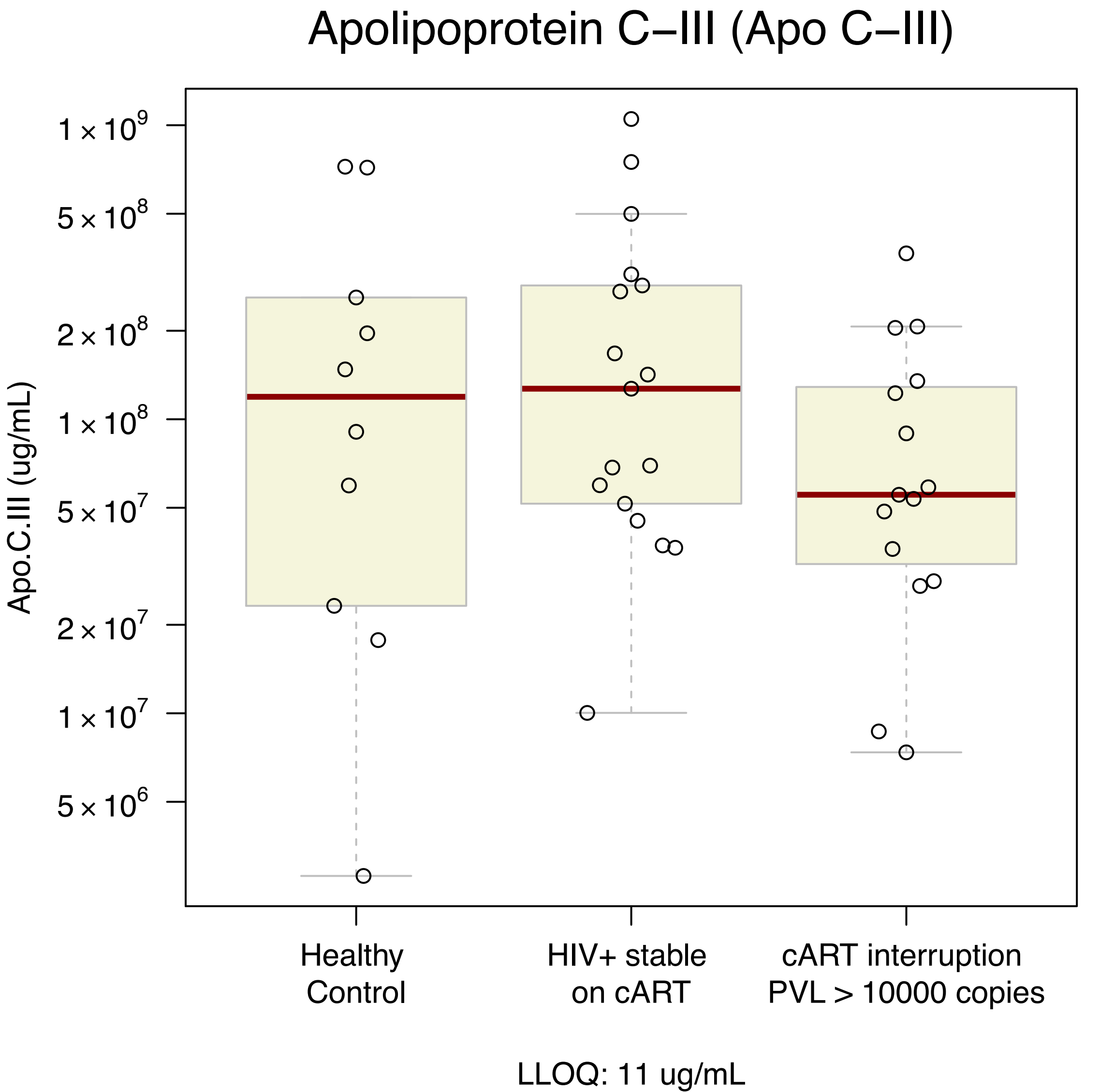

Apolipoprotein C−III (Apo C−III)
Apo.C.III (ug/mL)
1×10^9
5×10^8
2×10^8
1×10^8
5×10^7
2×10^7
1×10^7
5×10^6
Healthy Control
HIV+ stable on cART
cART interruption PVL > 10000 copies
LLOQ: 11 ug/mL

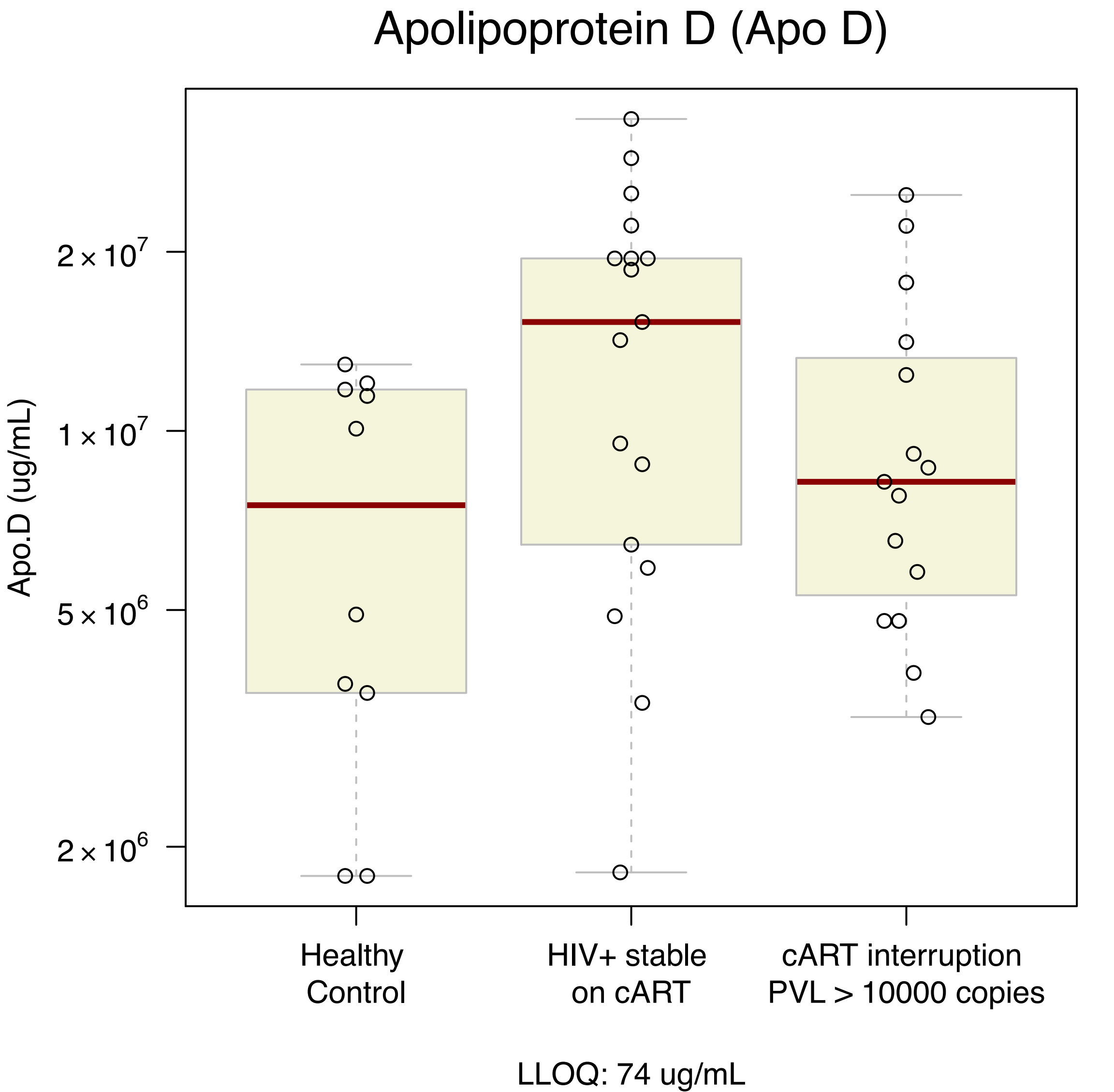
Apolipoprotein D (Apo D)
Apo.D (ug/mL)
2 × 10^7
1 × 10^7
5 × 10^6
2 × 10^6
Healthy Control
HIV+ stable on cART
cART interruption PVL > 10000 copies
LLOQ: 74 ug/mL

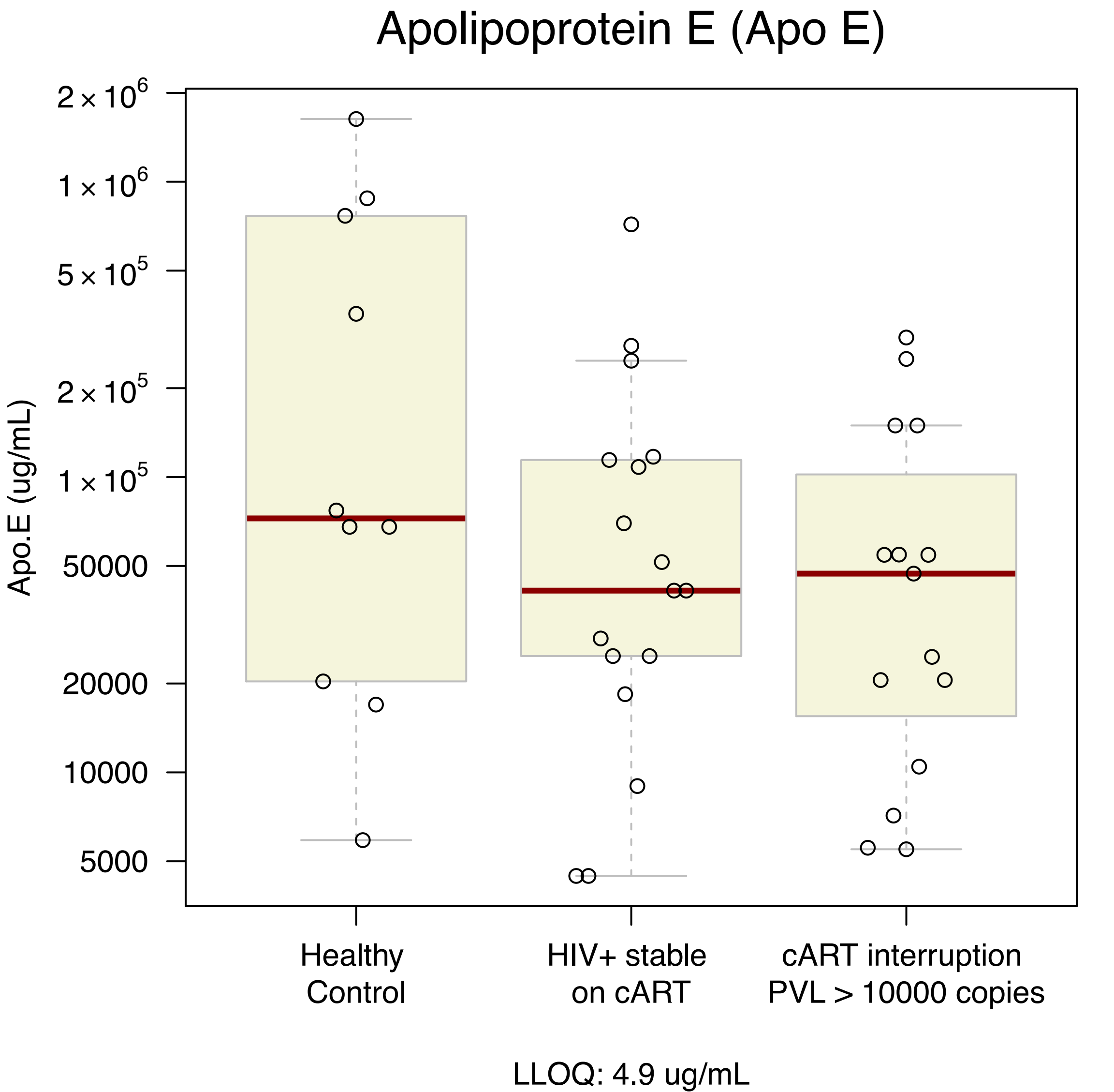
Apolipoprotein E (Apo E)
Apo.E (ug/mL)
2×10^6
1×10^6
5×10^5
2×10^5
1×10^5
50000
20000
10000
5000
Healthy Control
HIV+ stable on cART
cART interruption PVL > 10000 copies
LLOQ: 4.9 ug/mL

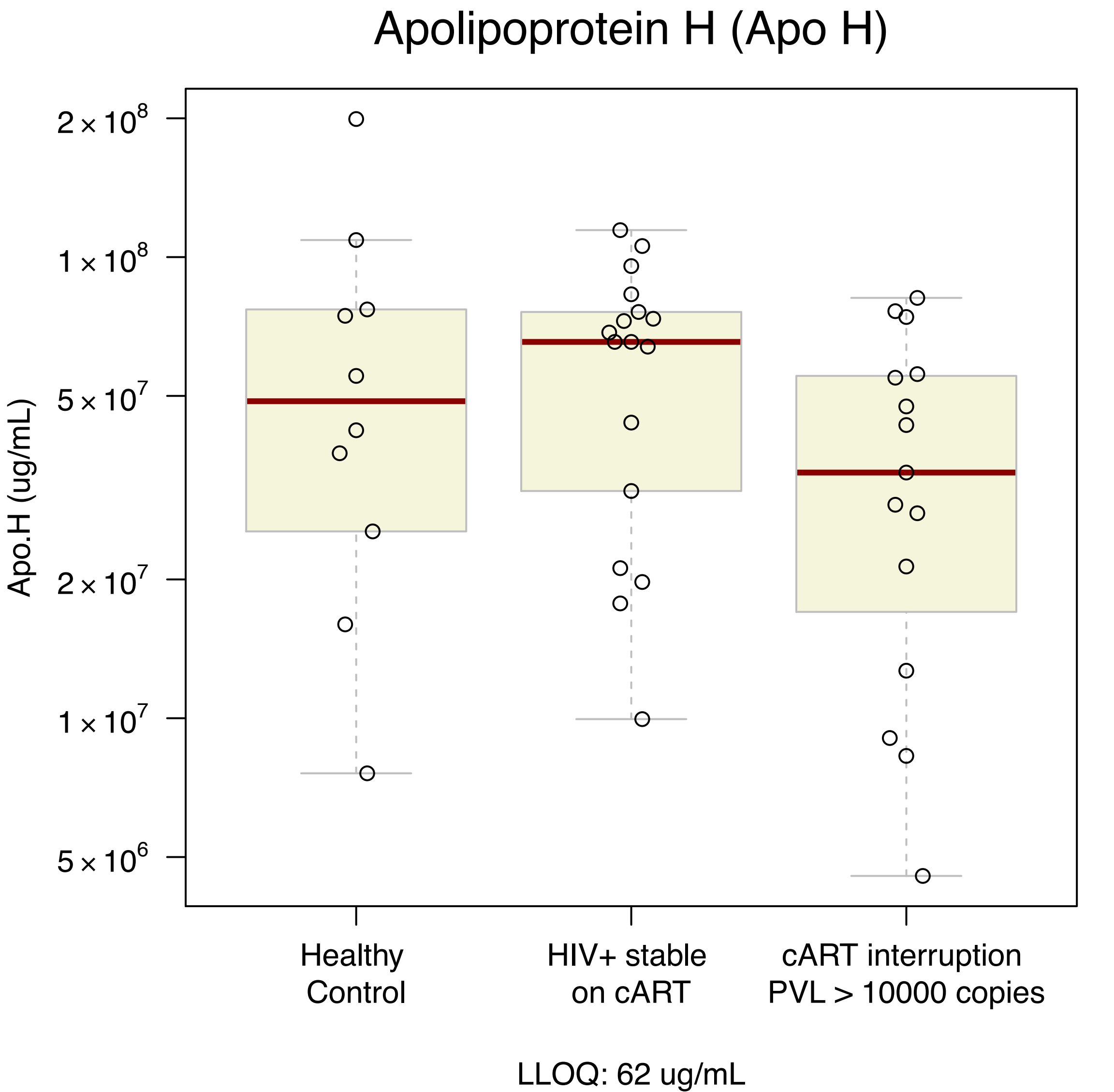

Apolipoprotein H (Apo H)
Apo.H (ug/mL)
2 × 10^8
1 × 10^8
5 × 10^7
2 × 10^7
1 × 10^7
5 × 10^6
Healthy Control
HIV+ stable on cART
cART interruption PVL > 10000 copies
LLOQ: 62 ug/mL

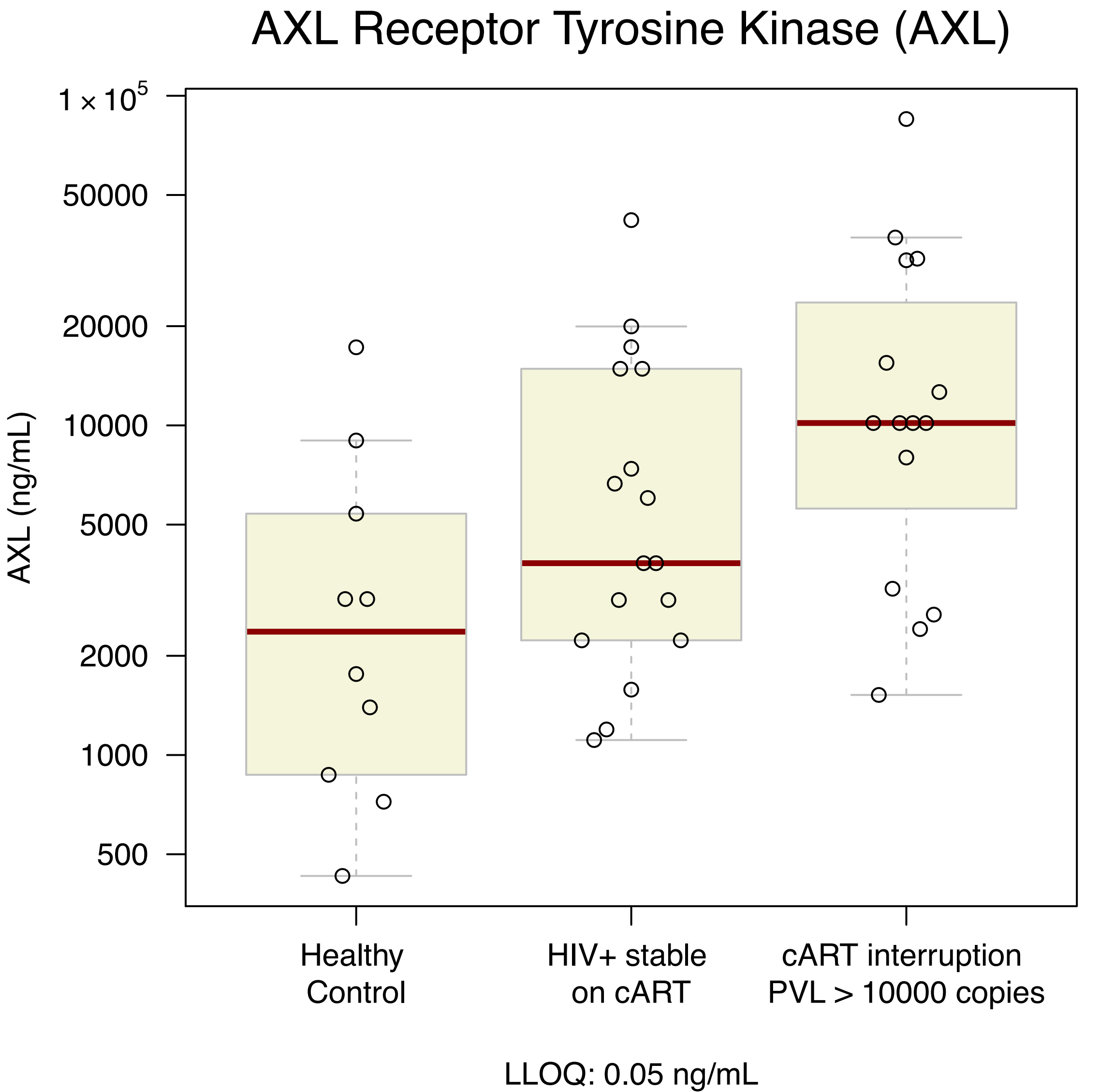

AXL Receptor Tyrosine Kinase (AXL)
AXL (ng/mL)
1×10^5
50000
20000
10000
5000
2000
1000
500
Healthy Control
HIV+ stable on cART
cART interruption PVL > 10000 copies
LLOQ: 0.05 ng/mL

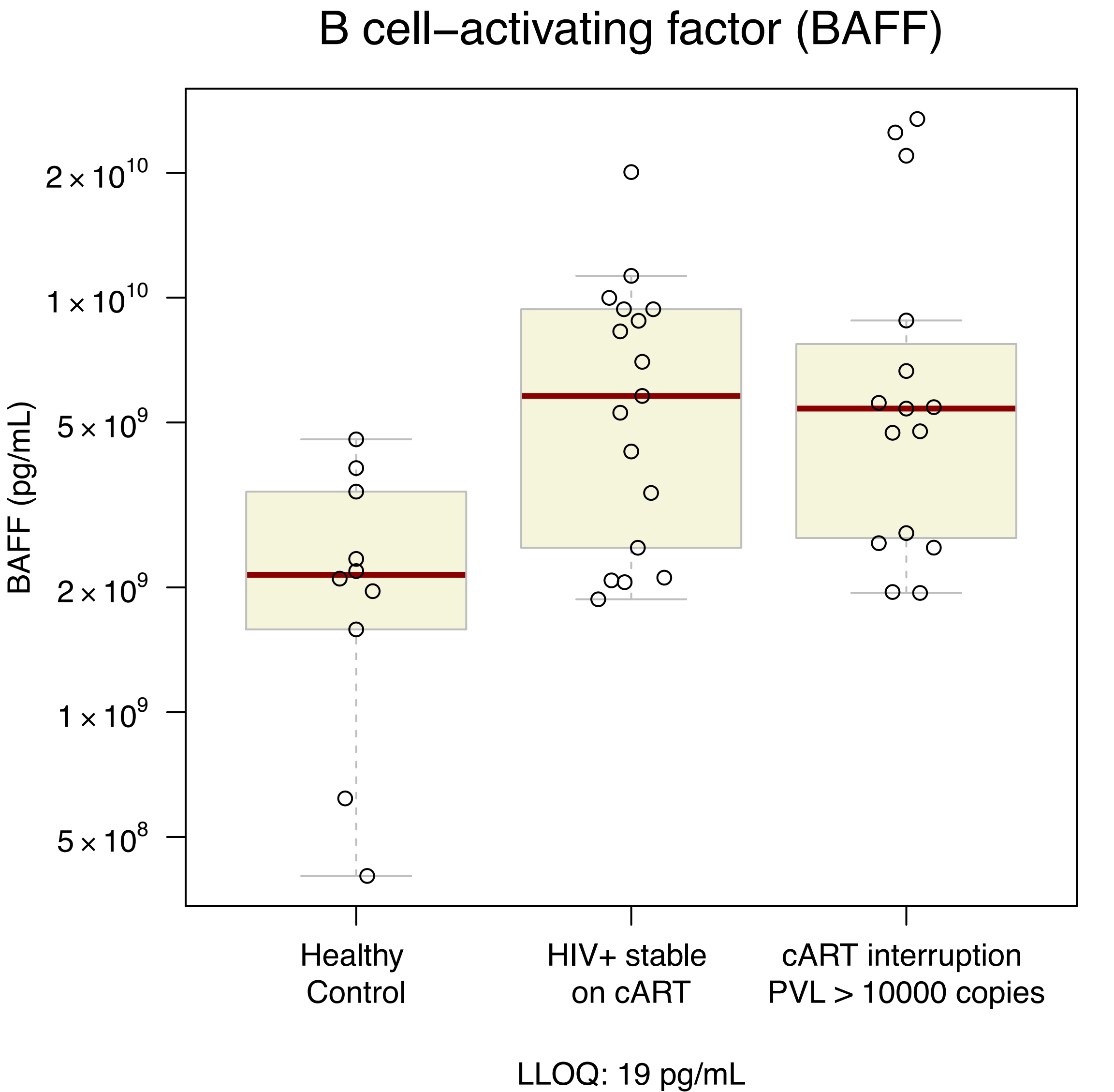
B cell–activating factor (BAFF)
BAFF (pg/mL)
2 × 10^10
1 × 10^10
5 × 10^9
2 × 10^9
1 × 10^9
5 × 10^8
Healthy Control
HIV+ stable on cART
cART interruption PVL > 10000 copies
LLOQ: 19 pg/mL

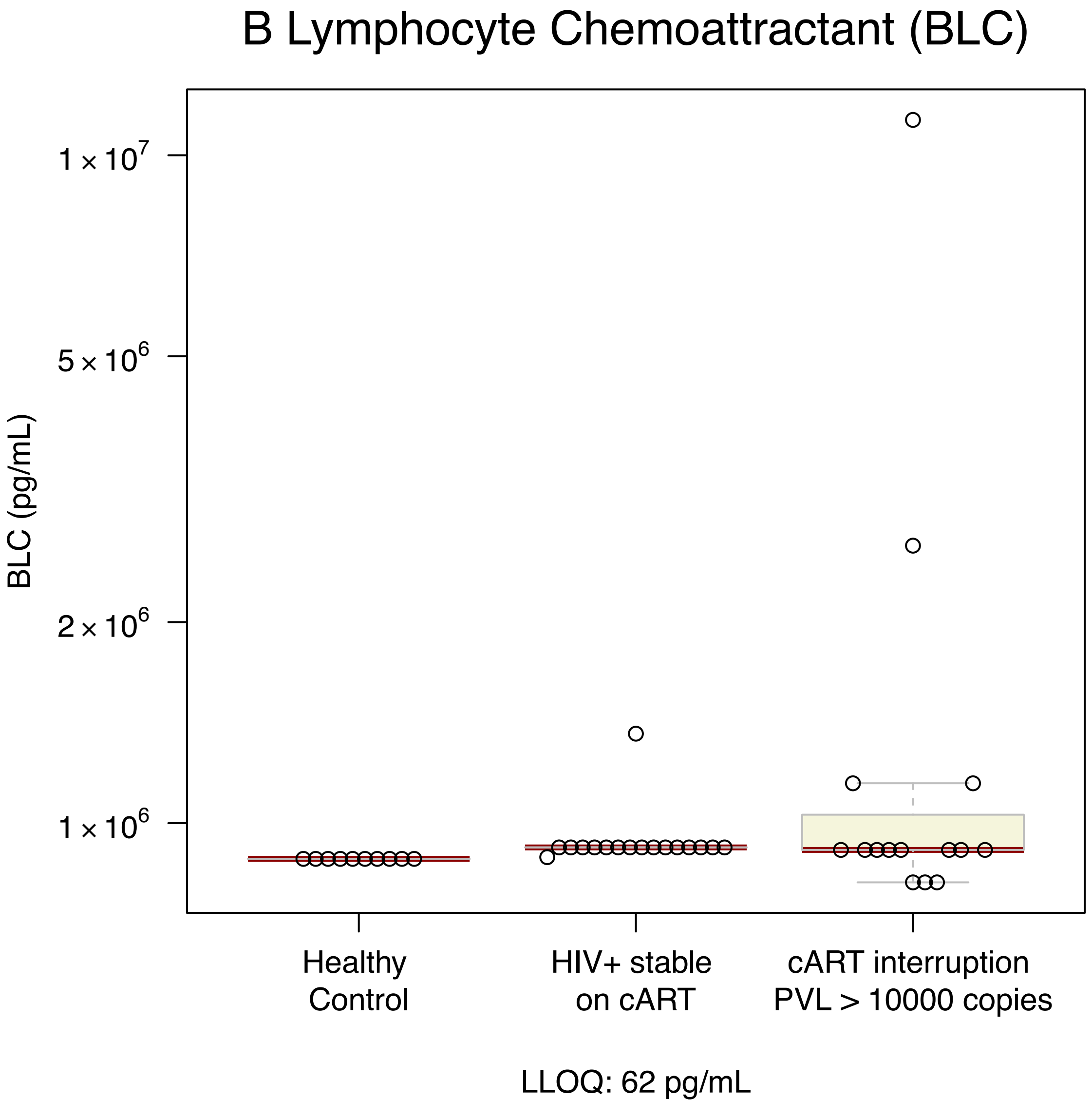

B Lymphocyte Chemoattractant (BLC)
BLC (pg/mL)
1×10^7
5×10^6
2×10^6
1×10^6
Healthy Control
HIV+ stable on cART
cART interruption PVL > 10000 copies
LLOQ: 62 pg/mL

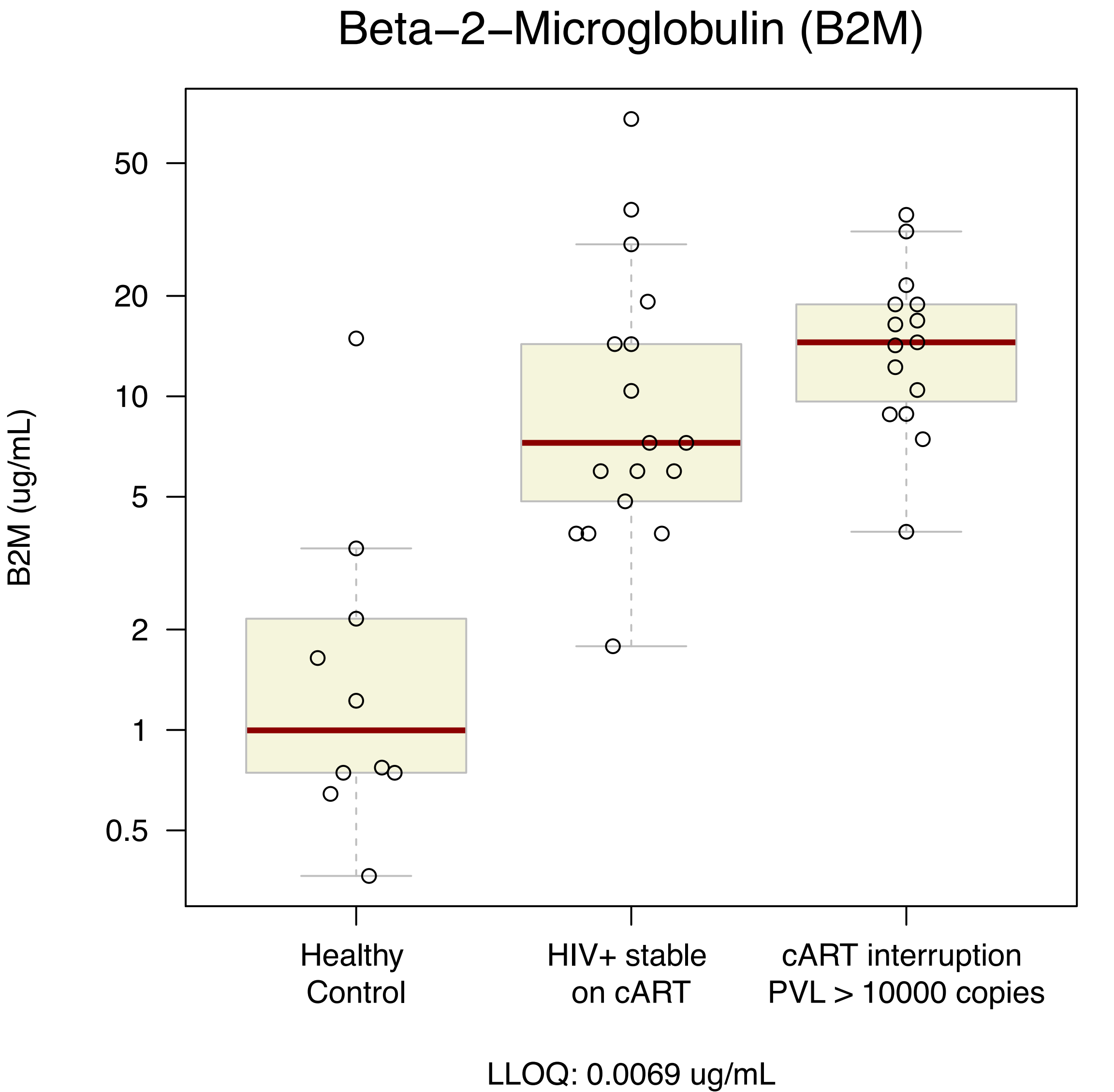
Beta−2−Microglobulin (B2M)
B2M (ug/mL)
50
20
10
5
2
1
0.5
Healthy
Control
HIV+ stable
on cART
cART interruption
PVL > 10000 copies
LLOQ: 0.0069 ug/mL

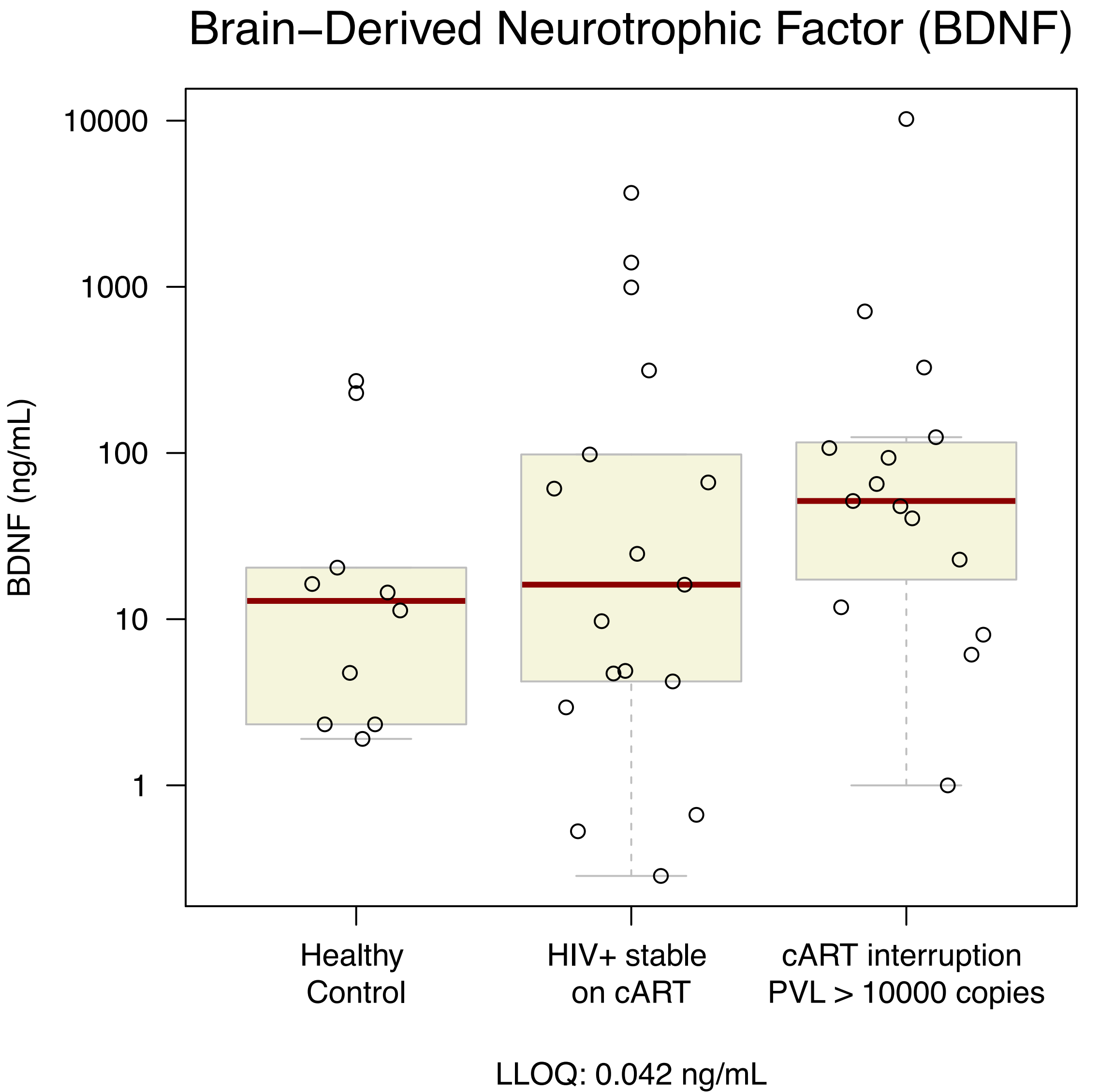
Brain−Derived Neurotrophic Factor (BDNF)
10000
1000
100
10
1
BDNF (ng/mL)
Healthy Control
HIV+ stable on cART
cART interruption PVL > 10000 copies
LLOQ: 0.042 ng/mL

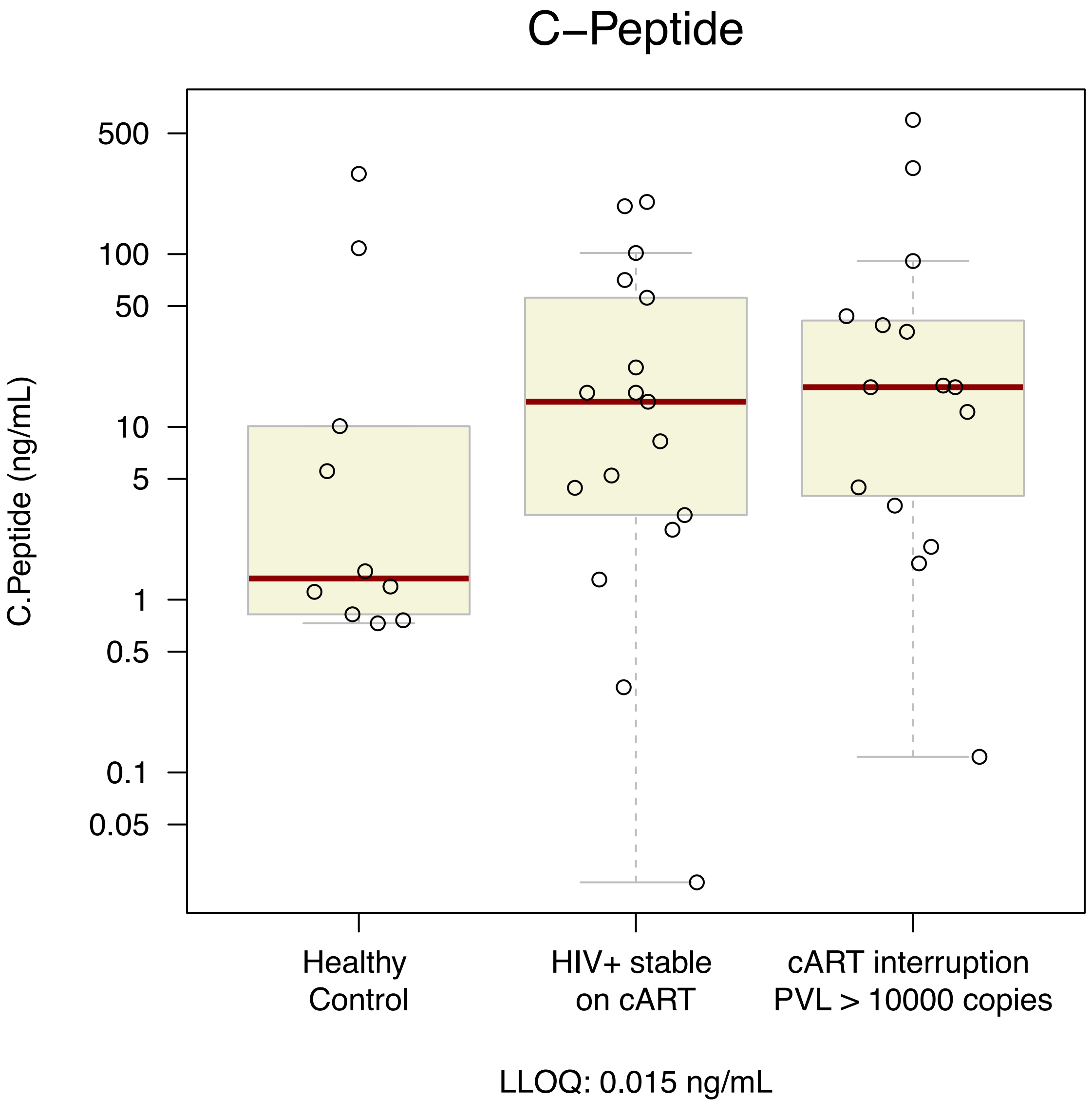
C−Peptide
500
100
50
10
5
1
0.5
0.1
0.05
C.Peptide (ng/mL)
Healthy Control
HIV+ stable on cART
cART interruption PVL > 10000 copies
LLOQ: 0.015 ng/mL

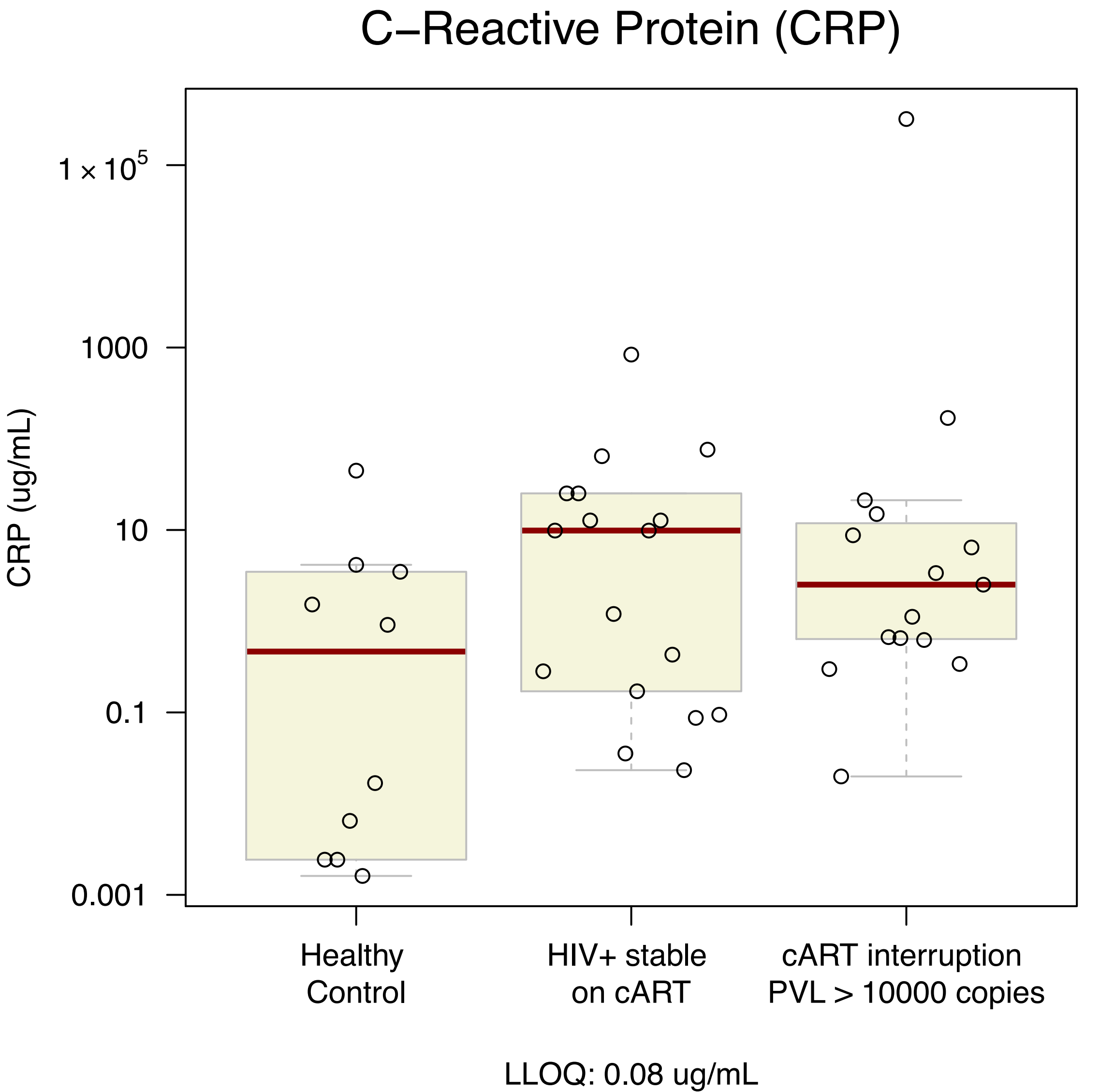
C–Reactive Protein (CRP)
CRP (ug/mL)
1×10^5
1000
10
0.1
0.001
Healthy Control
HIV+ stable on cART
cART interruption PVL > 10000 copies
LLOQ: 0.08 ug/mL

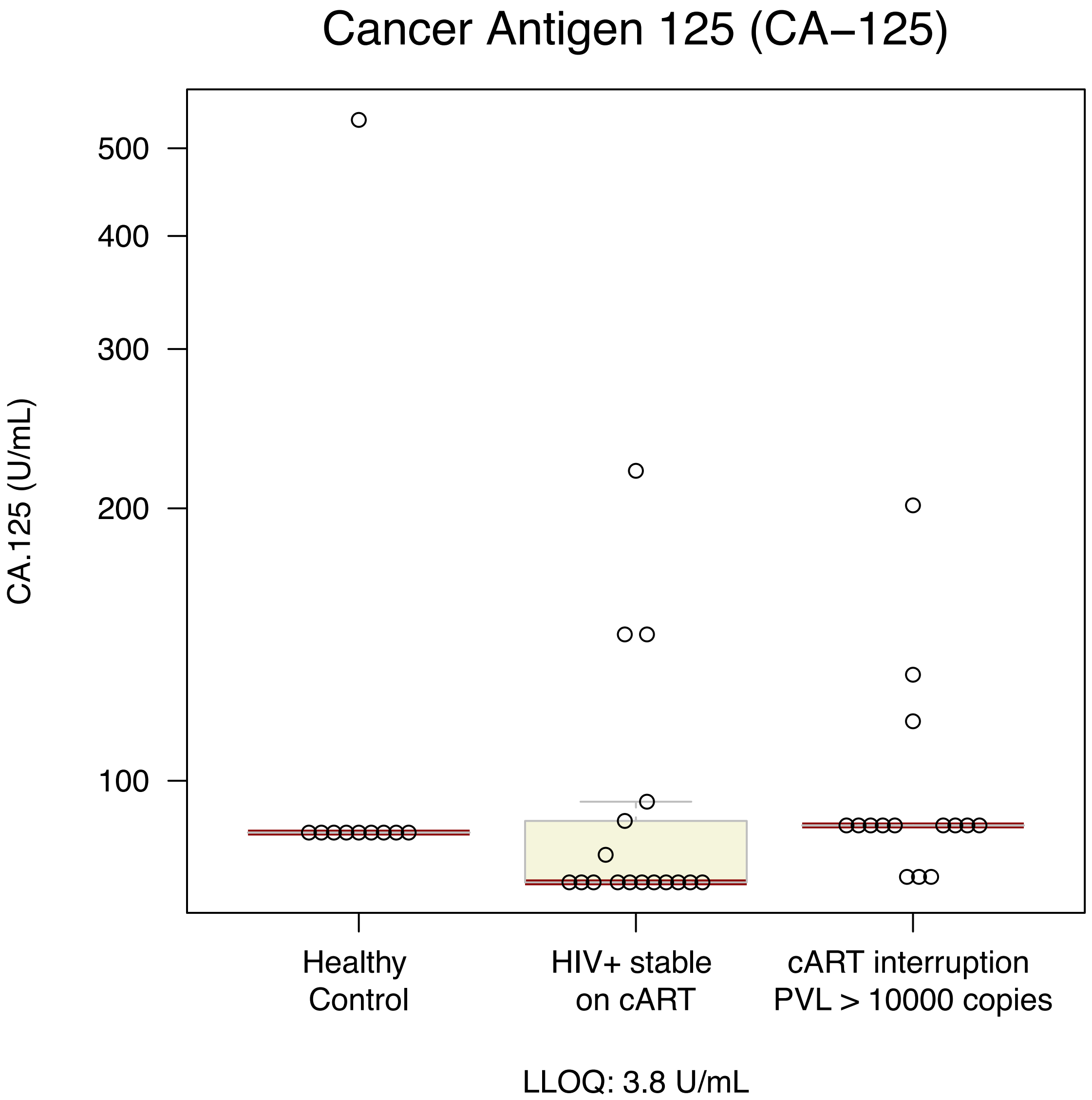

Cancer Antigen 125 (CA−125)
CA.125 (U/mL)
500
400
300
200
100
Healthy Control
HIV+ stable on cART
cART interruption PVL > 10000 copies
LLOQ: 3.8 U/mL

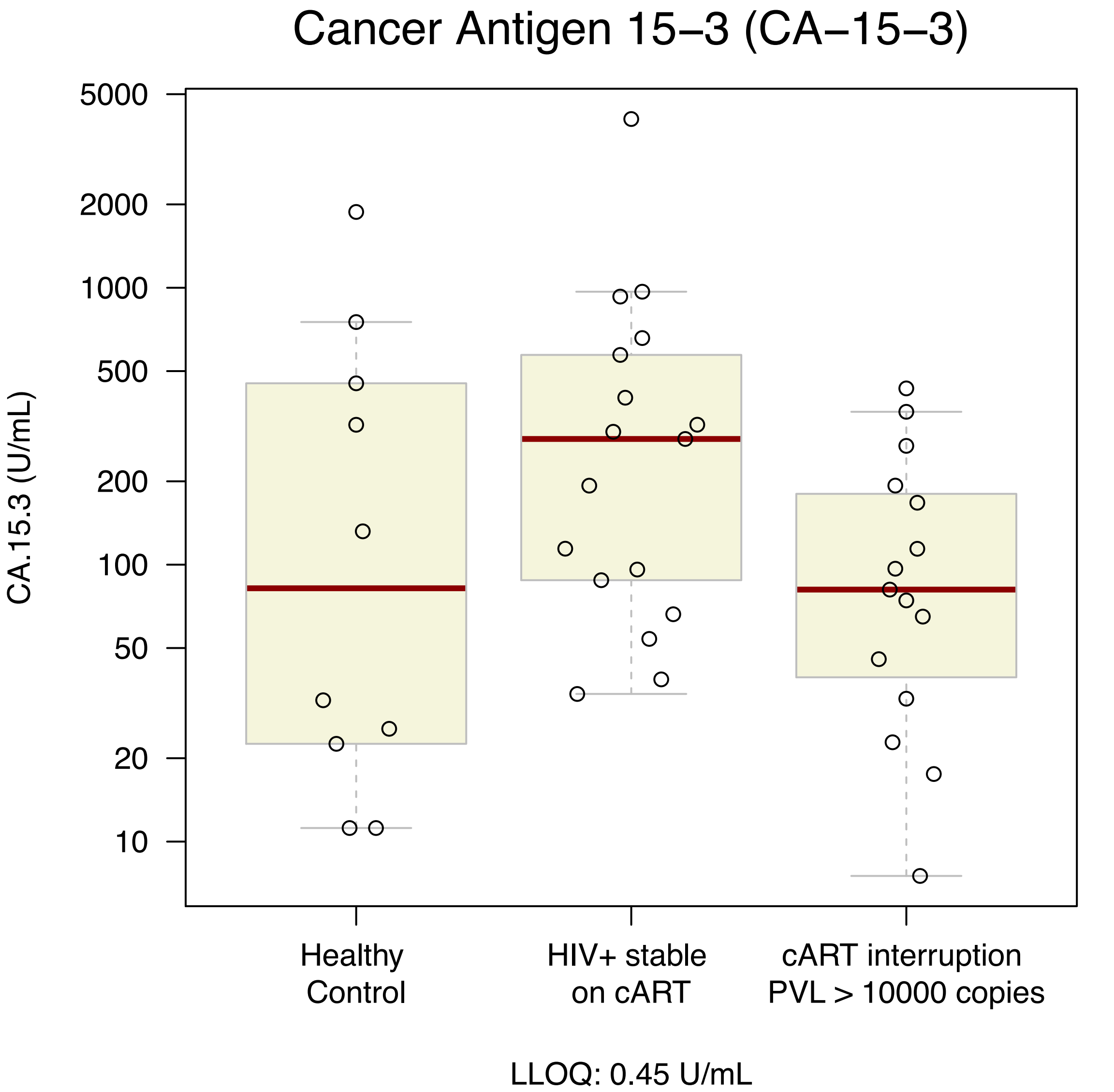
Cancer Antigen 15−3 (CA−15−3)
5000
2000
1000
500
200
100
50
20
10
CA.15.3 (U/mL)
Healthy Control
HIV+ stable on cART
cART interruption PVL > 10000 copies
LLOQ: 0.45 U/mL

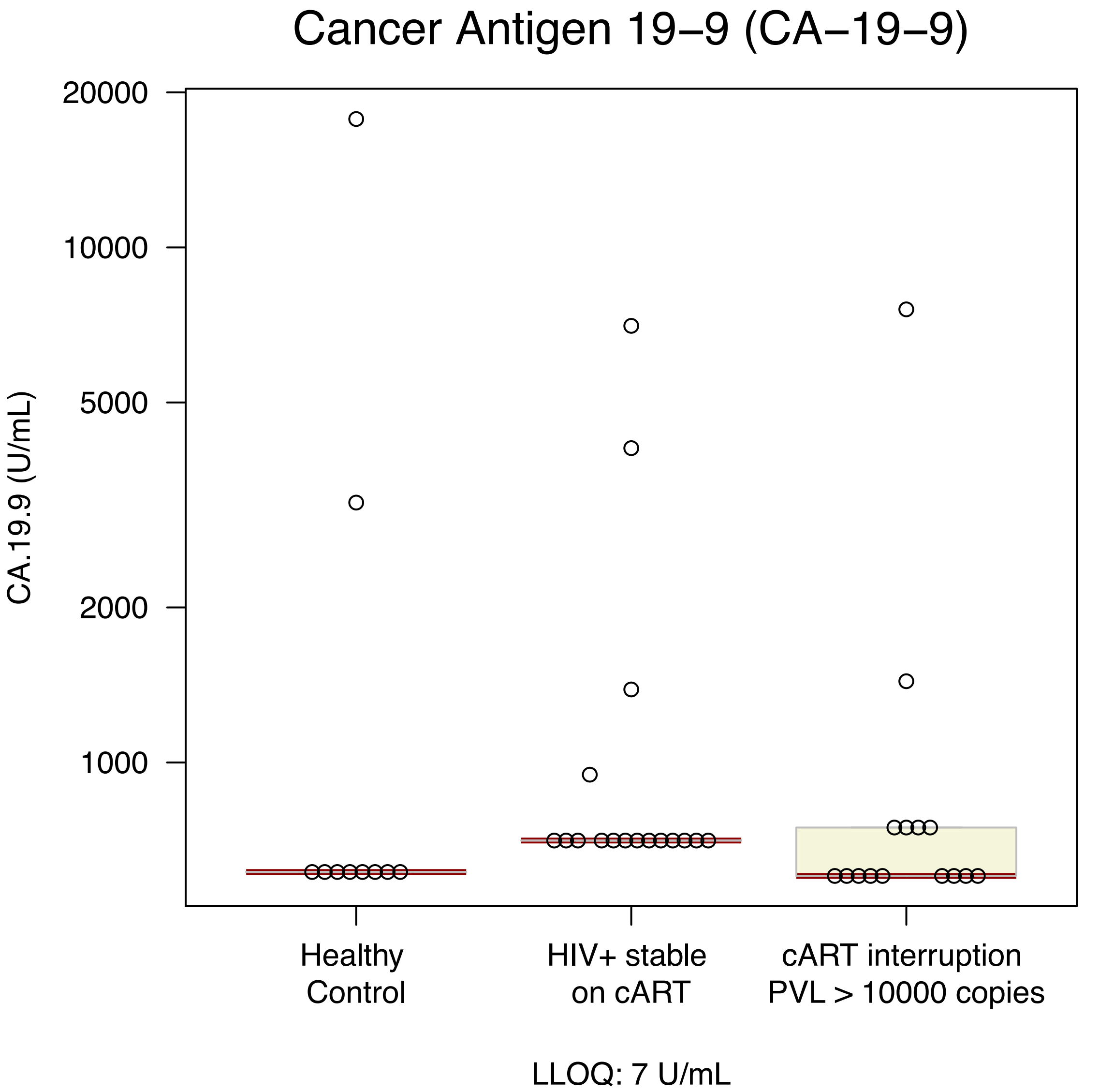
Cancer Antigen 19−9 (CA−19−9)
CA.19.9 (U/mL)
20000
10000
5000
2000
1000
Healthy Control
HIV+ stable on cART
cART interruption PVL > 10000 copies
LLOQ: 7 U/mL

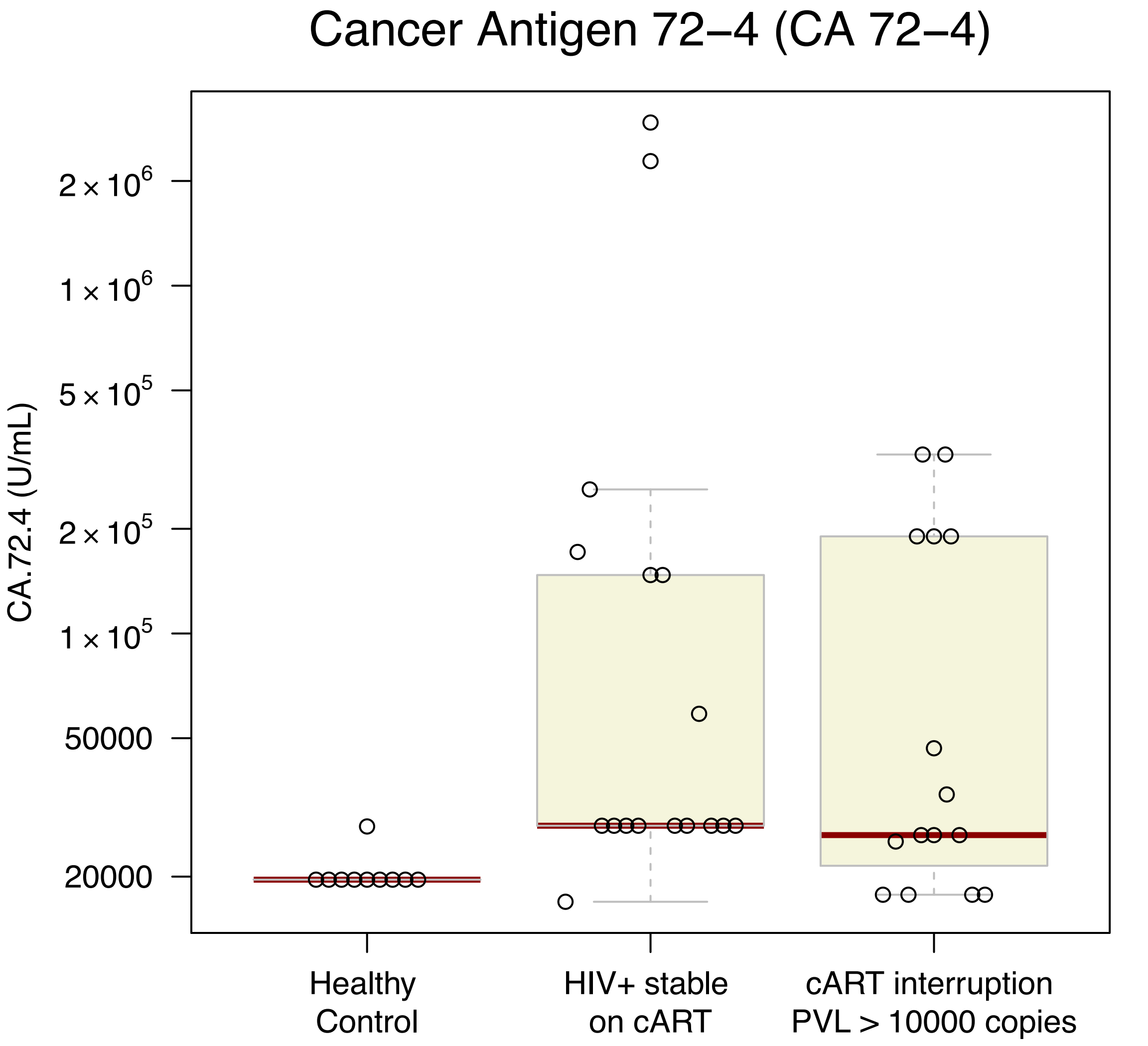
Cancer Antigen 72−4 (CA 72−4)
CA.72.4 (U/mL)
2 × 10^6
1 × 10^6
5 × 10^5
2 × 10^5
1 × 10^5
50000
20000
Healthy Control
HIV+ stable on cART
cART interruption PVL > 10000 copies

LLOQ: 20 U/mL

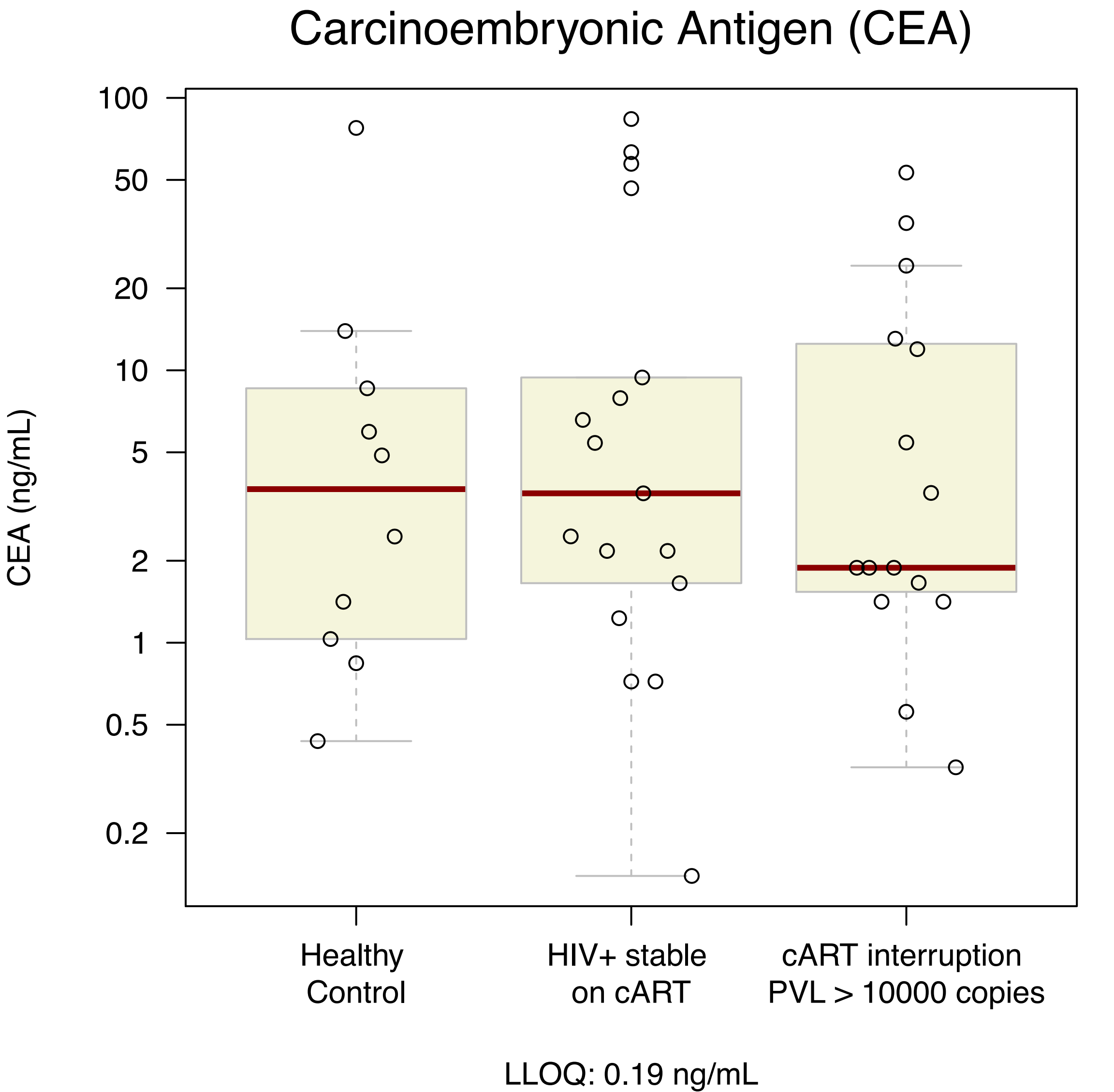

Carcinoembryonic Antigen (CEA)
CEA (ng/mL)
100
50
20
10
5
2
1
0.5
0.2
Healthy Control
HIV+ stable on cART
cART interruption PVL > 10000 copies
LLOQ: 0.19 ng/mL

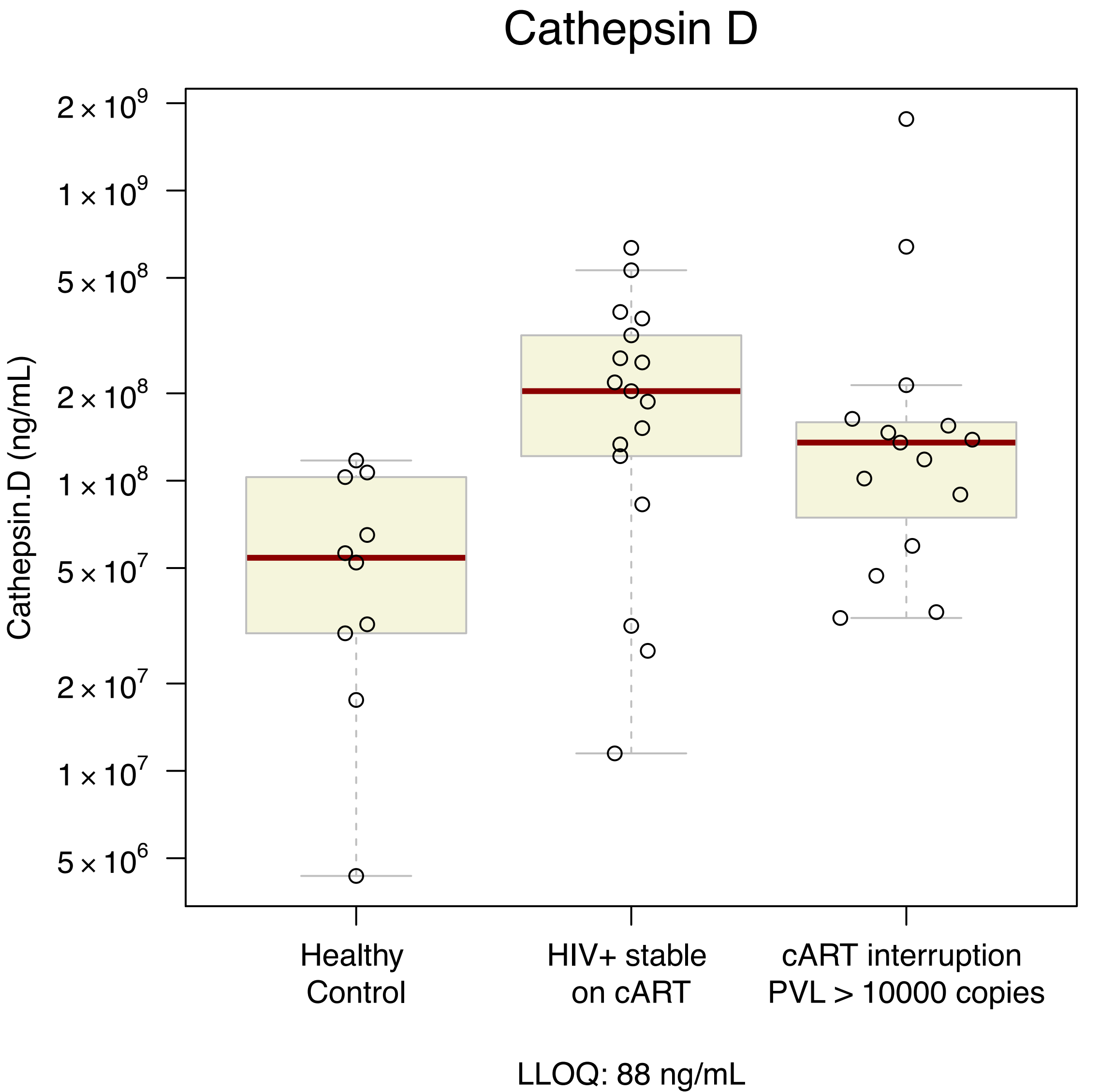

Cathepsin D
Cathepsin.D (ng/mL)
2×10^9
1×10^9
5×10^8
2×10^8
1×10^8
5×10^7
2×10^7
1×10^7
5×10^6
Healthy Control
HIV+ stable on cART
cART interruption PVL > 10000 copies
LLOQ: 88 ng/mL

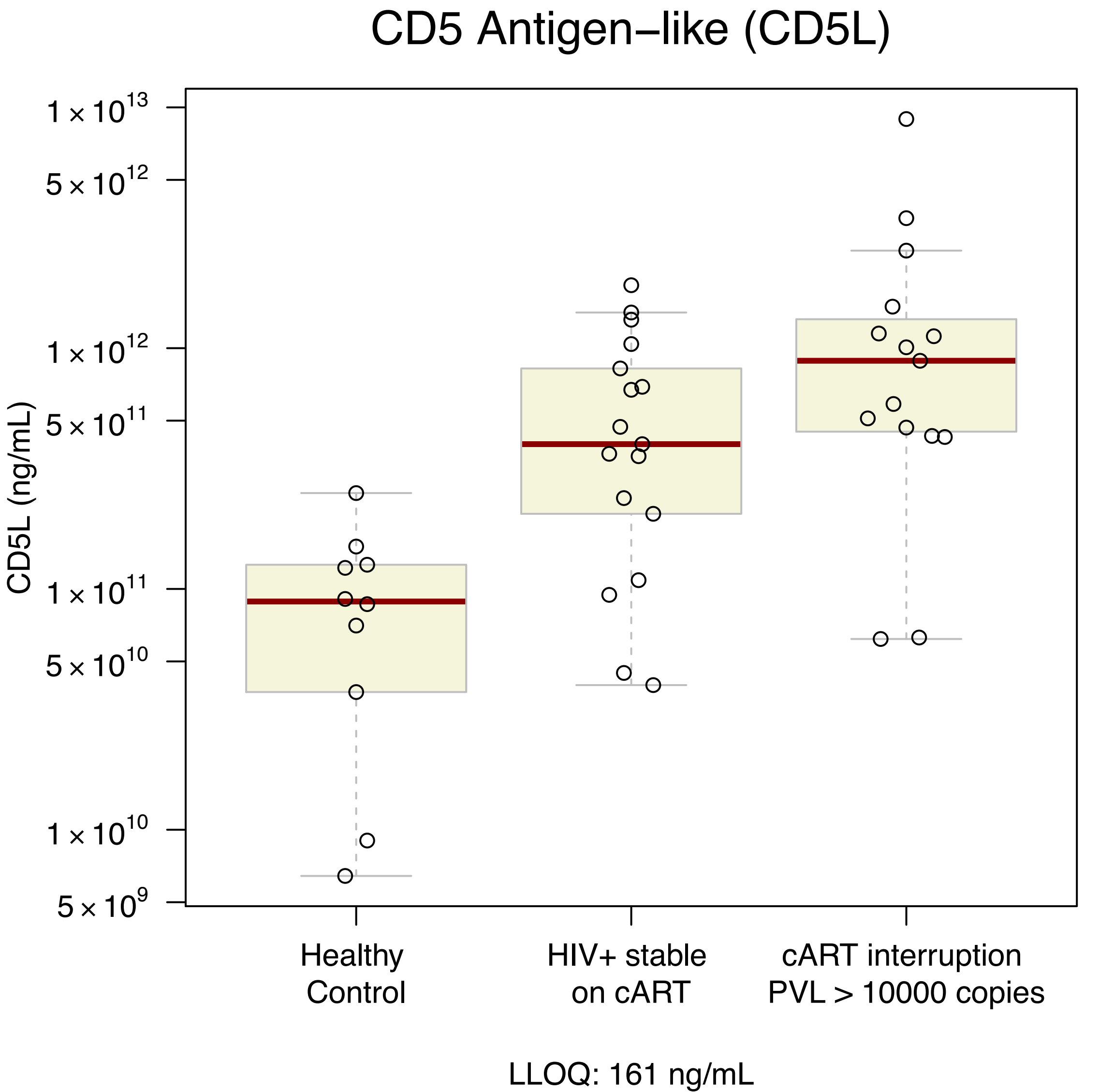
CD5 Antigen−like (CD5L)
CD5L (ng/mL)
1 × 10^13
5 × 10^12
1 × 10^12
5 × 10^11
1 × 10^11
5 × 10^10
1 × 10^10
5 × 10^9
Healthy Control
HIV+ stable on cART
cART interruption PVL > 10000 copies
LLOQ: 161 ng/mL

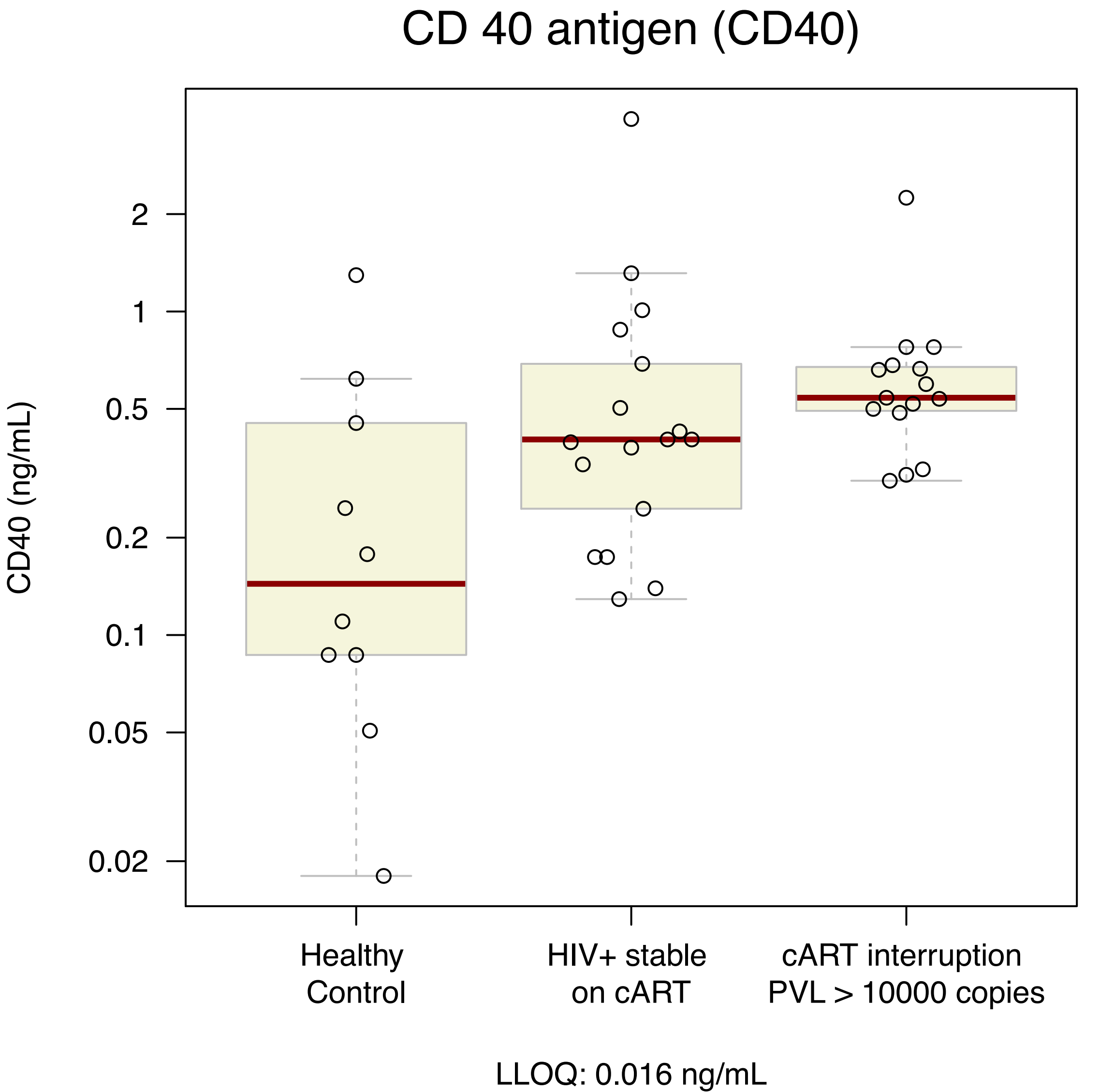

CD 40 antigen (CD40)
CD40 (ng/mL)
2
1
0.5
0.2
0.1
0.05
0.02
Healthy Control
HIV+ stable on cART
cART interruption PVL > 10000 copies
LLOQ: 0.016 ng/mL

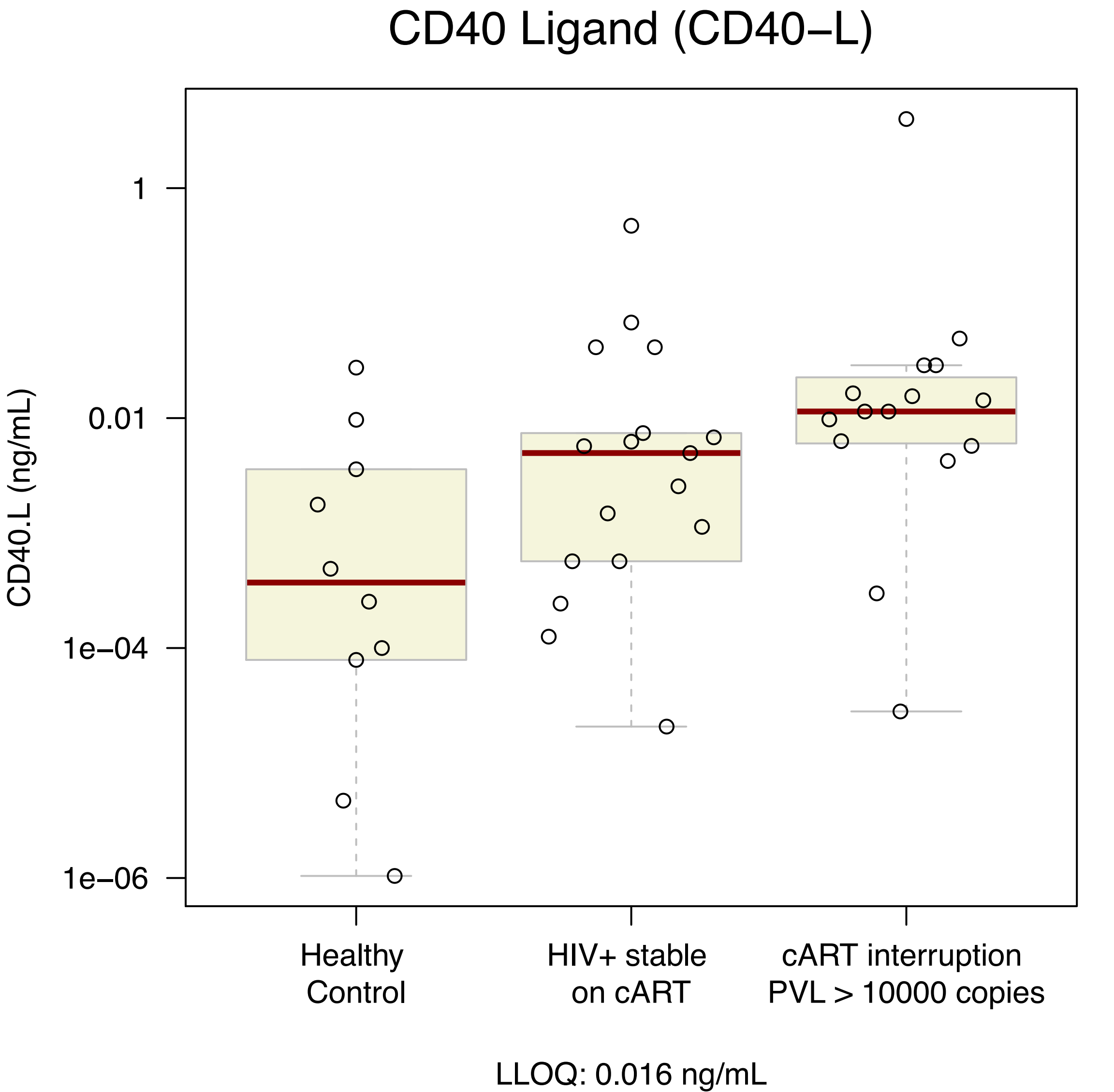

CD40 Ligand (CD40−L)
CD40.L (ng/mL)
1
0.01
1e−04
1e−06
Healthy Control
HIV+ stable on cART
cART interruption PVL > 10000 copies
LLOQ: 0.016 ng/mL

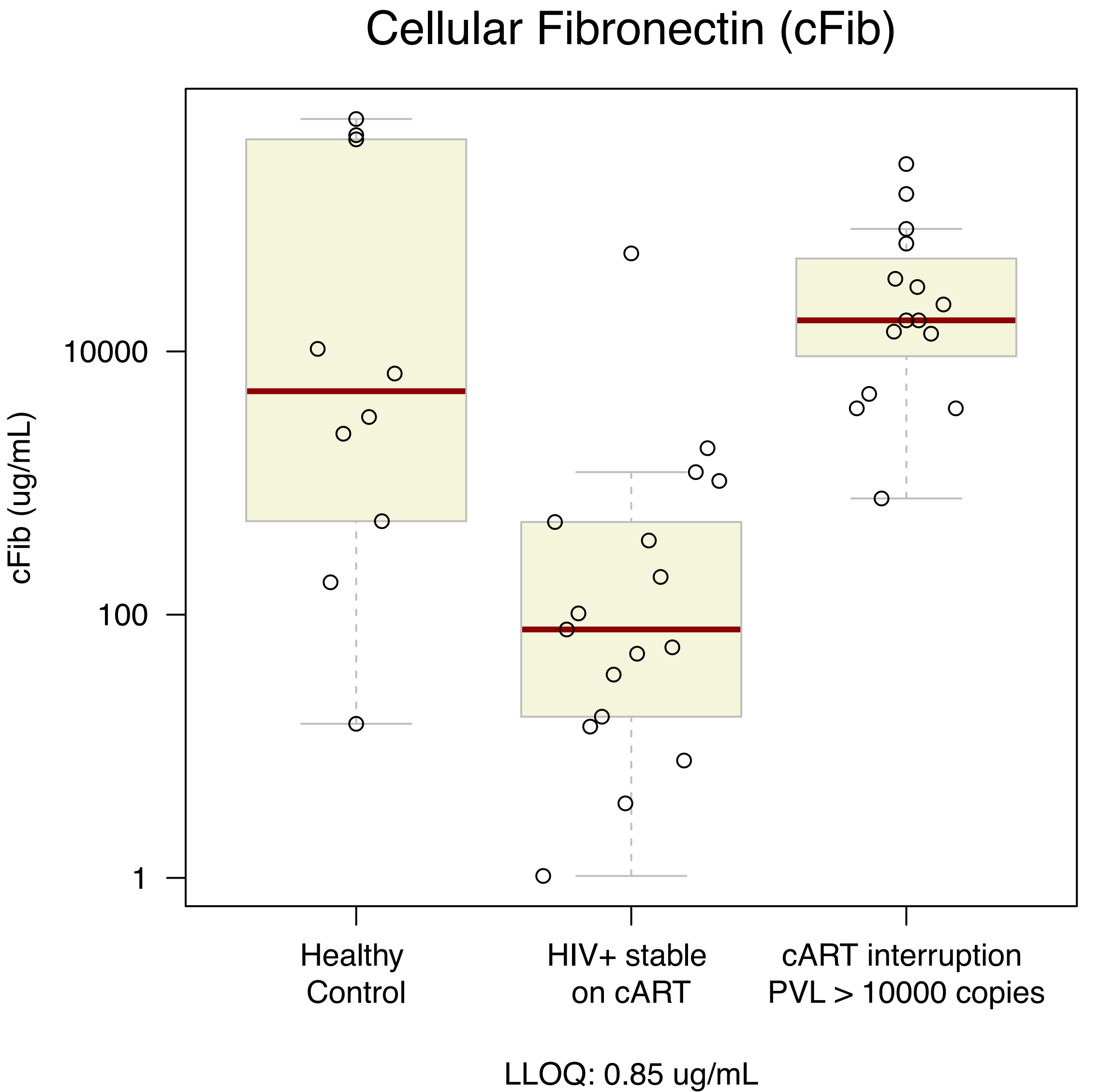

Cellular Fibronectin (cFib)
cFib (ug/mL)
10000
100
1
Healthy Control
HIV+ stable on cART
cART interruption PVL > 10000 copies
LLOQ: 0.85 ug/mL

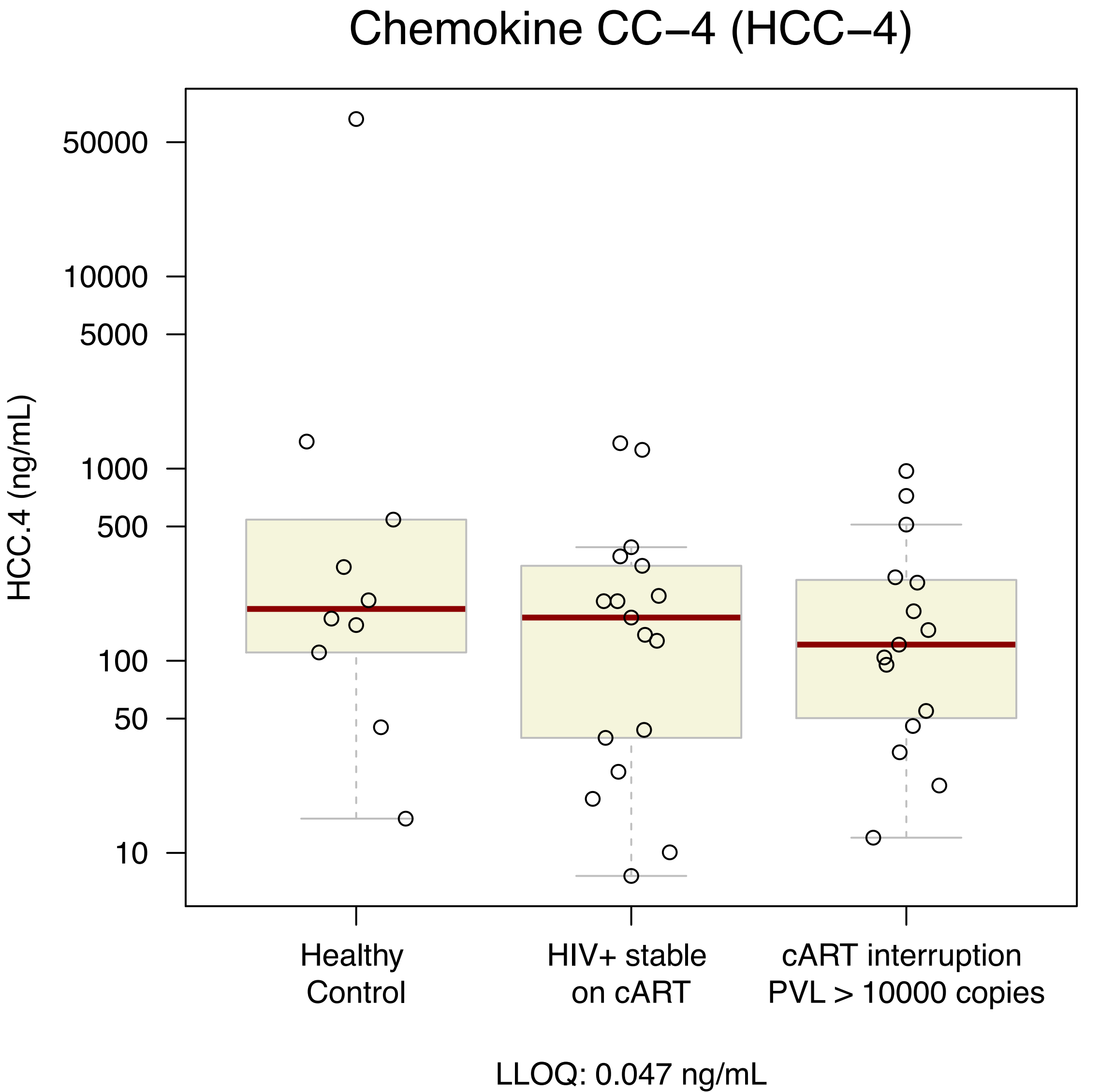
Chemokine CC−4 (HCC−4)
HCC.4 (ng/mL)
50000
10000
5000
1000
500
100
50
10
Healthy Control
HIV+ stable on cART
cART interruption PVL > 10000 copies
LLOQ: 0.047 ng/mL

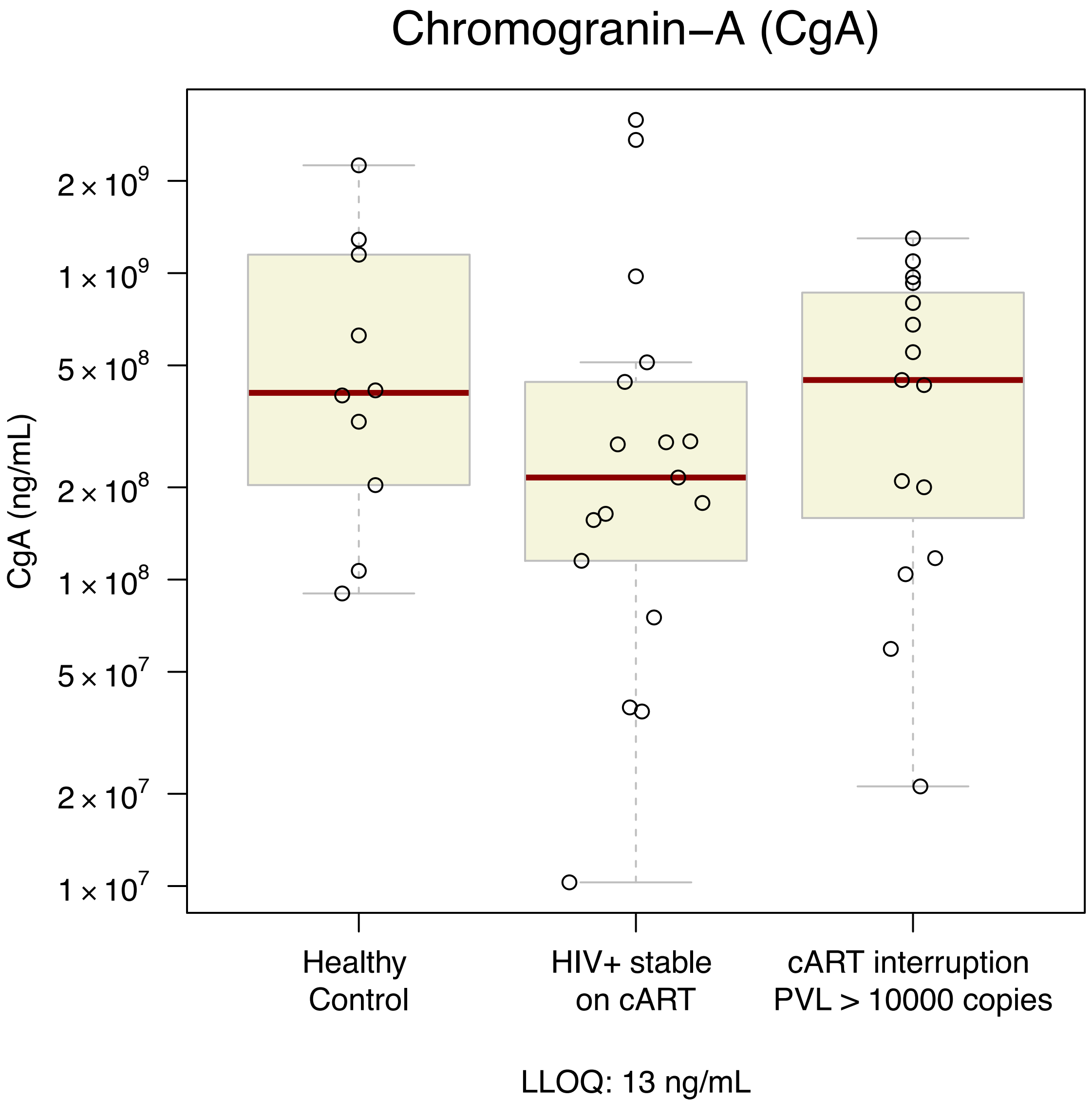
Chromogranin–A (CgA)
CgA (ng/mL)
2×10^9
1×10^9
5×10^8
2×10^8
1×10^8
5×10^7
2×10^7
1×10^7
Healthy Control
HIV+ stable on cART
cART interruption PVL > 10000 copies
LLOQ: 13 ng/mL

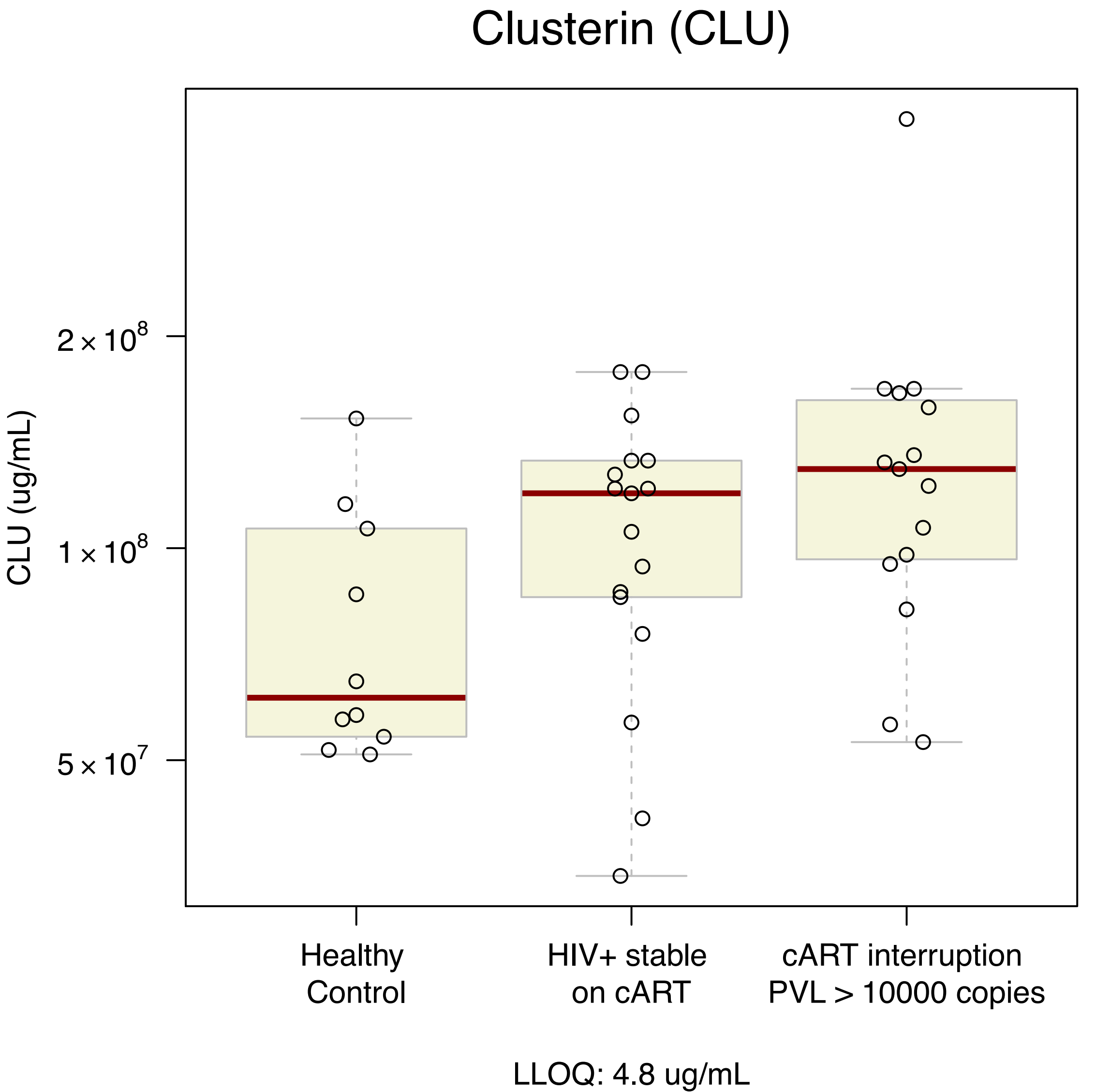
Clusterin (CLU)
CLU (ug/mL)
2×10^8
1×10^8
5×10^7
Healthy Control
HIV+ stable on cART
cART interruption PVL > 10000 copies
LLOQ: 4.8 ug/mL

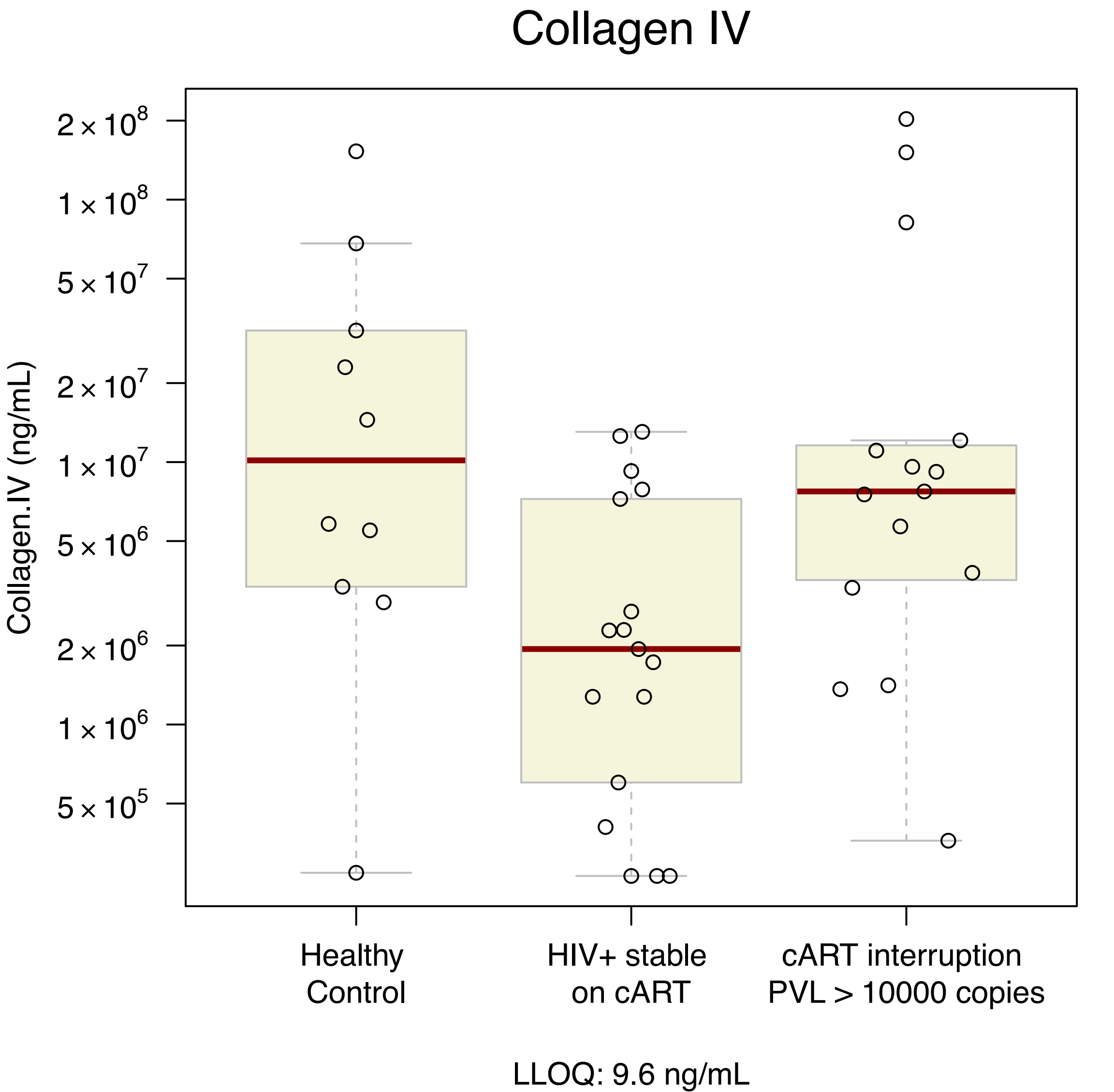

Collagen IV
Collagen.IV (ng/mL)
2×10^8
1×10^8
5×10^7
2×10^7
1×10^7
5×10^6
2×10^6
1×10^6
5×10^5
Healthy Control
HIV+ stable on cART
cART interruption PVL > 10000 copies
LLOQ: 9.6 ng/mL

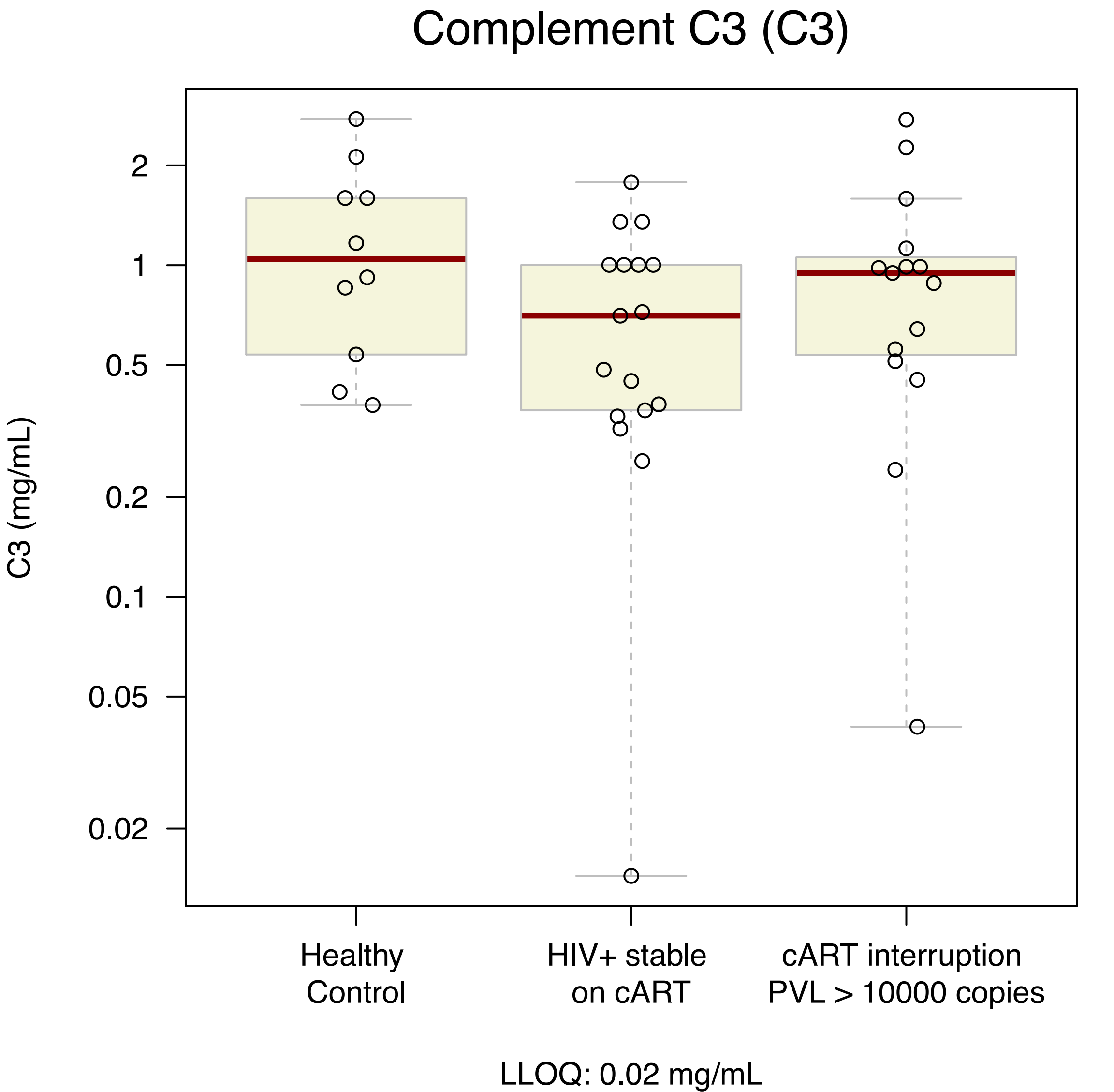
Complement C3 (C3)
C3 (mg/mL)
2
1
0.5
0.2
0.1
0.05
0.02
Healthy Control
HIV+ stable on cART
cART interruption PVL > 10000 copies
LLOQ: 0.02 mg/mL

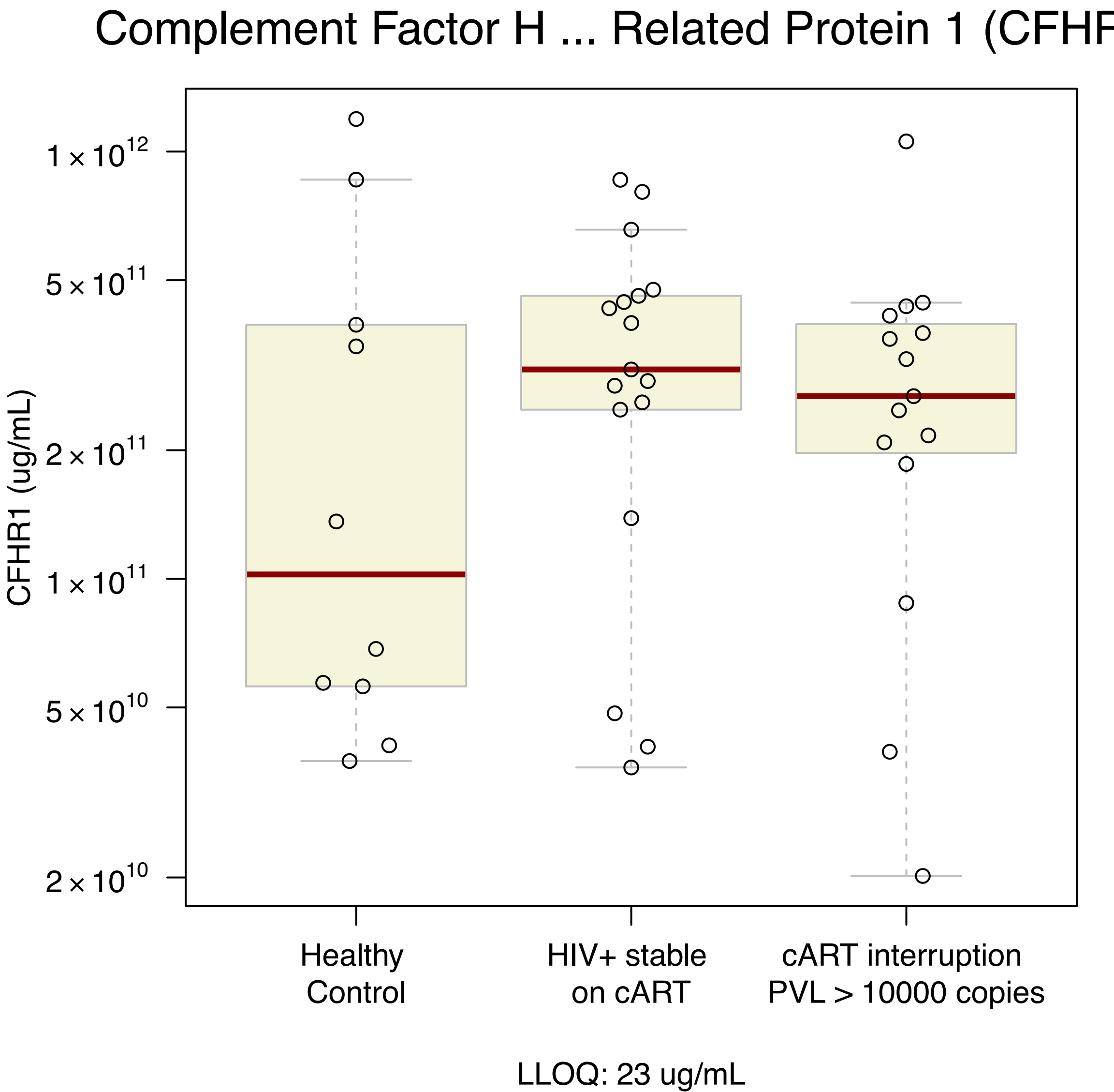

Complement Factor H ... Related Protein 1 (CFHR
CFHR1 (ug/mL)
1×10^{12}
5×10^{11}
2×10^{11}
1×10^{11}
5×10^{10}
2×10^{10}
Healthy Control
HIV+ stable on cART
cART interruption PVL > 10000 copies
LLOQ: 23 ug/mL

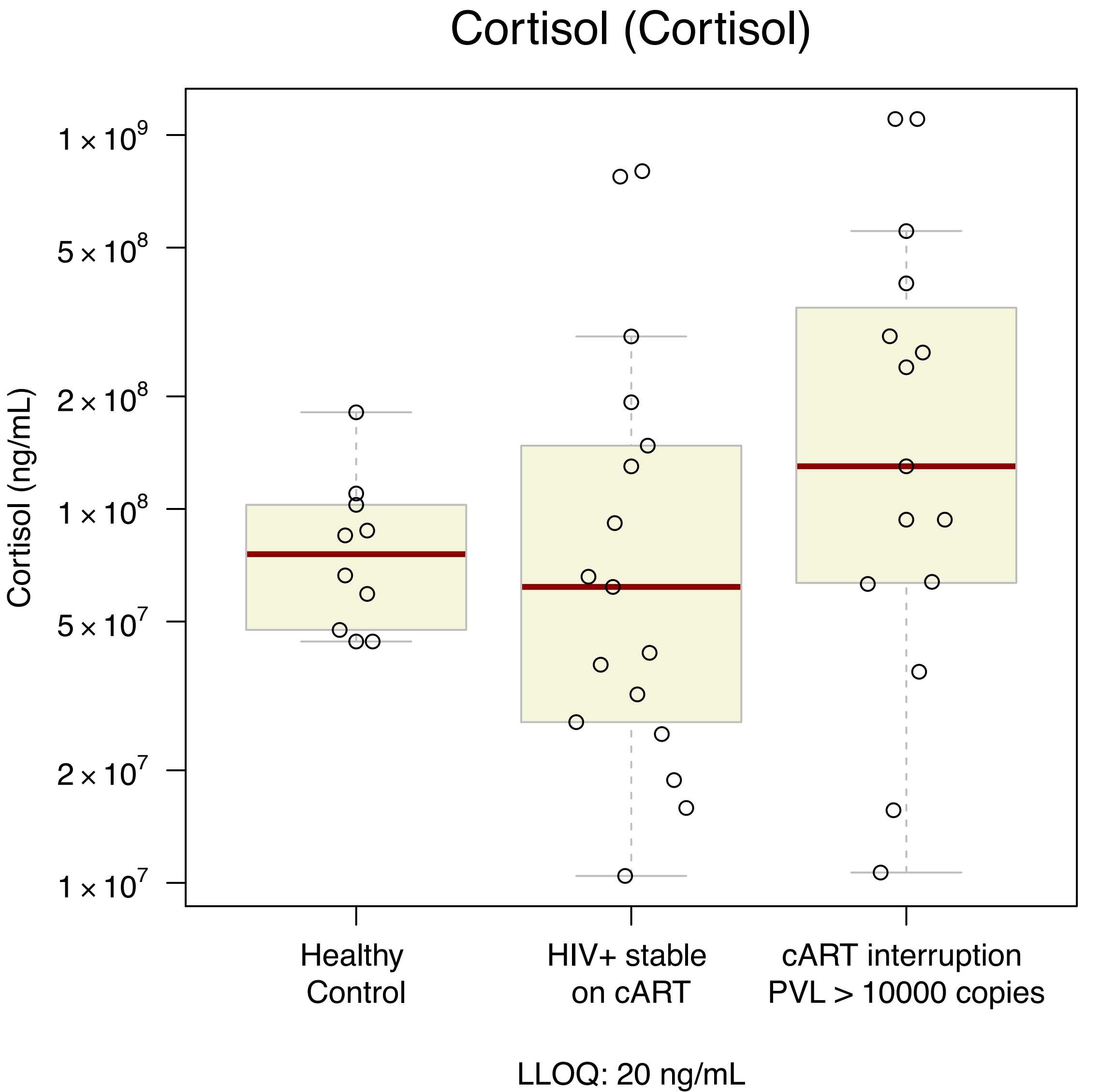

Cortisol (Cortisol)
Cortisol (ng/mL)
1 × 10^9
5 × 10^8
2 × 10^8
1 × 10^8
5 × 10^7
2 × 10^7
1 × 10^7
Healthy Control
HIV+ stable on cART
cART interruption PVL > 10000 copies
LLOQ: 20 ng/mL

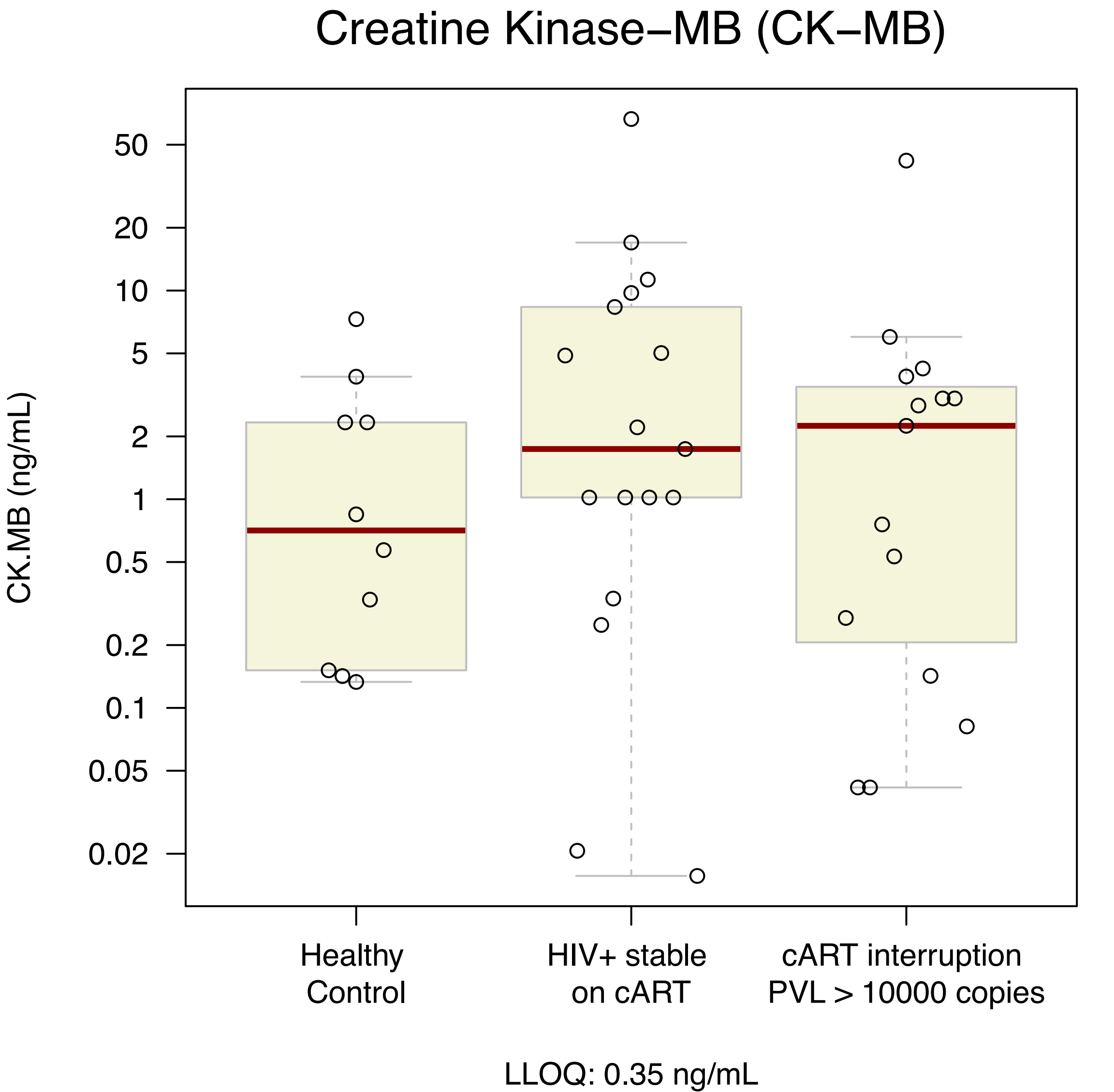
Creatine Kinase−MB (CK−MB)
CK.MB (ng/mL)
50
20
10
5
2
1
0.5
0.2
0.1
0.05
0.02
Healthy Control
HIV+ stable on cART
cART interruption PVL > 10000 copies
LLOQ: 0.35 ng/mL

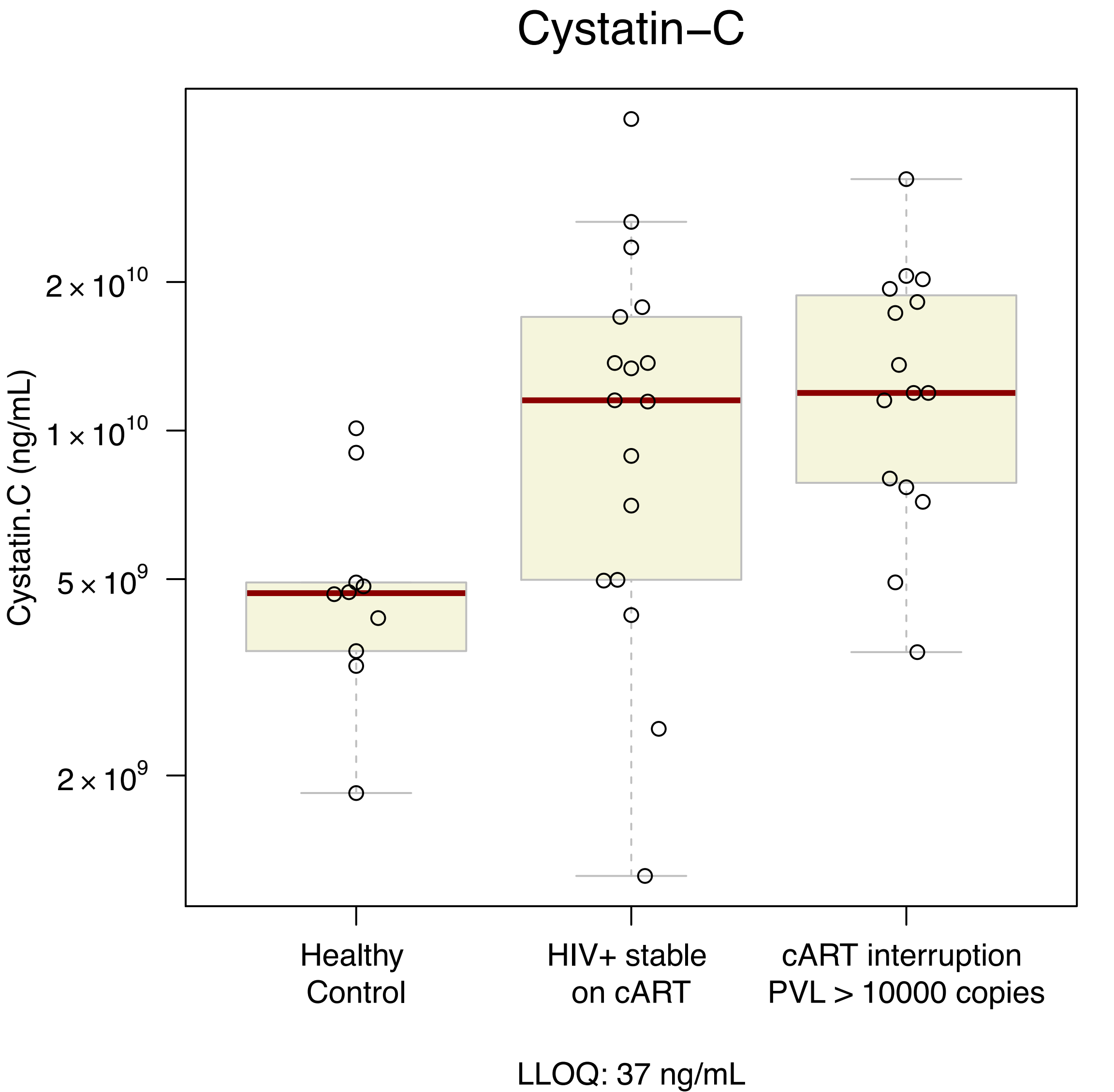

Cystatin−C
Cystatin.C (ng/mL)
2 × 10^10
1 × 10^10
5 × 10^9
2 × 10^9
Healthy Control
HIV+ stable on cART
cART interruption PVL > 10000 copies
LLOQ: 37 ng/mL

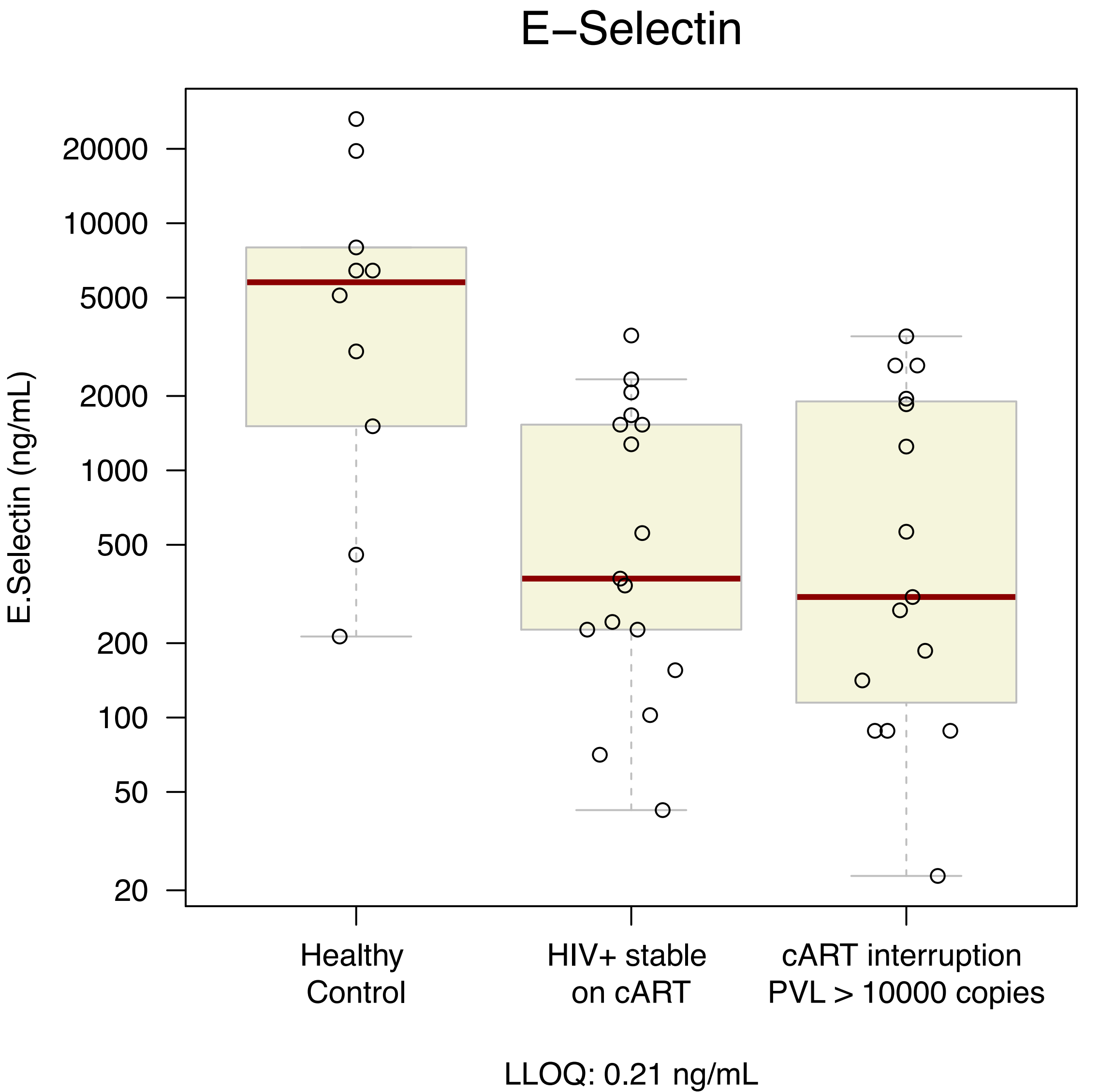

E−Selectin
E.Selectin (ng/mL)
20000
10000
5000
2000
1000
500
200
100
50
20
Healthy Control
HIV+ stable on cART
cART interruption PVL > 10000 copies
LLOQ: 0.21 ng/mL

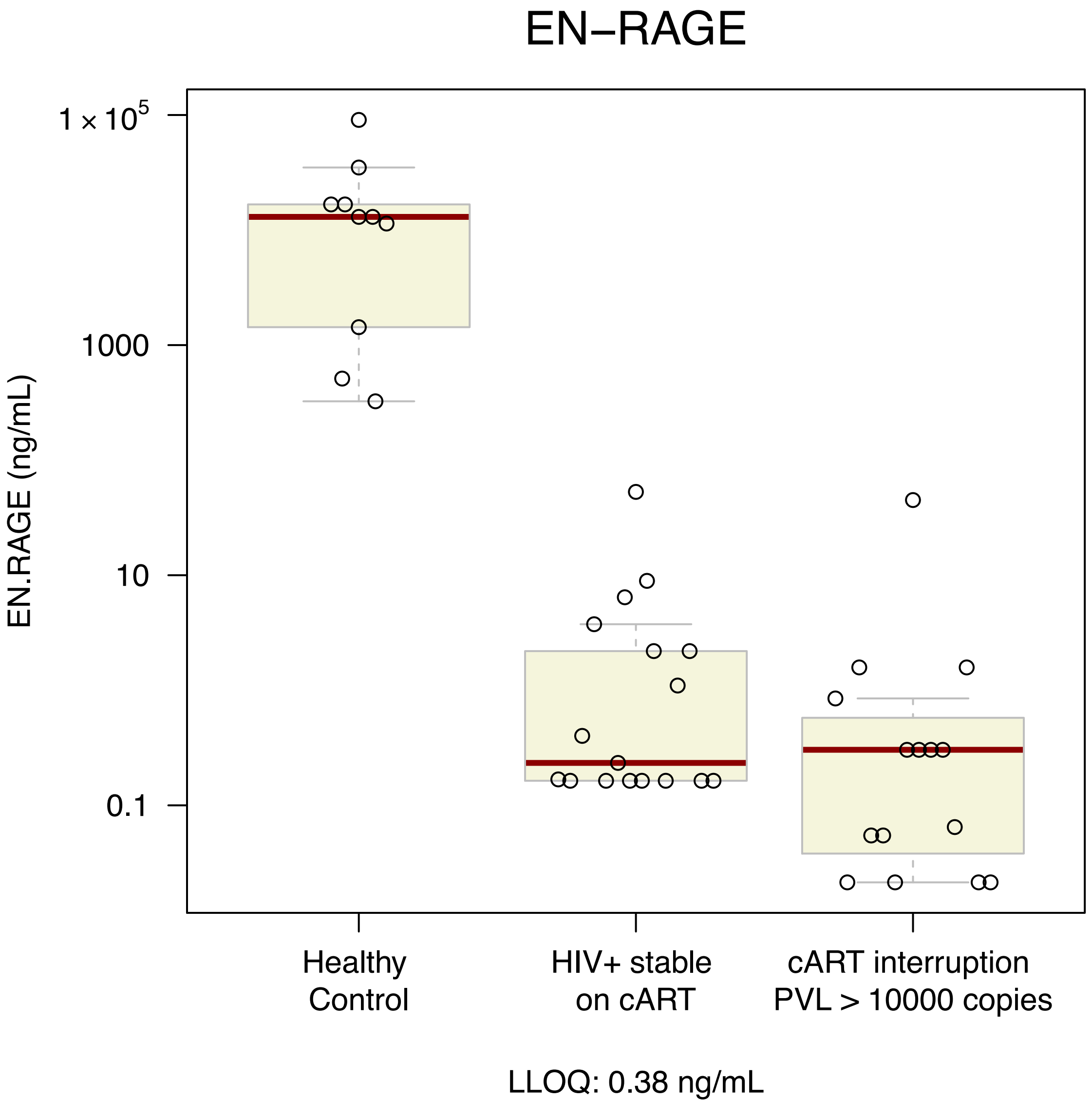
EN−RAGE
EN.RAGE (ng/mL)
1×10^5
1000
10
0.1
Healthy Control
HIV+ stable on cART
cART interruption PVL > 10000 copies
LLOQ: 0.38 ng/mL

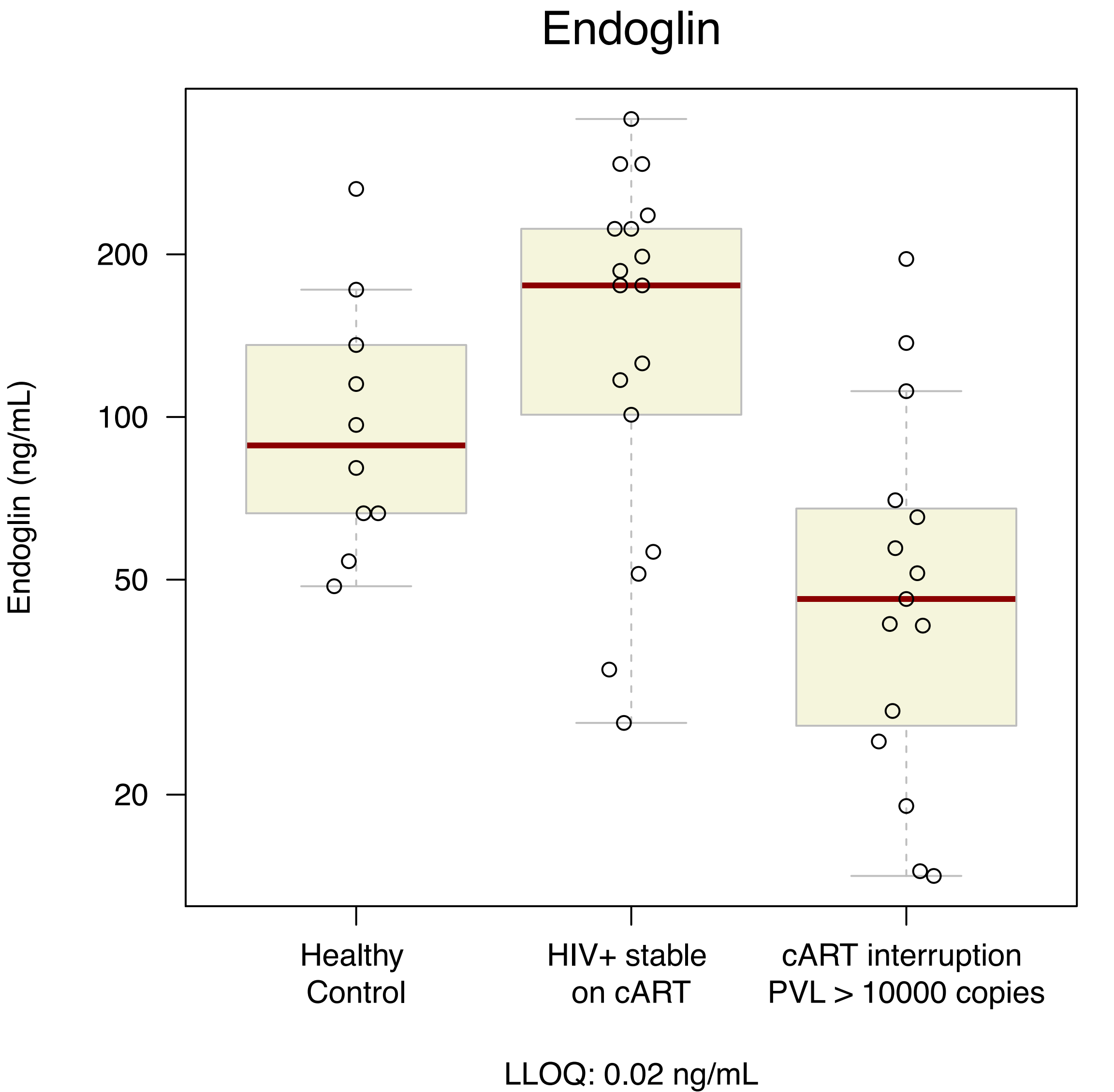

Endoglin
Endoglin (ng/mL)
200
100
50
20
Healthy
Control
HIV+ stable
on cART
cART interruption
PVL > 10000 copies
LLOQ: 0.02 ng/mL

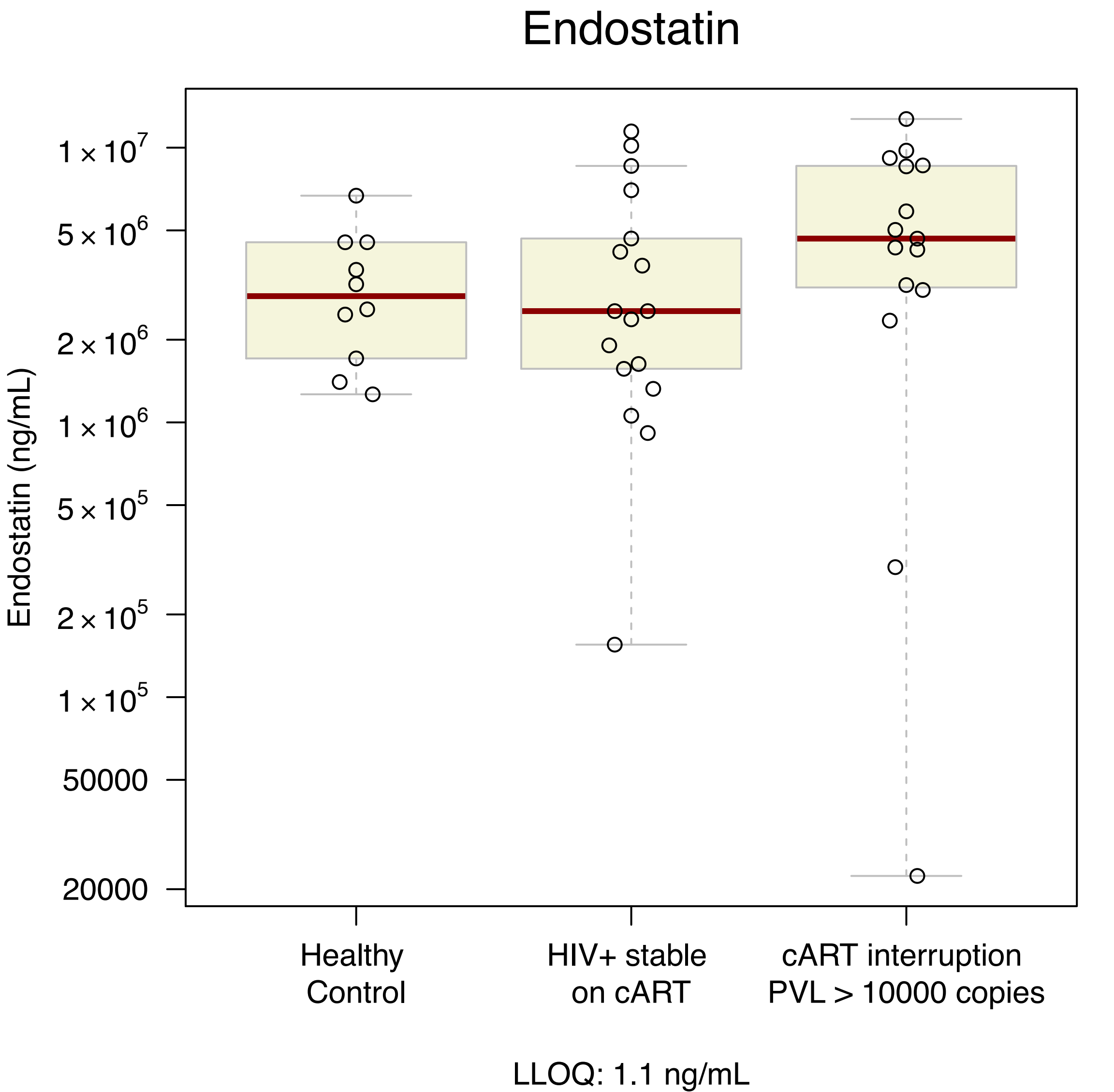

Endostatin
Endostatin (ng/mL)
1×10^7
5×10^6
2×10^6
1×10^6
5×10^5
2×10^5
1×10^5
50000
20000
Healthy Control
HIV+ stable on cART
cART interruption PVL > 10000 copies
LLOQ: 1.1 ng/mL

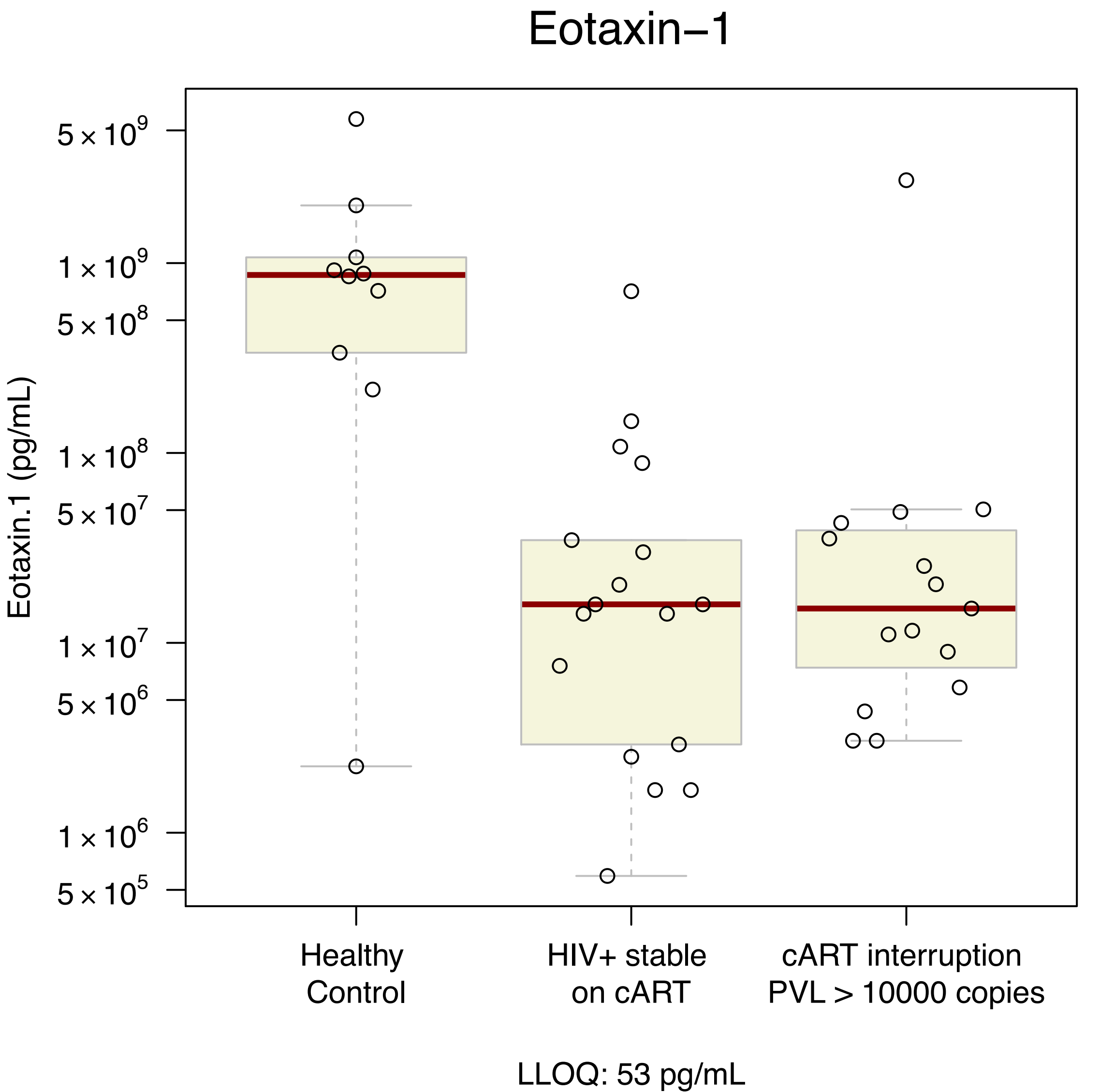
Eotaxin−1
Eotaxin.1 (pg/mL)
5 × 10^9
1 × 10^9
5 × 10^8
1 × 10^8
5 × 10^7
1 × 10^7
5 × 10^6
1 × 10^6
5 × 10^5
Healthy Control
HIV+ stable on cART
cART interruption PVL > 10000 copies
LLOQ: 53 pg/mL

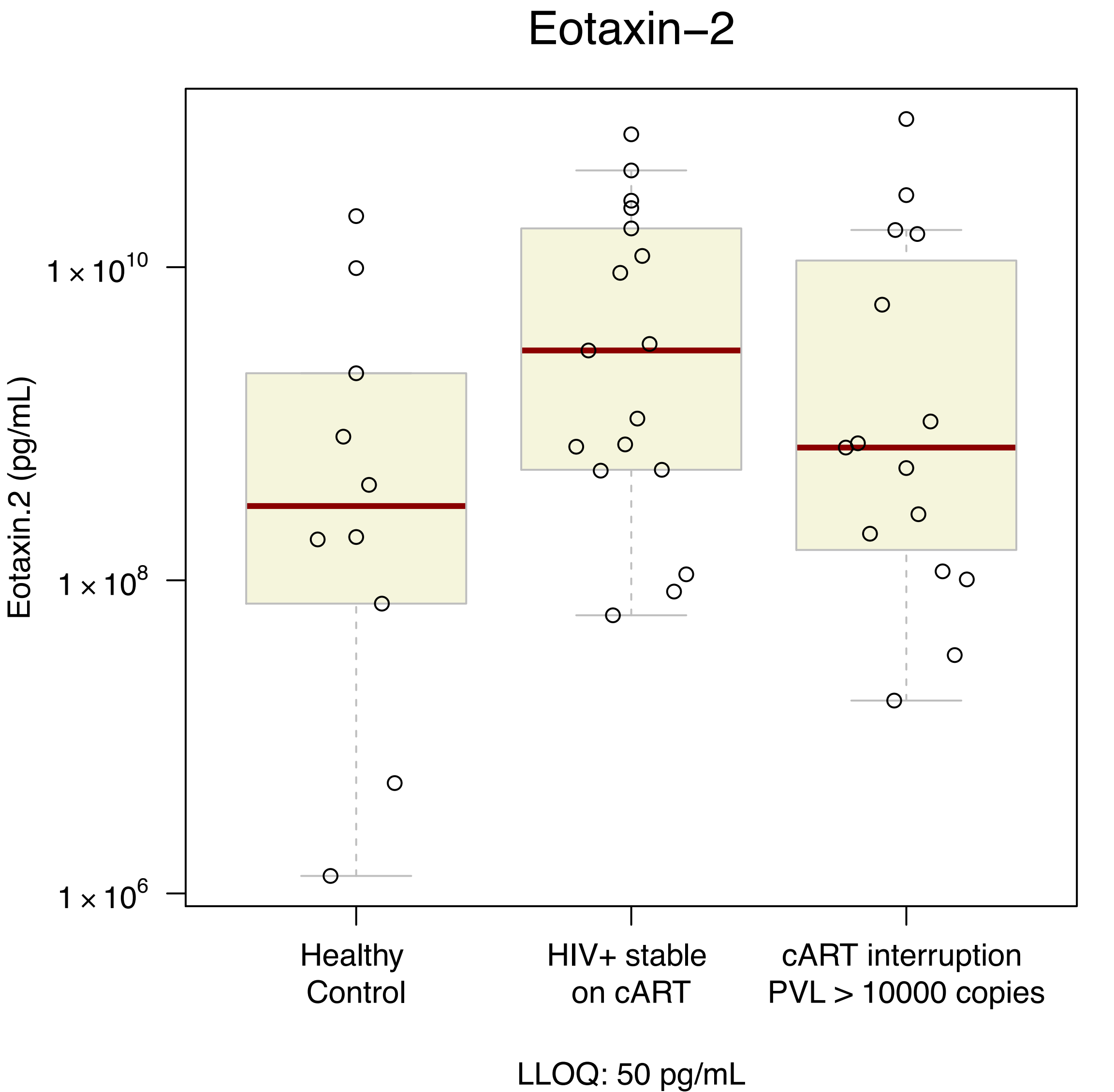

Eotaxin−2
Eotaxin.2 (pg/mL)
1 × 10^10
1 × 10^8
1 × 10^6
Healthy Control
HIV+ stable on cART
cART interruption PVL > 10000 copies
LLOQ: 50 pg/mL

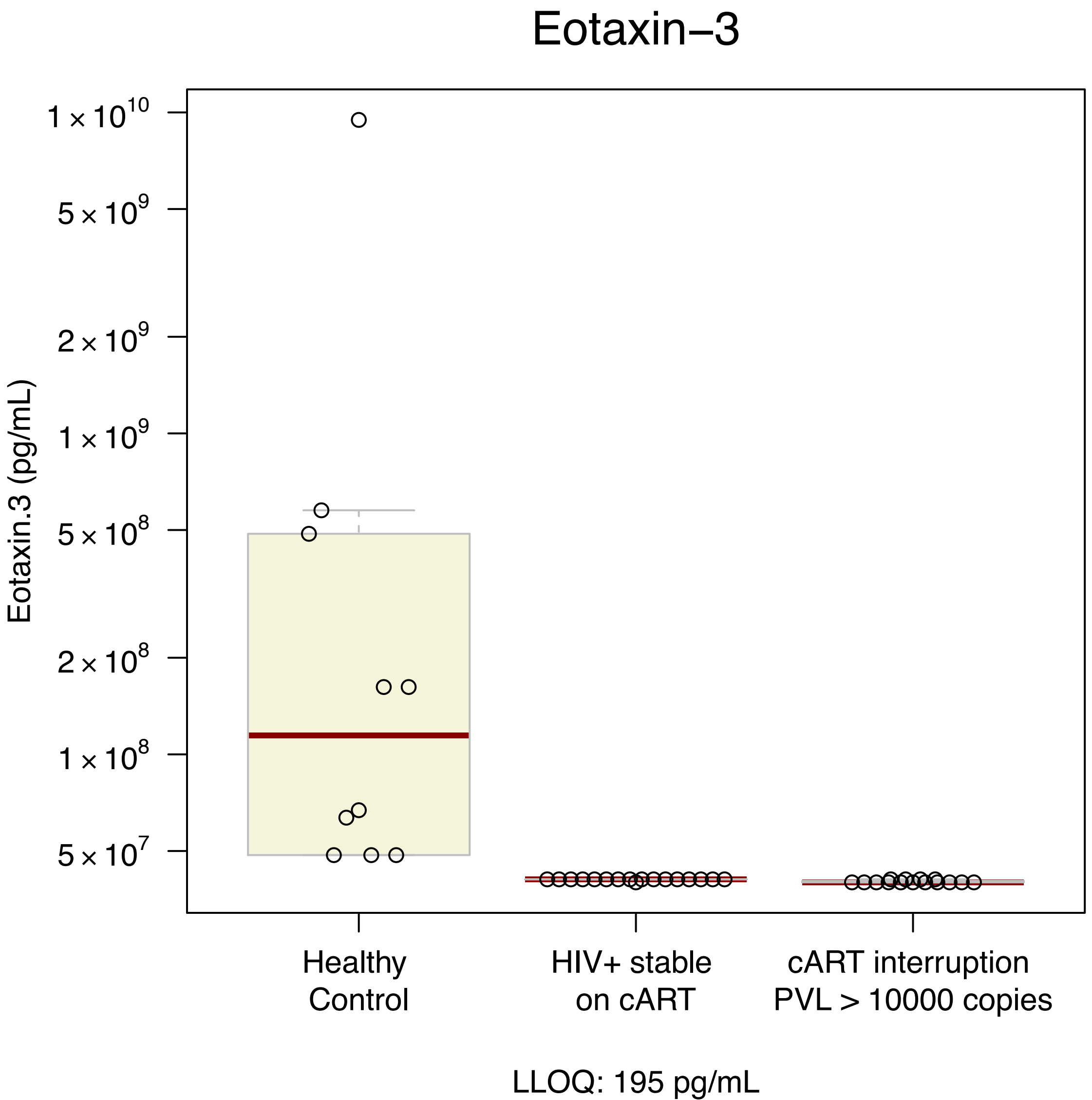

Eotaxin−3
Eotaxin.3 (pg/mL)
1 × 10^10
5 × 10^9
2 × 10^9
1 × 10^9
5 × 10^8
2 × 10^8
1 × 10^8
5 × 10^7
Healthy Control
HIV+ stable on cART
cART interruption PVL > 10000 copies
LLOQ: 195 pg/mL

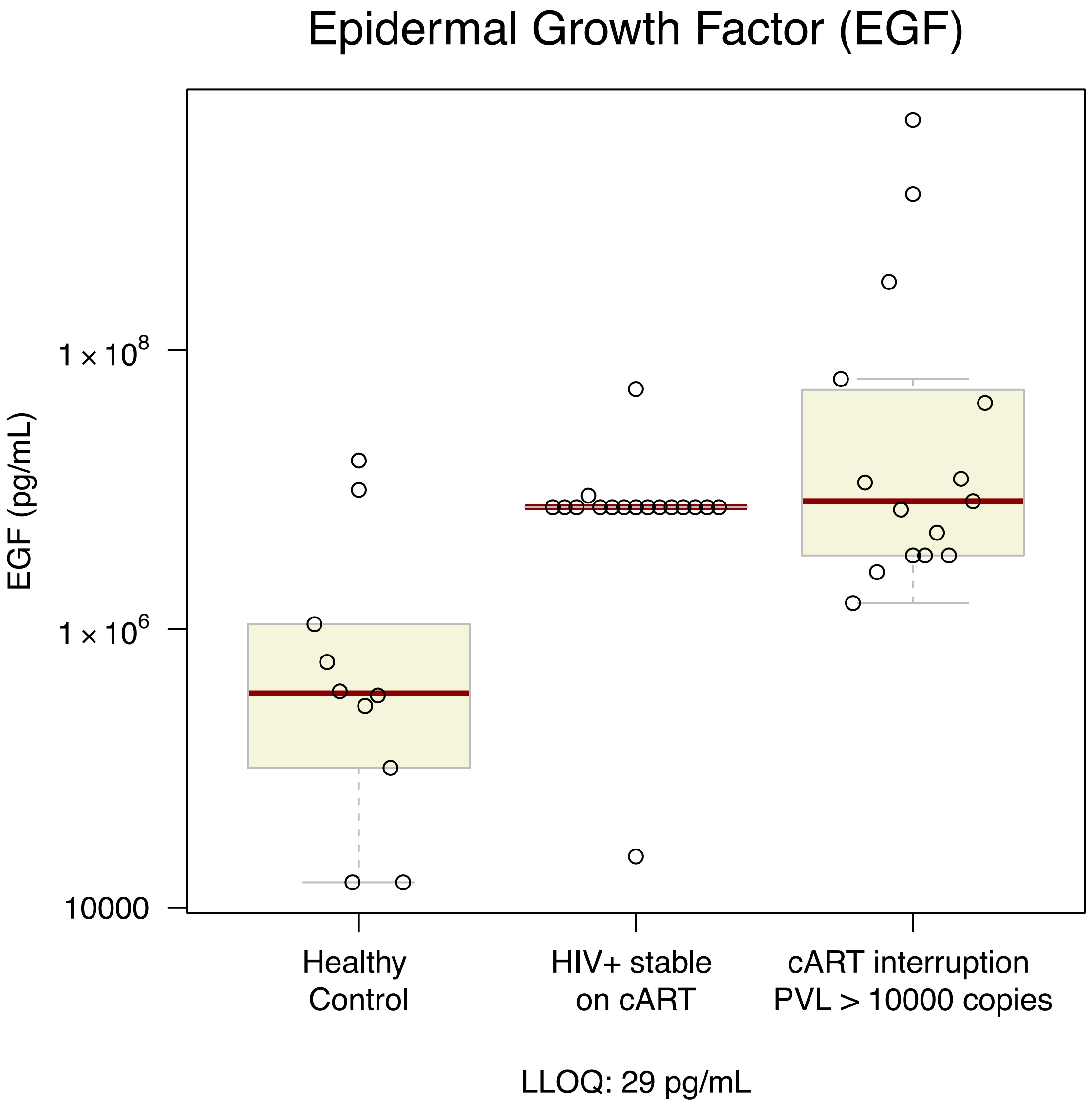

Epidermal Growth Factor (EGF)
EGF (pg/mL)
1×10^8
1×10^6
10000
Healthy Control
HIV+ stable on cART
cART interruption PVL > 10000 copies
LLOQ: 29 pg/mL

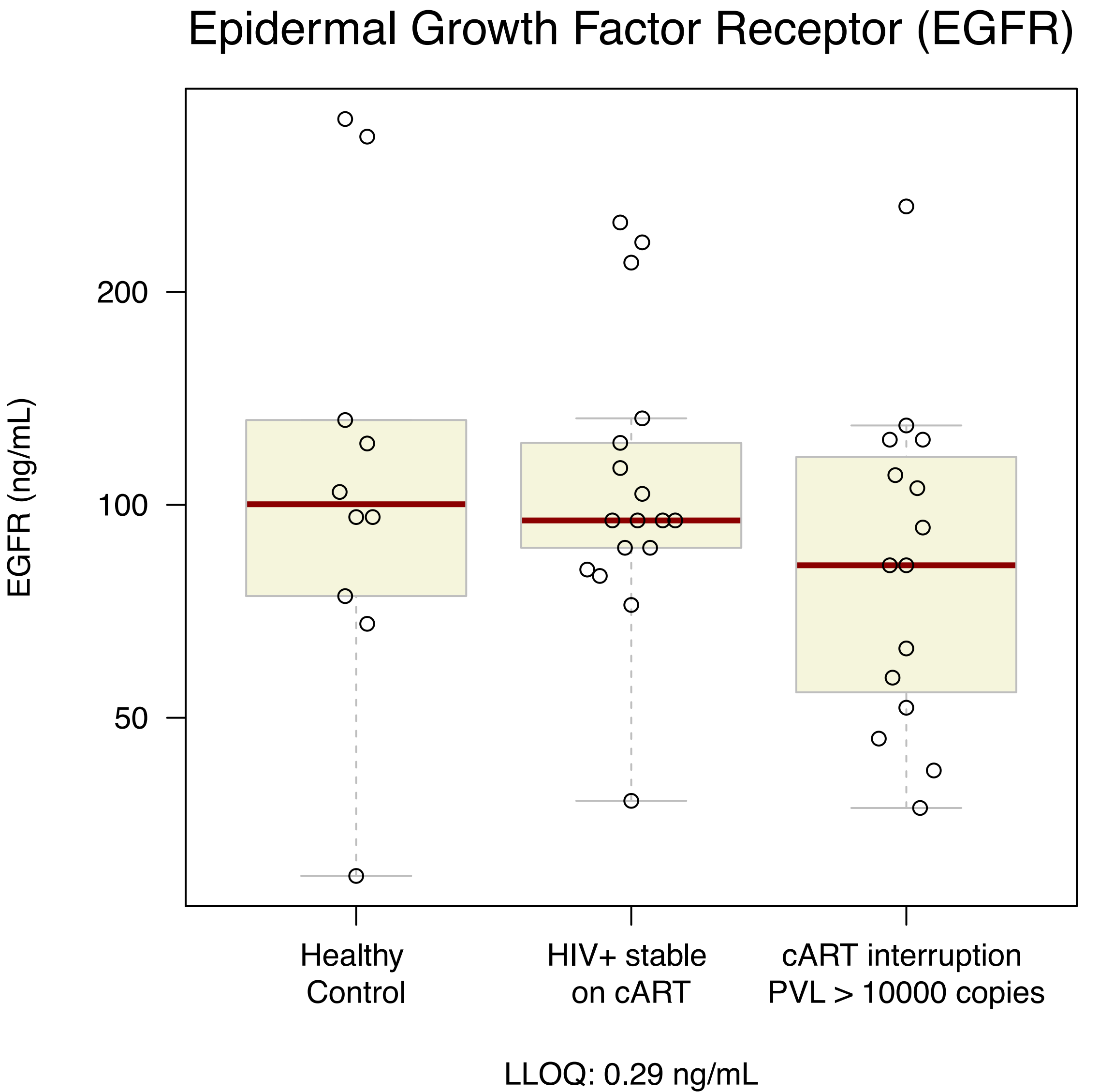
Epidermal Growth Factor Receptor (EGFR)
EGFR (ng/mL)
200
100
50
Healthy Control
HIV+ stable on cART
cART interruption PVL > 10000 copies
LLOQ: 0.29 ng/mL

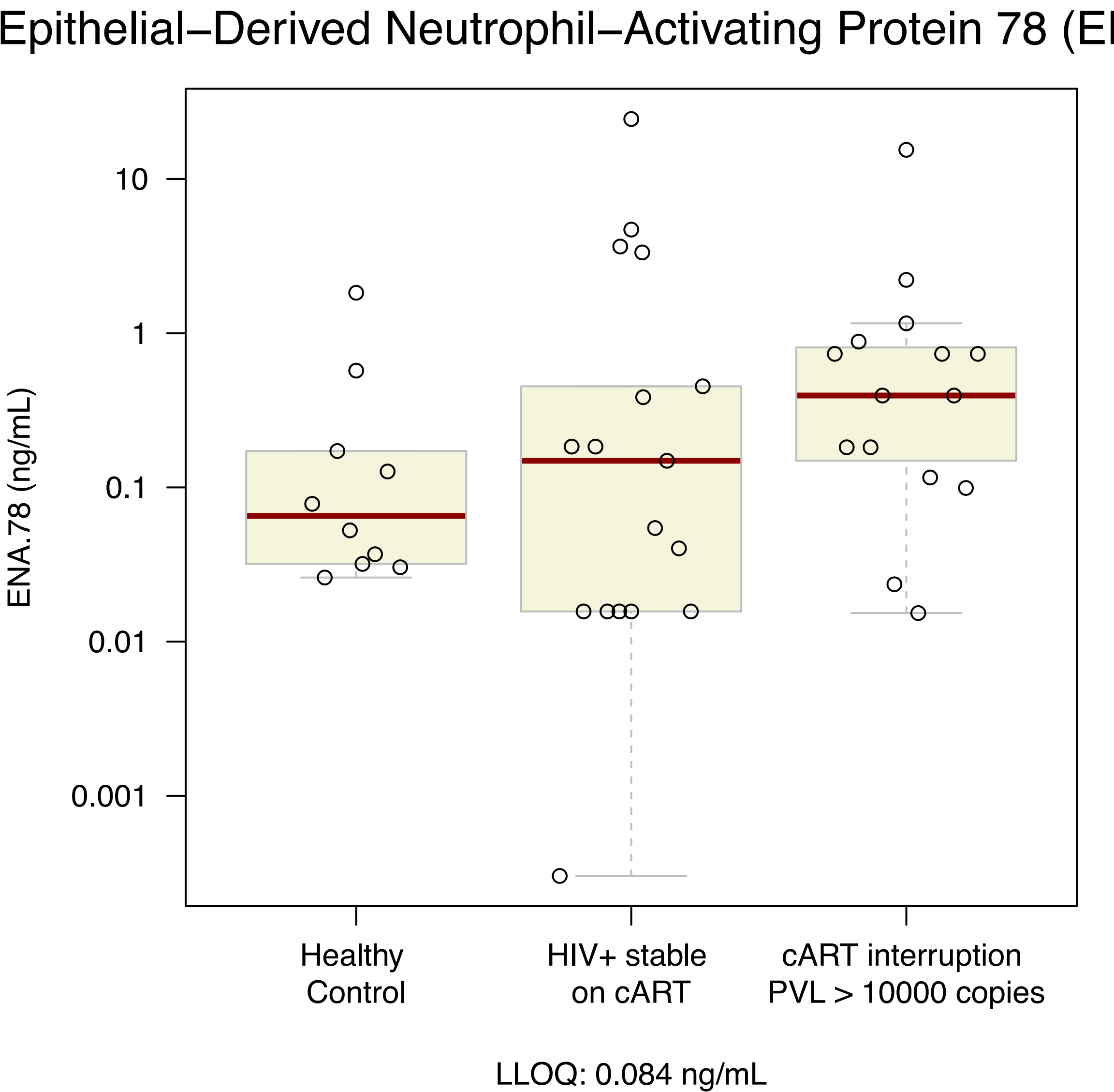

Epithelial–Derived Neutrophil–Activating Protein 78 (EI
ENA.78 (ng/mL)
10
1
0.1
0.01
0.001
Healthy Control
HIV+ stable on cART
cART interruption PVL > 10000 copies
LLOQ: 0.084 ng/mL

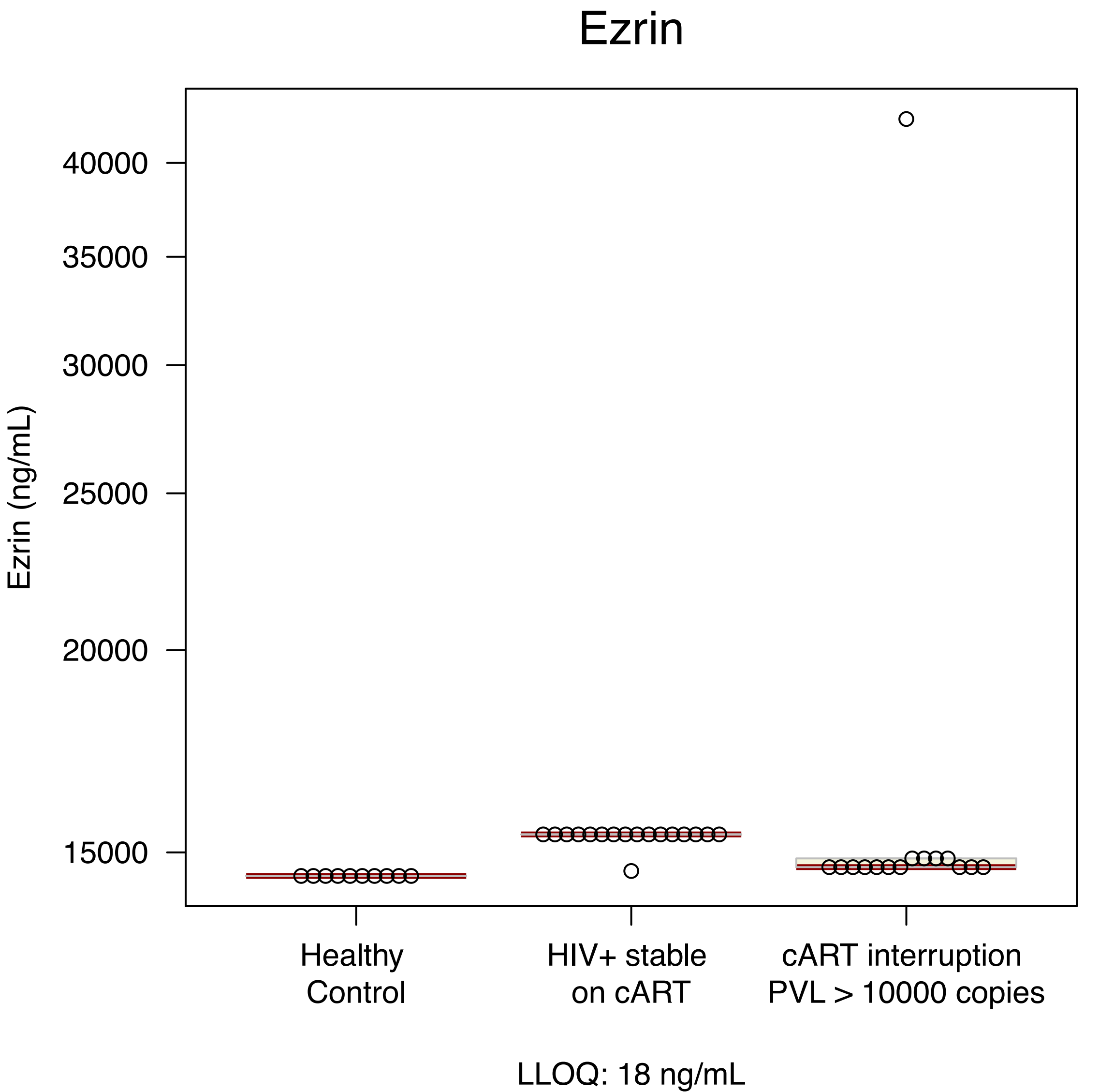

Ezrin
Ezrin (ng/mL)
40000
35000
30000
25000
20000
15000
Healthy Control
HIV+ stable on cART
cART interruption PVL > 10000 copies
LLOQ: 18 ng/mL

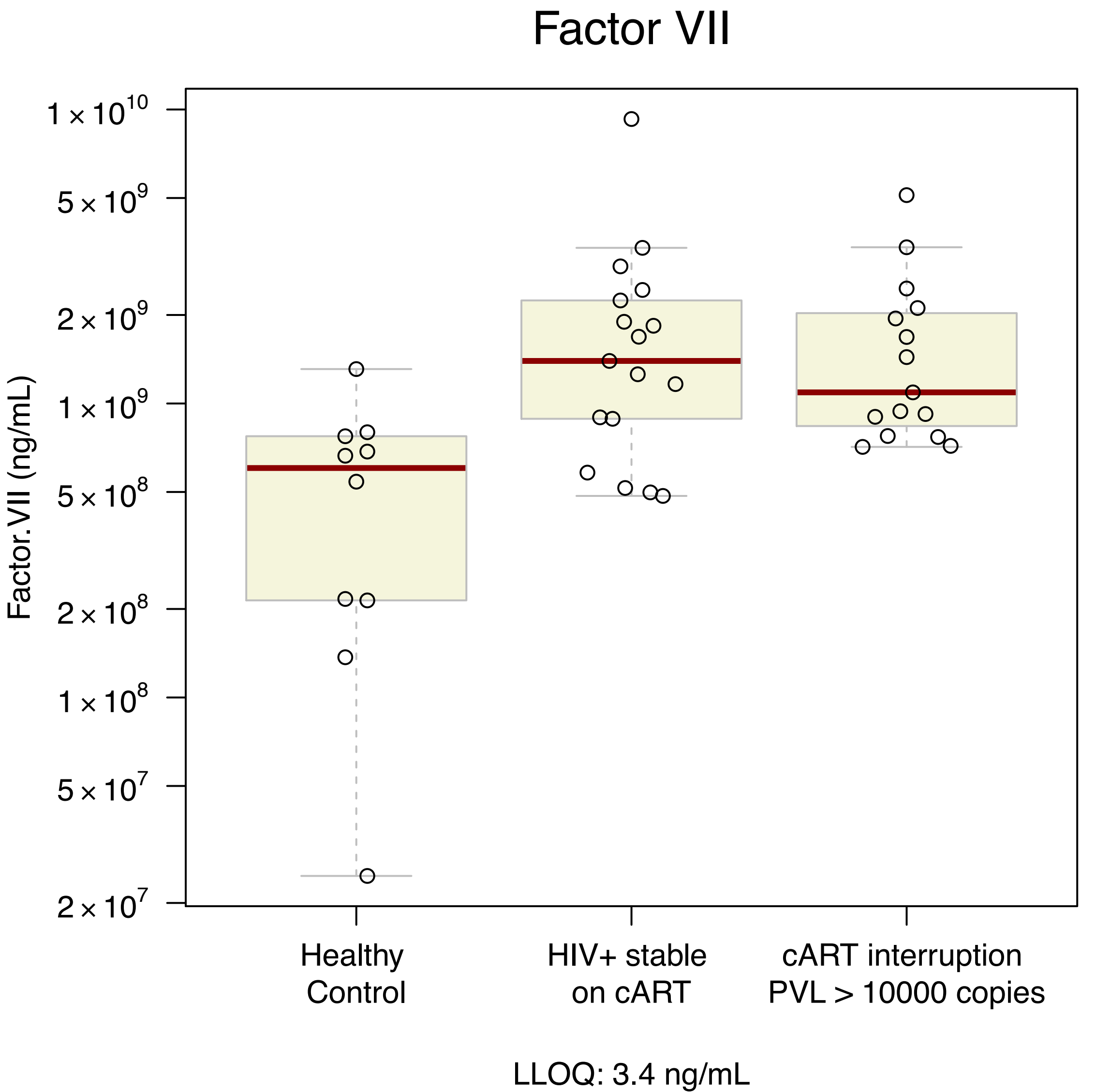

Factor VII
Factor.VII (ng/mL)
1 × 10^10
5 × 10^9
2 × 10^9
1 × 10^9
5 × 10^8
2 × 10^8
1 × 10^8
5 × 10^7
2 × 10^7
Healthy Control
HIV+ stable on cART
cART interruption PVL > 10000 copies
LLOQ: 3.4 ng/mL

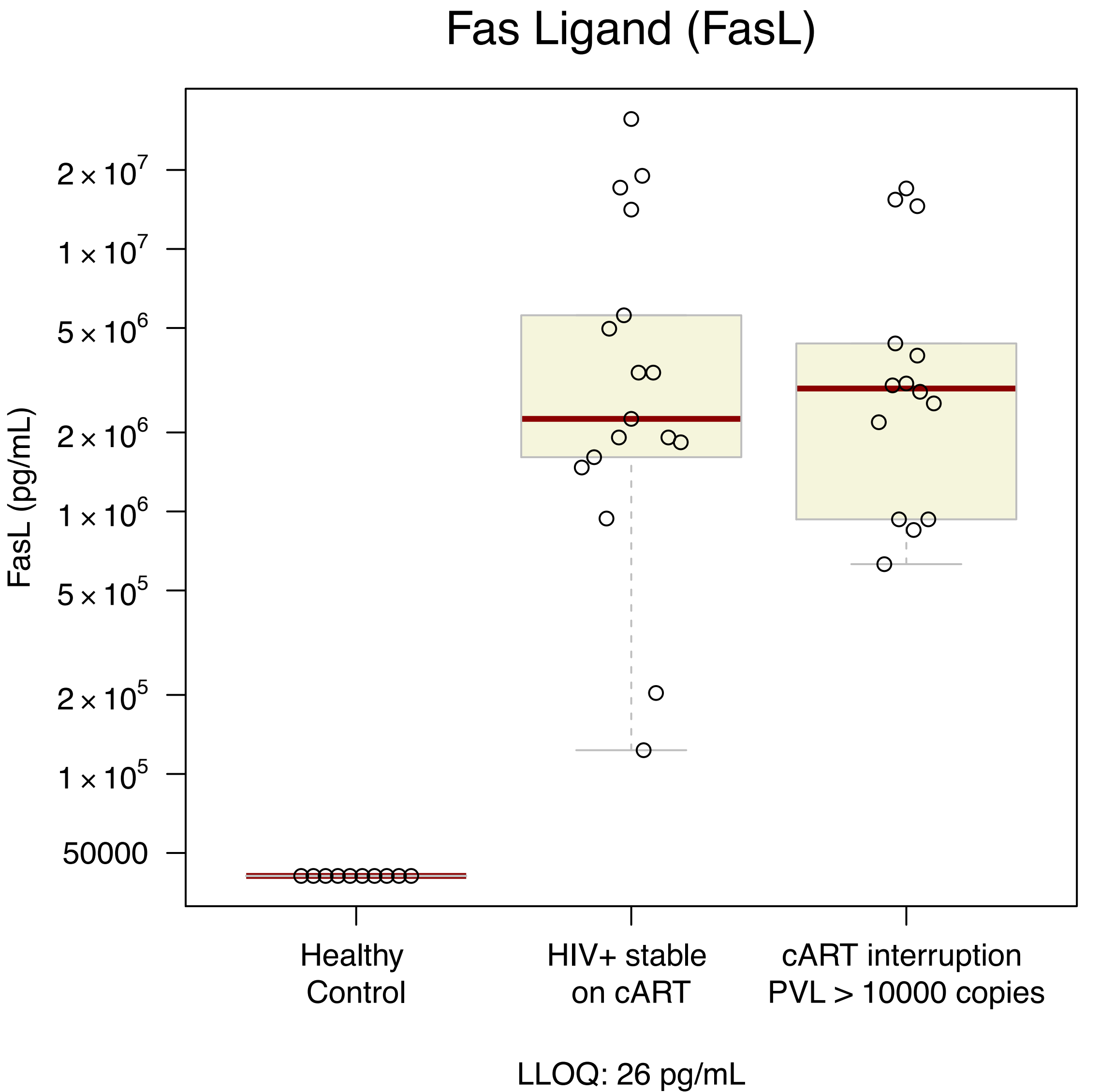
Fas Ligand (FasL)
FasL (pg/mL)
2 × 10^7
1 × 10^7
5 × 10^6
2 × 10^6
1 × 10^6
5 × 10^5
2 × 10^5
1 × 10^5
50000
Healthy Control
HIV+ stable on cART
cART interruption PVL > 10000 copies
LLOQ: 26 pg/mL

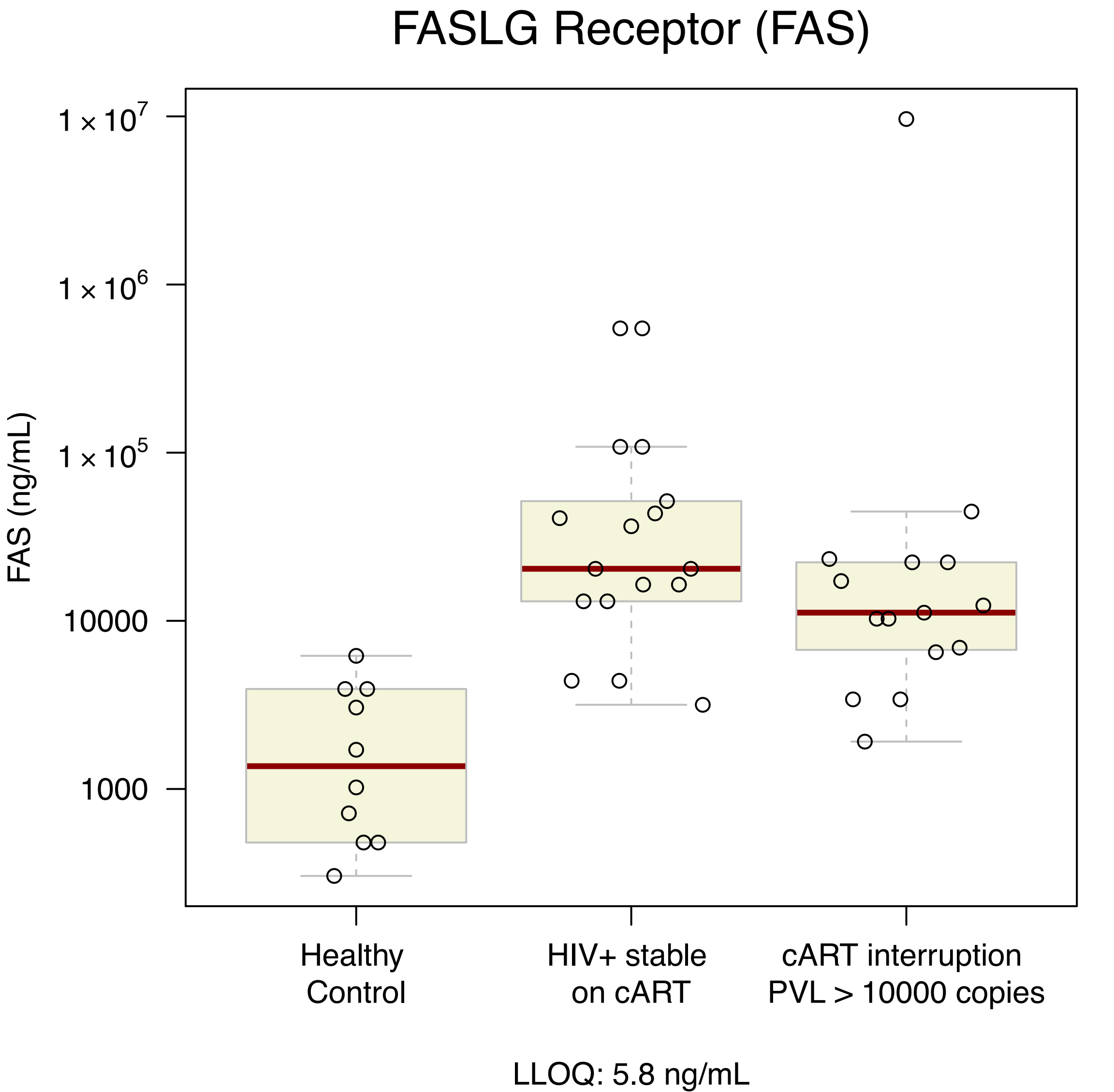
FASLG Receptor (FAS)
FAS (ng/mL)
1×10^7
1×10^6
1×10^5
10000
1000
Healthy Control
HIV+ stable on cART
cART interruption PVL > 10000 copies
LLOQ: 5.8 ng/mL

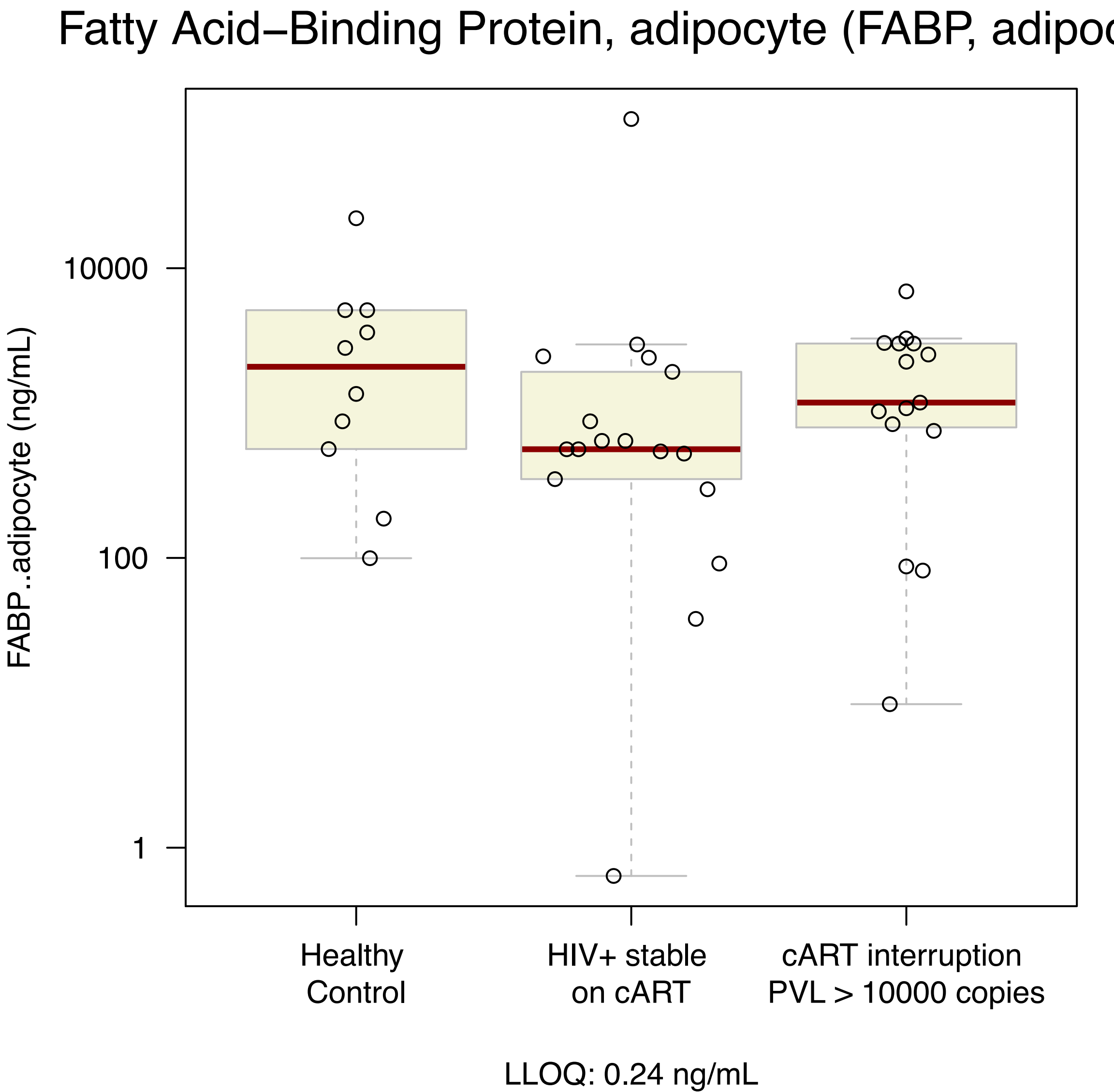
Fatty Acid–Binding Protein, adipocyte (FABP, adipoc
FABP..adipocyte (ng/mL)
10000
100
1
Healthy Control
HIV+ stable on cART
cART interruption PVL > 10000 copies
LLOQ: 0.24 ng/mL

Ferritin (FRTN)

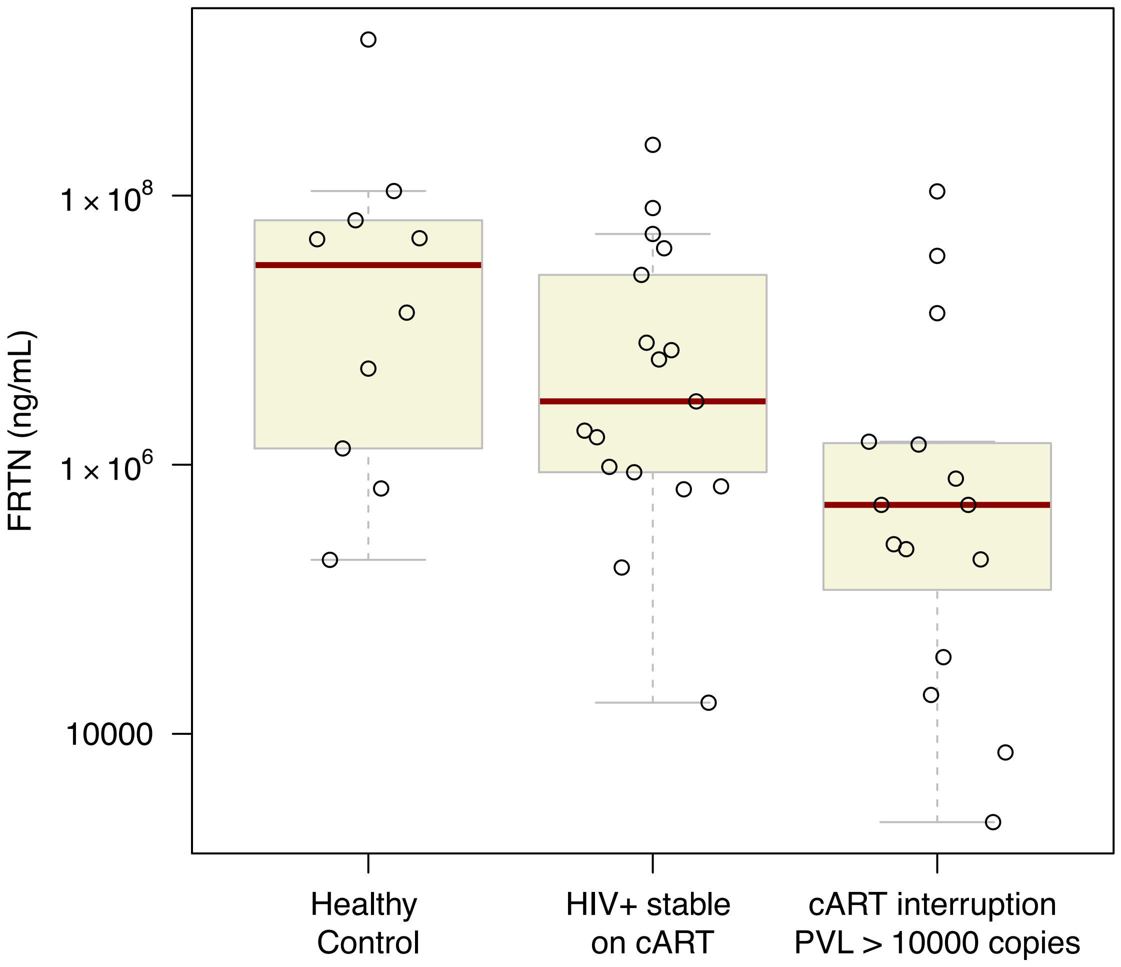

LLOQ: 4.3 ng/mL

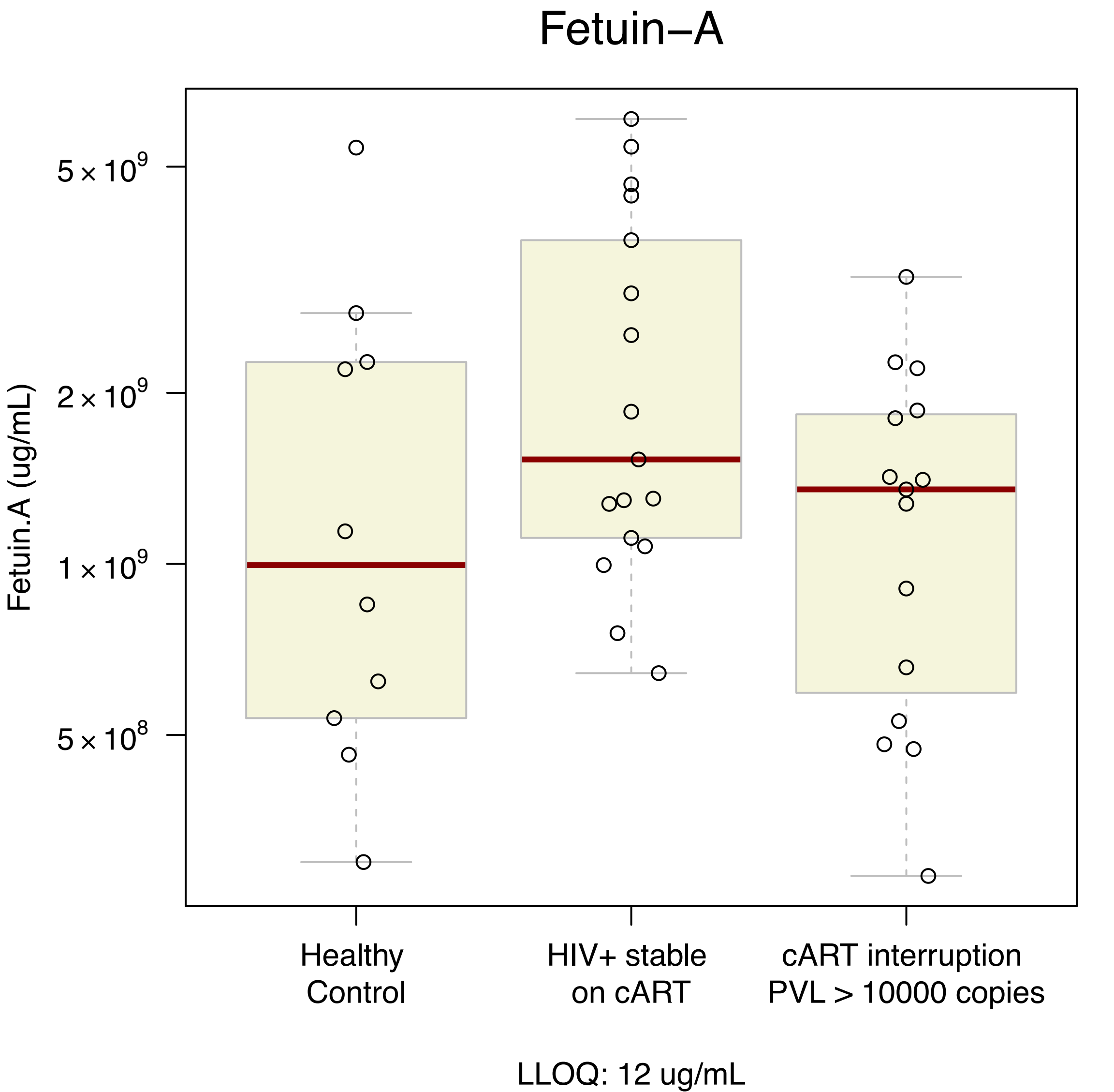
Fetuin–A
Fetuin.A (ug/mL)
5 × 10^9
2 × 10^9
1 × 10^9
5 × 10^8
Healthy Control
HIV+ stable on cART
cART interruption PVL > 10000 copies
LLOQ: 12 ug/mL

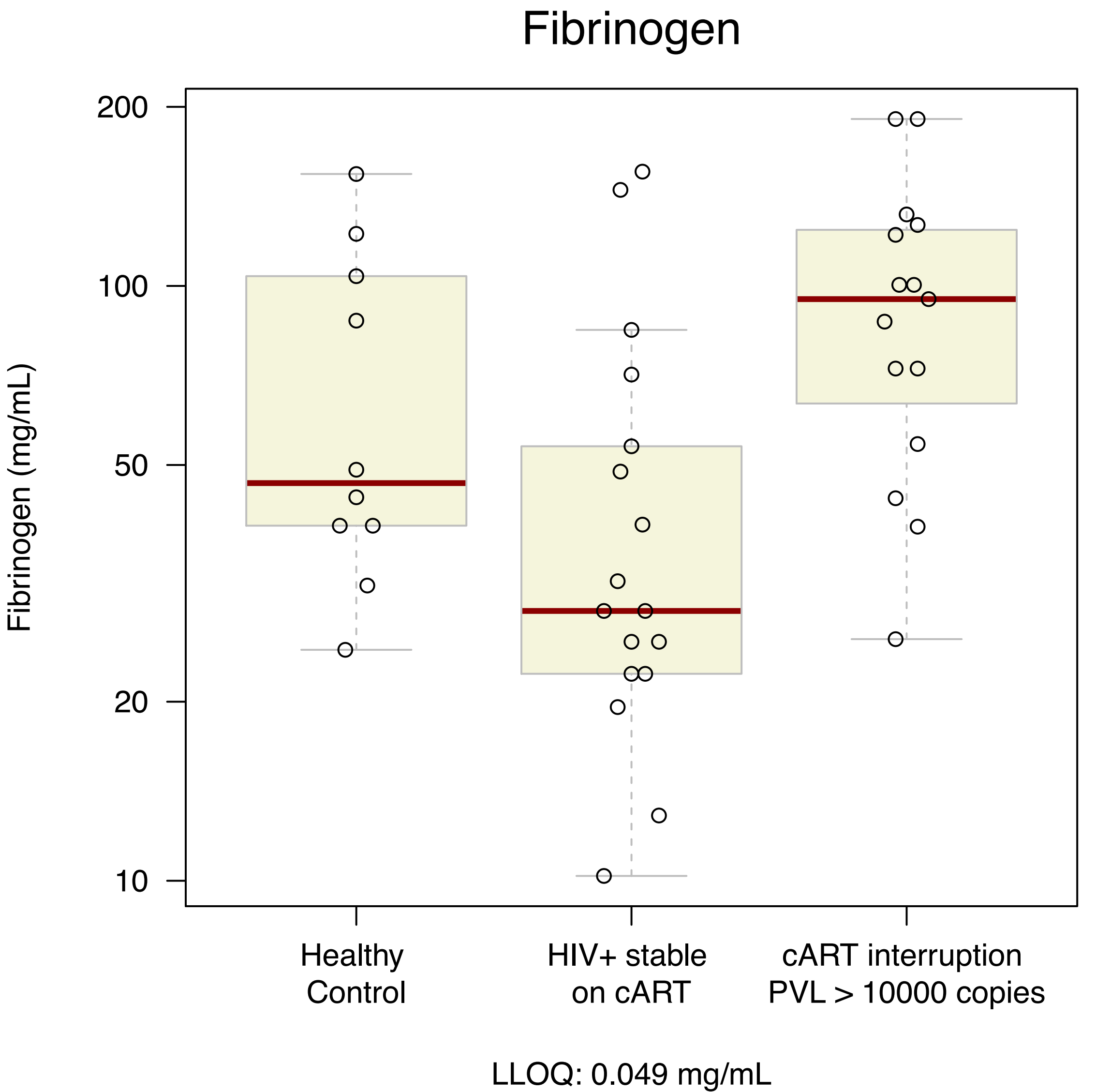
Fibrinogen
Fibrinogen (mg/mL)
200
100
50
20
10
Healthy Control
HIV+ stable on cART
cART interruption PVL > 10000 copies
LLOQ: 0.049 mg/mL

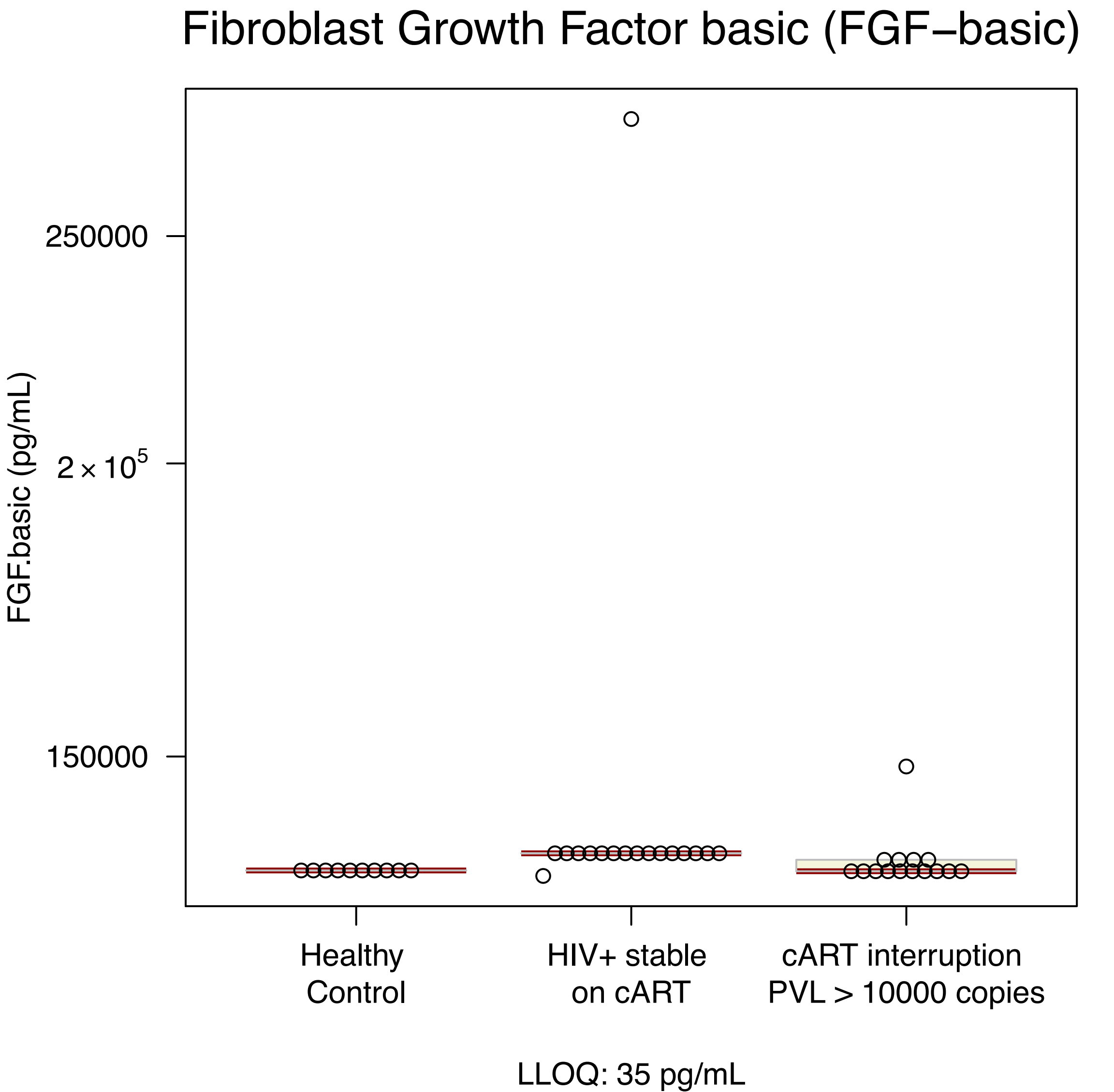

Fibroblast Growth Factor basic (FGF−basic)
FGF.basic (pg/mL)
250000
2×10^5
150000
Healthy Control
HIV+ stable on cART
cART interruption PVL > 10000 copies
LLOQ: 35 pg/mL

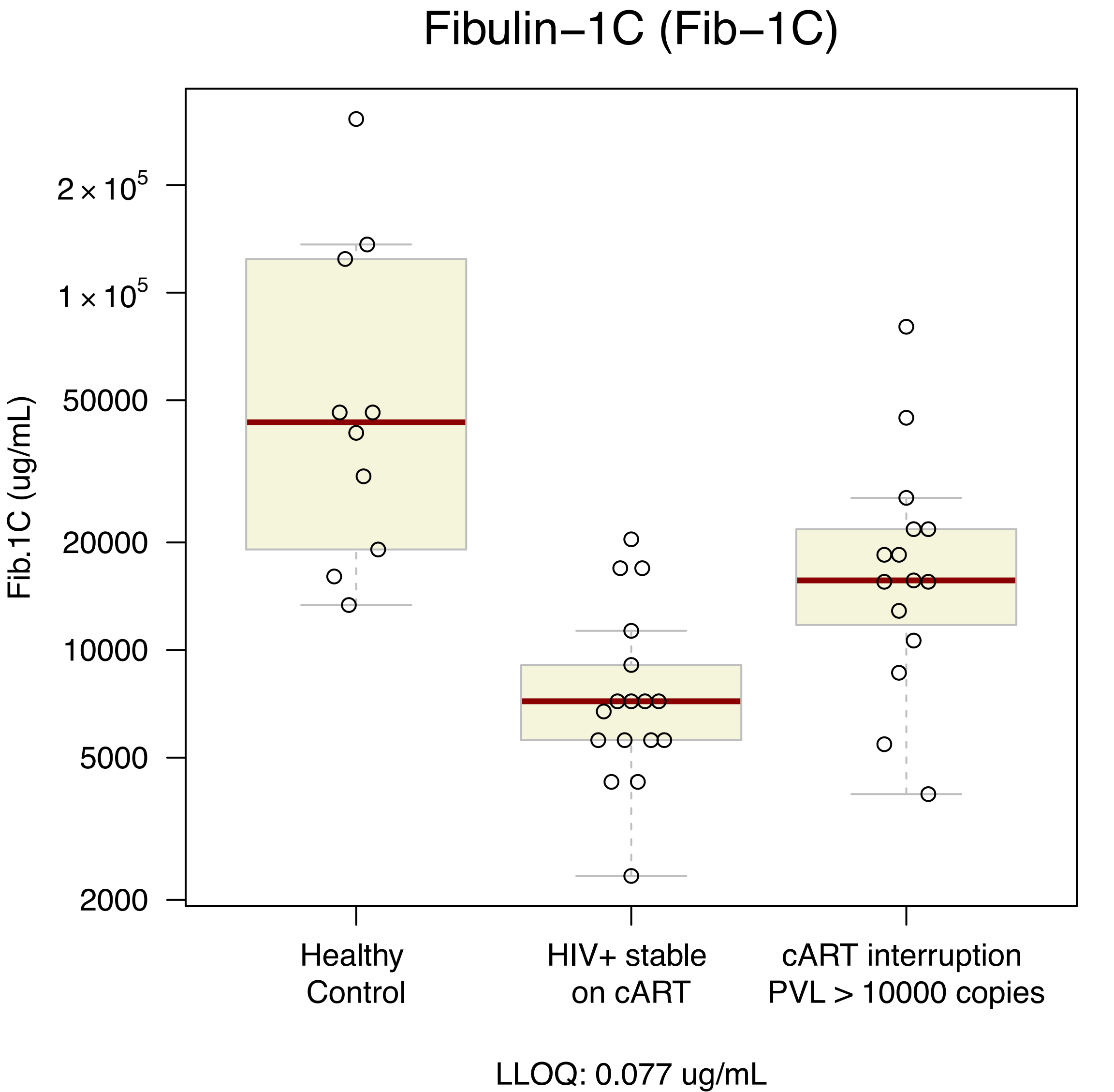

Fibulin−1C (Fib−1C)
Fib.1C (ug/mL)
2×10^5
1×10^5
50000
20000
10000
5000
2000
Healthy Control
HIV+ stable on cART
cART interruption PVL > 10000 copies
LLOQ: 0.077 ug/mL

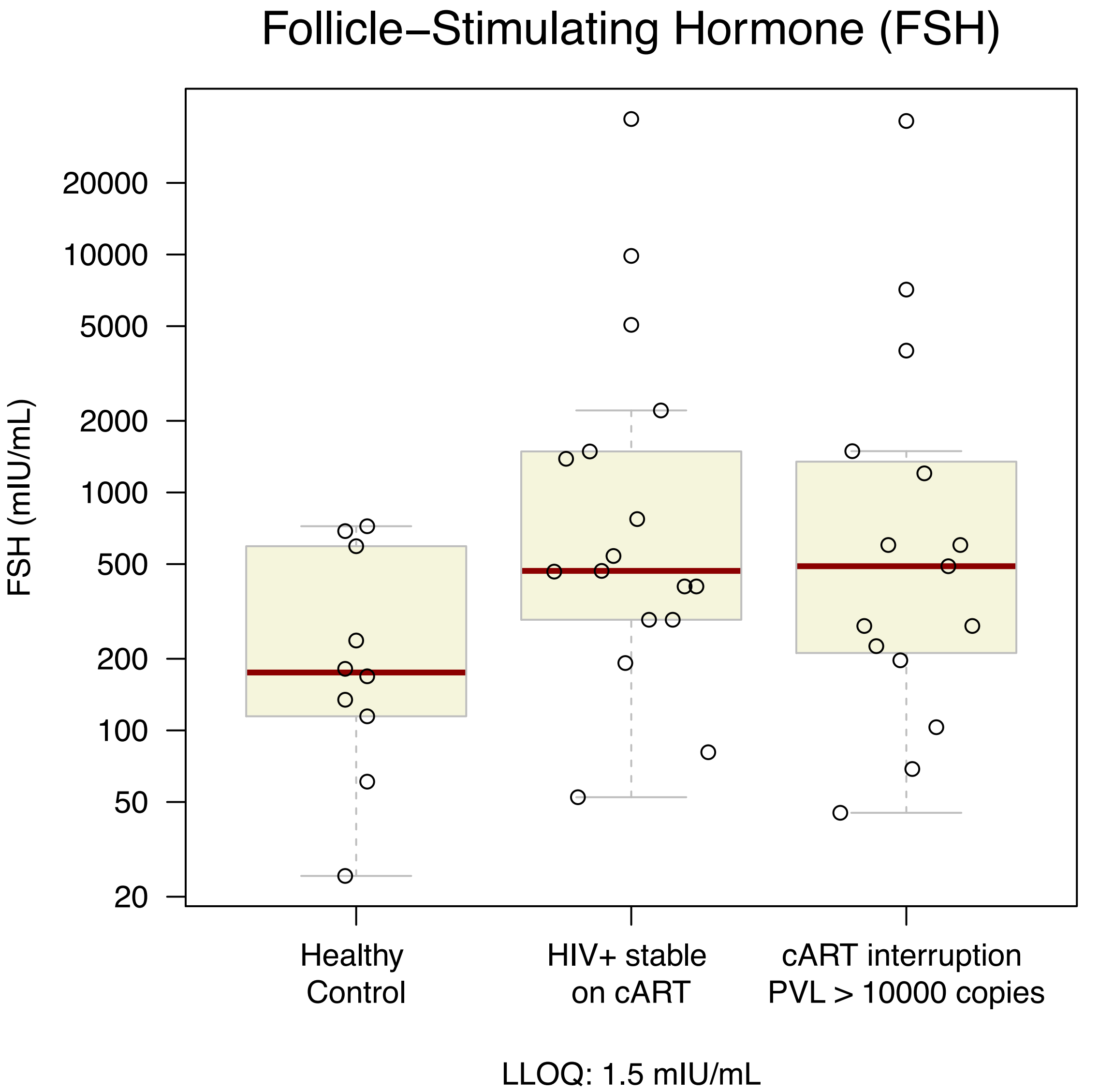

Follicle−Stimulating Hormone (FSH)
FSH (mIU/mL)
20000
10000
5000
2000
1000
500
200
100
50
20
Healthy Control
HIV+ stable on cART
cART interruption PVL > 10000 copies
LLOQ: 1.5 mIU/mL

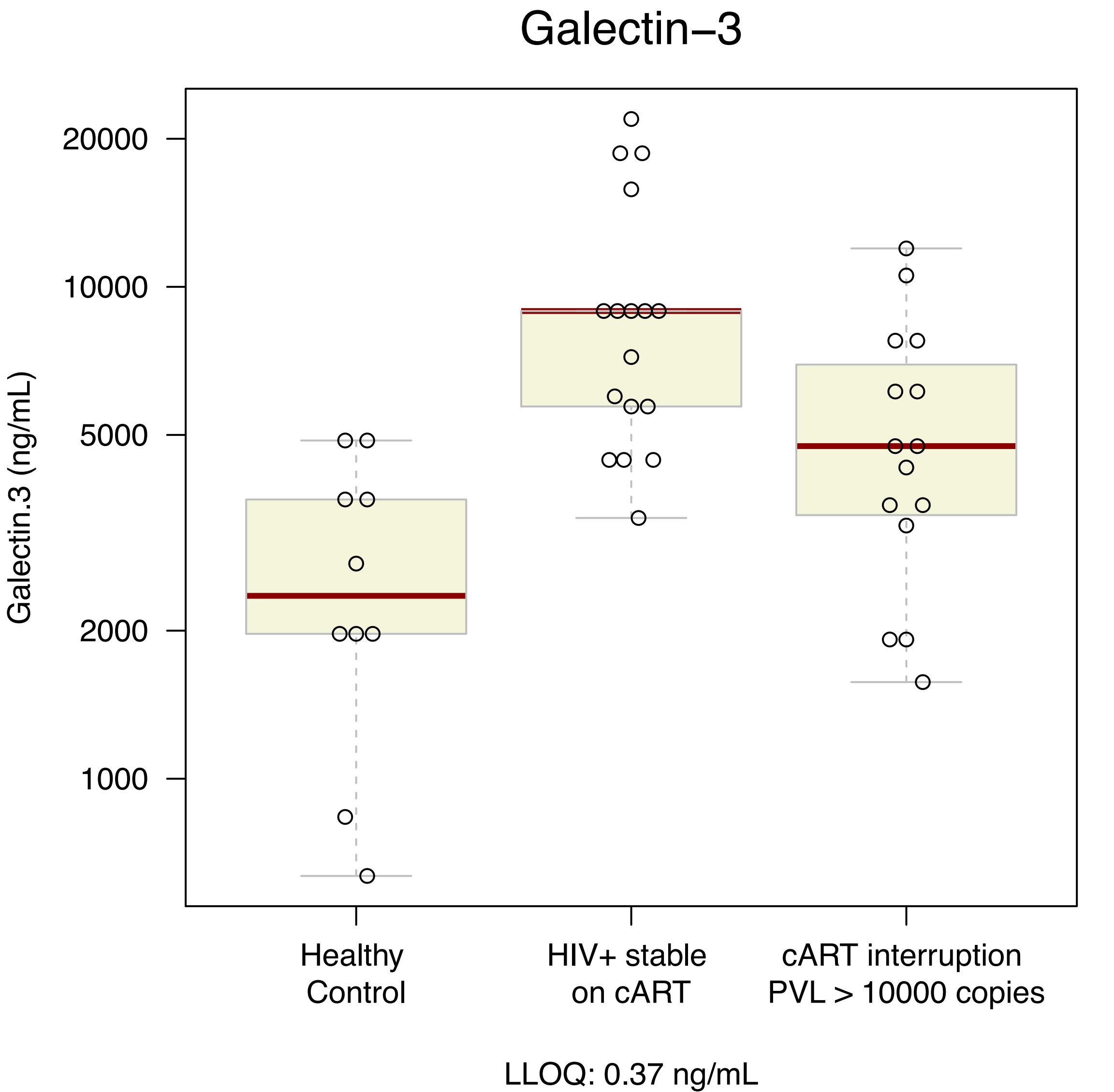

Galectin−3
Galectin.3 (ng/mL)
20000
10000
5000
2000
1000
Healthy Control
HIV+ stable on cART
cART interruption PVL > 10000 copies
LLOQ: 0.37 ng/mL

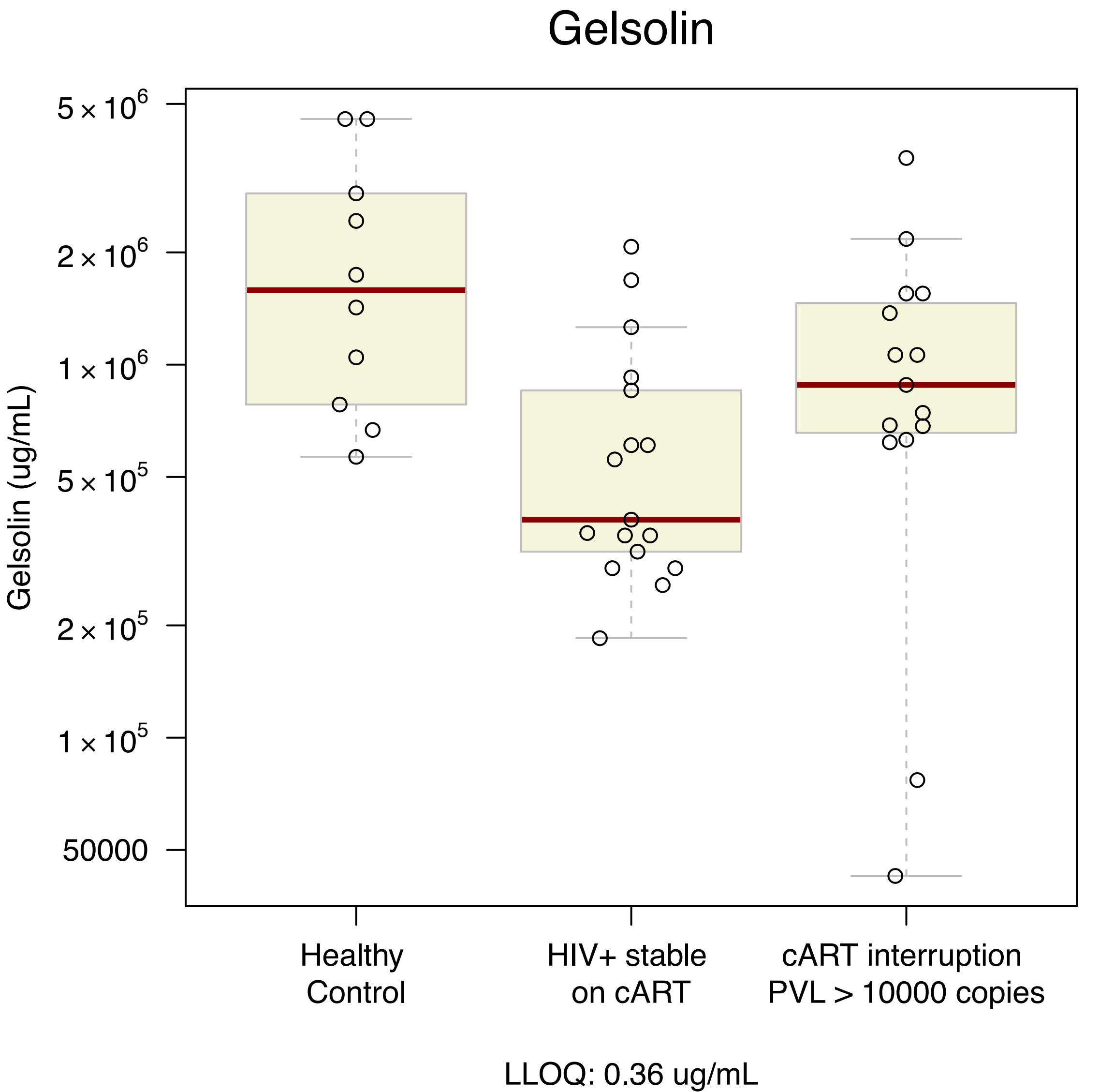

Gelsolin
Gelsolin (ug/mL)
5×10^6
2×10^6
1×10^6
5×10^5
2×10^5
1×10^5
50000
Healthy Control
HIV+ stable on cART
cART interruption PVL > 10000 copies
LLOQ: 0.36 ug/mL

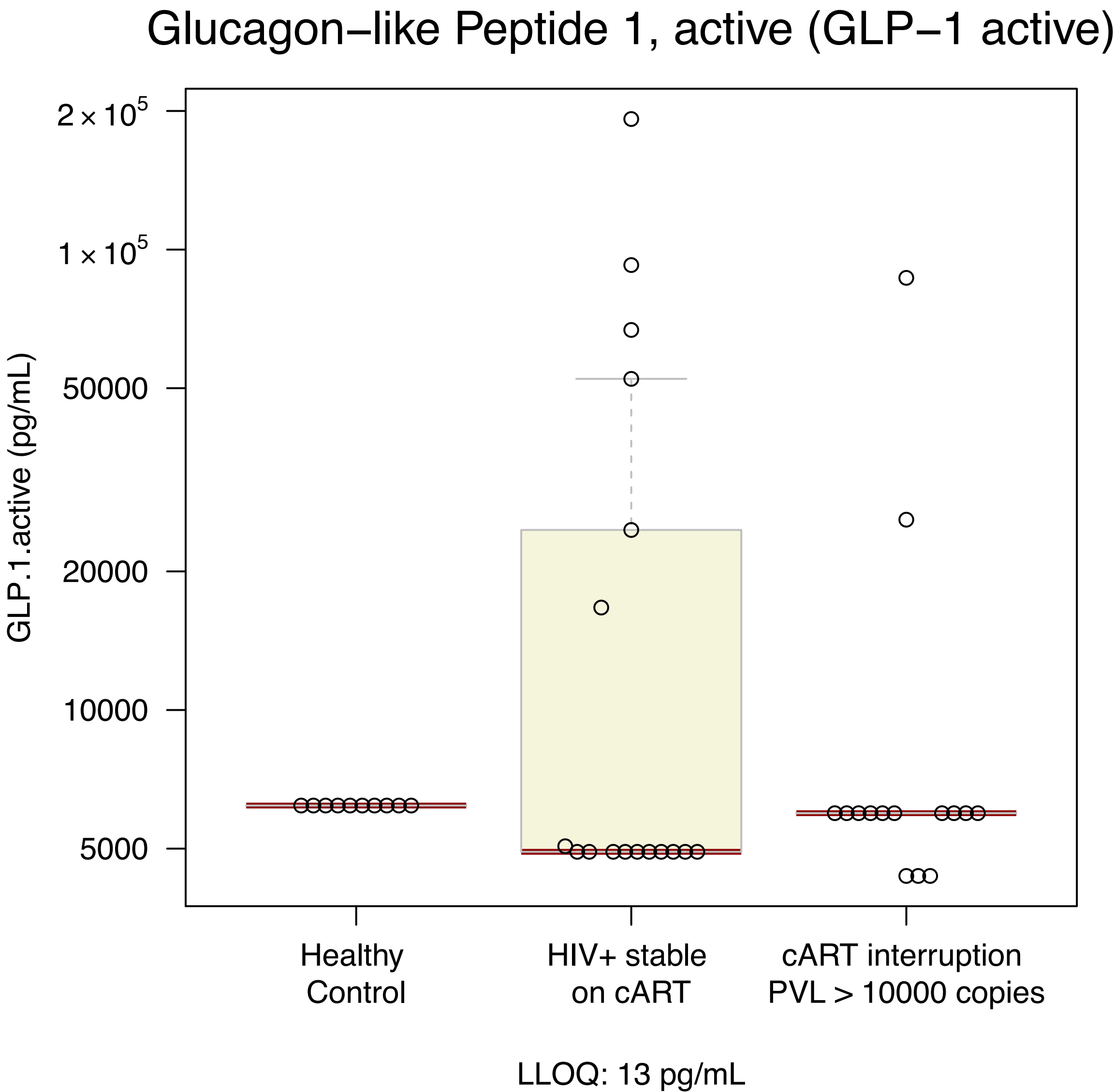

Glucagon−like Peptide 1, active (GLP−1 active)
GLP.1.active (pg/mL)
2×10^5
1×10^5
50000
20000
10000
5000
Healthy Control
HIV+ stable on cART
cART interruption PVL > 10000 copies
LLOQ: 13 pg/mL

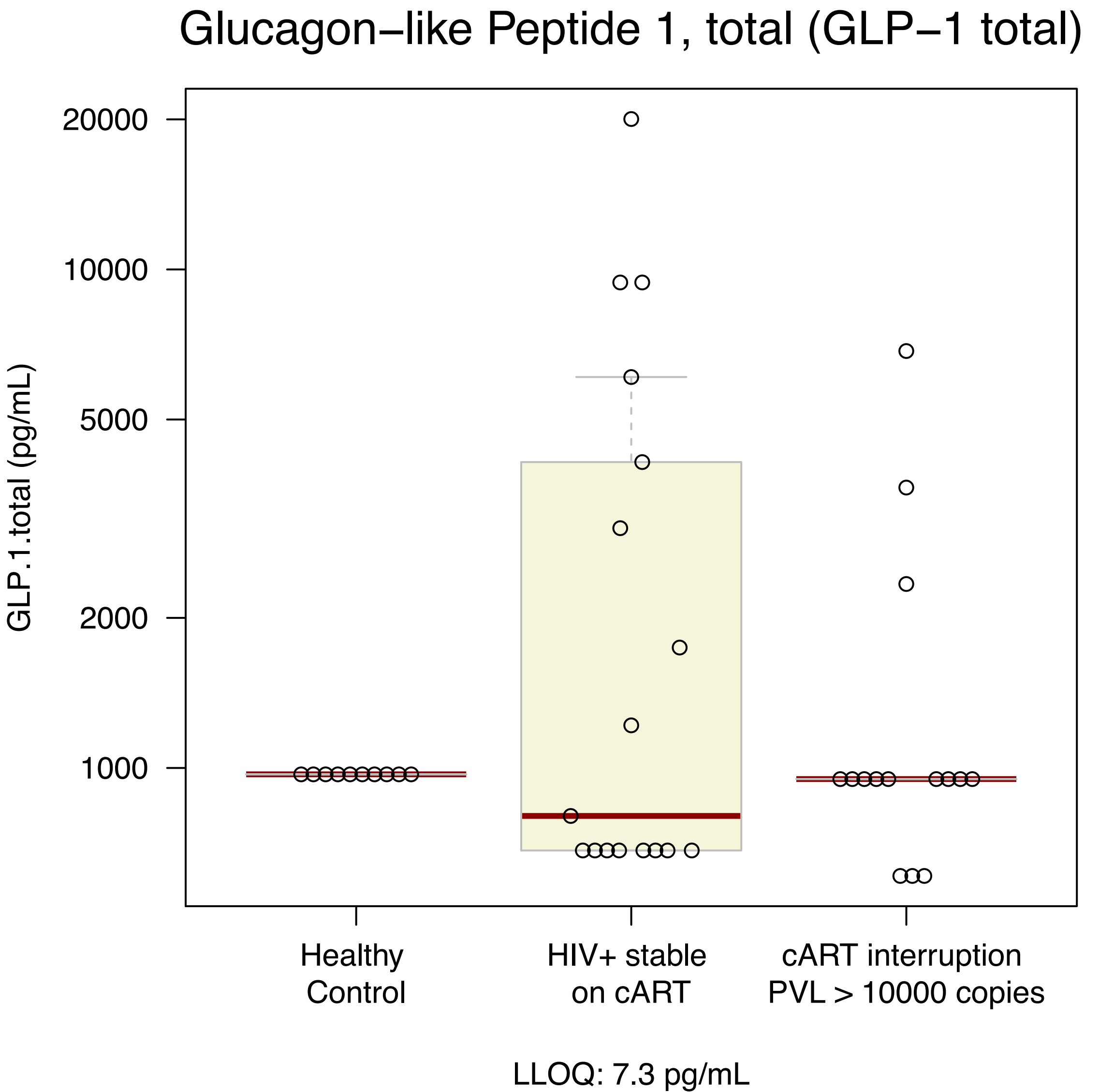

Glucagon−like Peptide 1, total (GLP−1 total)
GLP.1.total (pg/mL)
20000
10000
5000
2000
1000
Healthy Control
HIV+ stable on cART
cART interruption PVL > 10000 copies
LLOQ: 7.3 pg/mL

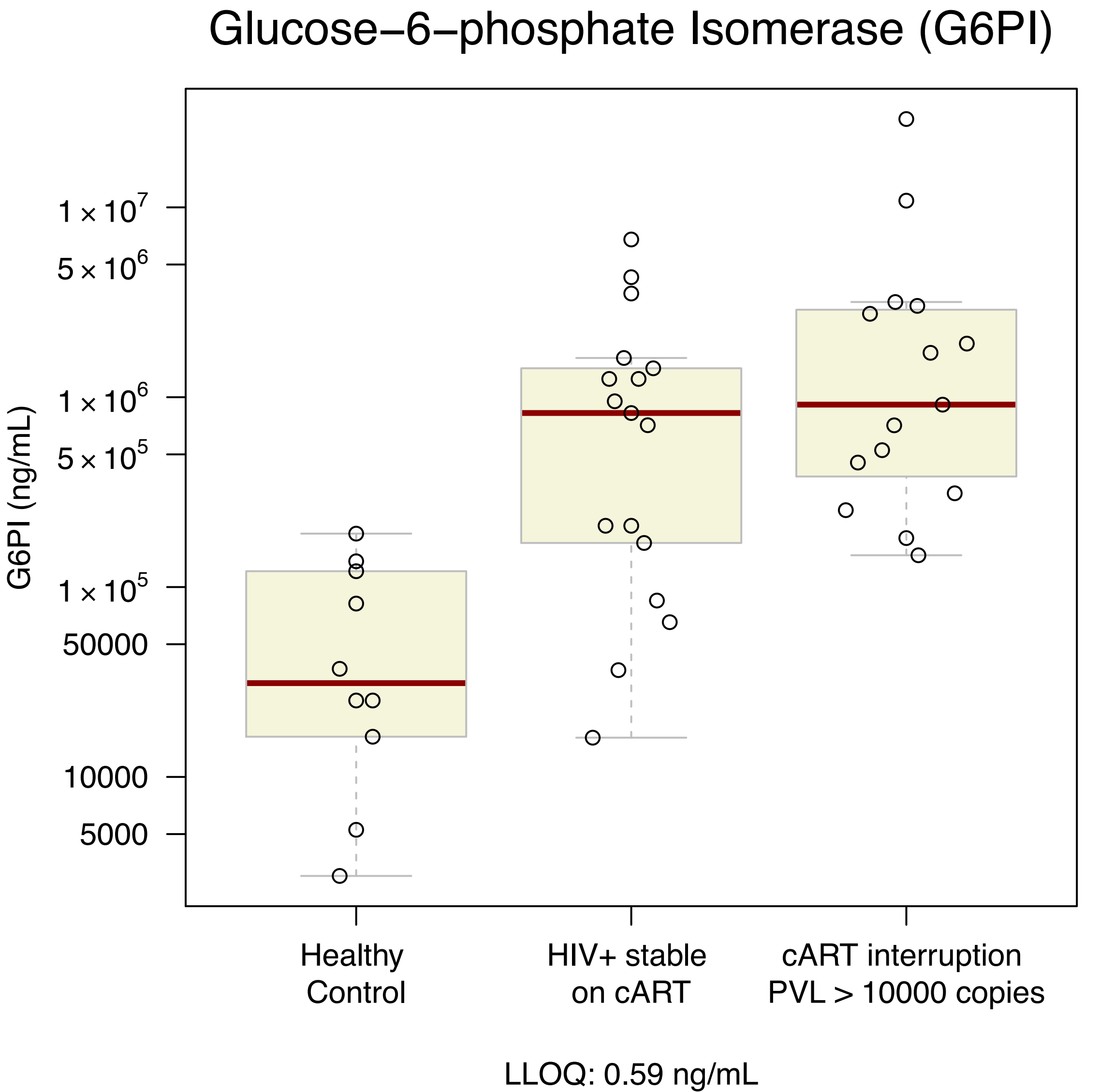
Glucose−6−phosphate Isomerase (G6PI)
G6PI (ng/mL)
1 × 10^7
5 × 10^6
1 × 10^6
5 × 10^5
1 × 10^5
50000
10000
5000
Healthy Control
HIV+ stable on cART
cART interruption PVL > 10000 copies
LLOQ: 0.59 ng/mL

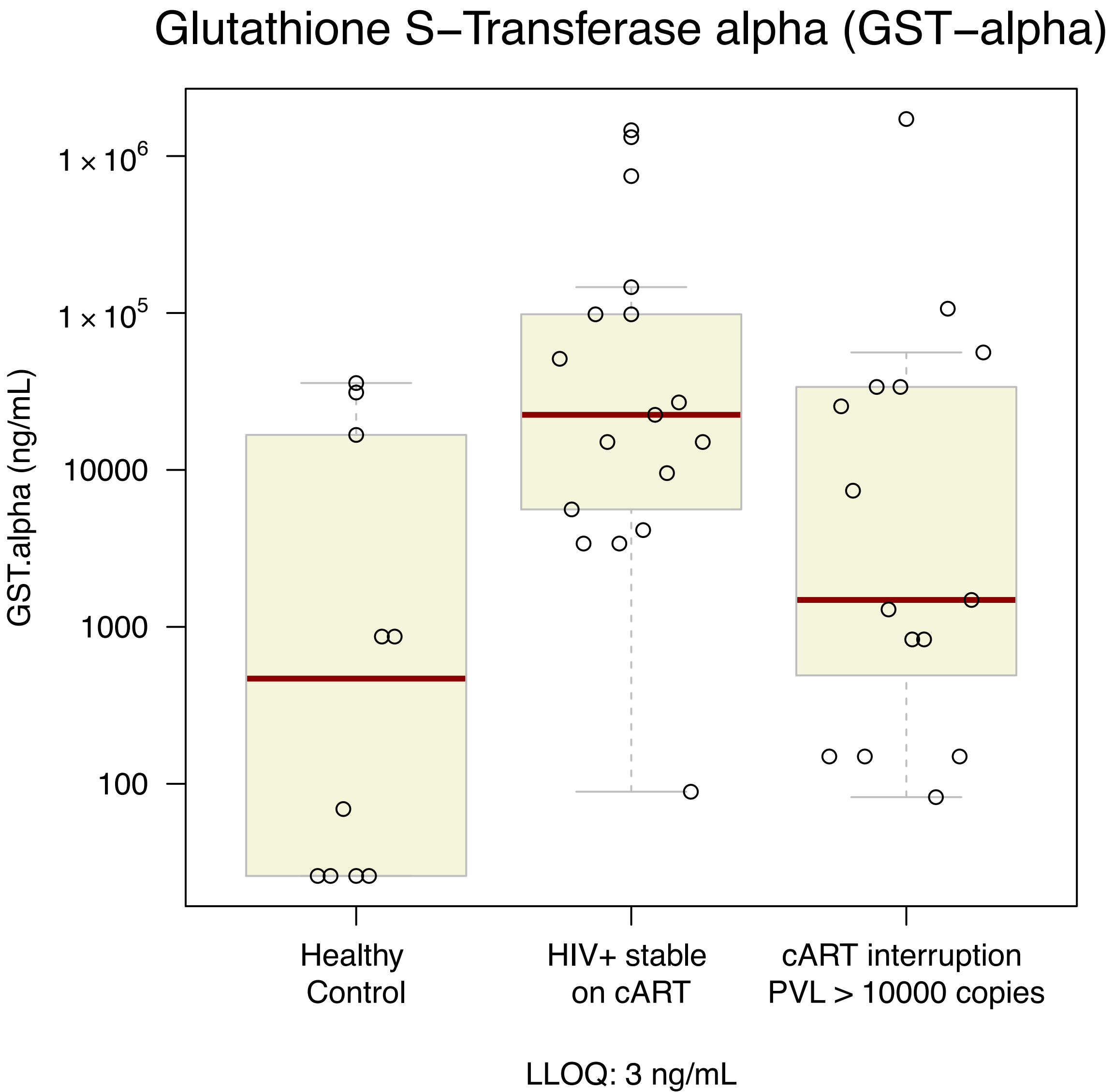

Glutathione S−Transferase alpha (GST−alpha)
GST.alpha (ng/mL)
1×10^6
1×10^5
10000
1000
100
Healthy Control
HIV+ stable on cART
cART interruption PVL > 10000 copies
LLOQ: 3 ng/mL

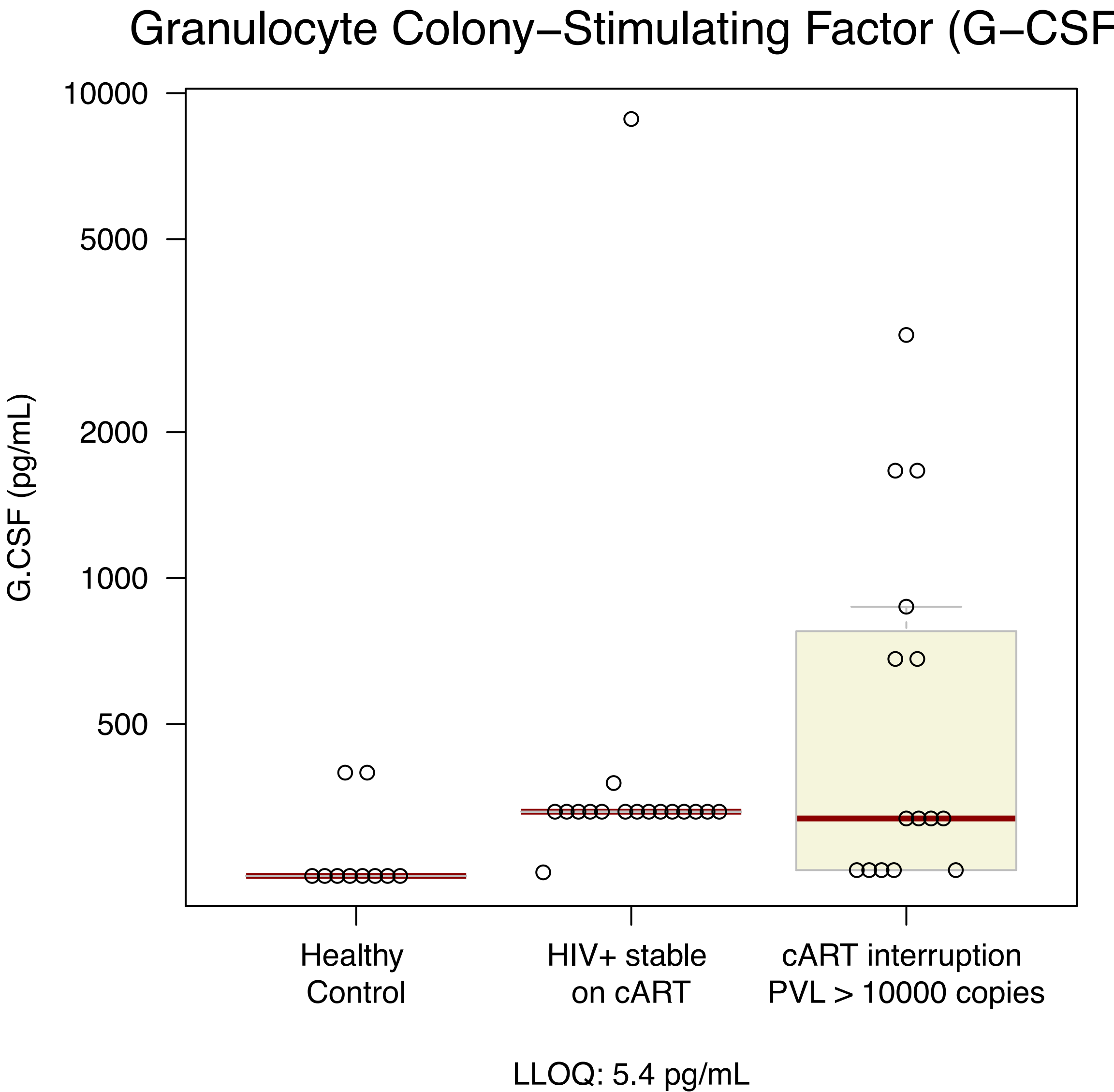
Granulocyte Colony−Stimulating Factor (G−CSF
G.CSF (pg/mL)
10000
5000
2000
1000
500
Healthy Control
HIV+ stable on cART
cART interruption PVL > 10000 copies
LLOQ: 5.4 pg/mL

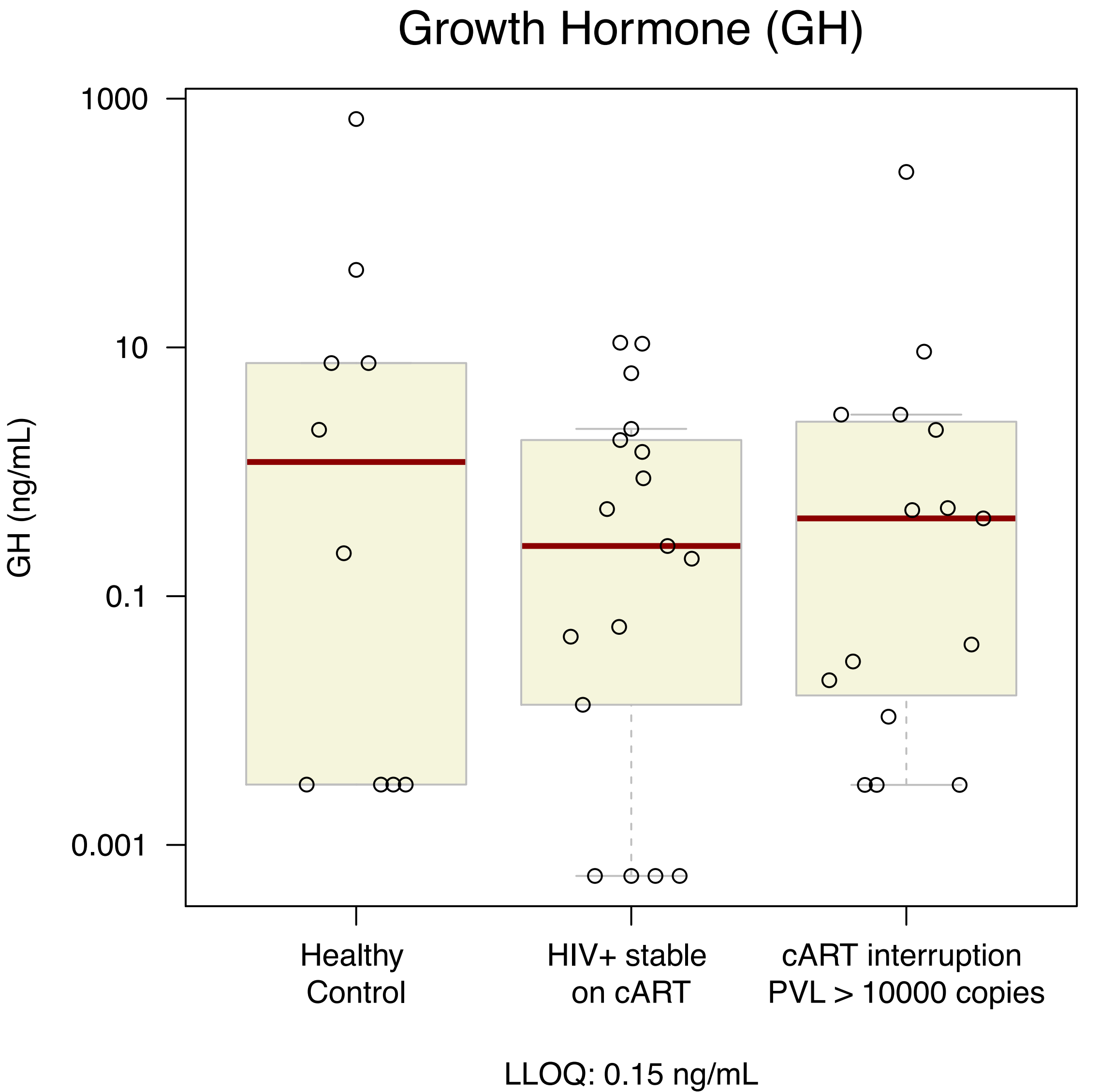

Growth Hormone (GH)
1000
10
0.1
0.001
GH (ng/mL)
Healthy Control
HIV+ stable on cART
cART interruption PVL > 10000 copies
LLOQ: 0.15 ng/mL

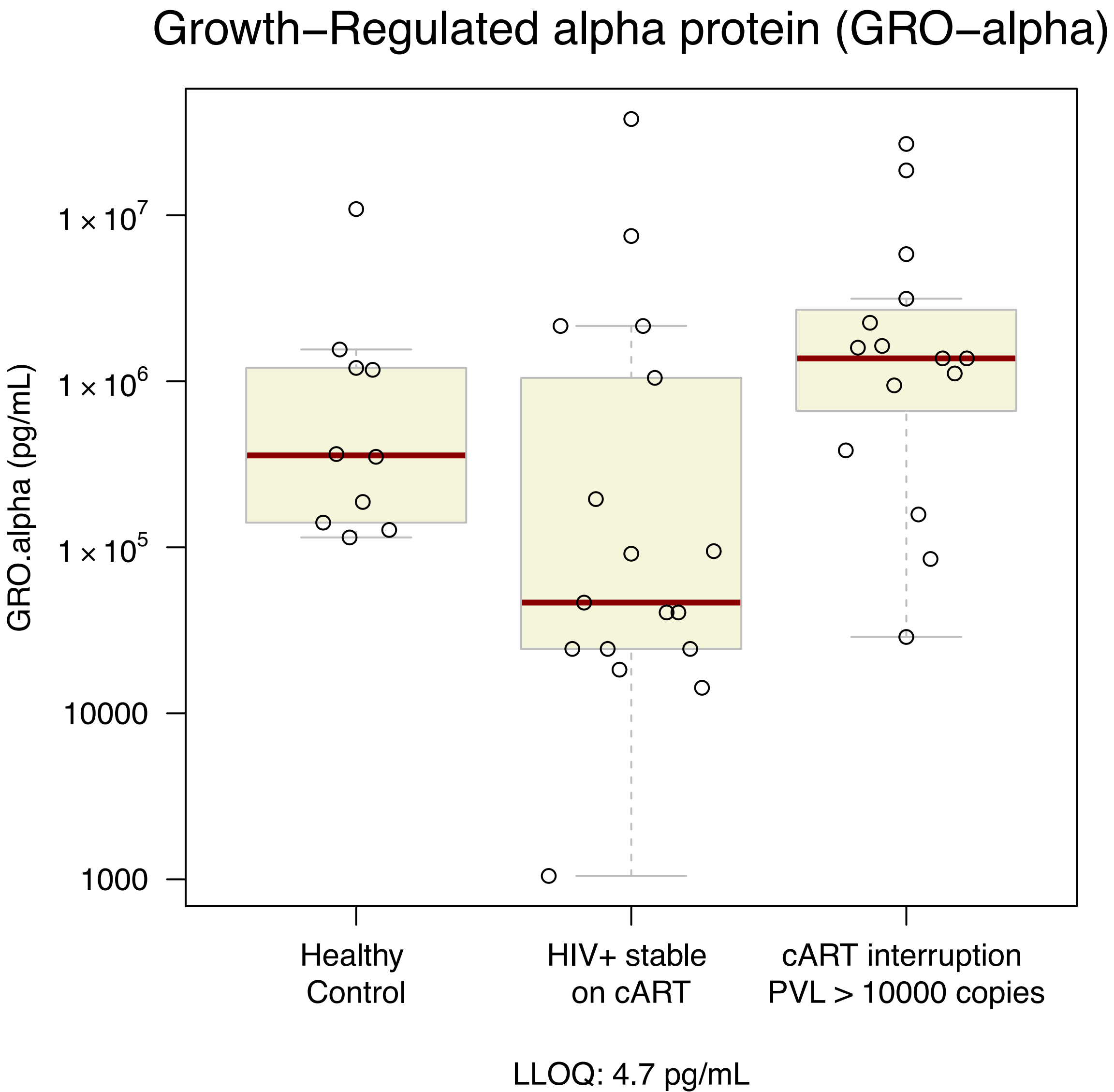
Growth−Regulated alpha protein (GRO−alpha)
GRO.alpha (pg/mL)
1 × 10^7
1 × 10^6
1 × 10^5
10000
1000
Healthy Control
HIV+ stable on cART
cART interruption PVL > 10000 copies
LLOQ: 4.7 pg/mL

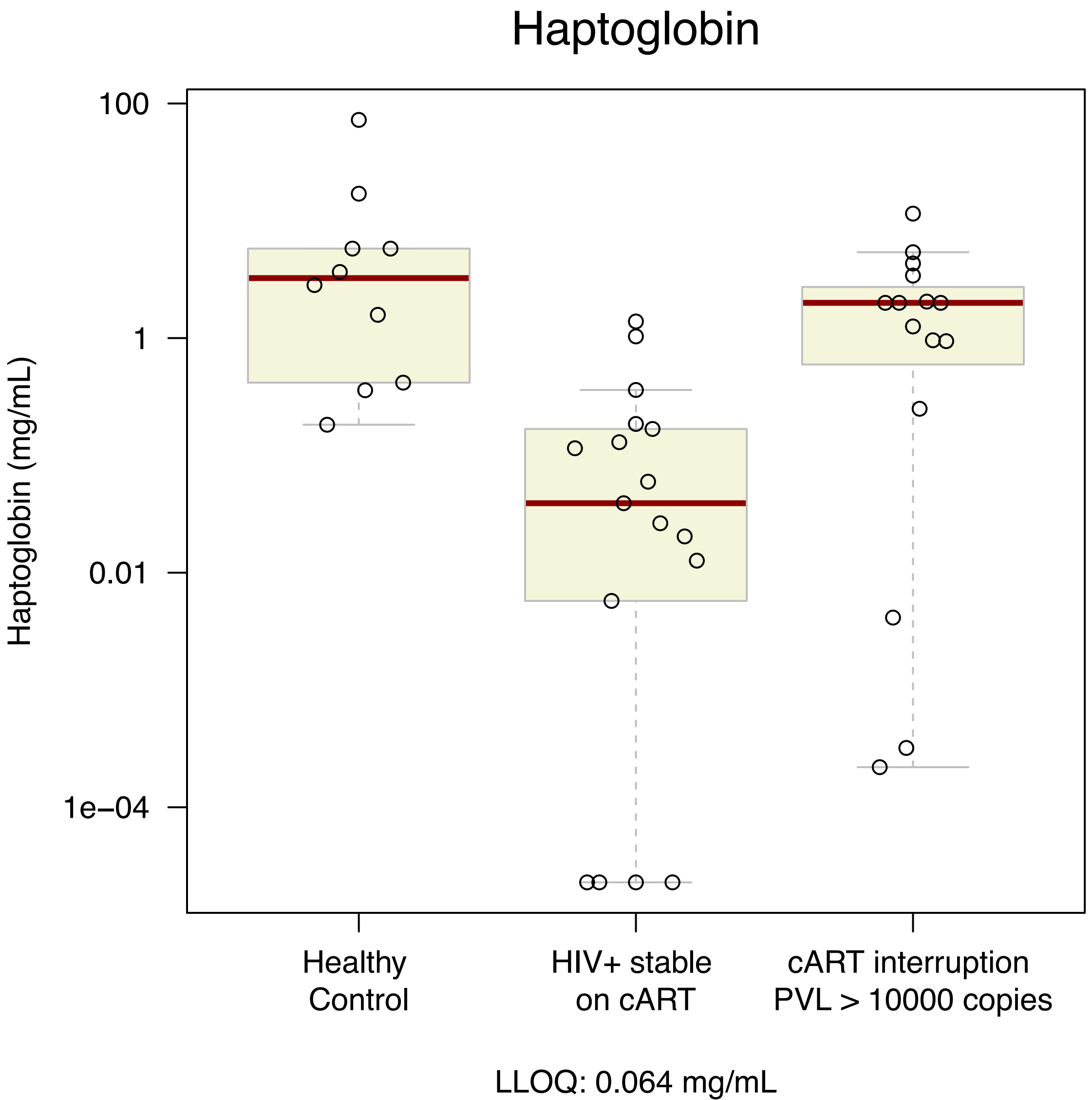
Haptoglobin
Haptoglobin (mg/mL)
100
1
0.01
1e−04
Healthy Control
HIV+ stable on cART
cART interruption PVL > 10000 copies
LLOQ: 0.064 mg/mL

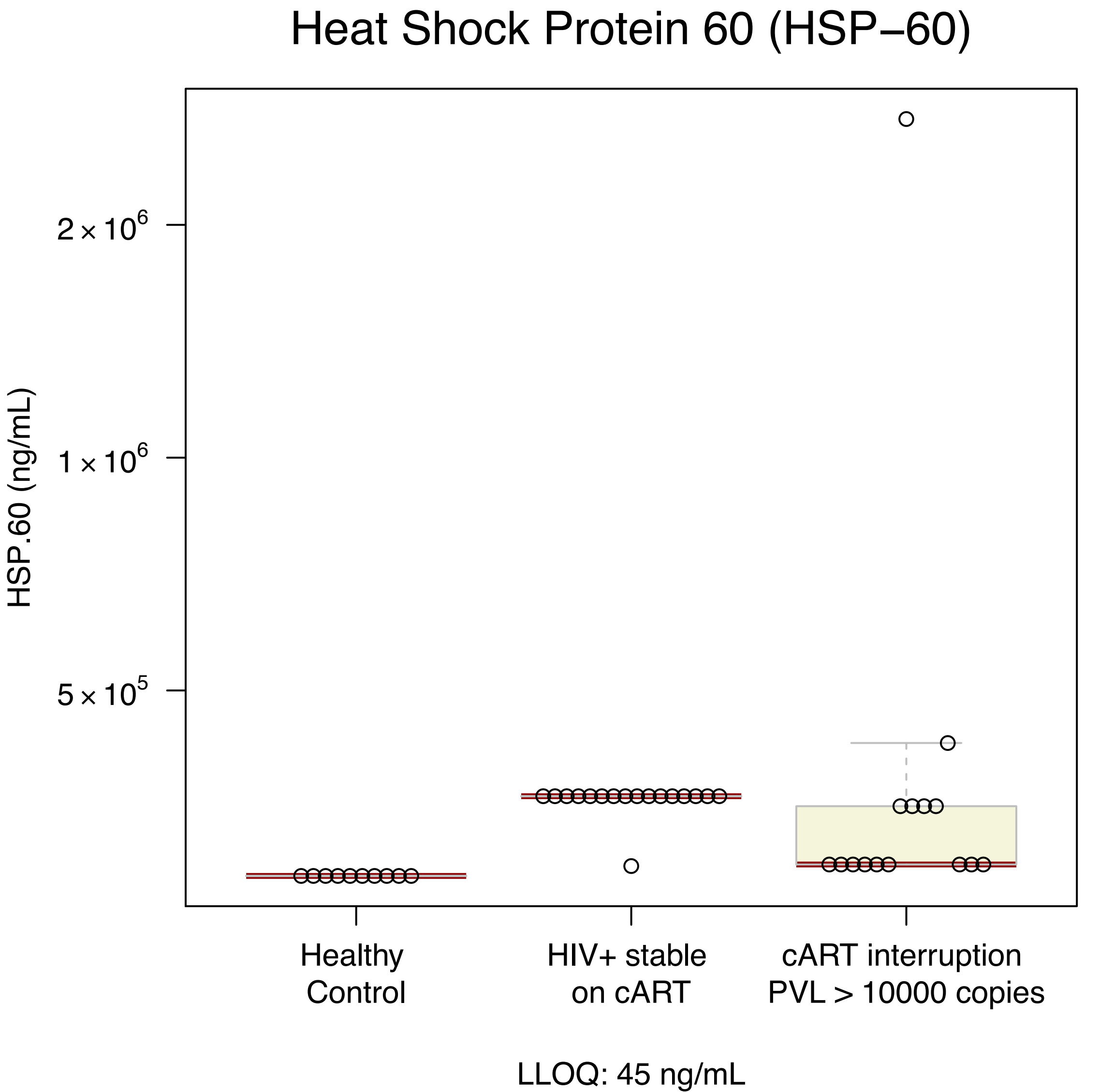

Heat Shock Protein 60 (HSP−60)
HSP.60 (ng/mL)
2×10^6
1×10^6
5×10^5
Healthy Control
HIV+ stable on cART
cART interruption PVL > 10000 copies
LLOQ: 45 ng/mL

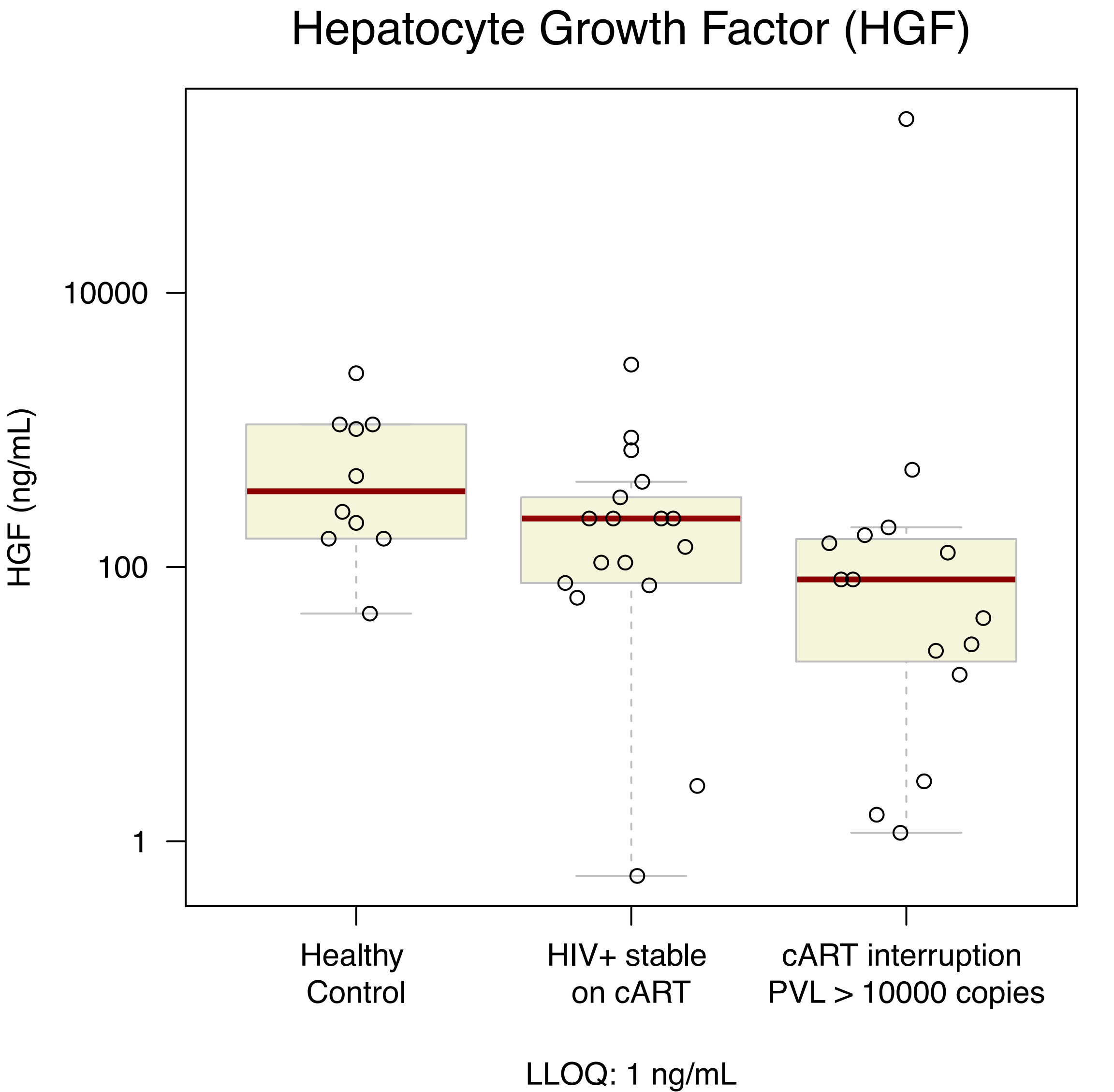
Hepatocyte Growth Factor (HGF)
HGF (ng/mL)
10000
100
1
Healthy Control
HIV+ stable on cART
cART interruption PVL > 10000 copies
LLOQ: 1 ng/mL

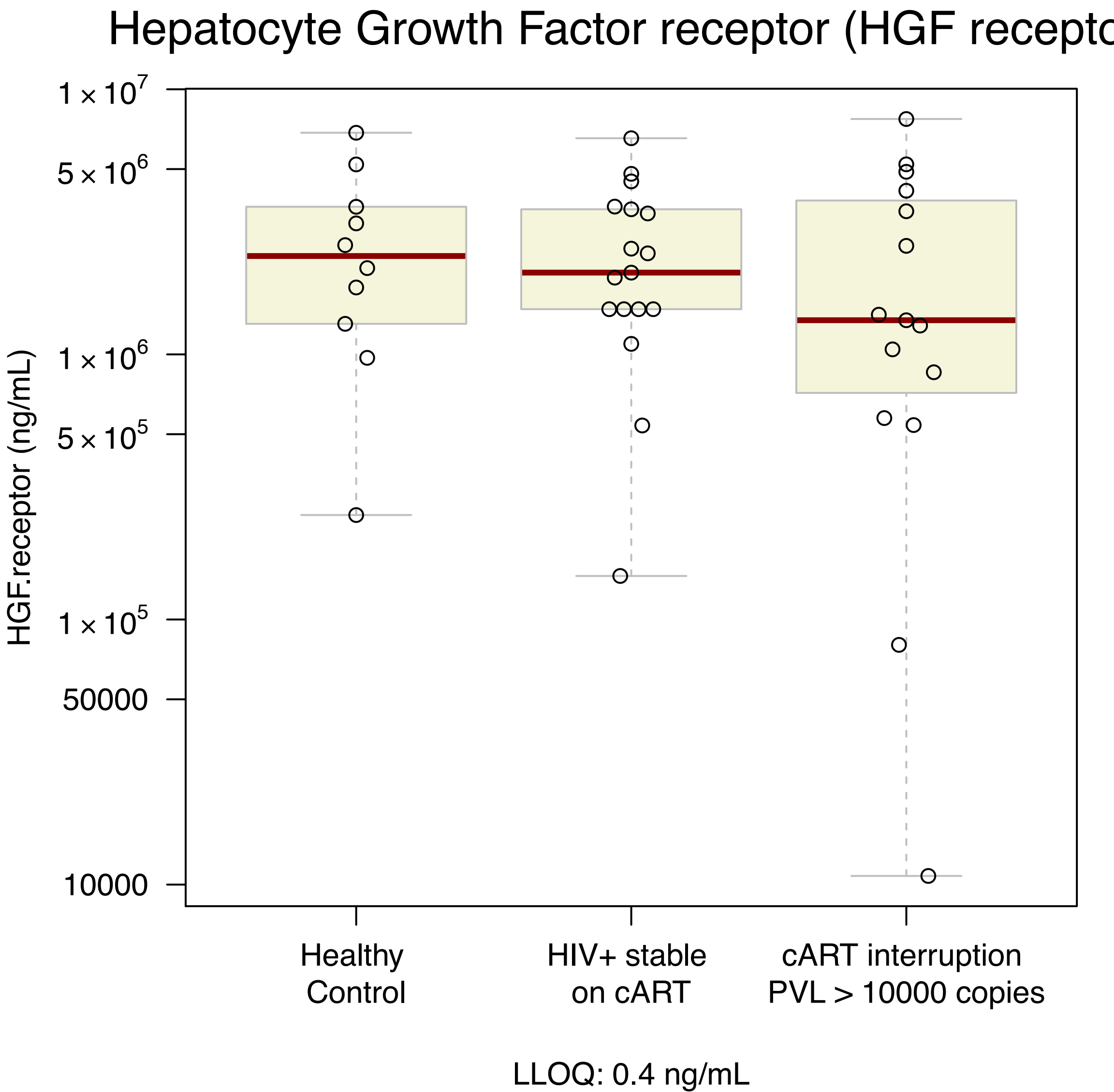
Hepatocyte Growth Factor receptor (HGF recepto
HGF.receptor (ng/mL)
1 × 10^7
5 × 10^6
1 × 10^6
5 × 10^5
1 × 10^5
50000
10000
Healthy Control
HIV+ stable on cART
cART interruption PVL > 10000 copies
LLOQ: 0.4 ng/mL

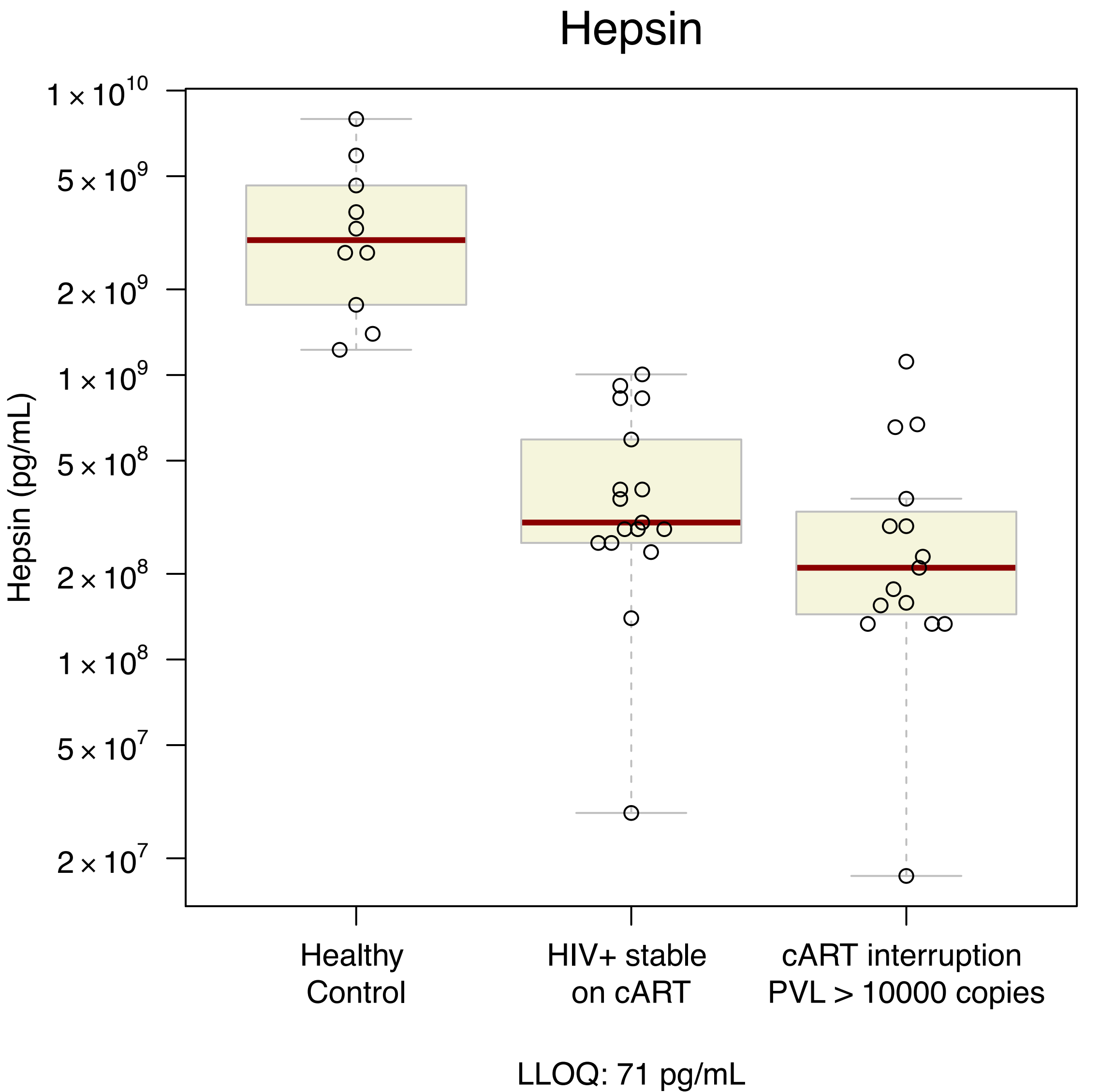
Hepsin
Hepsin (pg/mL)
1 × 10^10
5 × 10^9
2 × 10^9
1 × 10^9
5 × 10^8
2 × 10^8
1 × 10^8
5 × 10^7
2 × 10^7
Healthy Control
HIV+ stable on cART
cART interruption PVL > 10000 copies
LLOQ: 71 pg/mL

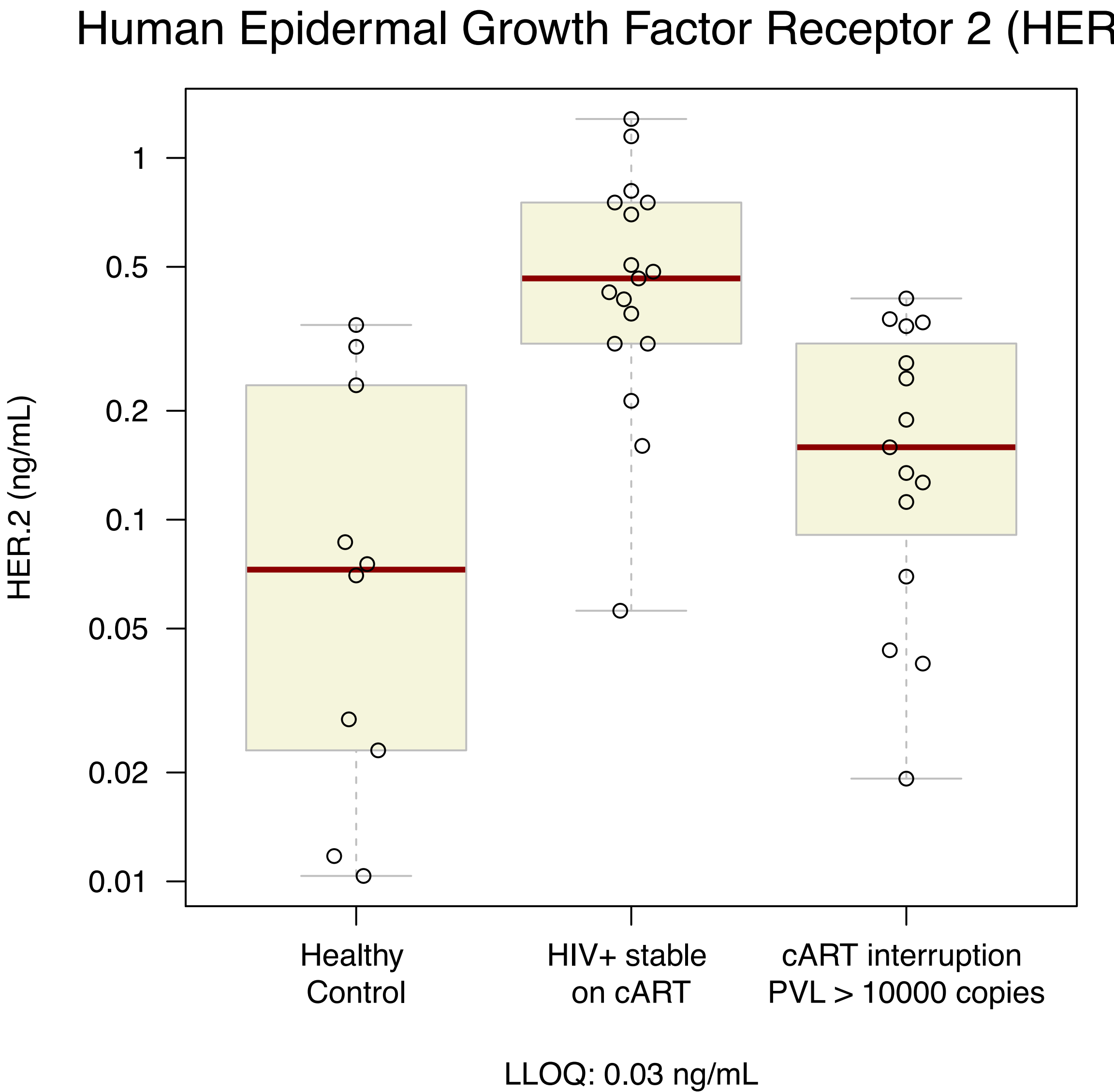
Human Epidermal Growth Factor Receptor 2 (HER
HER.2 (ng/mL)
1
0.5
0.2
0.1
0.05
0.02
0.01
Healthy Control
HIV+ stable on cART
cART interruption PVL > 10000 copies
LLOQ: 0.03 ng/mL

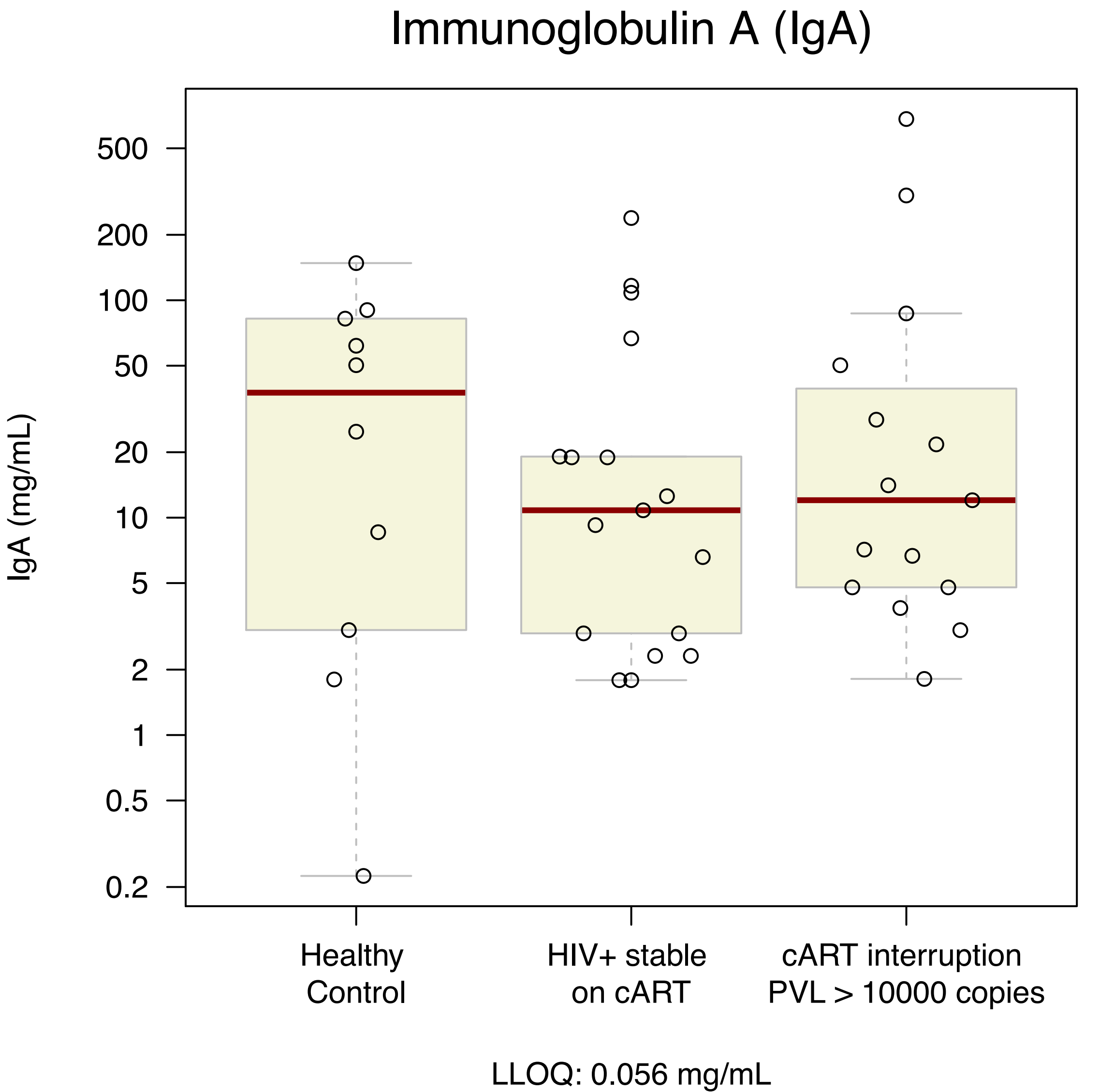

Immunoglobulin A (IgA)
IgA (mg/mL)
500
200
100
50
20
10
5
2
1
0.5
0.2
Healthy Control
HIV+ stable on cART
cART interruption PVL > 10000 copies
LLOQ: 0.056 mg/mL

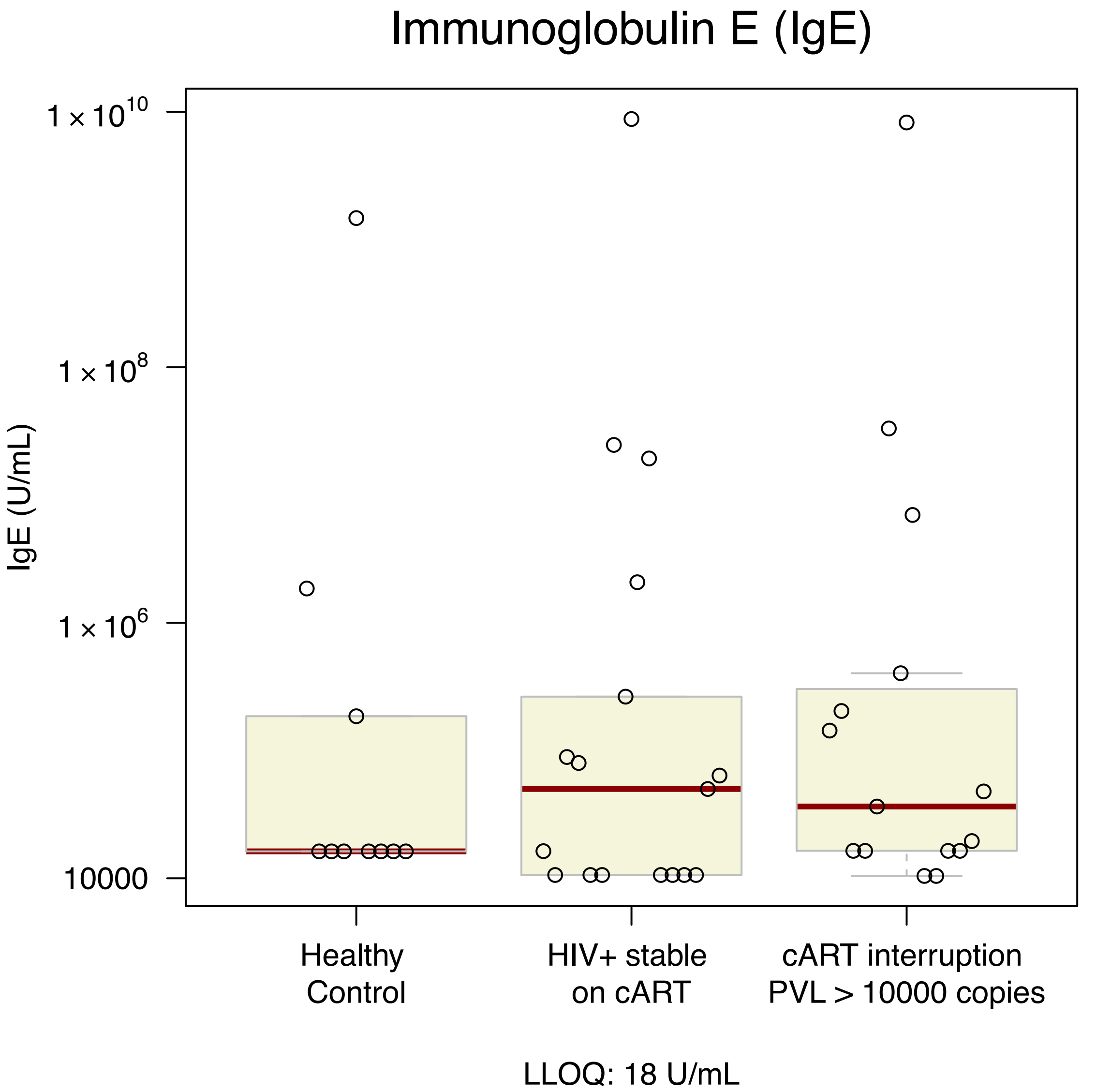
Immunoglobulin E (IgE)
IgE (U/mL)
1×10^{10}
1×10^{8}
1×10^{6}
10000
Healthy Control
HIV+ stable on cART
cART interruption PVL > 10000 copies
LLOQ: 18 U/mL

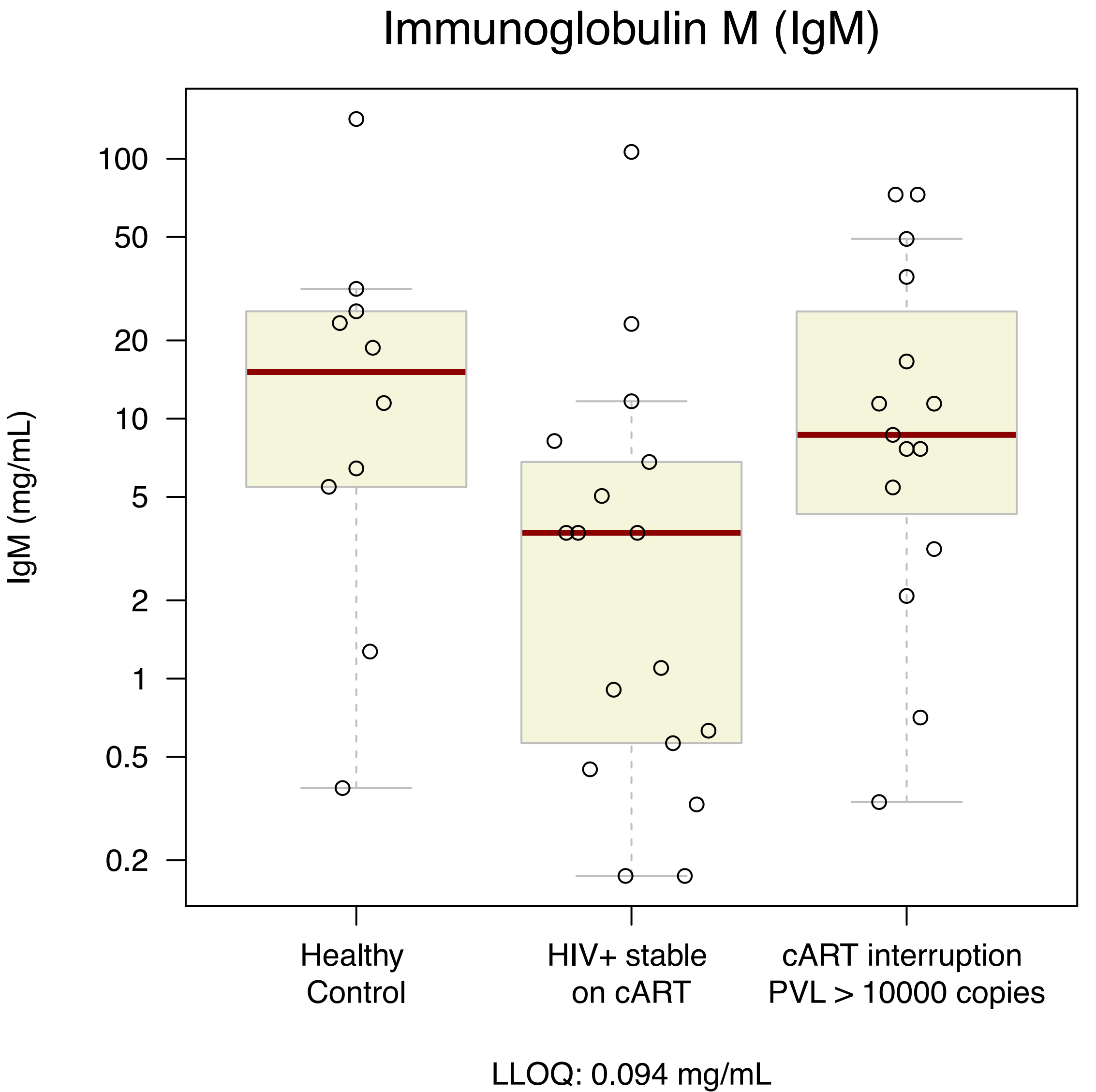

Immunoglobulin M (IgM)
IgM (mg/mL)
100
50
20
10
5
2
1
0.5
0.2
Healthy Control
HIV+ stable on cART
cART interruption PVL > 10000 copies
LLOQ: 0.094 mg/mL

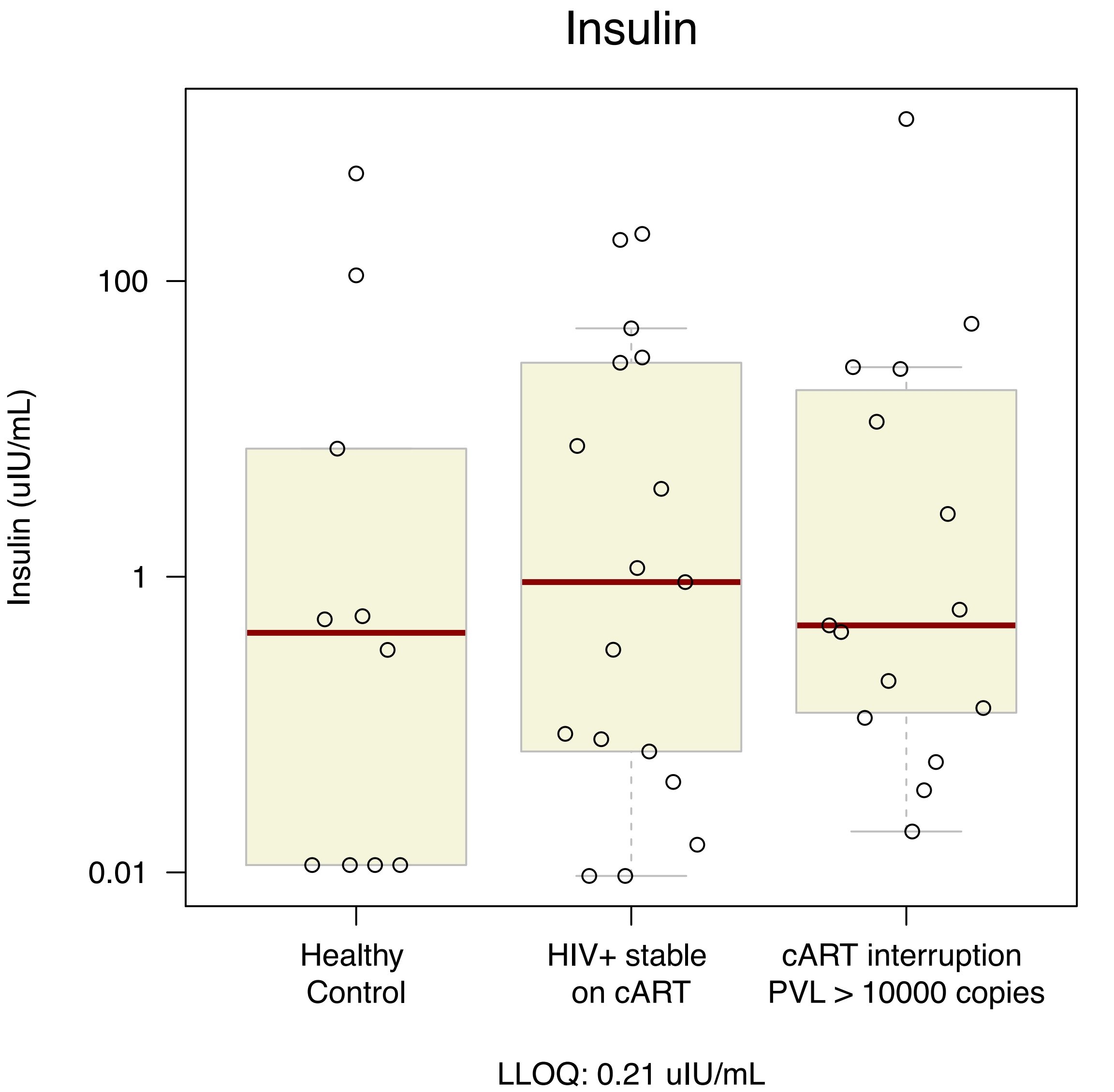

Insulin
Insulin (uIU/mL)
100
1
0.01
Healthy Control
HIV+ stable on cART
cART interruption PVL > 10000 copies
LLOQ: 0.21 uIU/mL

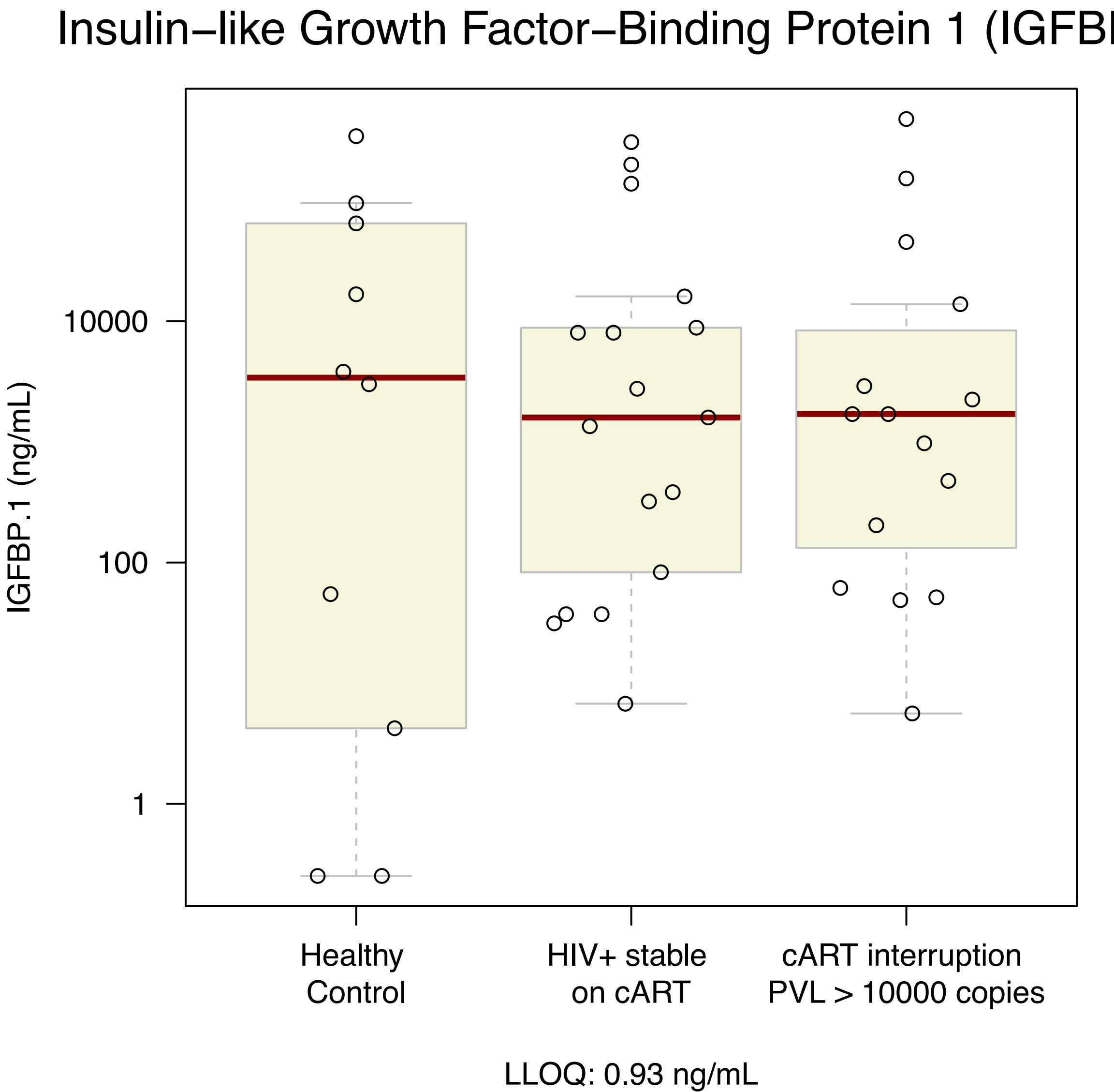
Insulin–like Growth Factor–Binding Protein 1 (IGFBP
IGFBP.1 (ng/mL)
10000
100
1
Healthy Control
HIV+ stable on cART
cART interruption PVL > 10000 copies
LLOQ: 0.93 ng/mL

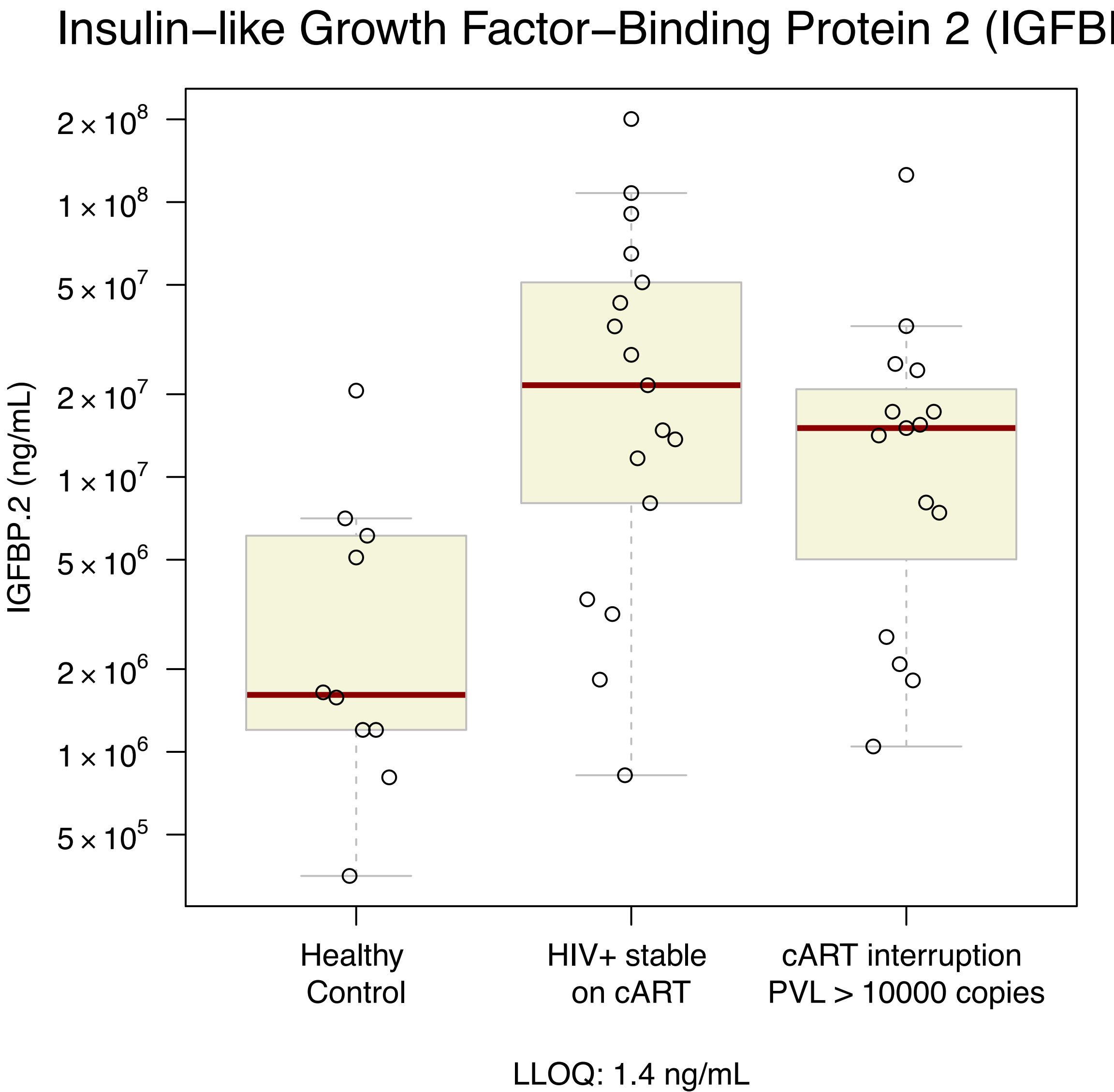

Insulin−like Growth Factor−Binding Protein 2 (IGFBI
IGFBP.2 (ng/mL)
2 × 10^8
1 × 10^8
5 × 10^7
2 × 10^7
1 × 10^7
5 × 10^6
2 × 10^6
1 × 10^6
5 × 10^5
Healthy Control
HIV+ stable on cART
cART interruption PVL > 10000 copies
LLOQ: 1.4 ng/mL

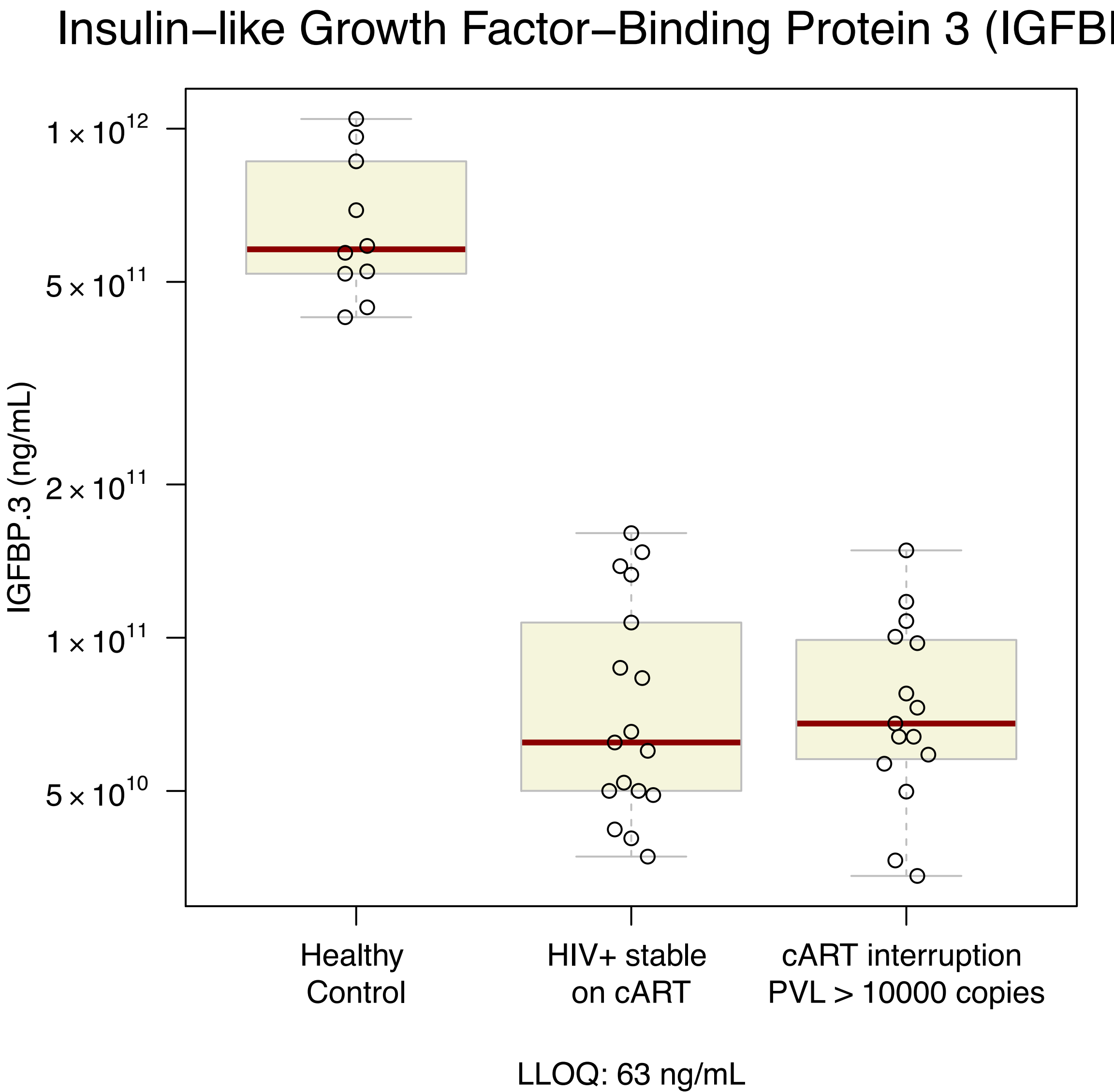
Insulin–like Growth Factor–Binding Protein 3 (IGFBI
IGFBP.3 (ng/mL)
1 × 10^12
5 × 10^11
2 × 10^11
1 × 10^11
5 × 10^10
Healthy Control
HIV+ stable on cART
cART interruption PVL > 10000 copies
LLOQ: 63 ng/mL

Insulin–like Growth Factor Binding Protein 4 (IGFB

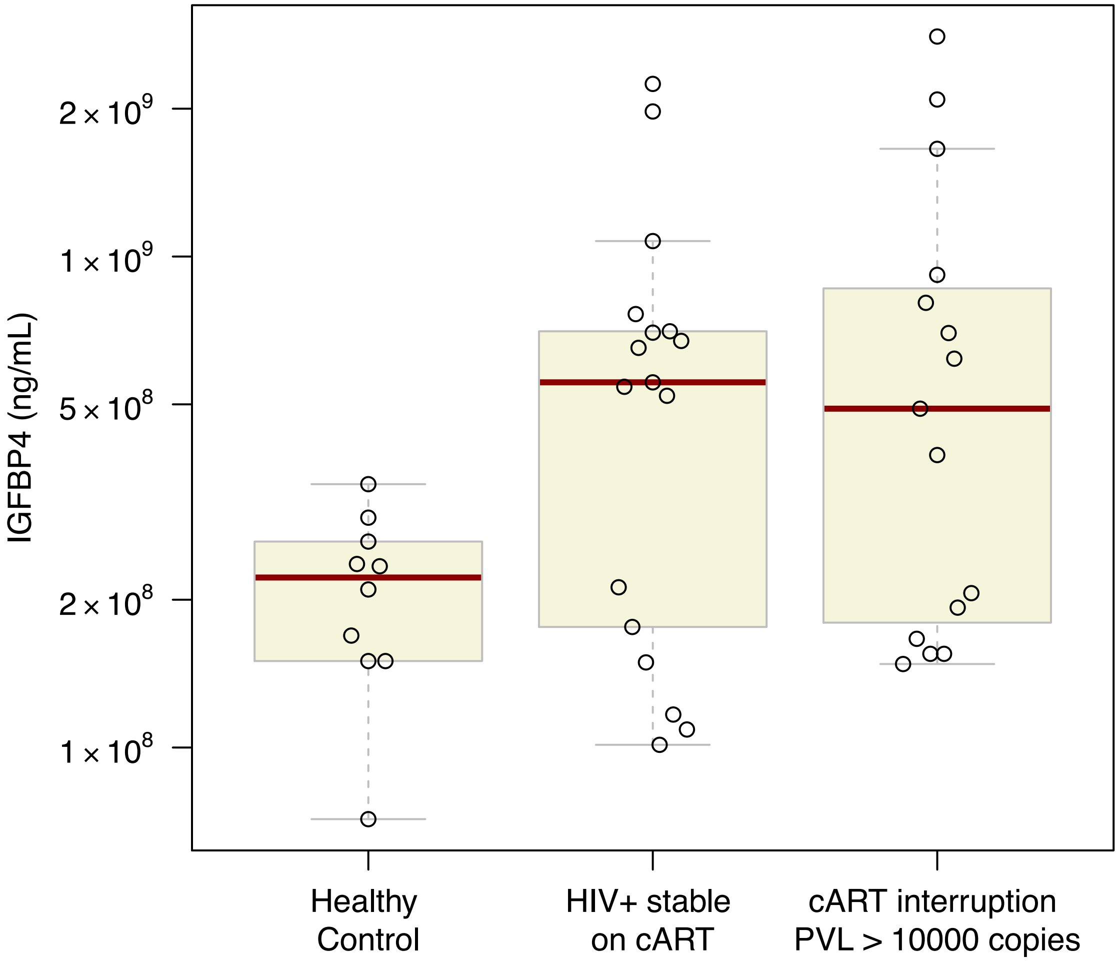

LLOQ: 6 ng/mL

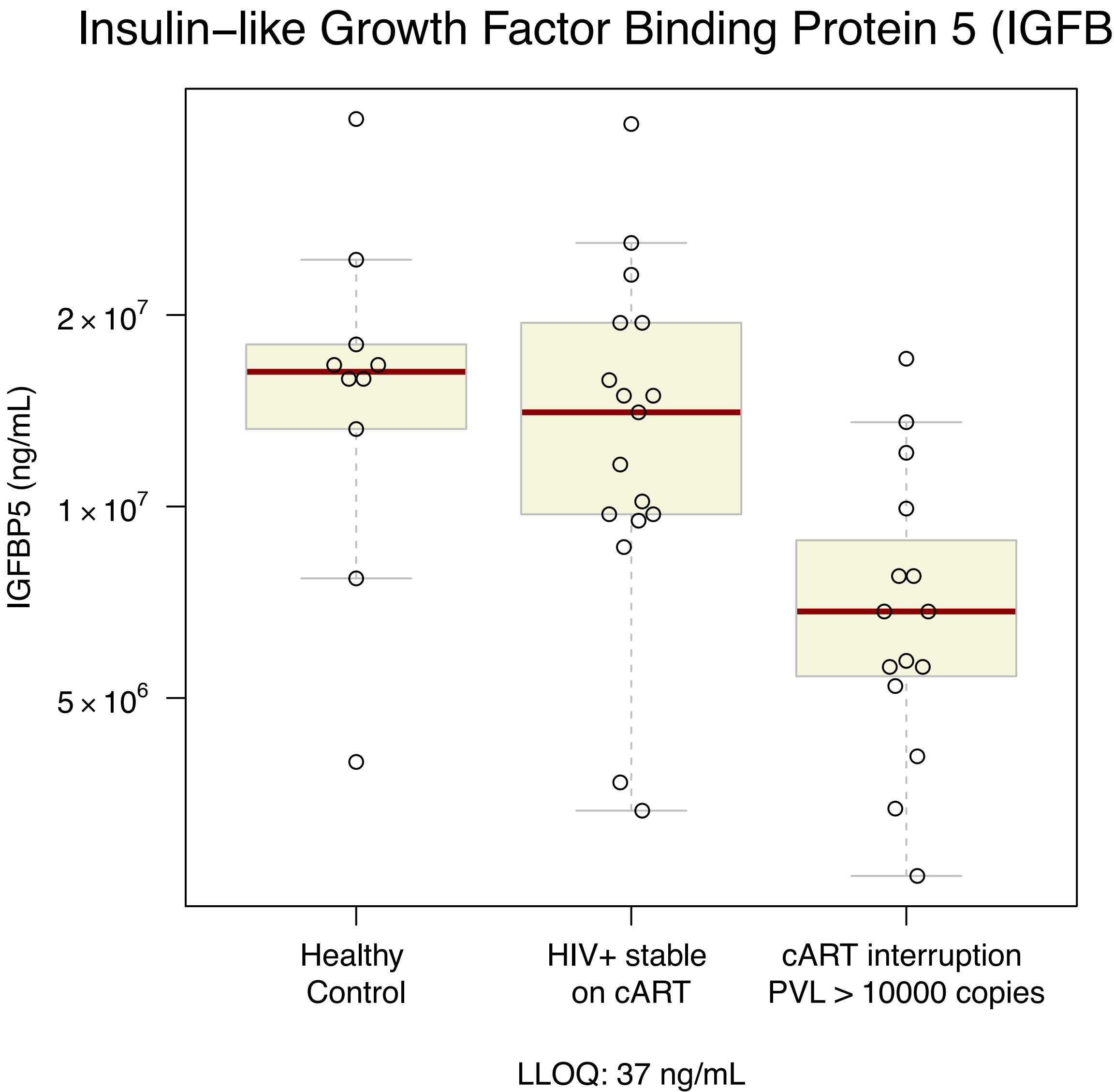

Insulin–like Growth Factor Binding Protein 5 (IGFB
IGFBP5 (ng/mL)
2×10^7
1×10^7
5×10^6
Healthy Control
HIV+ stable on cART
cART interruption PVL > 10000 copies
LLOQ: 37 ng/mL

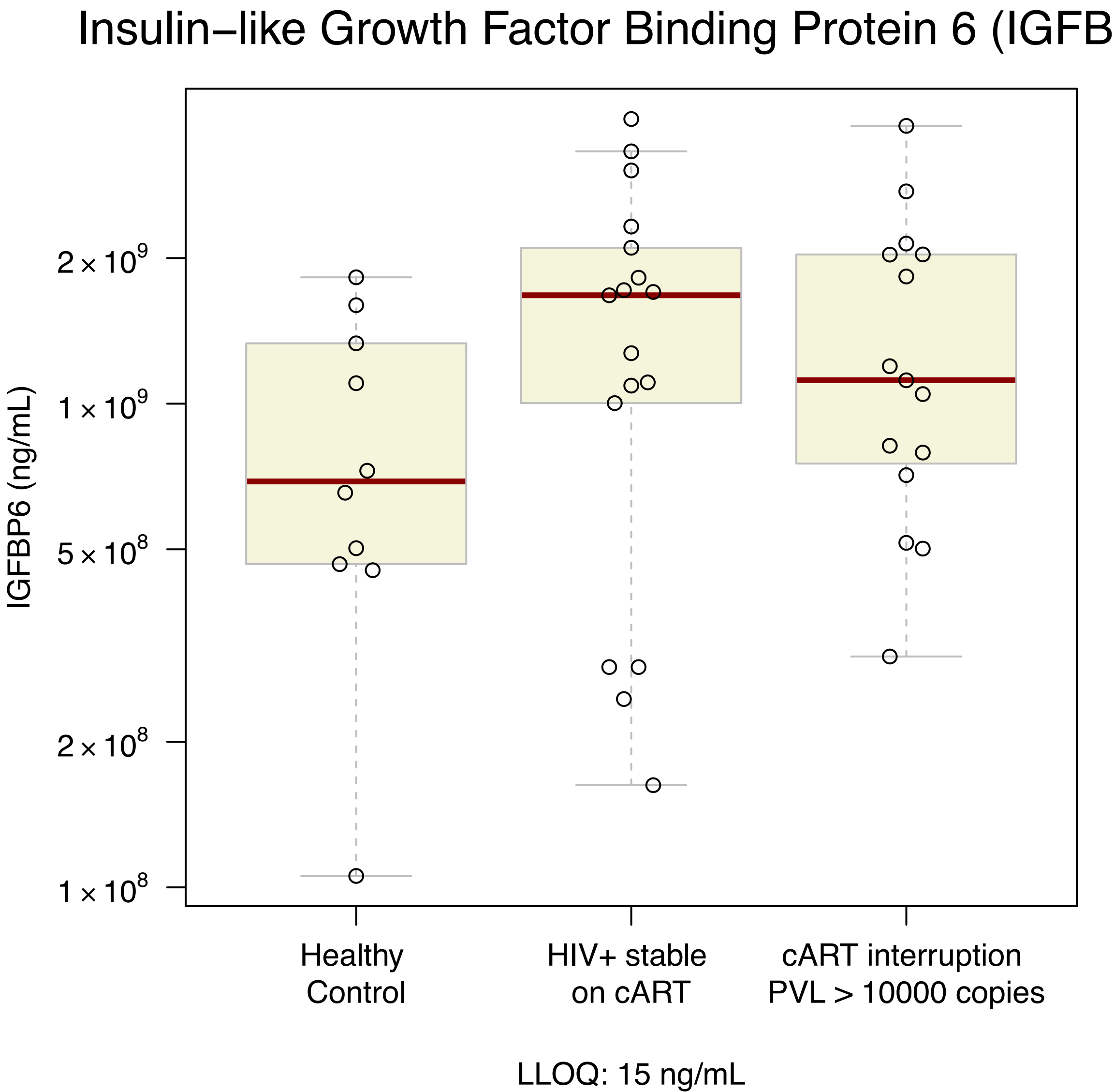

Insulin–like Growth Factor Binding Protein 6 (IGFB
IGFBP6 (ng/mL)
2×10^9
1×10^9
5×10^8
2×10^8
1×10^8
Healthy Control
HIV+ stable on cART
cART interruption PVL > 10000 copies
LLOQ: 15 ng/mL

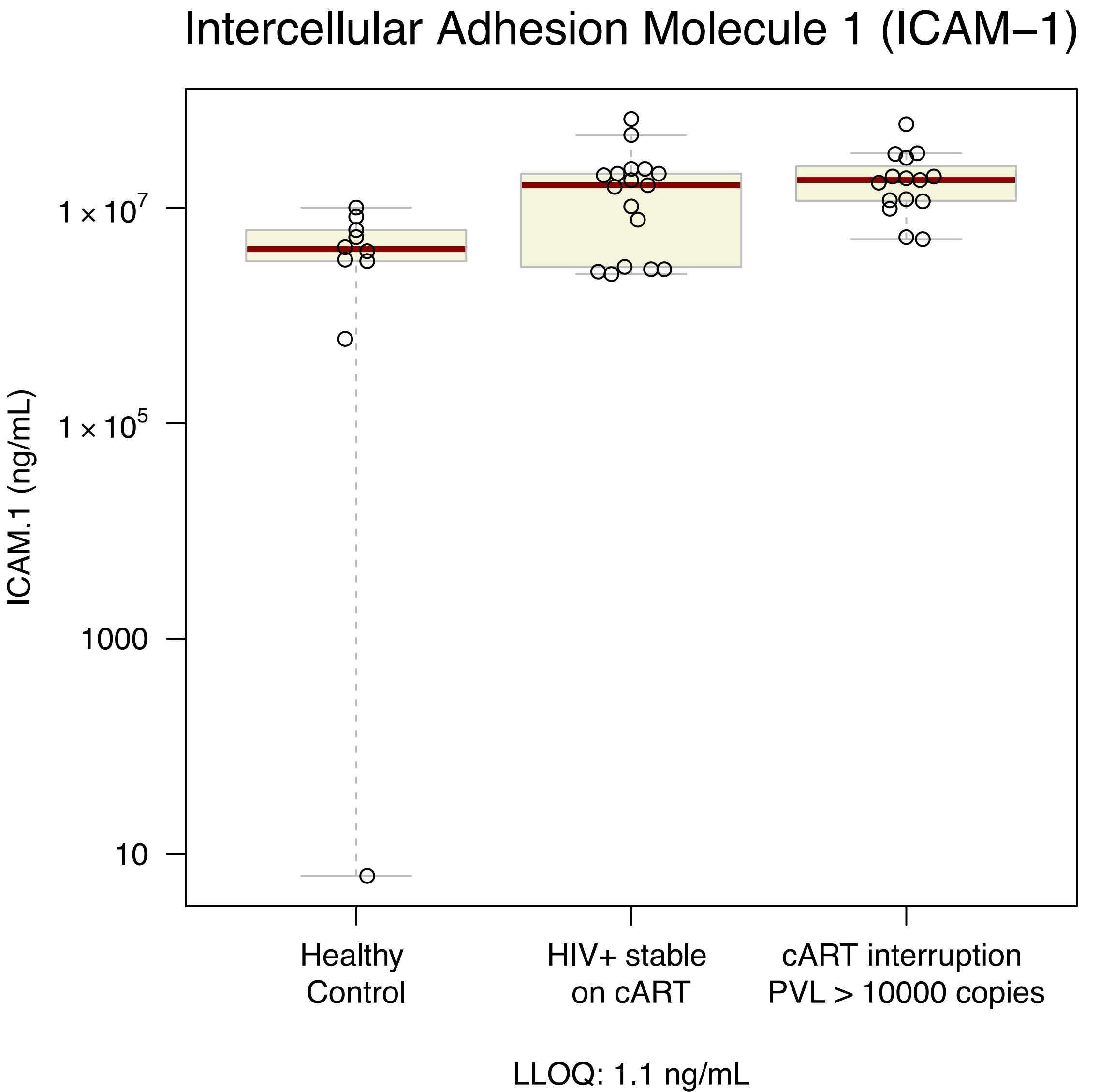

Intercellular Adhesion Molecule 1 (ICAM−1)
ICAM.1 (ng/mL)
1 × 10^7
1 × 10^5
1000
10
Healthy Control
HIV+ stable on cART
cART interruption PVL > 10000 copies
LLOQ: 1.1 ng/mL

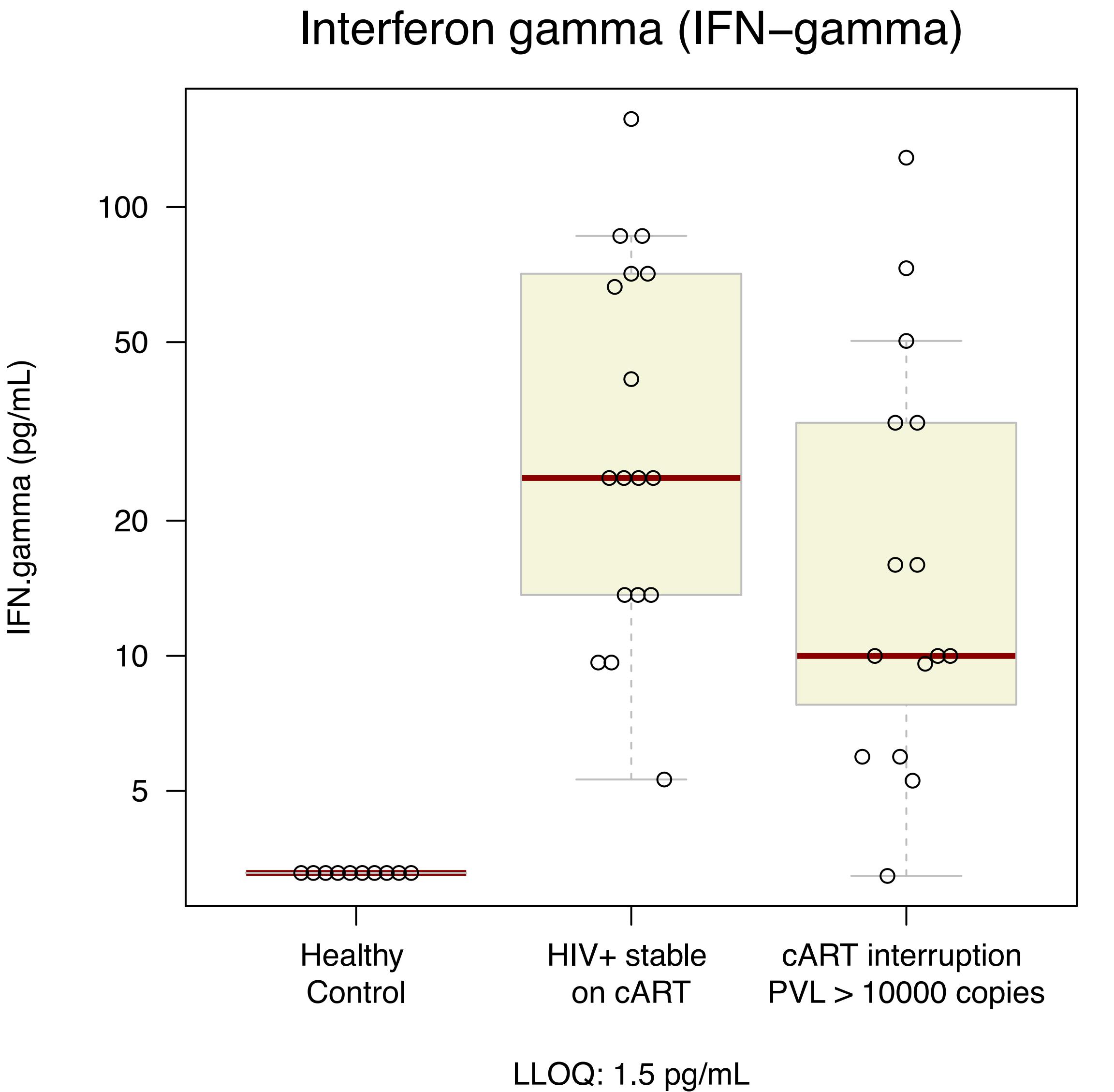
Interferon gamma (IFN–gamma)
IFN.gamma (pg/mL)
100
50
20
10
5
Healthy Control
HIV+ stable on cART
cART interruption PVL > 10000 copies
LLOQ: 1.5 pg/mL

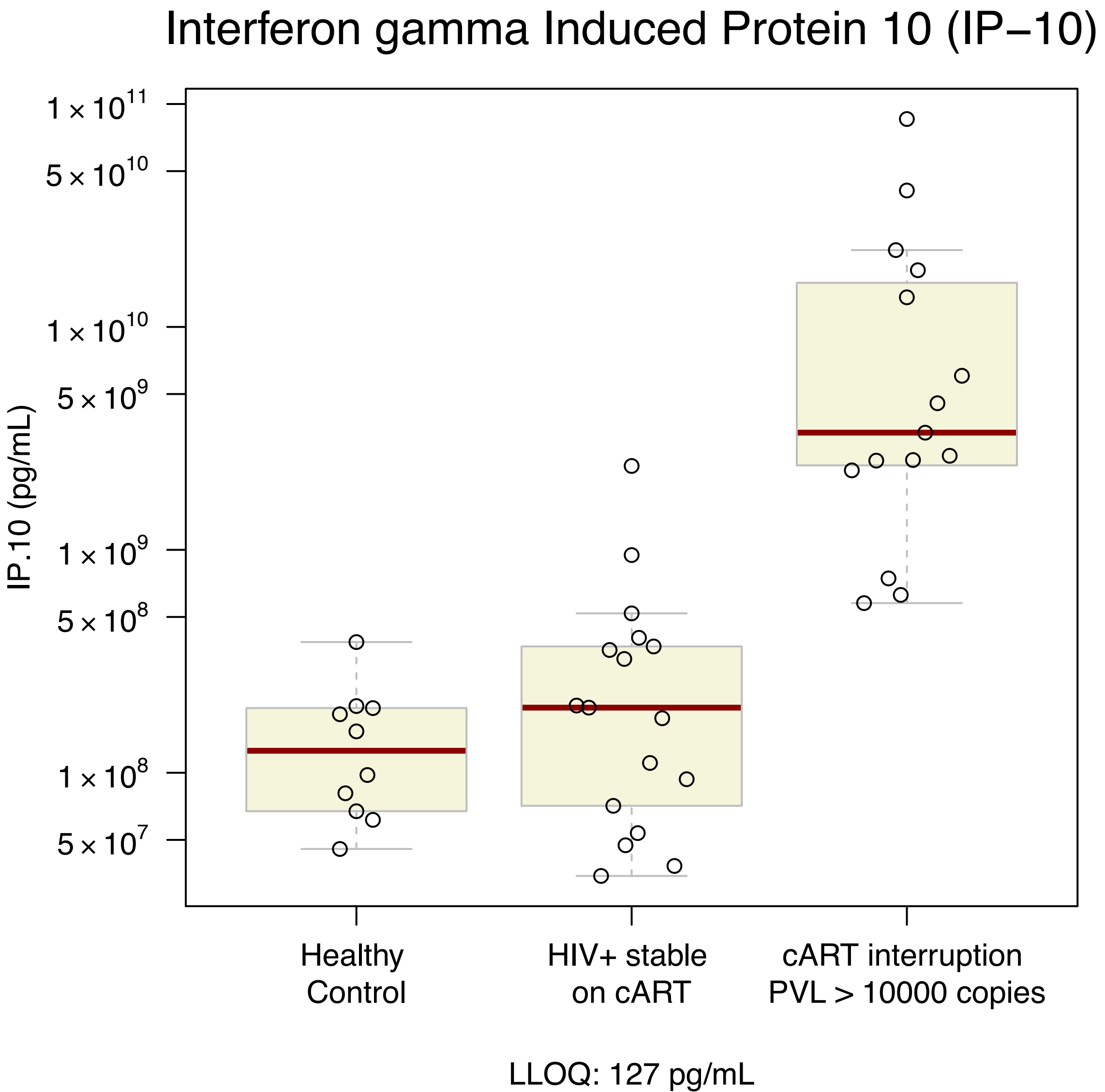
Interferon gamma Induced Protein 10 (IP−10)
IP.10 (pg/mL)
1 × 10^11
5 × 10^10
1 × 10^10
5 × 10^9
1 × 10^9
5 × 10^8
1 × 10^8
5 × 10^7
Healthy Control
HIV+ stable on cART
cART interruption PVL > 10000 copies
LLOQ: 127 pg/mL

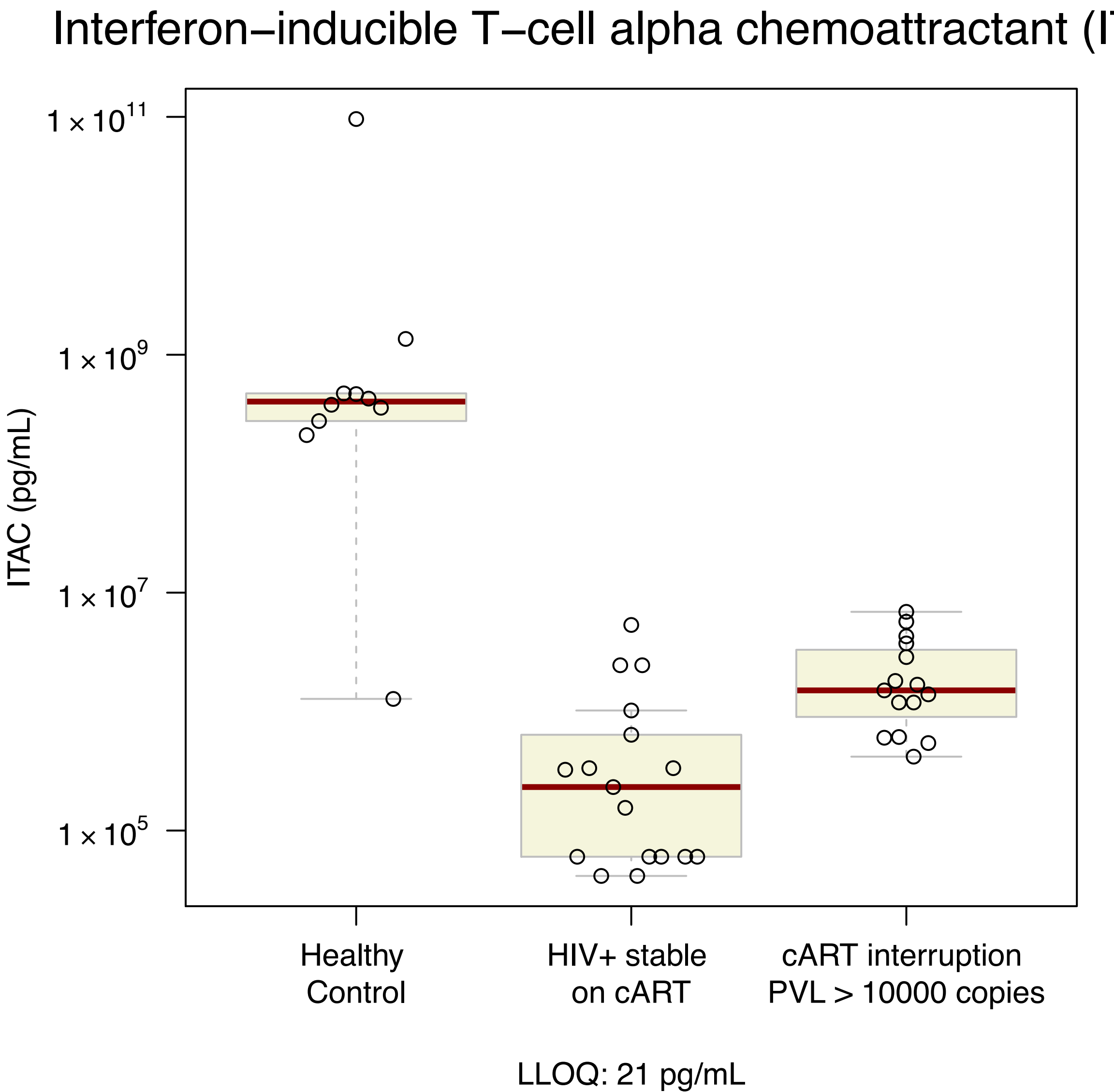
Interferon−inducible T−cell alpha chemoattractant (IT
ITAC (pg/mL)
1×10^{11}
1×10^{9}
1×10^{7}
1×10^{5}
Healthy Control
HIV+ stable on cART
cART interruption PVL > 10000 copies
LLOQ: 21 pg/mL

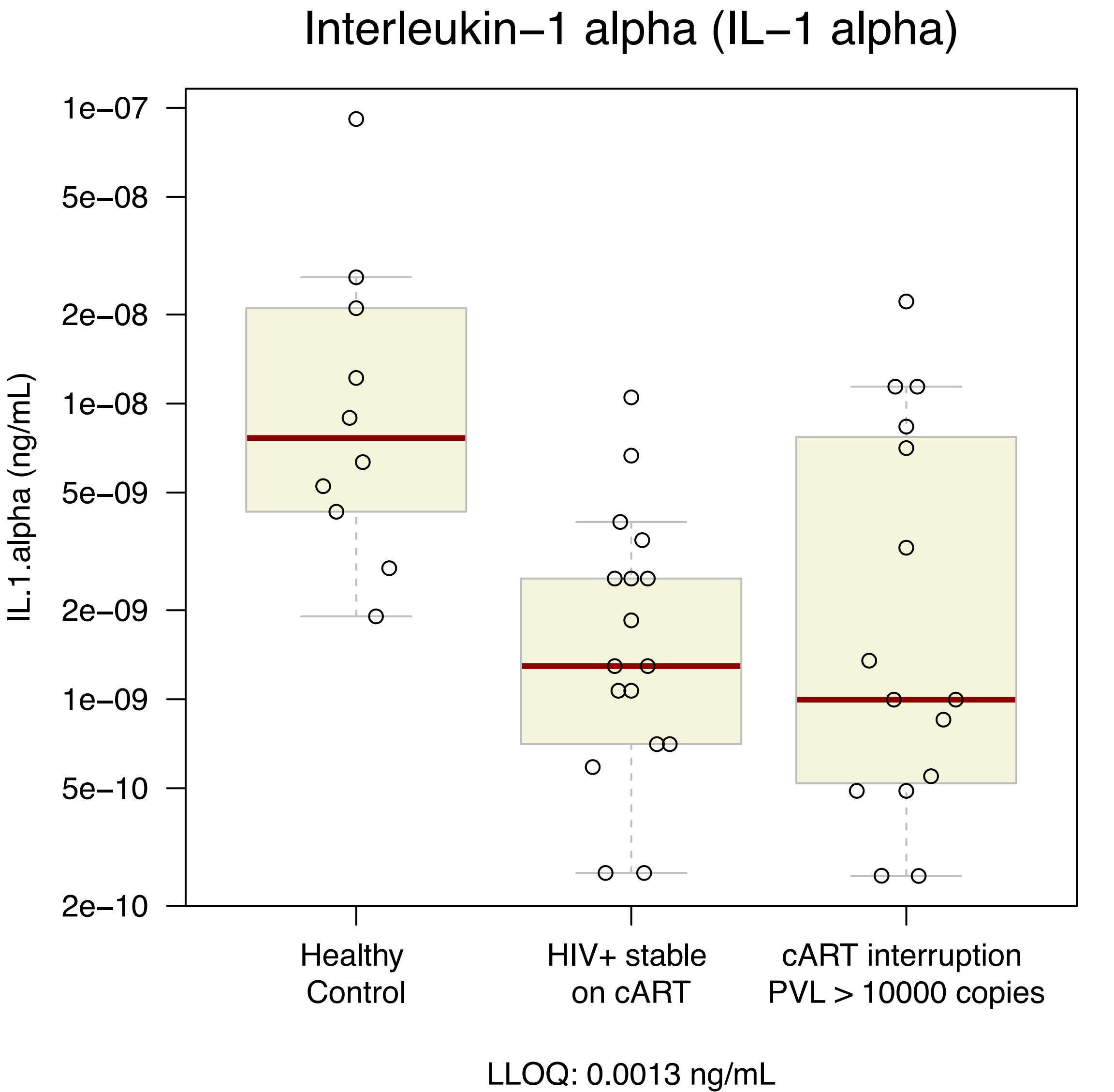
Interleukin−1 alpha (IL−1 alpha)
IL.1.alpha (ng/mL)
1e−07
5e−08
2e−08
1e−08
5e−09
2e−09
1e−09
5e−10
2e−10
Healthy Control
HIV+ stable on cART
cART interruption PVL > 10000 copies
LLOQ: 0.0013 ng/mL

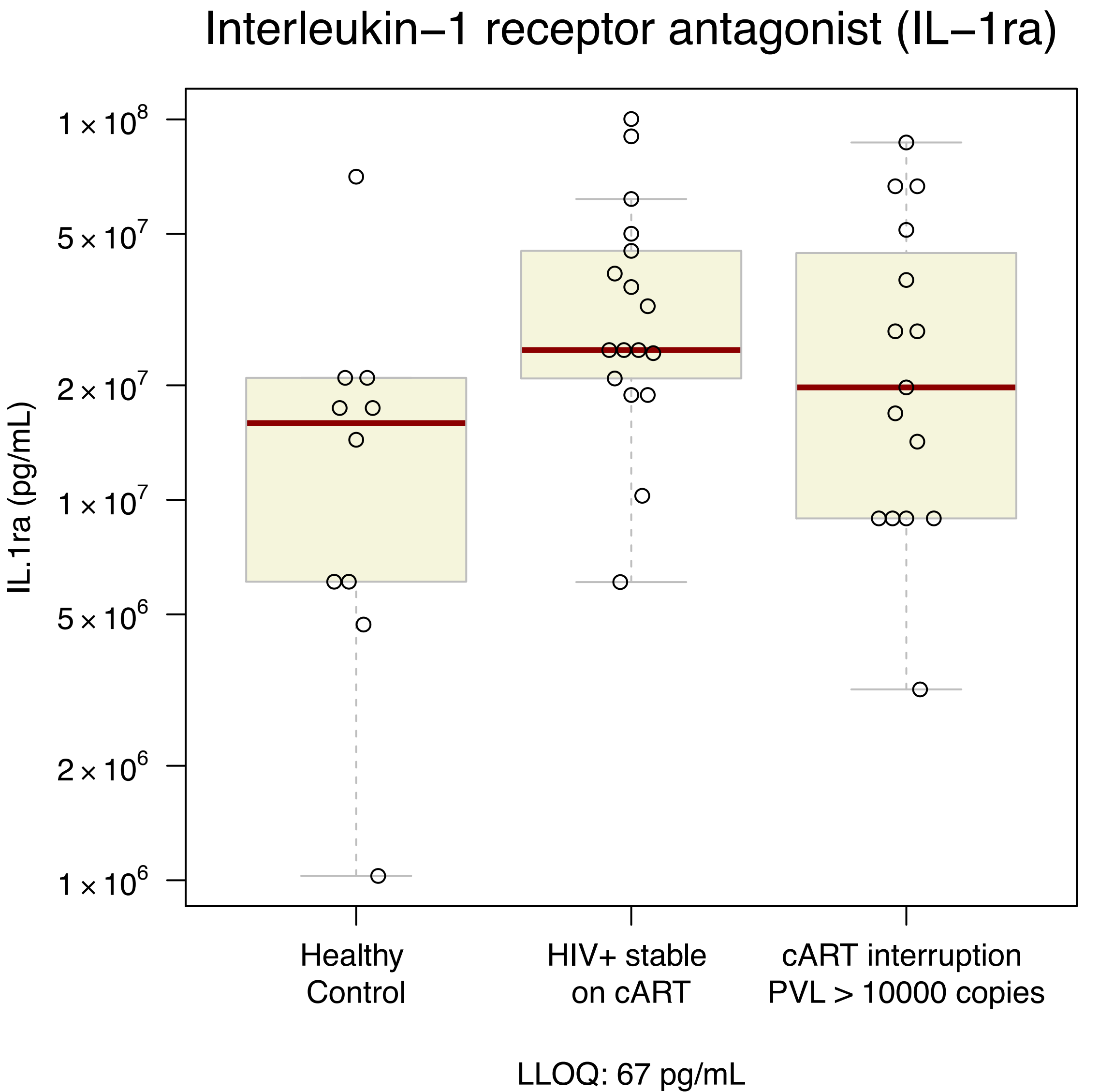
Interleukin−1 receptor antagonist (IL−1ra)
IL.1ra (pg/mL)
1 × 10^8
5 × 10^7
2 × 10^7
1 × 10^7
5 × 10^6
2 × 10^6
1 × 10^6
Healthy Control
HIV+ stable on cART
cART interruption PVL > 10000 copies
LLOQ: 67 pg/mL

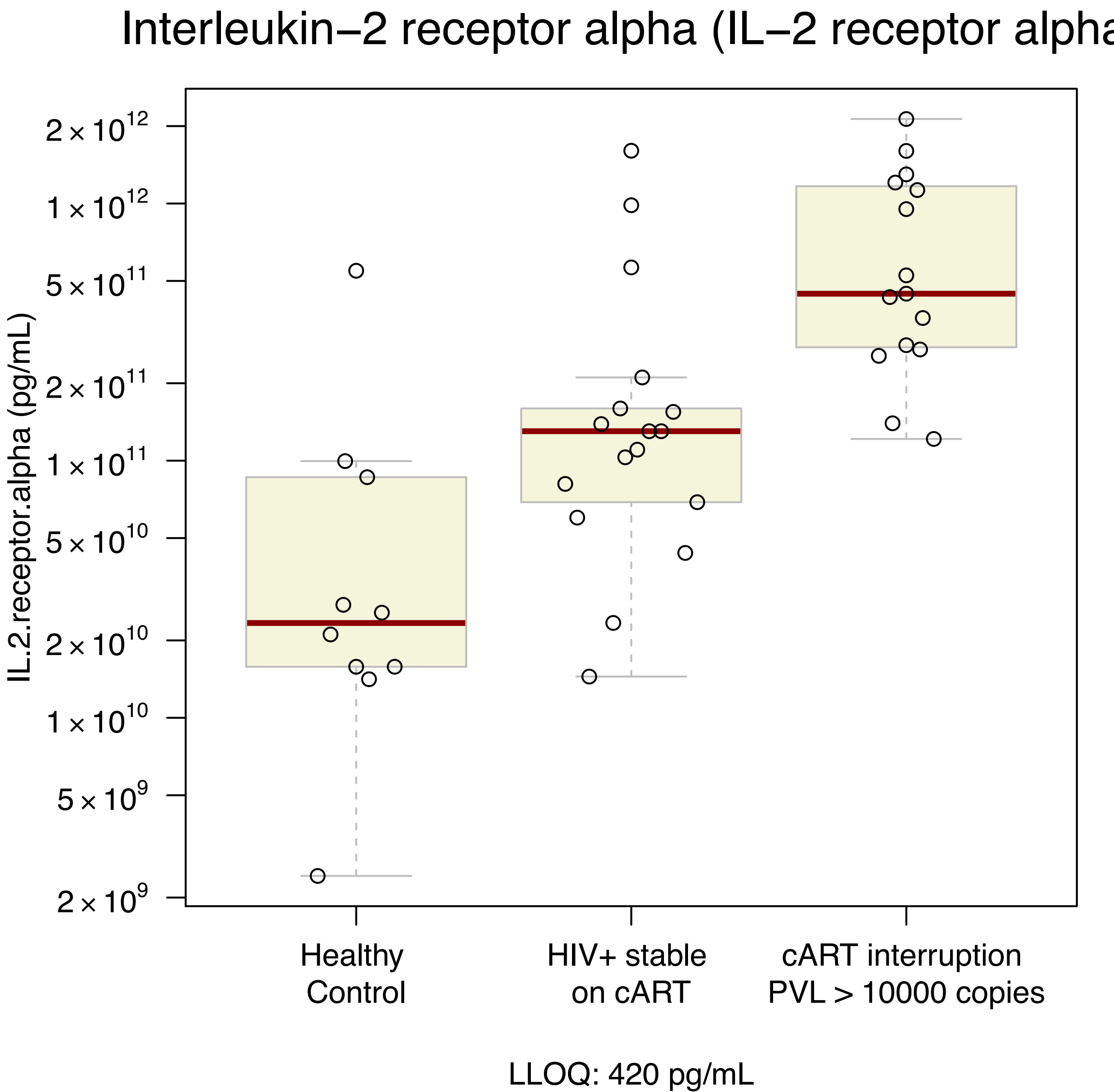
Interleukin−2 receptor alpha (IL−2 receptor alpha
IL.2.receptor.alpha (pg/mL)
2 × 10^12
1 × 10^12
5 × 10^11
2 × 10^11
1 × 10^11
5 × 10^10
2 × 10^10
1 × 10^10
5 × 10^9
2 × 10^9
Healthy Control
HIV+ stable on cART
cART interruption PVL > 10000 copies
LLOQ: 420 pg/mL

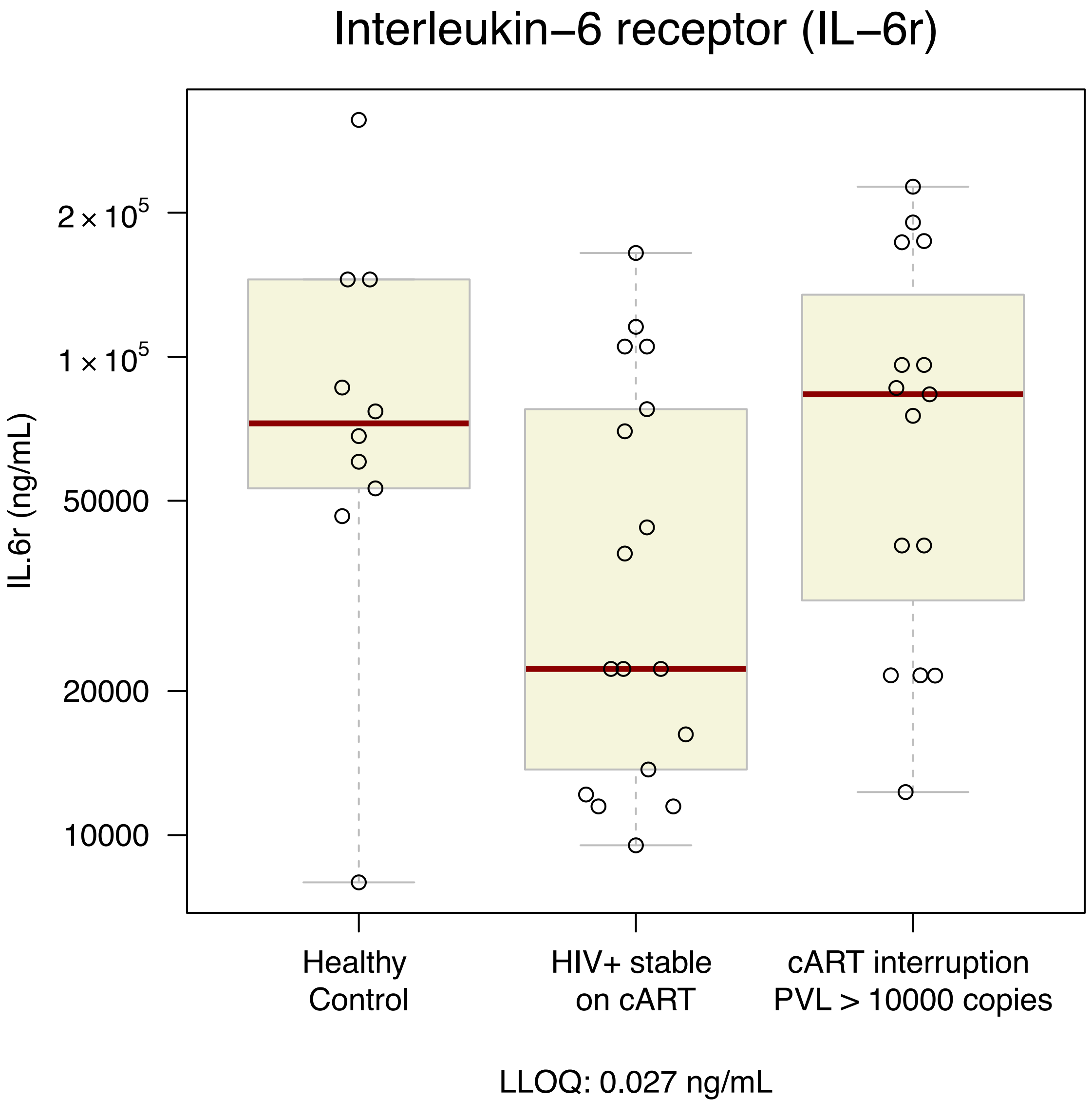
Interleukin–6 receptor (IL–6r)
IL.6r (ng/mL)
2 × 10^5
1 × 10^5
50000
20000
10000
Healthy Control
HIV+ stable on cART
cART interruption PVL > 10000 copies
LLOQ: 0.027 ng/mL

Interleukin−6 receptor subunit beta (IL−6R beta)

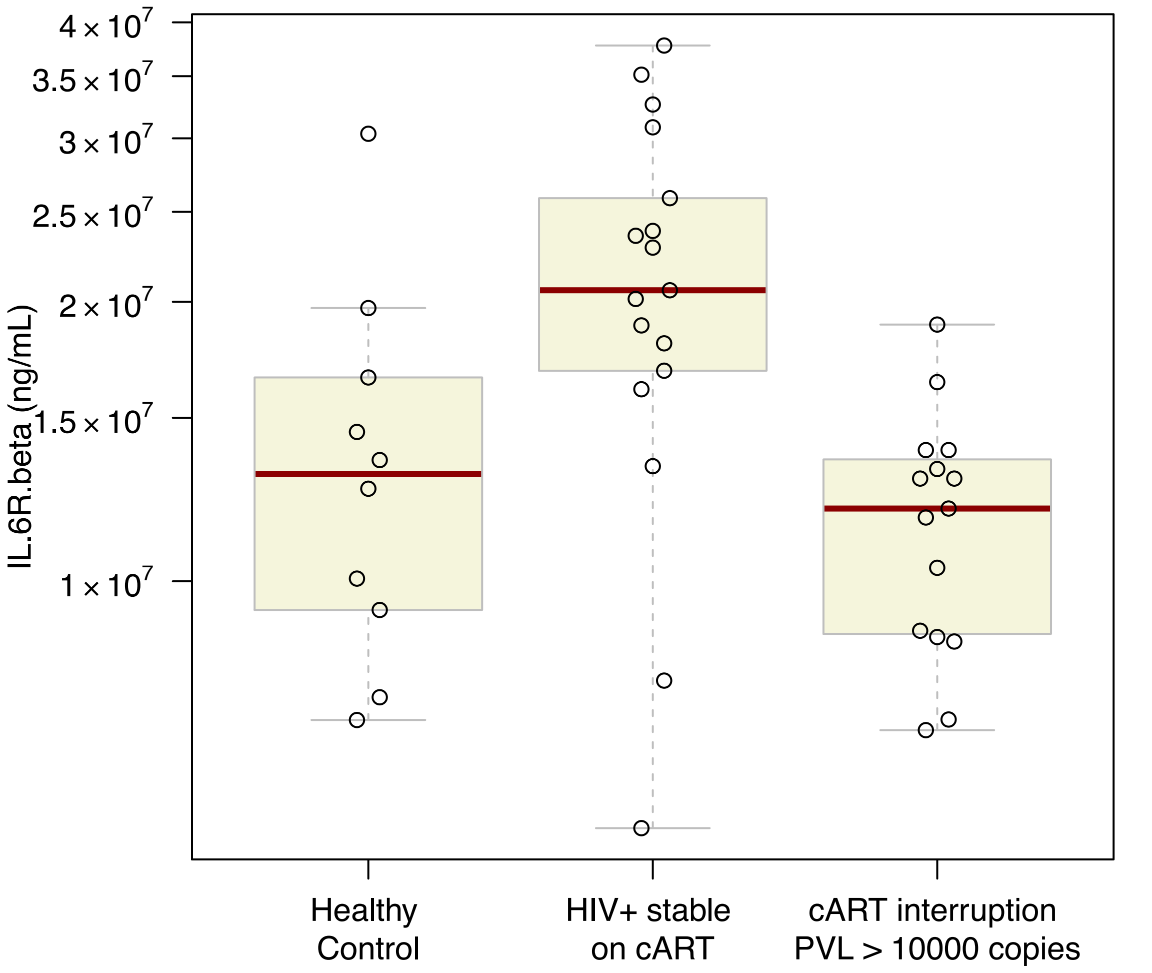

LLOQ: 25 ng/mL

Interleukin−7 (IL−7)

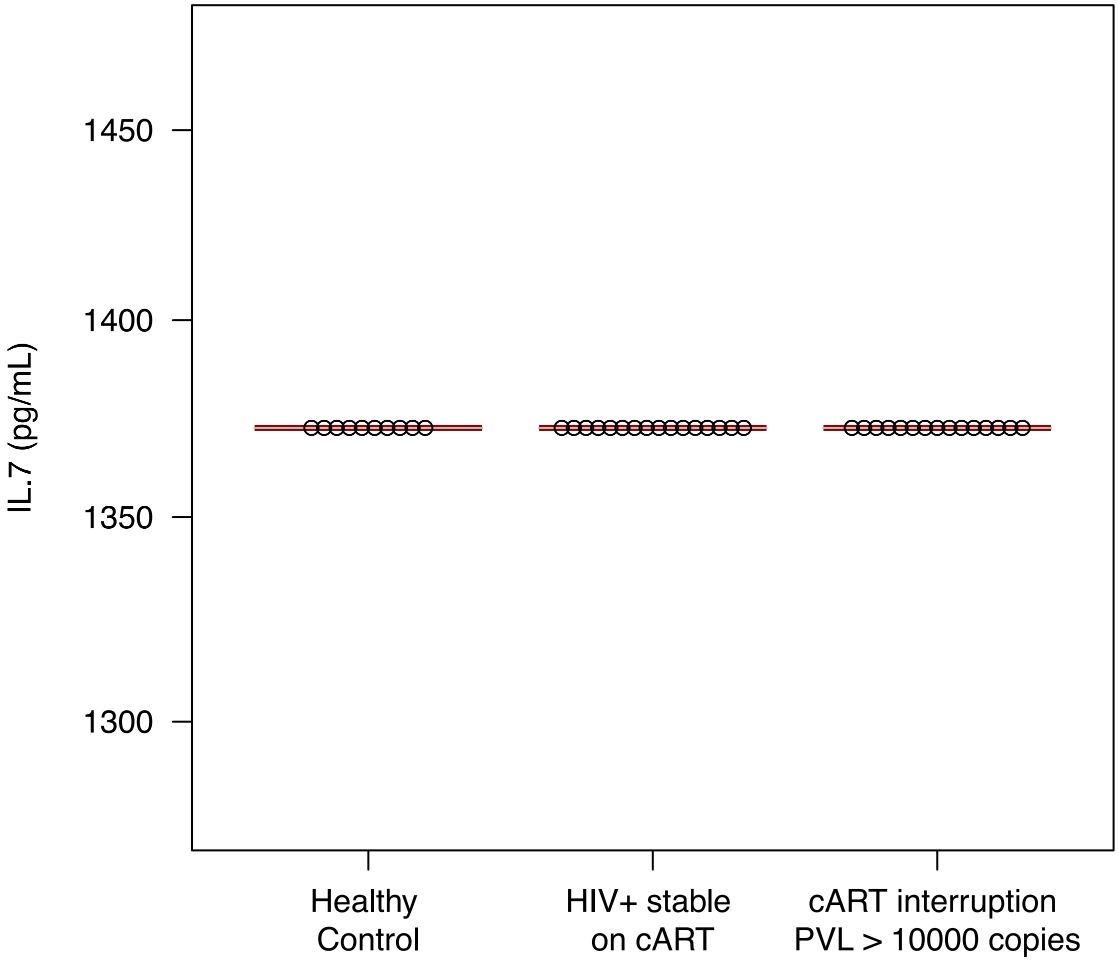

LLOQ: 8.8 pg/mL

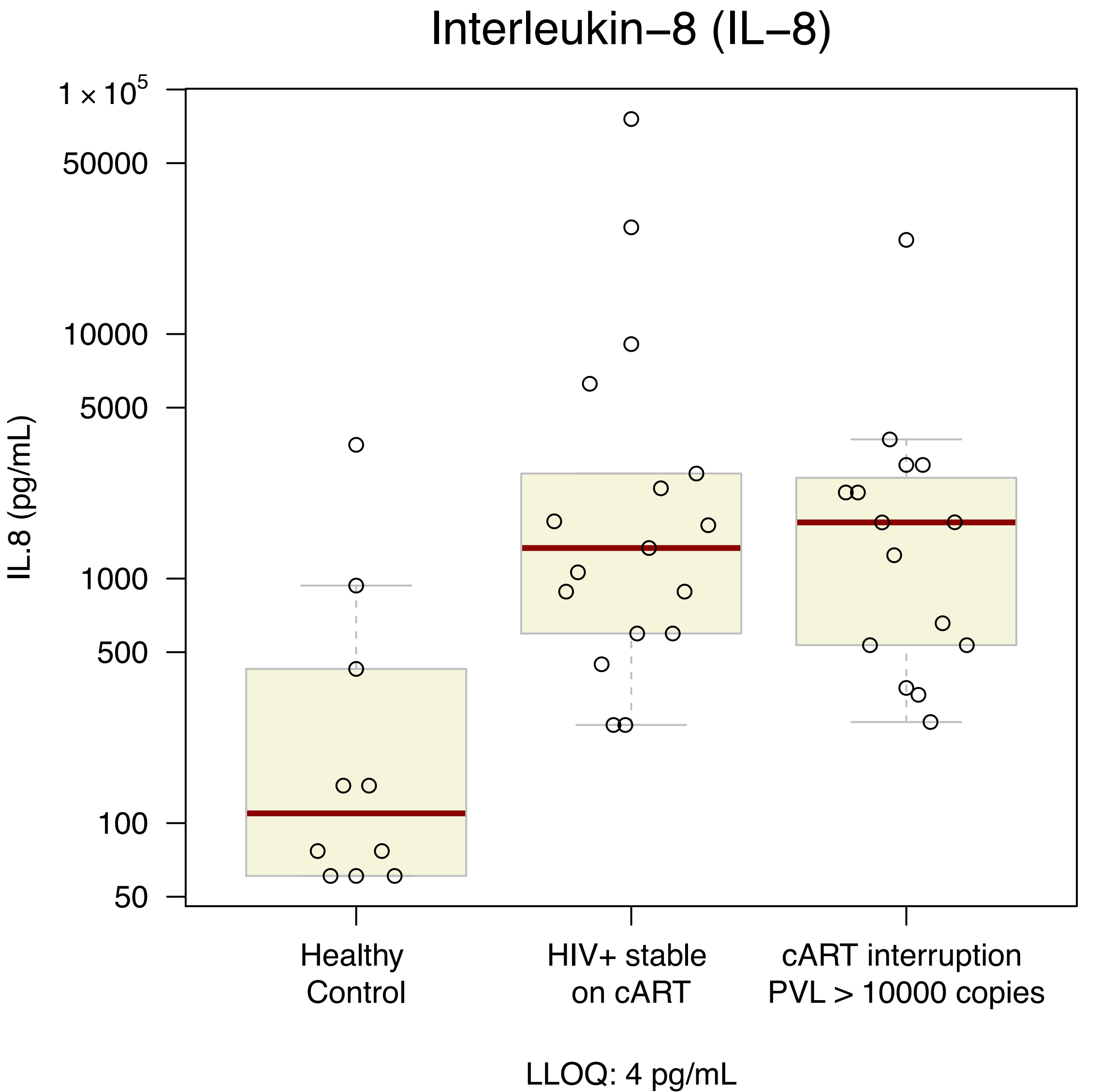
Interleukin−8 (IL−8)
IL.8 (pg/mL)
1 × 10^5
50000
10000
5000
1000
500
100
50
Healthy Control
HIV+ stable on cART
cART interruption PVL > 10000 copies
LLOQ: 4 pg/mL

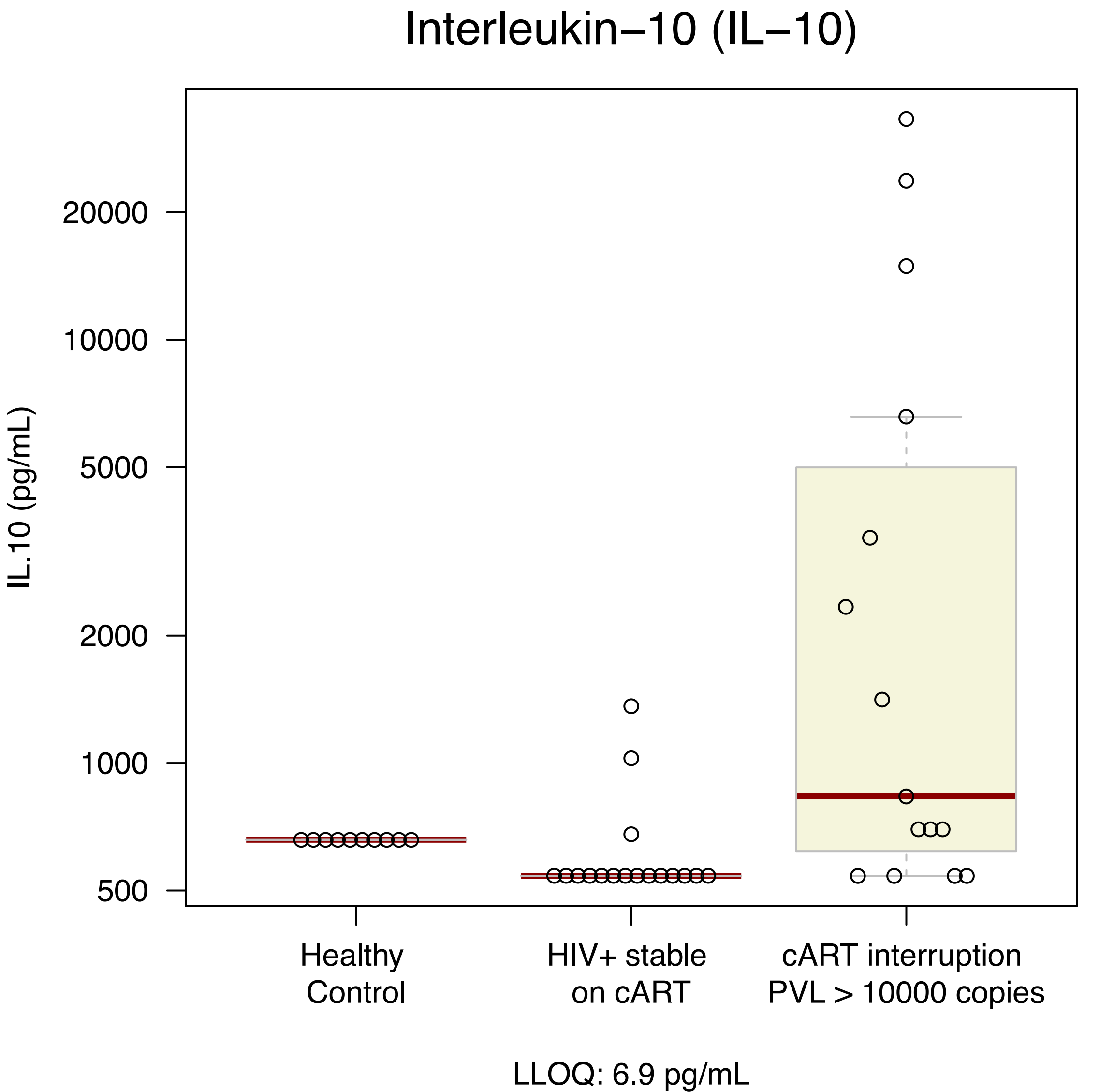

Interleukin−10 (IL−10)
IL.10 (pg/mL)
20000
10000
5000
2000
1000
500
Healthy Control
HIV+ stable on cART
cART interruption PVL > 10000 copies
LLOQ: 6.9 pg/mL

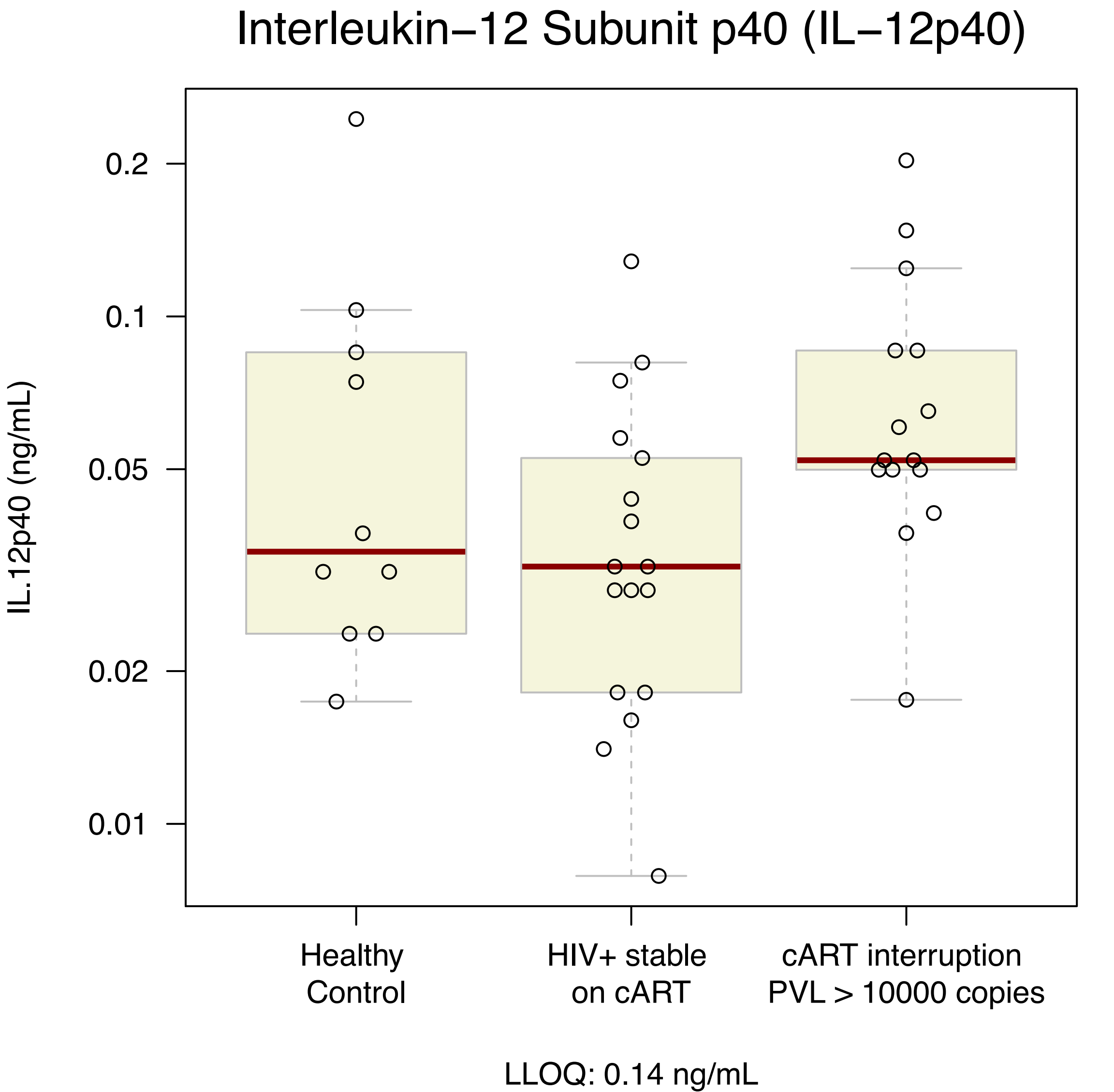

Interleukin−12 Subunit p40 (IL−12p40)
IL.12p40 (ng/mL)
0.2
0.1
0.05
0.02
0.01
Healthy Control
HIV+ stable on cART
cART interruption PVL > 10000 copies
LLOQ: 0.14 ng/mL

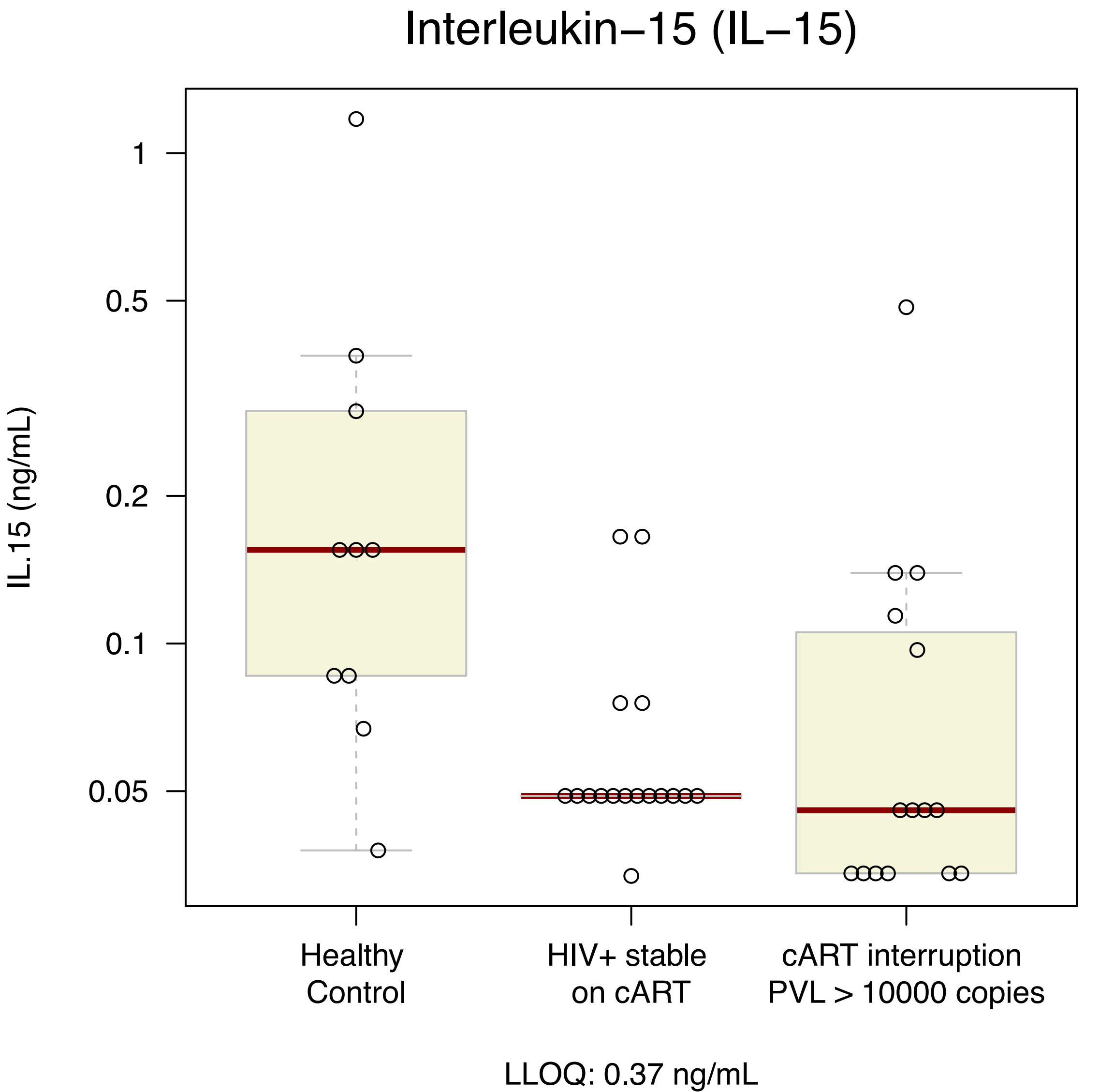
Interleukin−15 (IL−15)
IL.15 (ng/mL)
1
0.5
0.2
0.1
0.05
Healthy Control
HIV+ stable on cART
cART interruption PVL > 10000 copies
LLOQ: 0.37 ng/mL

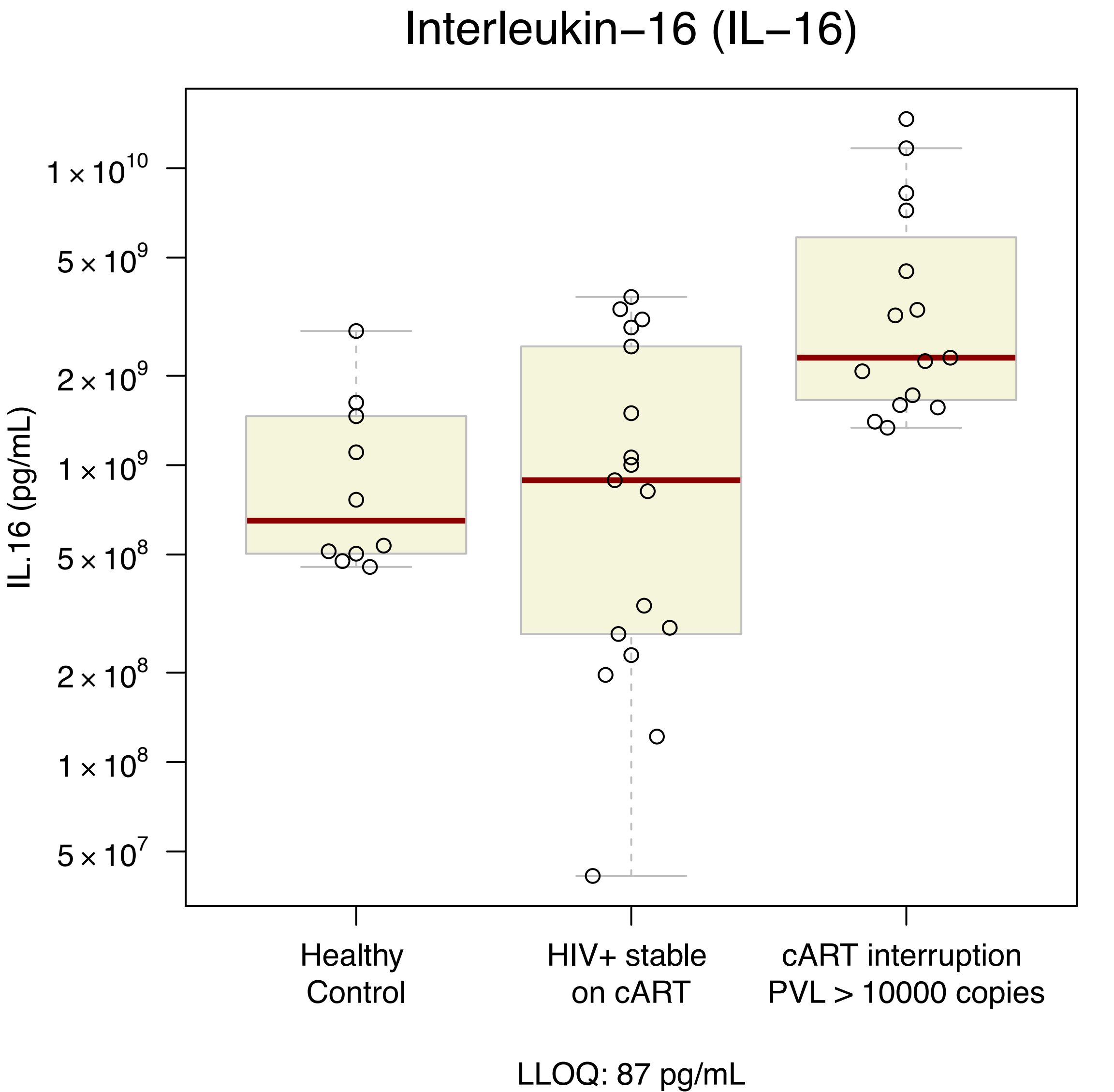

Interleukin−16 (IL−16)
IL.16 (pg/mL)
1 × 10^10
5 × 10^9
2 × 10^9
1 × 10^9
5 × 10^8
2 × 10^8
1 × 10^8
5 × 10^7
Healthy Control
HIV+ stable on cART
cART interruption PVL > 10000 copies
LLOQ: 87 pg/mL

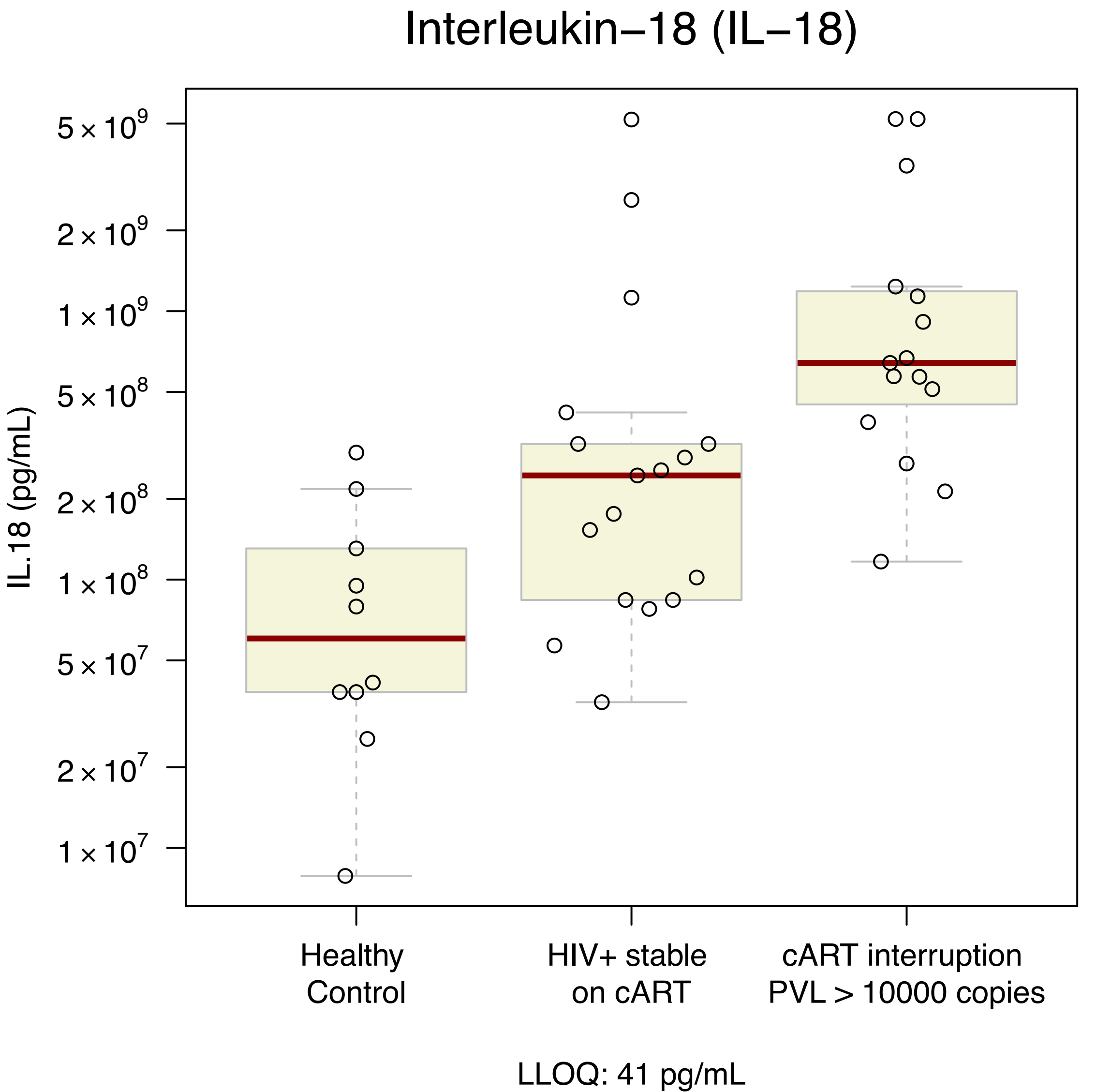
Interleukin−18 (IL−18)
IL.18 (pg/mL)
5×10^9
2×10^9
1×10^9
5×10^8
2×10^8
1×10^8
5×10^7
2×10^7
1×10^7
Healthy Control
HIV+ stable on cART
cART interruption PVL > 10000 copies
LLOQ: 41 pg/mL

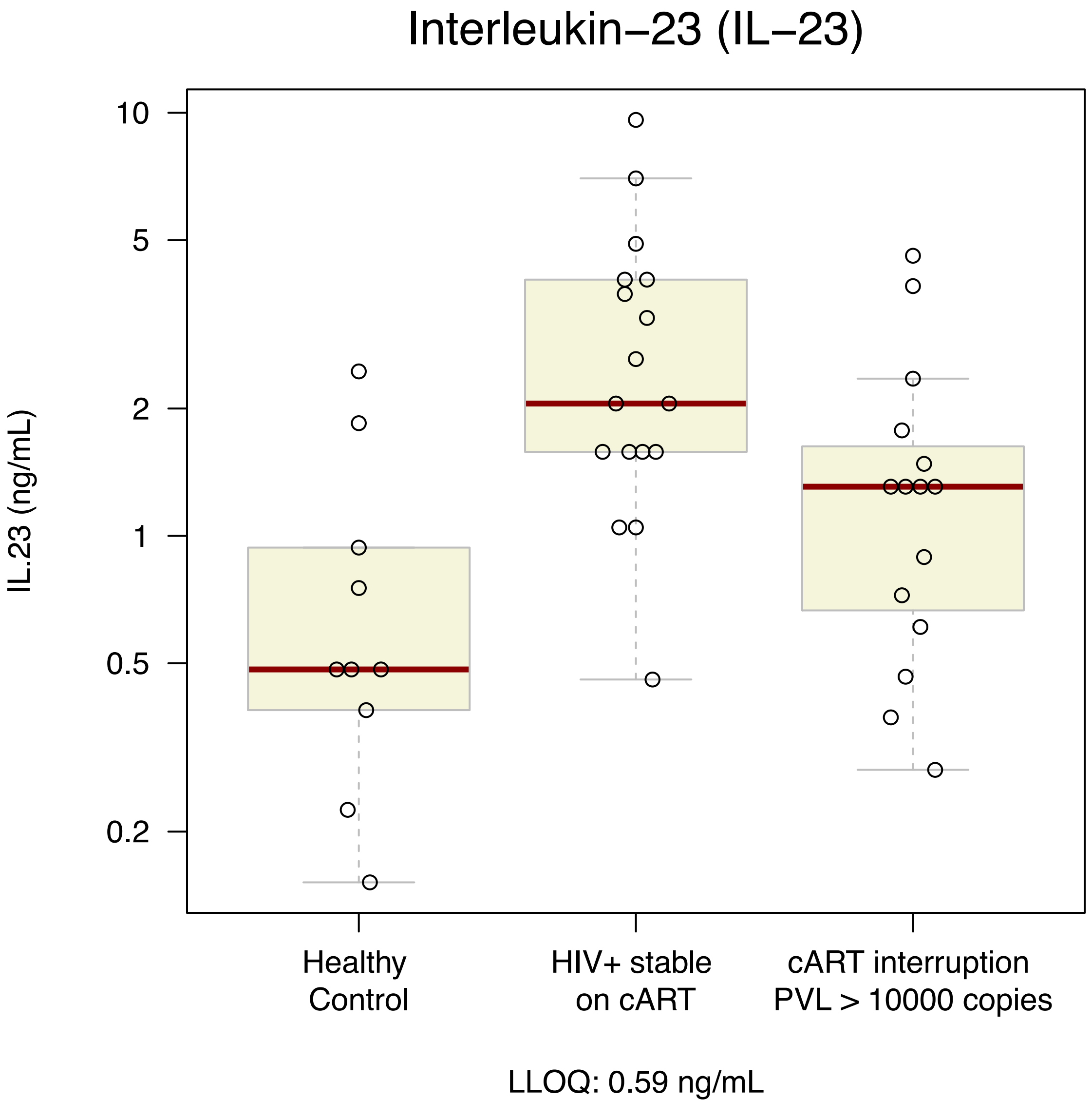
Interleukin−23 (IL−23)
IL.23 (ng/mL)
10
5
2
1
0.5
0.2
Healthy Control
HIV+ stable on cART
cART interruption PVL > 10000 copies
LLOQ: 0.59 ng/mL

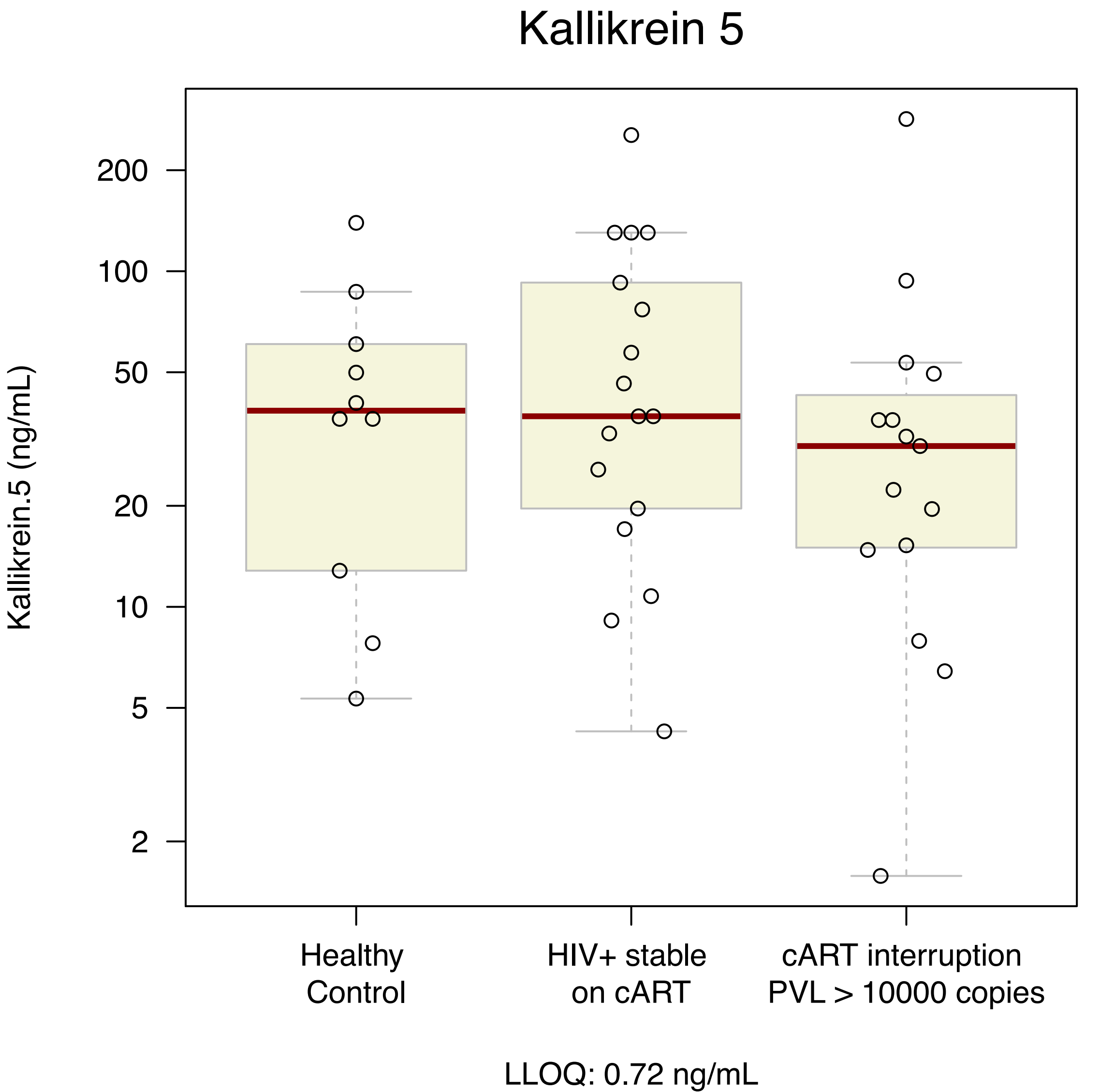

Kallikrein 5
Kallikrein.5 (ng/mL)
200
100
50
20
10
5
2
Healthy Control
HIV+ stable on cART
cART interruption PVL > 10000 copies
LLOQ: 0.72 ng/mL

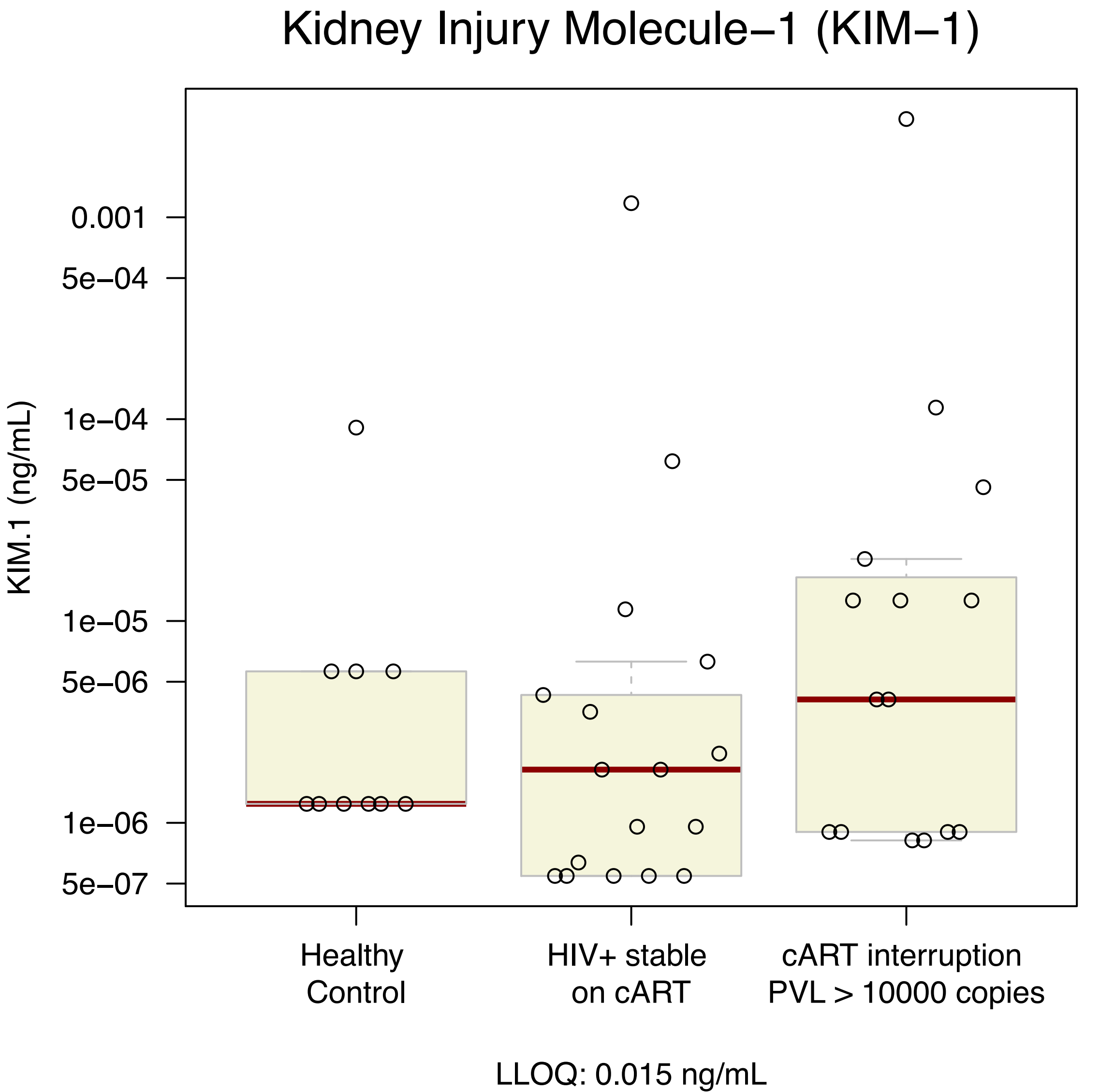
Kidney Injury Molecule−1 (KIM−1)
0.001
5e−04
1e−04
5e−05
1e−05
5e−06
1e−06
5e−07
KIM.1 (ng/mL)
Healthy Control
HIV+ stable on cART
cART interruption PVL > 10000 copies
LLOQ: 0.015 ng/mL

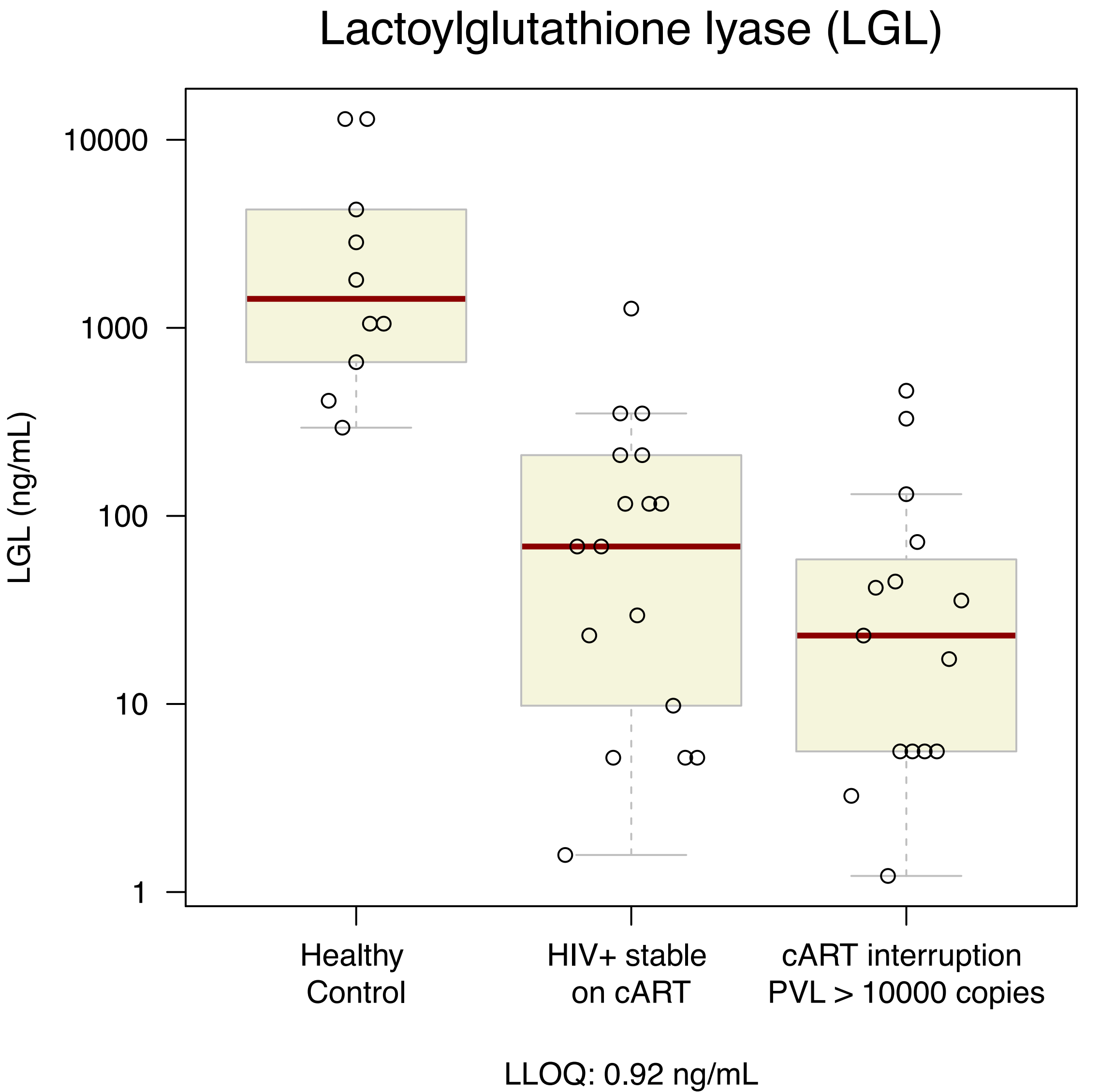
Lactoylglutathione lyase (LGL)
LGL (ng/mL)
10000
1000
100
10
1
Healthy Control
HIV+ stable on cART
cART interruption PVL > 10000 copies
LLOQ: 0.92 ng/mL

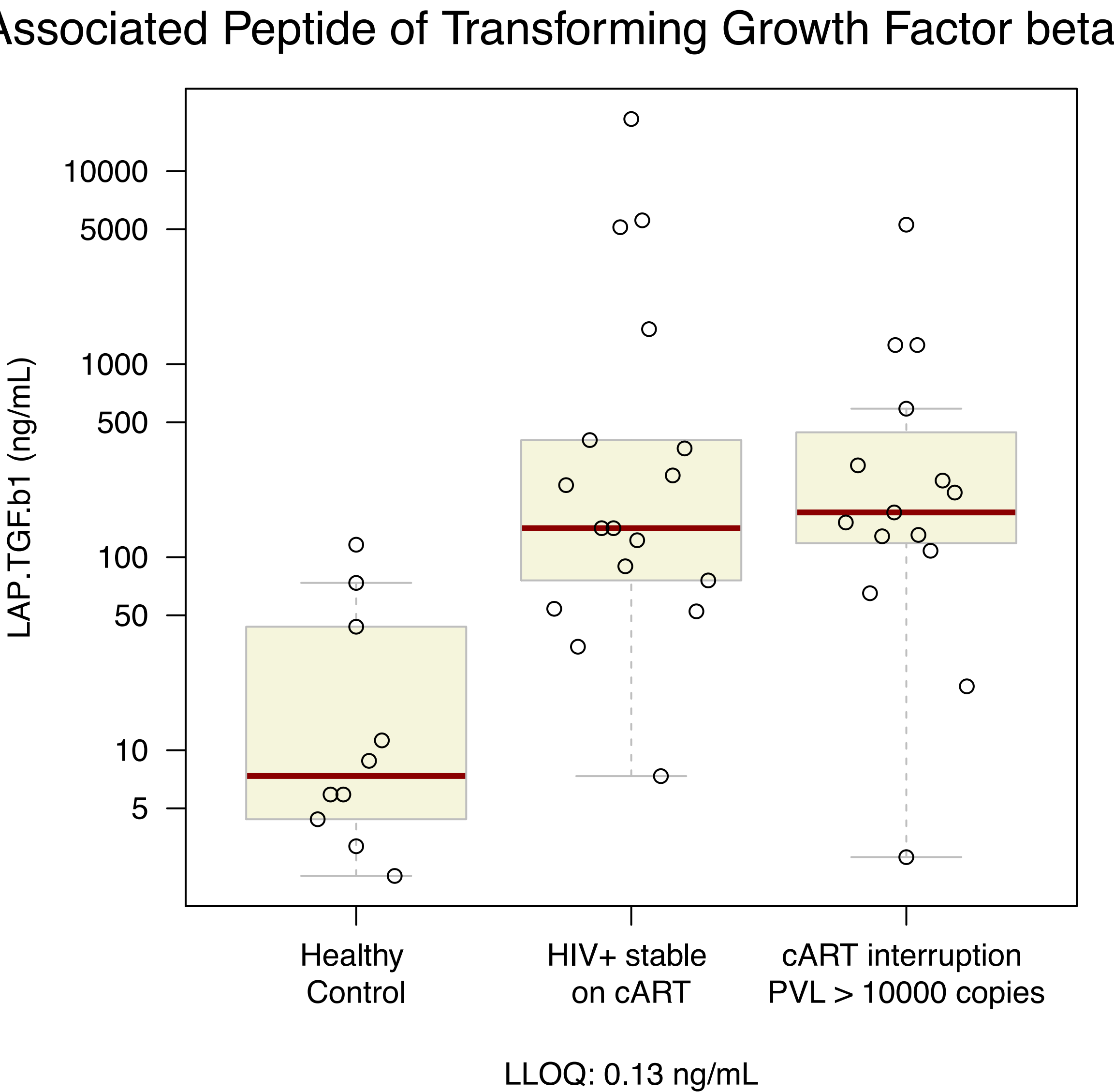

Associated Peptide of Transforming Growth Factor beta
LAP.TGF.b1 (ng/mL)
10000
5000
1000
500
100
50
10
5
Healthy Control
HIV+ stable on cART
cART interruption PVL > 10000 copies
LLOQ: 0.13 ng/mL

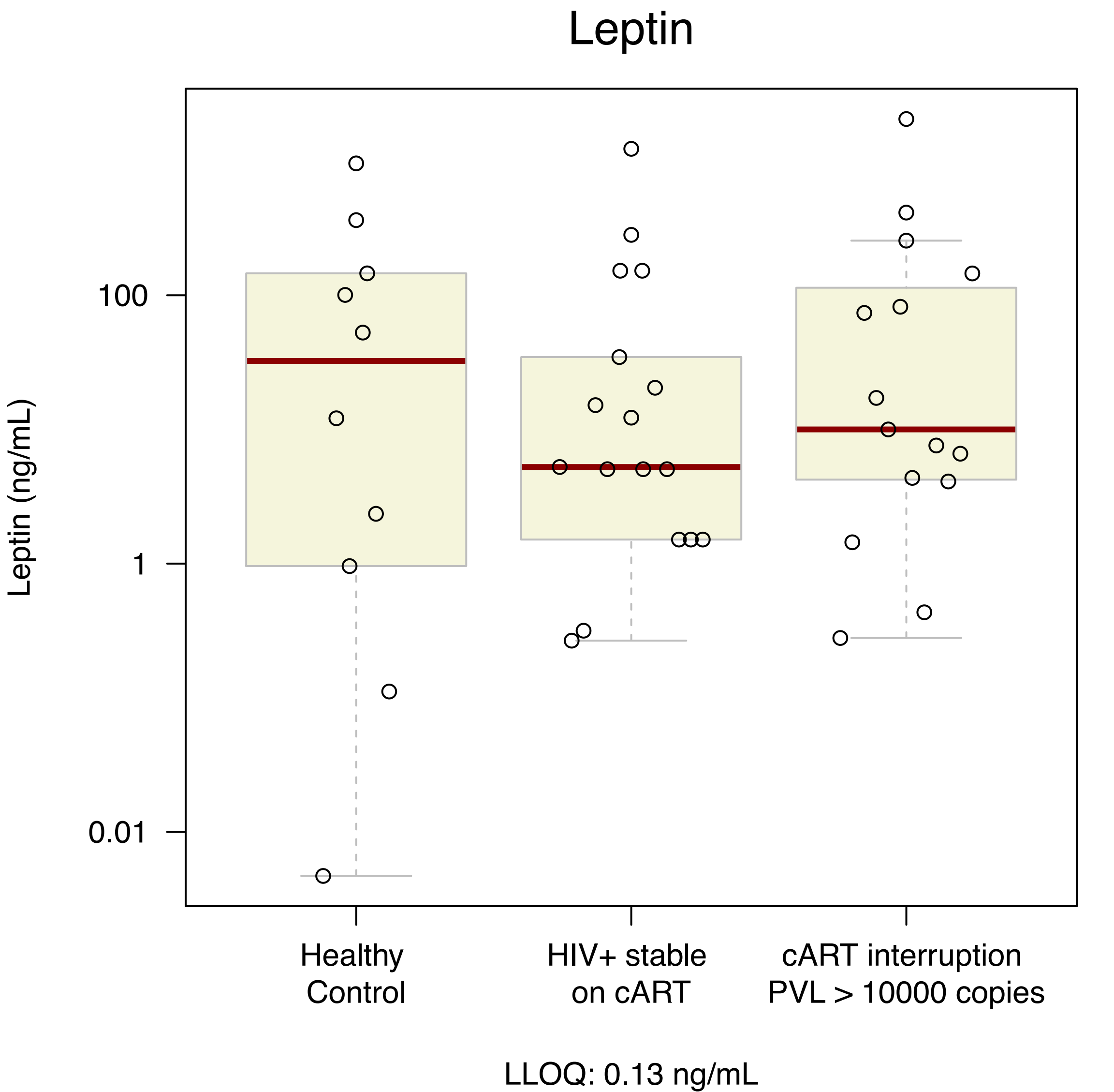
Leptin
Leptin (ng/mL)
100
1
0.01
Healthy Control
HIV+ stable on cART
cART interruption PVL > 10000 copies
LLOQ: 0.13 ng/mL

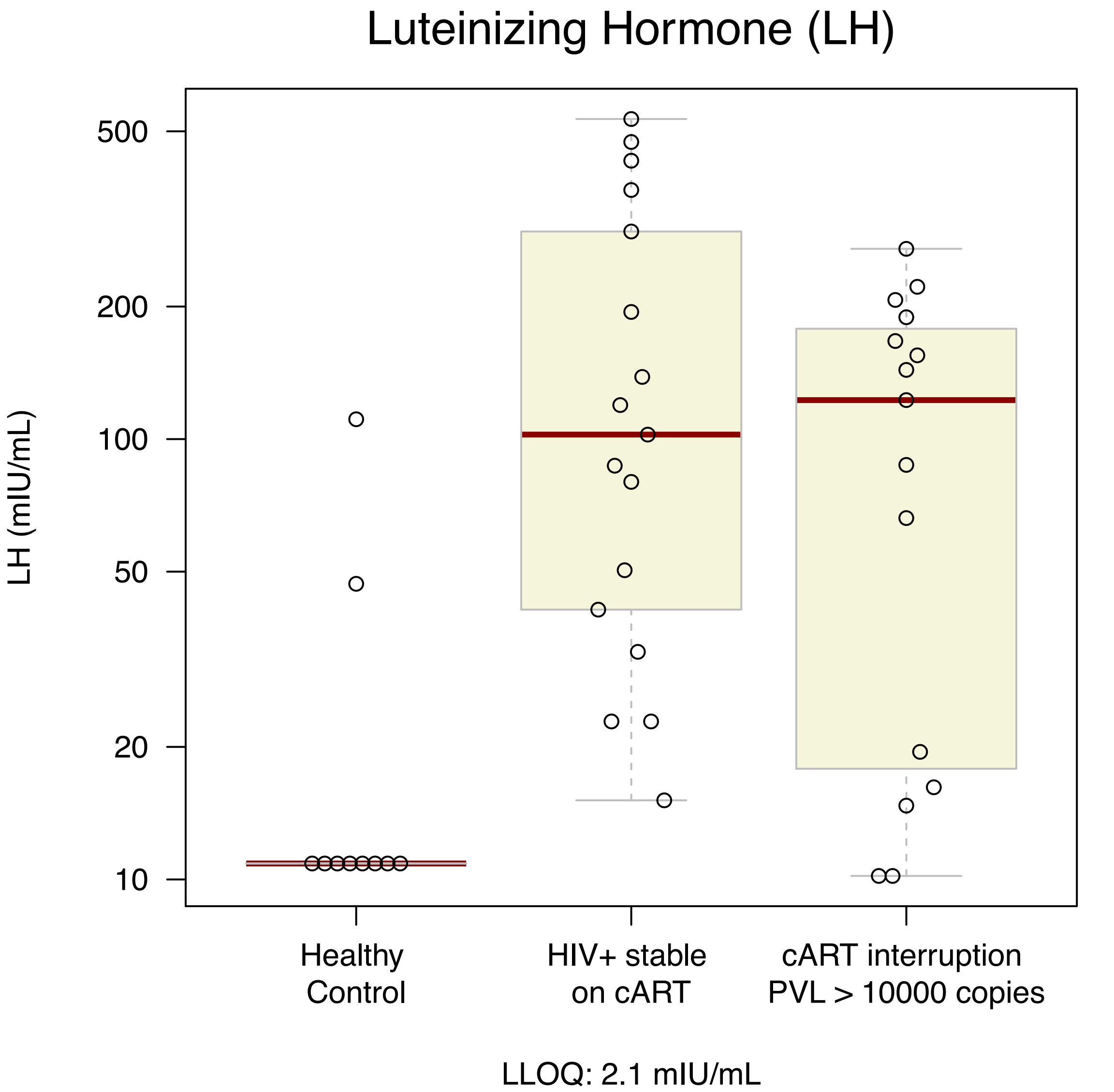

Luteinizing Hormone (LH)
LH (mIU/mL)
500
200
100
50
20
10
Healthy Control
HIV+ stable on cART
cART interruption PVL > 10000 copies
LLOQ: 2.1 mIU/mL

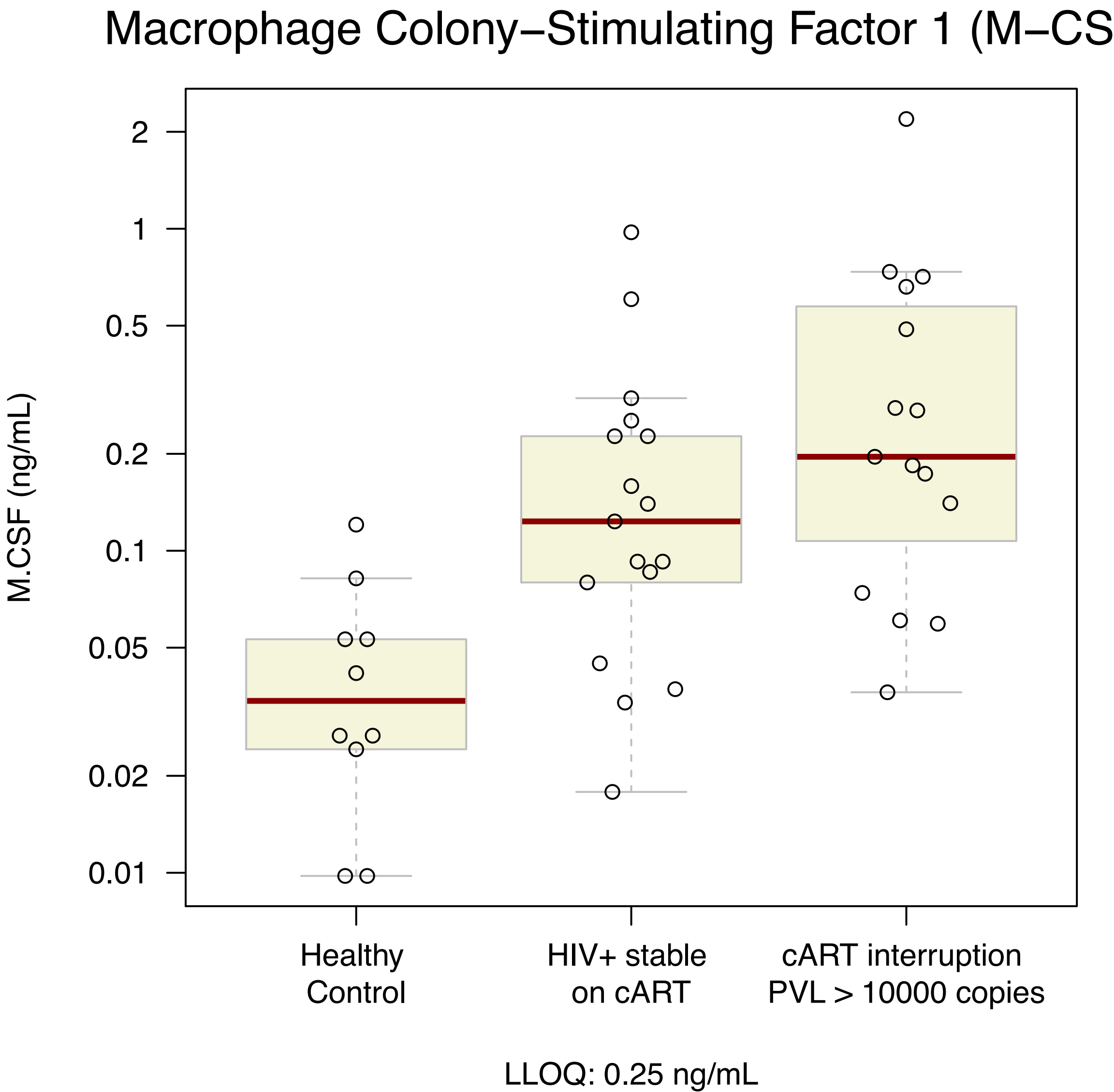

Macrophage Colony–Stimulating Factor 1 (M–CS
M.CSF (ng/mL)
2
1
0.5
0.2
0.1
0.05
0.02
0.01
Healthy Control
HIV+ stable on cART
cART interruption PVL > 10000 copies
LLOQ: 0.25 ng/mL

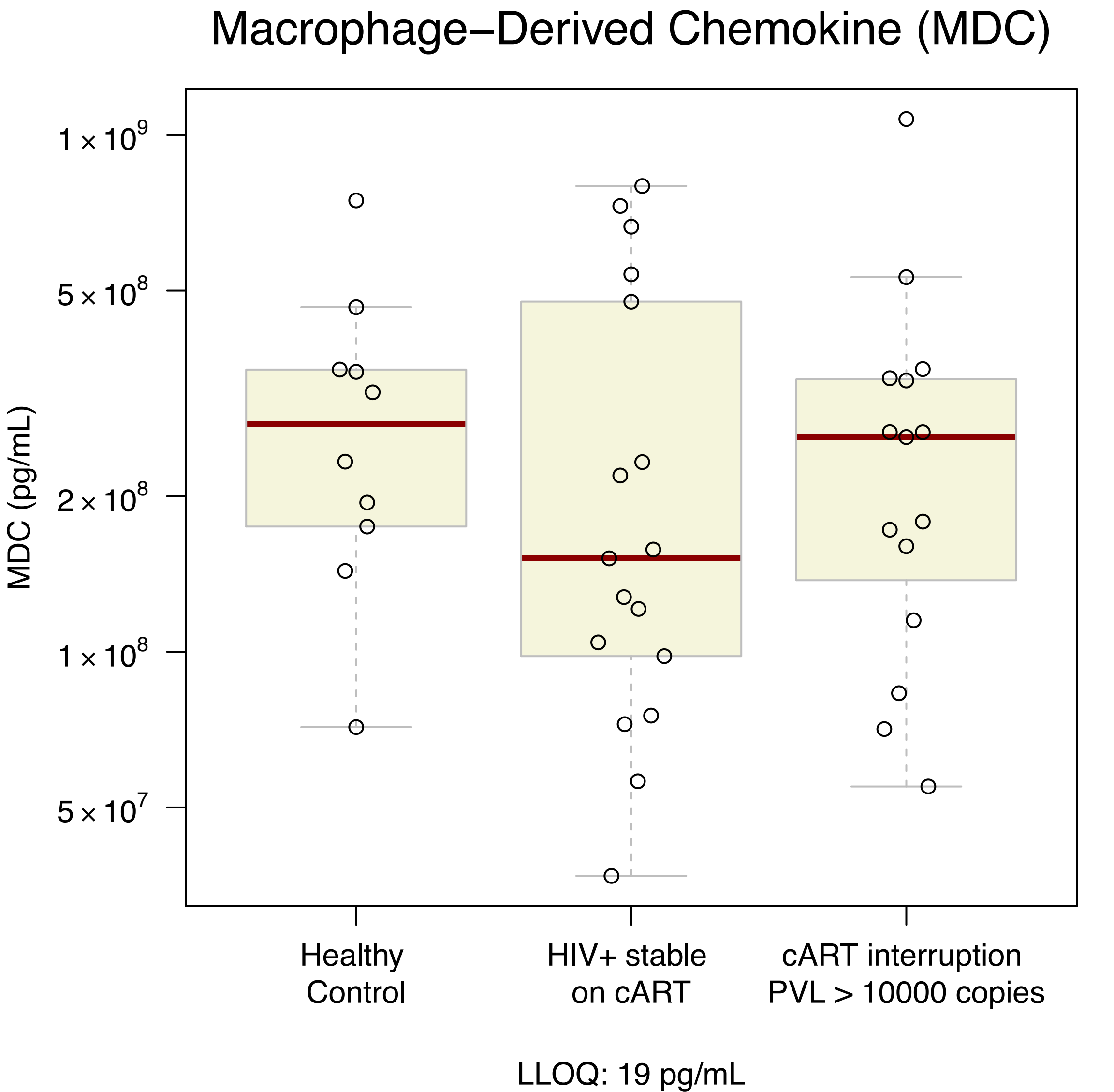
Macrophage−Derived Chemokine (MDC)
MDC (pg/mL)
1×10^9
5×10^8
2×10^8
1×10^8
5×10^7
Healthy Control
HIV+ stable on cART
cART interruption PVL > 10000 copies
LLOQ: 19 pg/mL

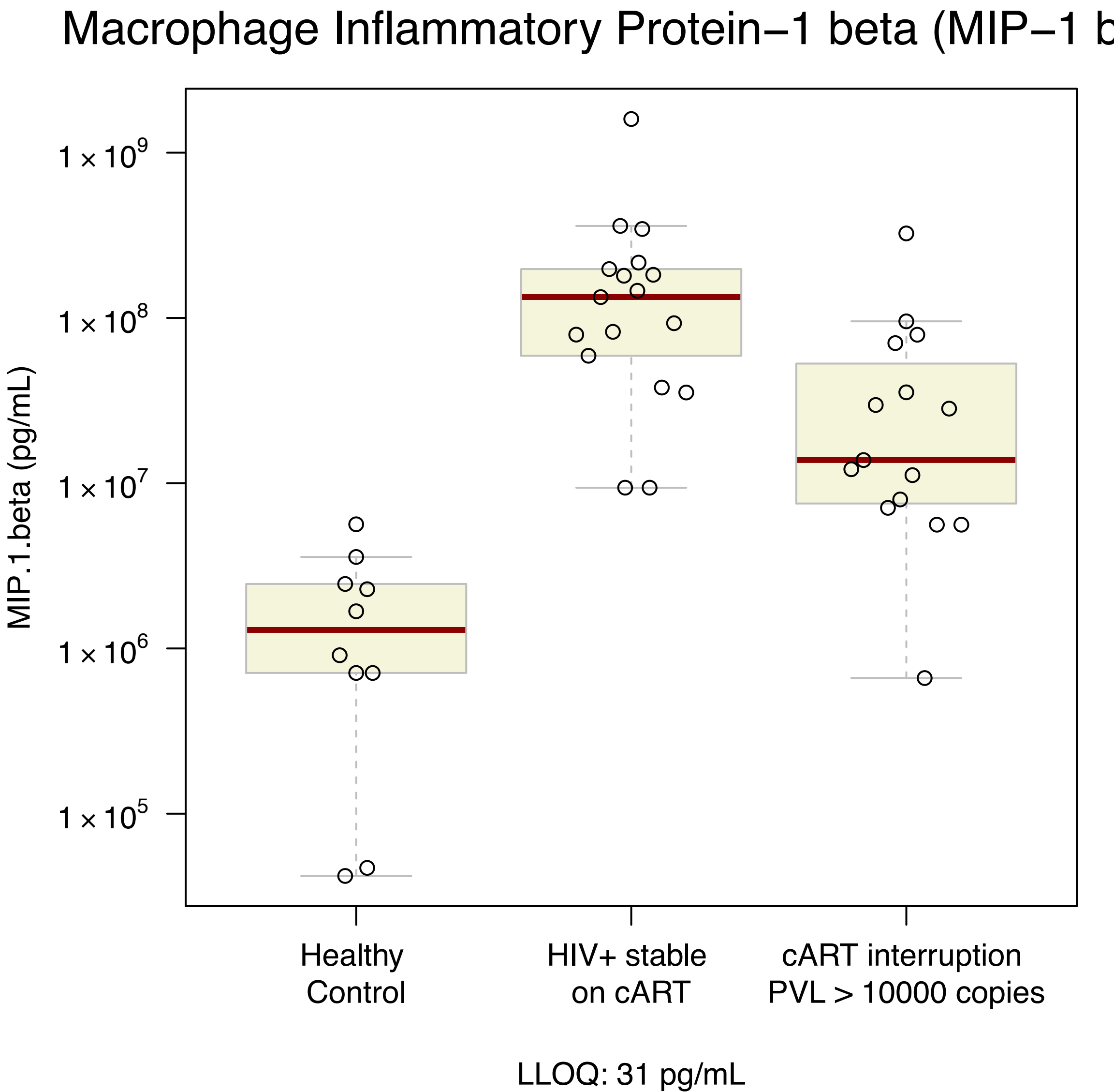

Macrophage Inflammatory Protein−1 beta (MIP−1 b
MIP.1.beta (pg/mL)
1 × 10^9
1 × 10^8
1 × 10^7
1 × 10^6
1 × 10^5
Healthy Control
HIV+ stable on cART
cART interruption PVL > 10000 copies
LLOQ: 31 pg/mL

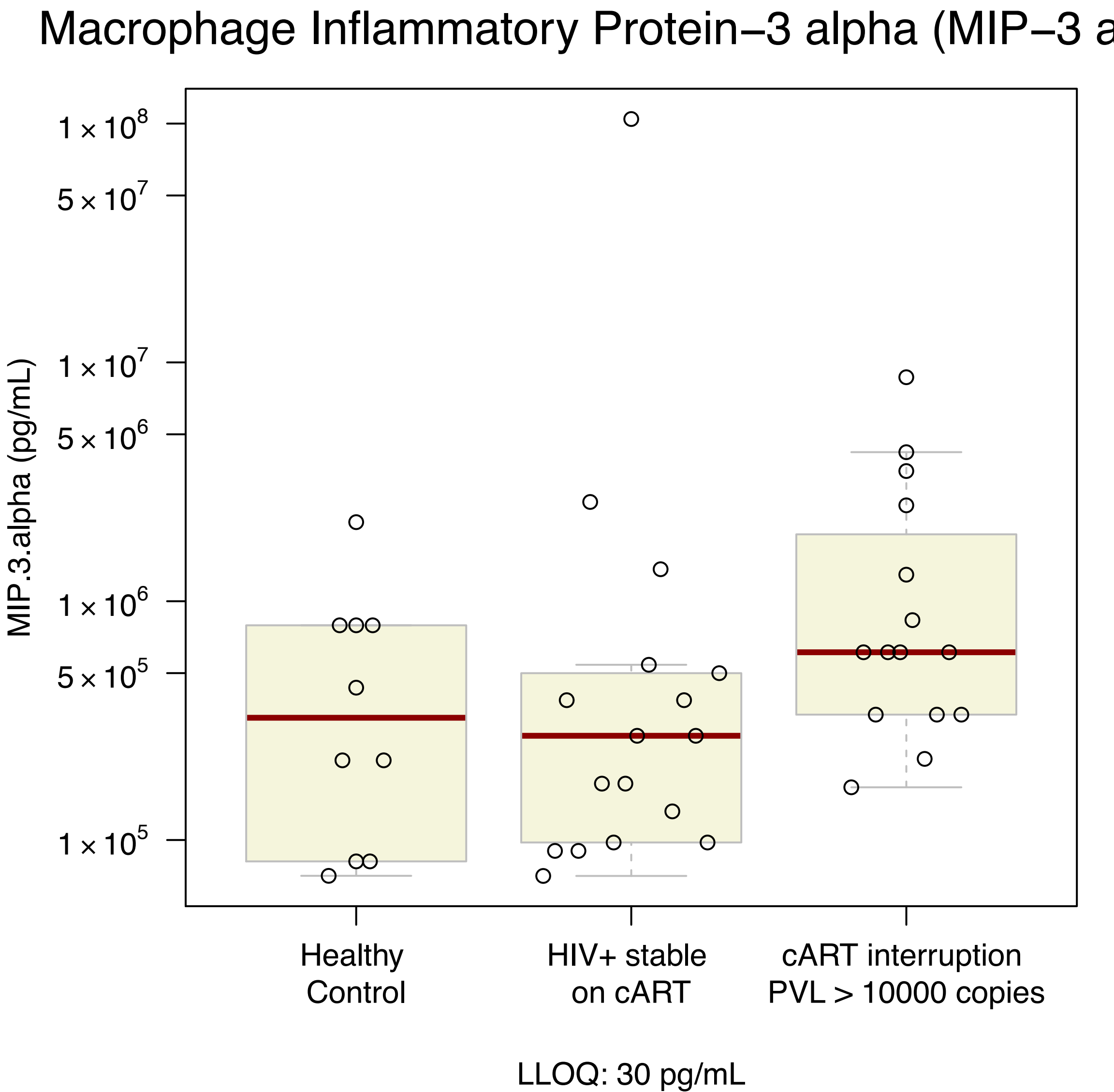

Macrophage Inflammatory Protein−3 alpha (MIP−3 a
MIP.3.alpha (pg/mL)
1 × 10^8
5 × 10^7
1 × 10^7
5 × 10^6
1 × 10^6
5 × 10^5
1 × 10^5
Healthy Control
HIV+ stable on cART
cART interruption PVL > 10000 copies
LLOQ: 30 pg/mL

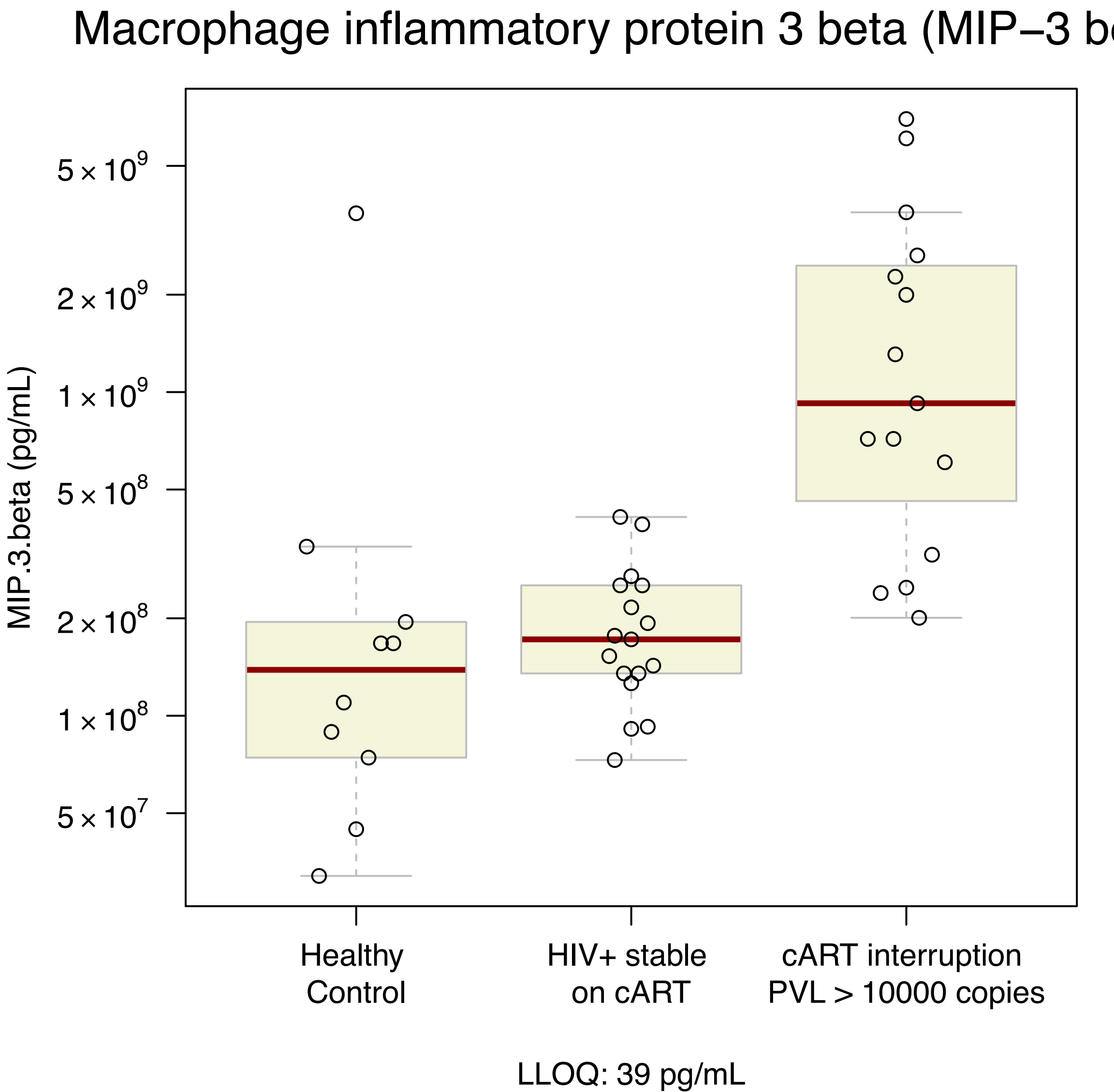

Macrophage inflammatory protein 3 beta (MIP−3 be
MIP.3.beta (pg/mL)
5 × 10^9
2 × 10^9
1 × 10^9
5 × 10^8
2 × 10^8
1 × 10^8
5 × 10^7
Healthy Control
HIV+ stable on cART
cART interruption PVL > 10000 copies
LLOQ: 39 pg/mL

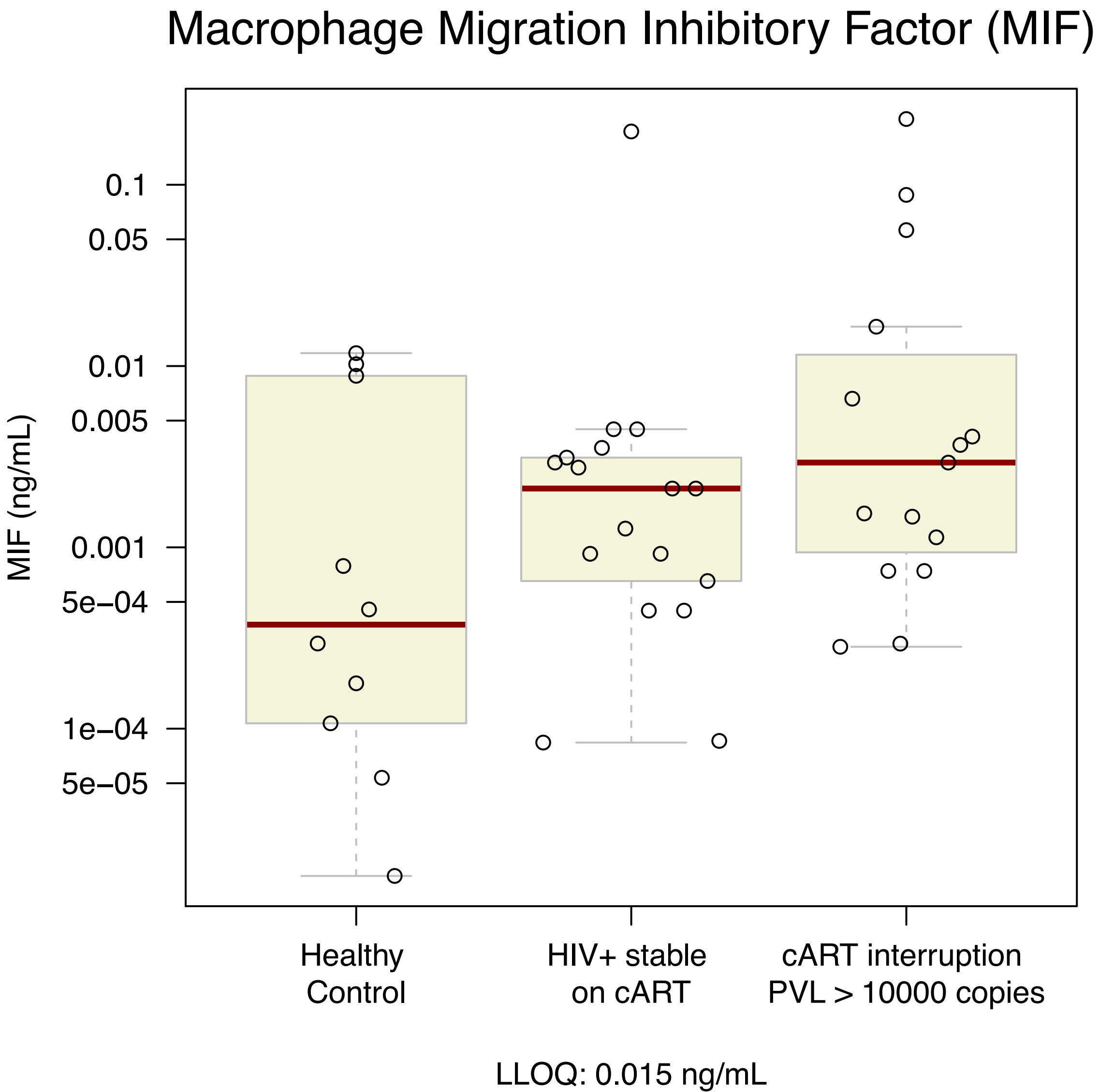

Macrophage Migration Inhibitory Factor (MIF)
MIF (ng/mL)
0.1
0.05
0.01
0.005
0.001
5e−04
1e−04
5e−05
Healthy Control
HIV+ stable on cART
cART interruption PVL > 10000 copies
LLOQ: 0.015 ng/mL

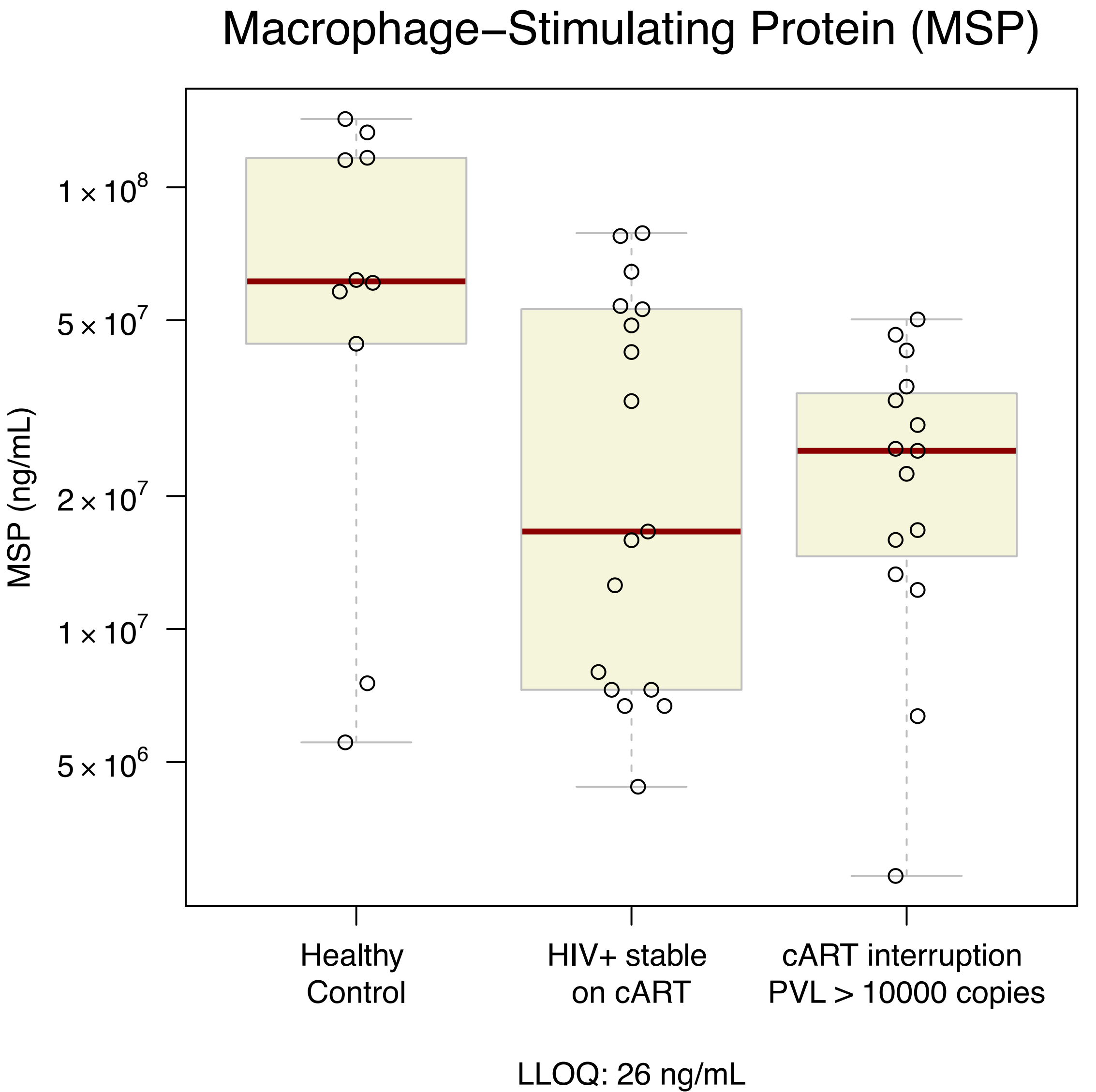
Macrophage–Stimulating Protein (MSP)
MSP (ng/mL)
1 × 10^8
5 × 10^7
2 × 10^7
1 × 10^7
5 × 10^6
Healthy Control
HIV+ stable on cART
cART interruption PVL > 10000 copies
LLOQ: 26 ng/mL

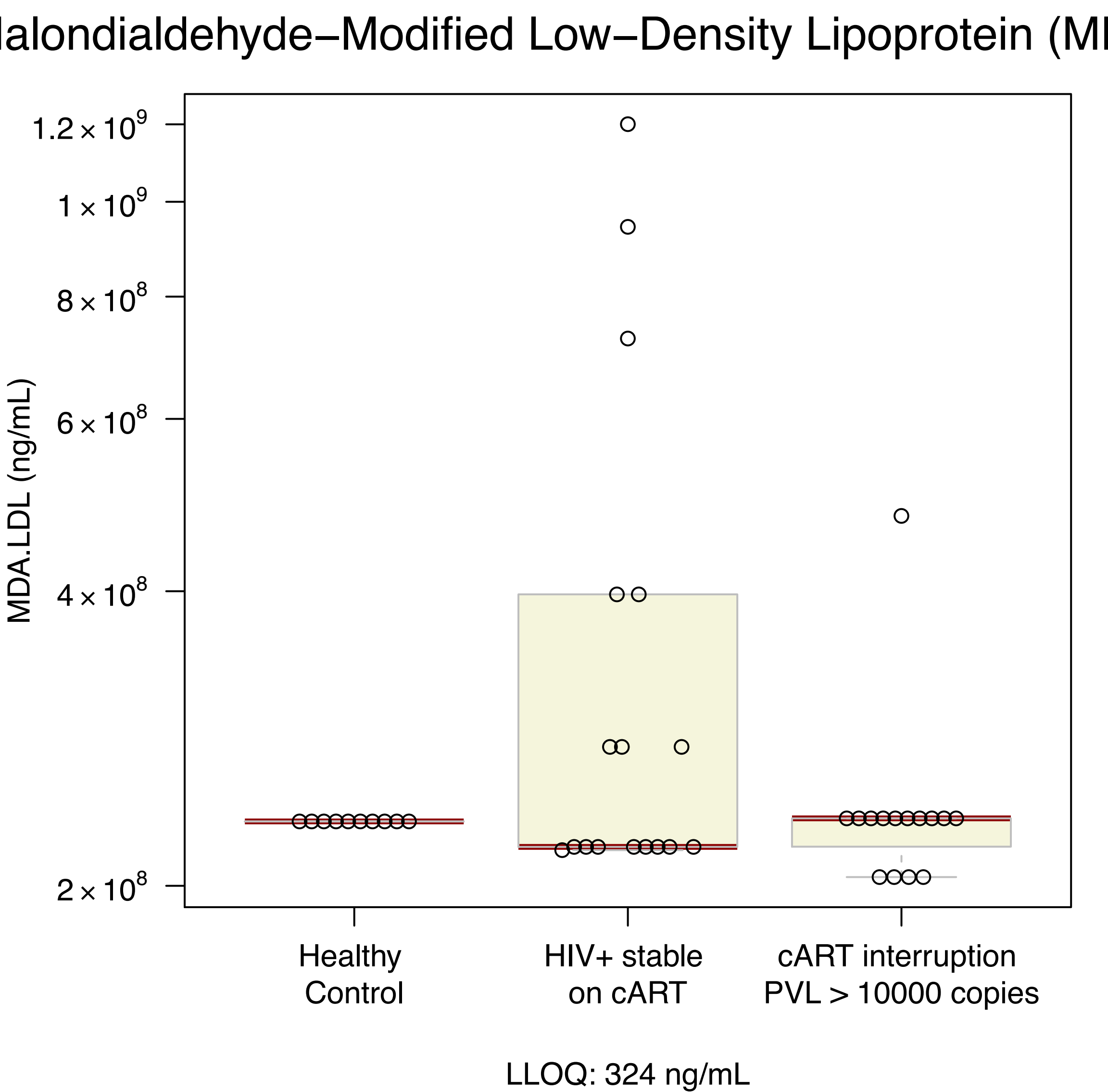

alondialdehyde−Modified Low−Density Lipoprotein (MI
MDA.LDL (ng/mL)
1.2 × 10^9
1 × 10^9
8 × 10^8
6 × 10^8
4 × 10^8
2 × 10^8
Healthy Control
HIV+ stable on cART
cART interruption PVL > 10000 copies
LLOQ: 324 ng/mL

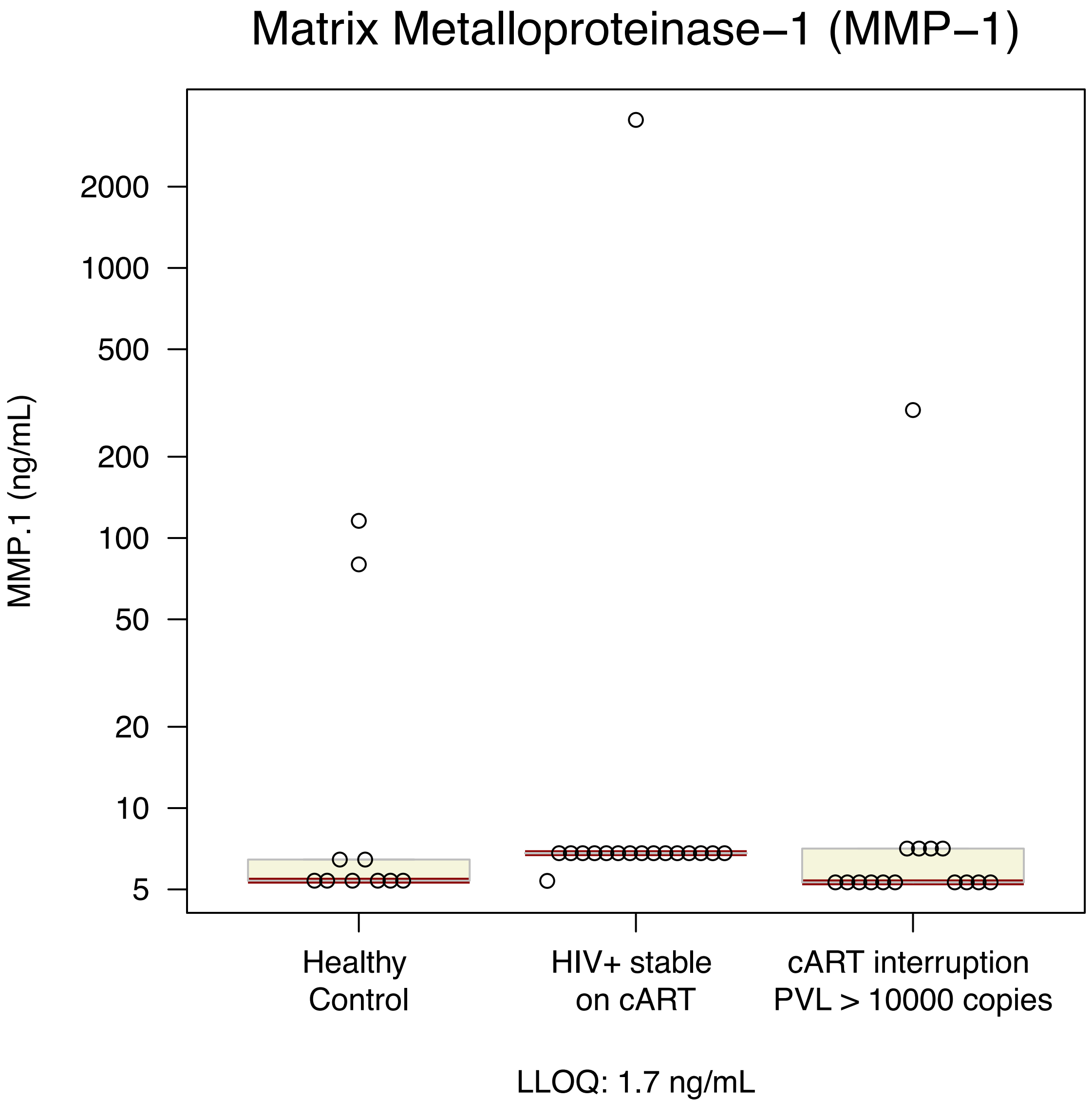
Matrix Metalloproteinase−1 (MMP−1)
MMP.1 (ng/mL)
2000
1000
500
200
100
50
20
10
5
Healthy Control
HIV+ stable on cART
cART interruption PVL > 10000 copies
LLOQ: 1.7 ng/mL

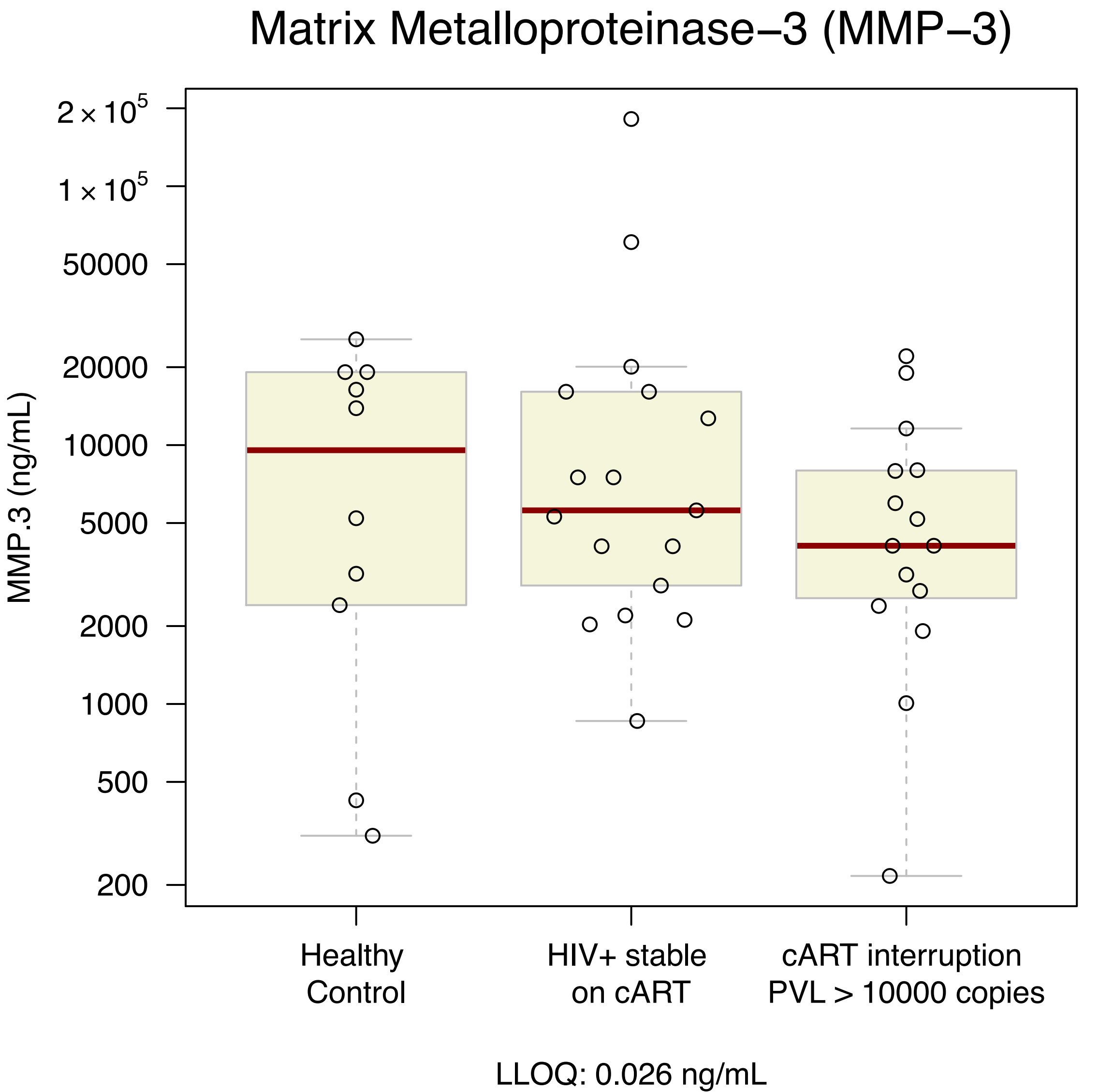
Matrix Metalloproteinase−3 (MMP−3)
MMP.3 (ng/mL)
2×10^5
1×10^5
50000
20000
10000
5000
2000
1000
500
200
Healthy Control
HIV+ stable on cART
cART interruption PVL > 10000 copies
LLOQ: 0.026 ng/mL

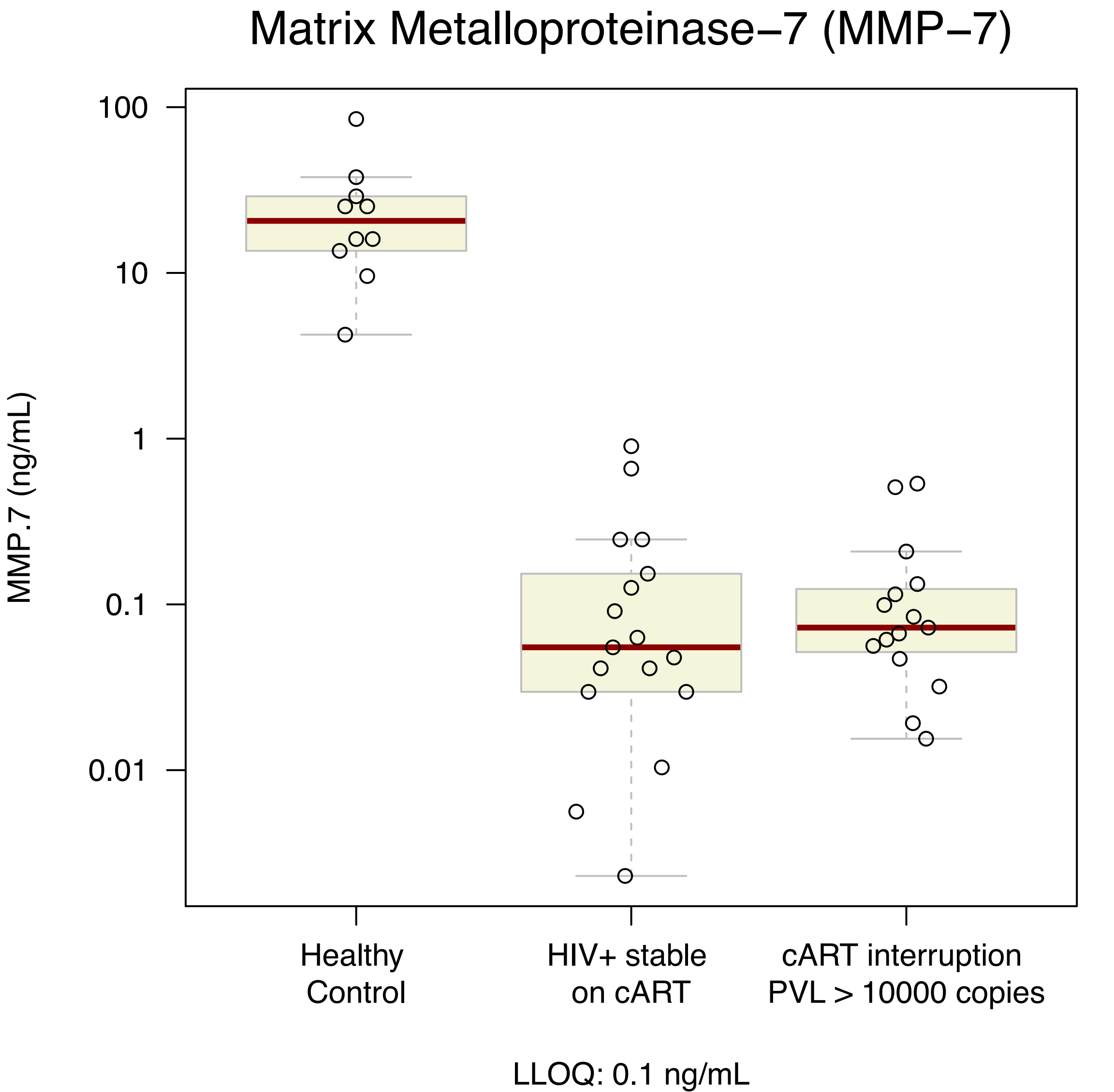
Matrix Metalloproteinase−7 (MMP−7)
MMP.7 (ng/mL)
100
10
1
0.1
0.01
Healthy Control
HIV+ stable on cART
cART interruption PVL > 10000 copies
LLOQ: 0.1 ng/mL

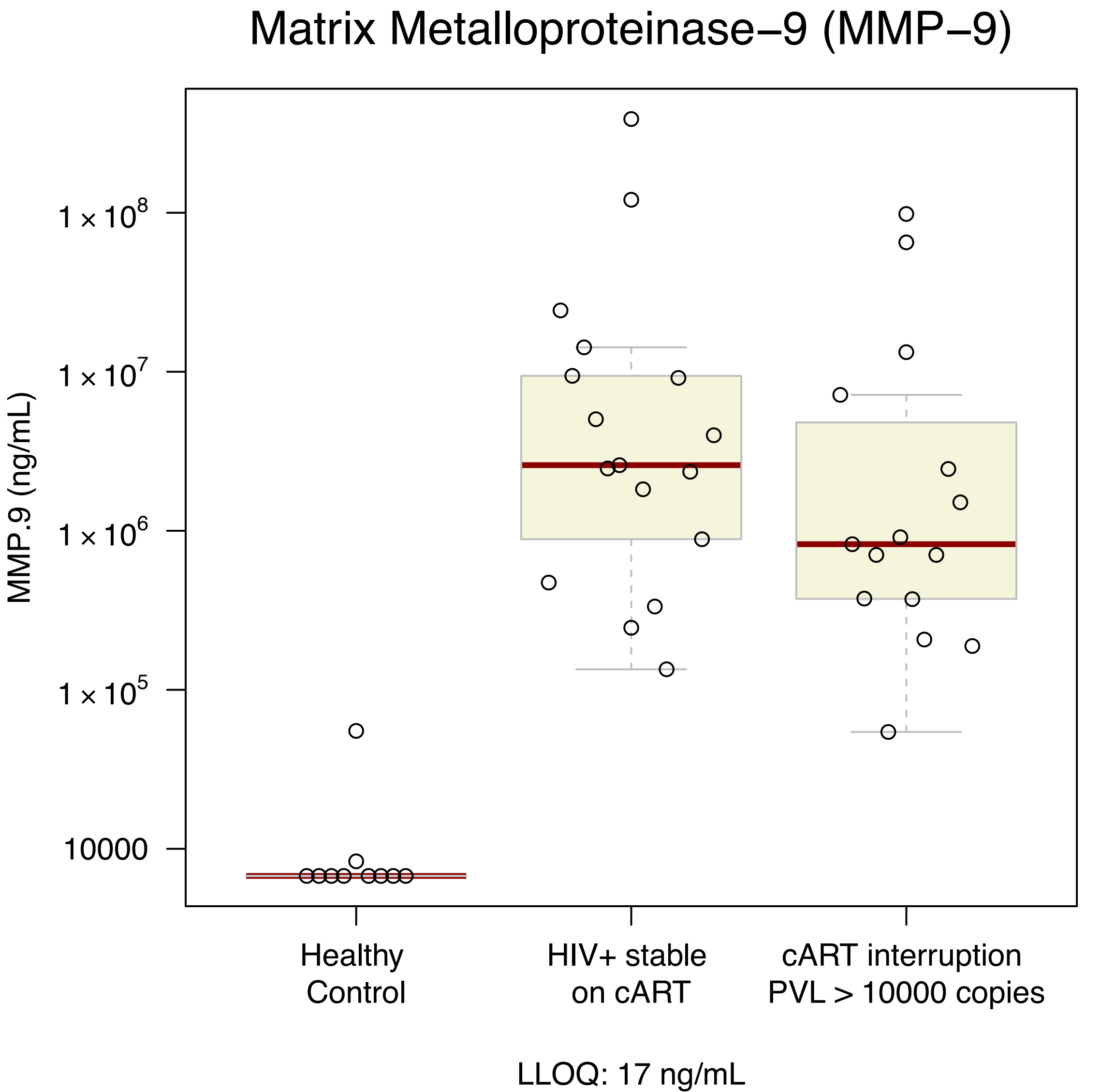

Matrix Metalloproteinase−9 (MMP−9)
MMP.9 (ng/mL)
1×10^8
1×10^7
1×10^6
1×10^5
10000
Healthy Control
HIV+ stable on cART
cART interruption PVL > 10000 copies
LLOQ: 17 ng/mL

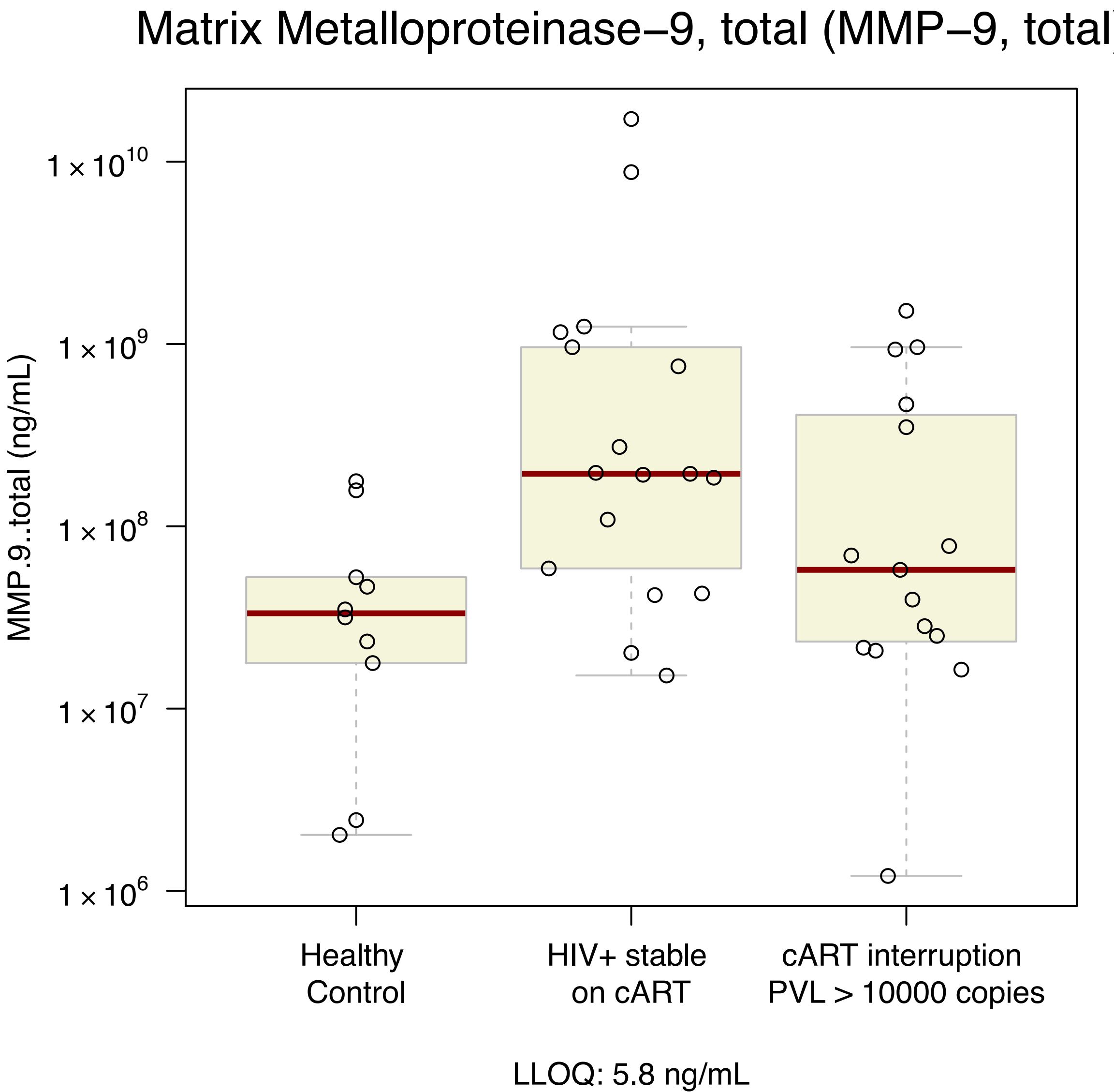

Matrix Metalloproteinase−9, total (MMP−9, total)
MMP.9..total (ng/mL)
1 × 10^10
1 × 10^9
1 × 10^8
1 × 10^7
1 × 10^6
Healthy Control
HIV+ stable on cART
cART interruption PVL > 10000 copies
LLOQ: 5.8 ng/mL

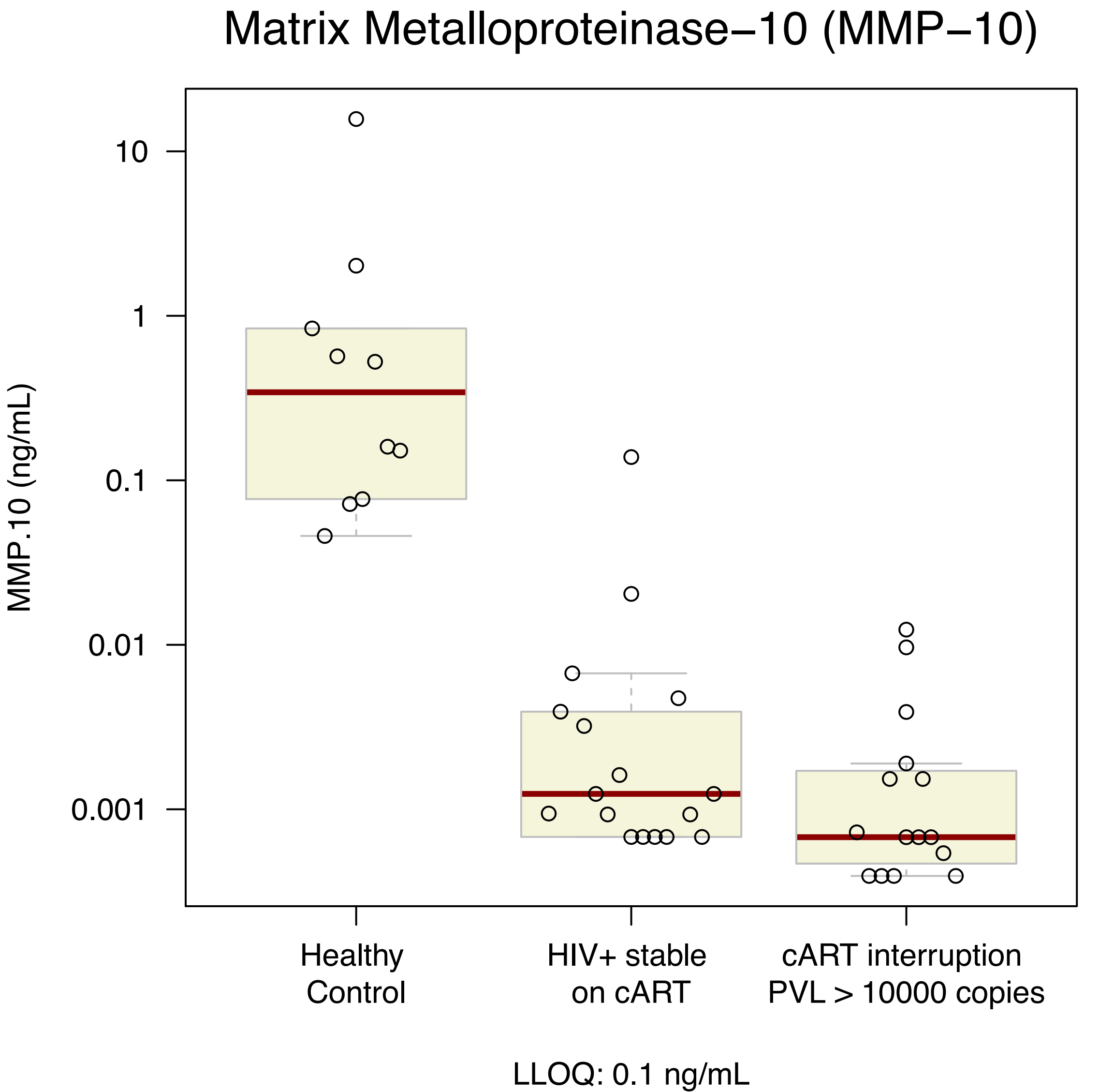

Matrix Metalloproteinase−10 (MMP−10)
MMP.10 (ng/mL)
10
1
0.1
0.01
0.001
Healthy Control
HIV+ stable on cART
cART interruption PVL > 10000 copies
LLOQ: 0.1 ng/mL

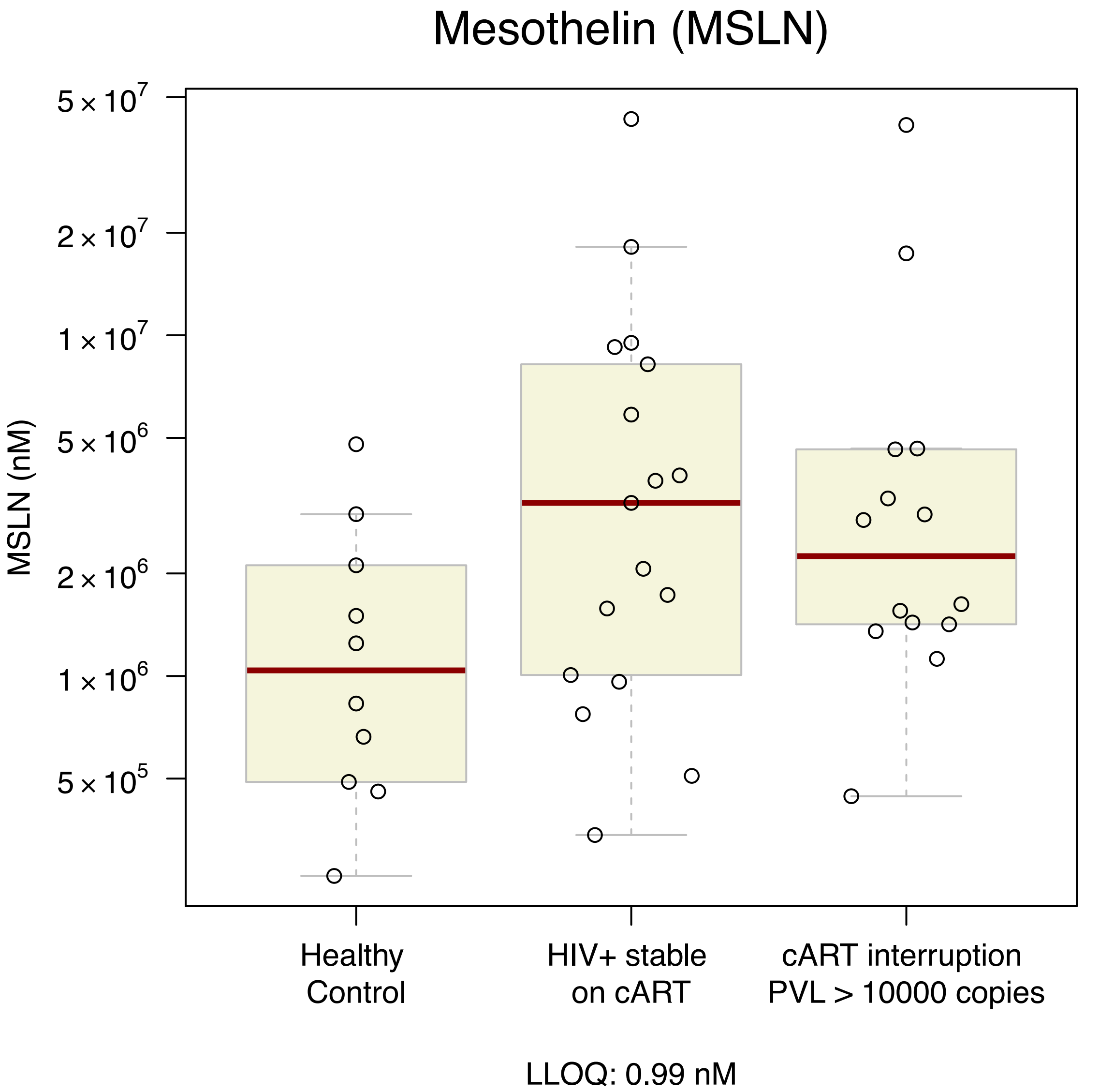

Mesothelin (MSLN)
MSLN (nM)
5 × 10^7
2 × 10^7
1 × 10^7
5 × 10^6
2 × 10^6
1 × 10^6
5 × 10^5
Healthy Control
HIV+ stable on cART
cART interruption PVL > 10000 copies
LLOQ: 0.99 nM

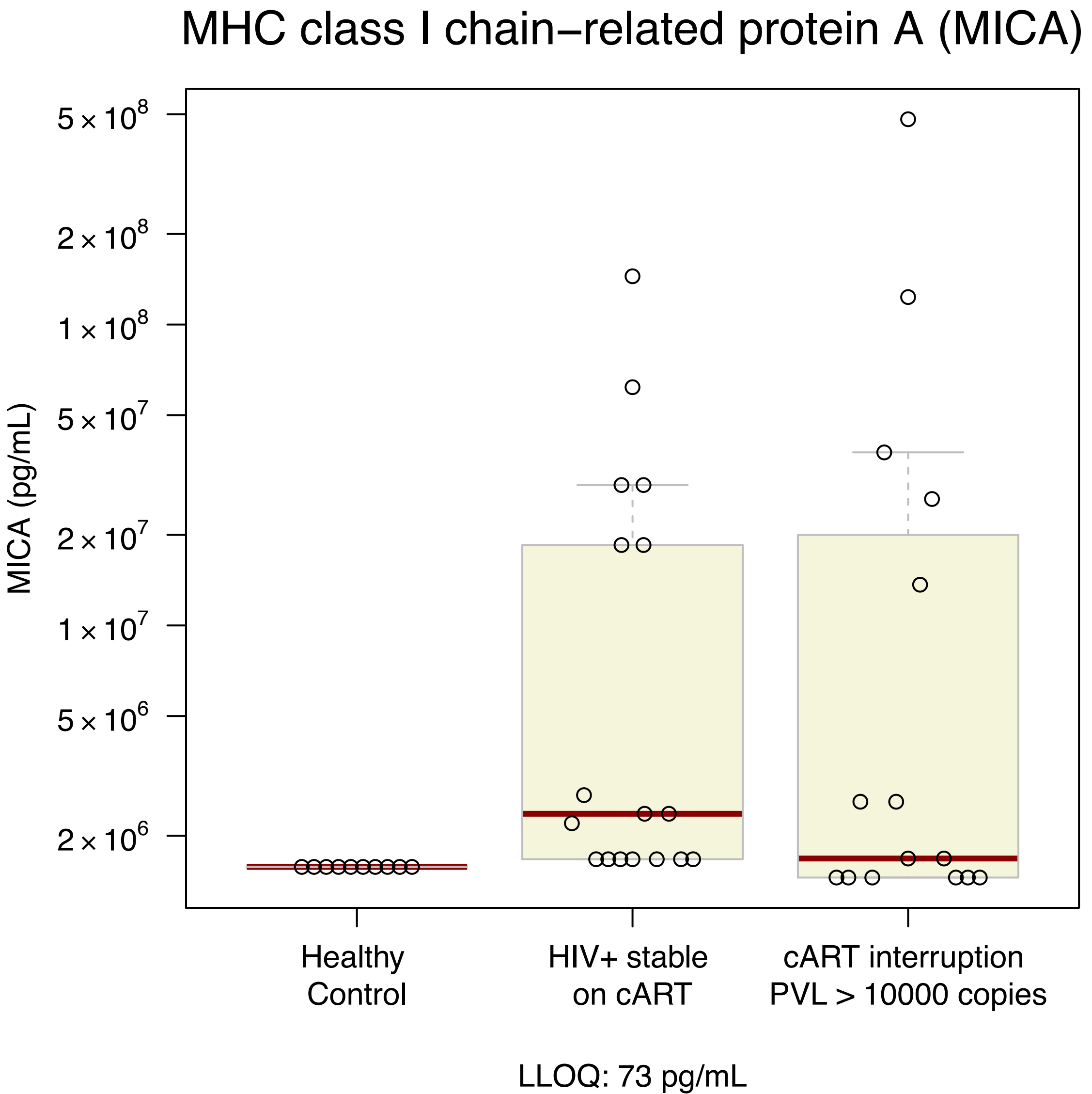
MHC class I chain−related protein A (MICA)
MICA (pg/mL)
5 × 10^8
2 × 10^8
1 × 10^8
5 × 10^7
2 × 10^7
1 × 10^7
5 × 10^6
2 × 10^6
Healthy Control
HIV+ stable on cART
cART interruption PVL > 10000 copies
LLOQ: 73 pg/mL

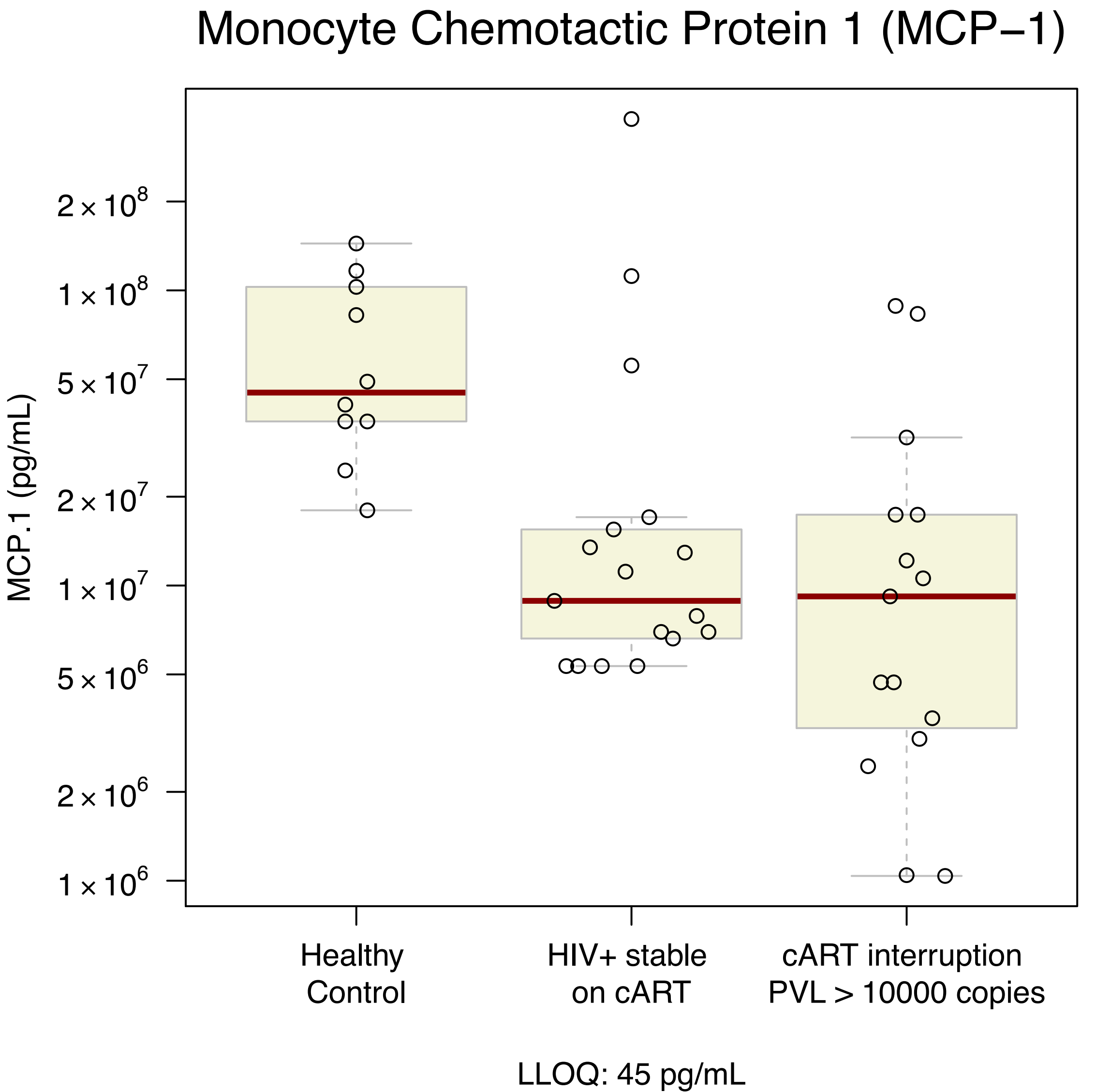

Monocyte Chemotactic Protein 1 (MCP−1)
MCP.1 (pg/mL)
2 × 10^8
1 × 10^8
5 × 10^7
2 × 10^7
1 × 10^7
5 × 10^6
2 × 10^6
1 × 10^6
Healthy Control
HIV+ stable on cART
cART interruption PVL > 10000 copies
LLOQ: 45 pg/mL

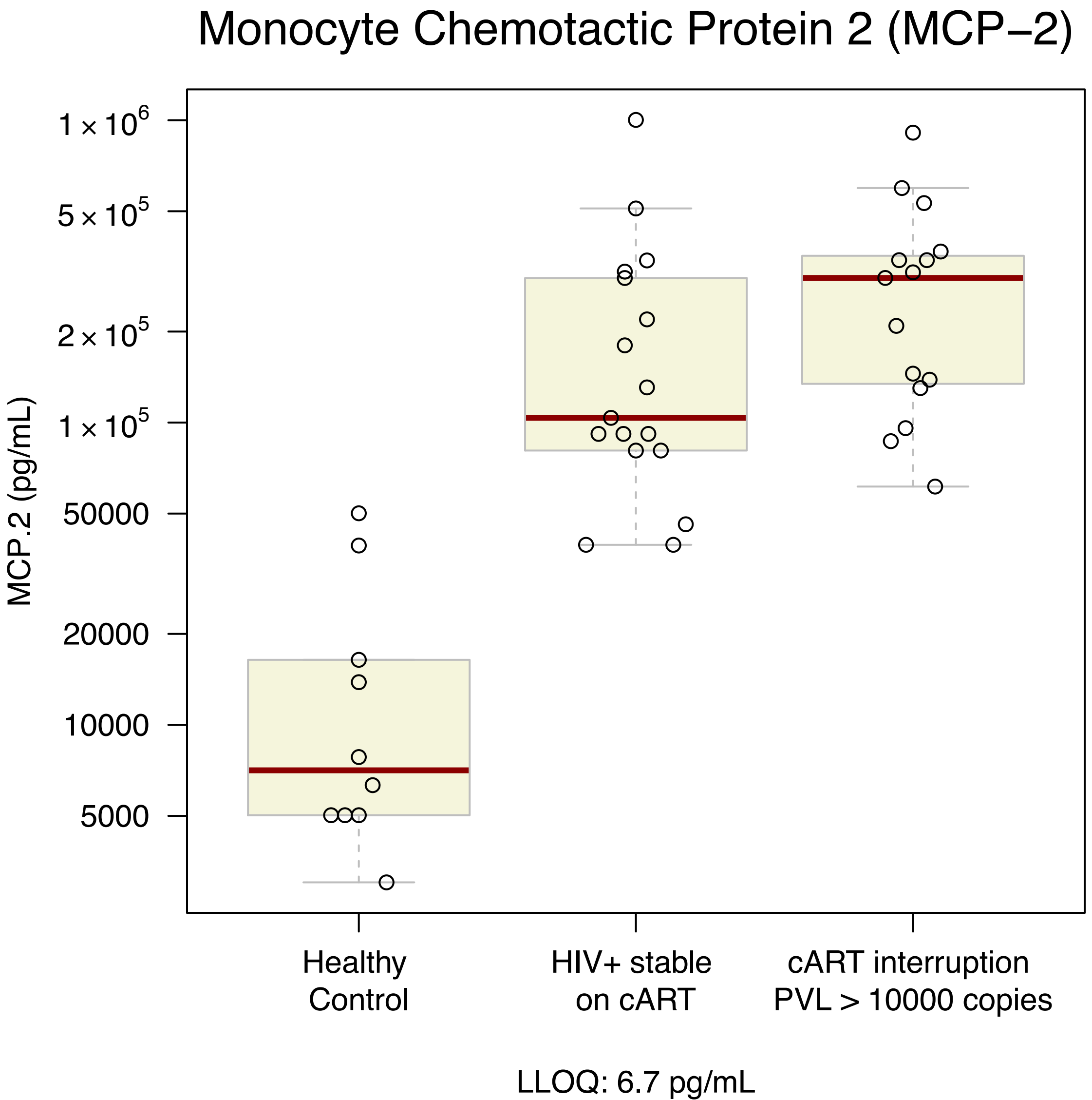
Monocyte Chemotactic Protein 2 (MCP–2)
MCP.2 (pg/mL)
1×10^6
5×10^5
2×10^5
1×10^5
50000
20000
10000
5000
Healthy Control
HIV+ stable on cART
cART interruption PVL > 10000 copies
LLOQ: 6.7 pg/mL

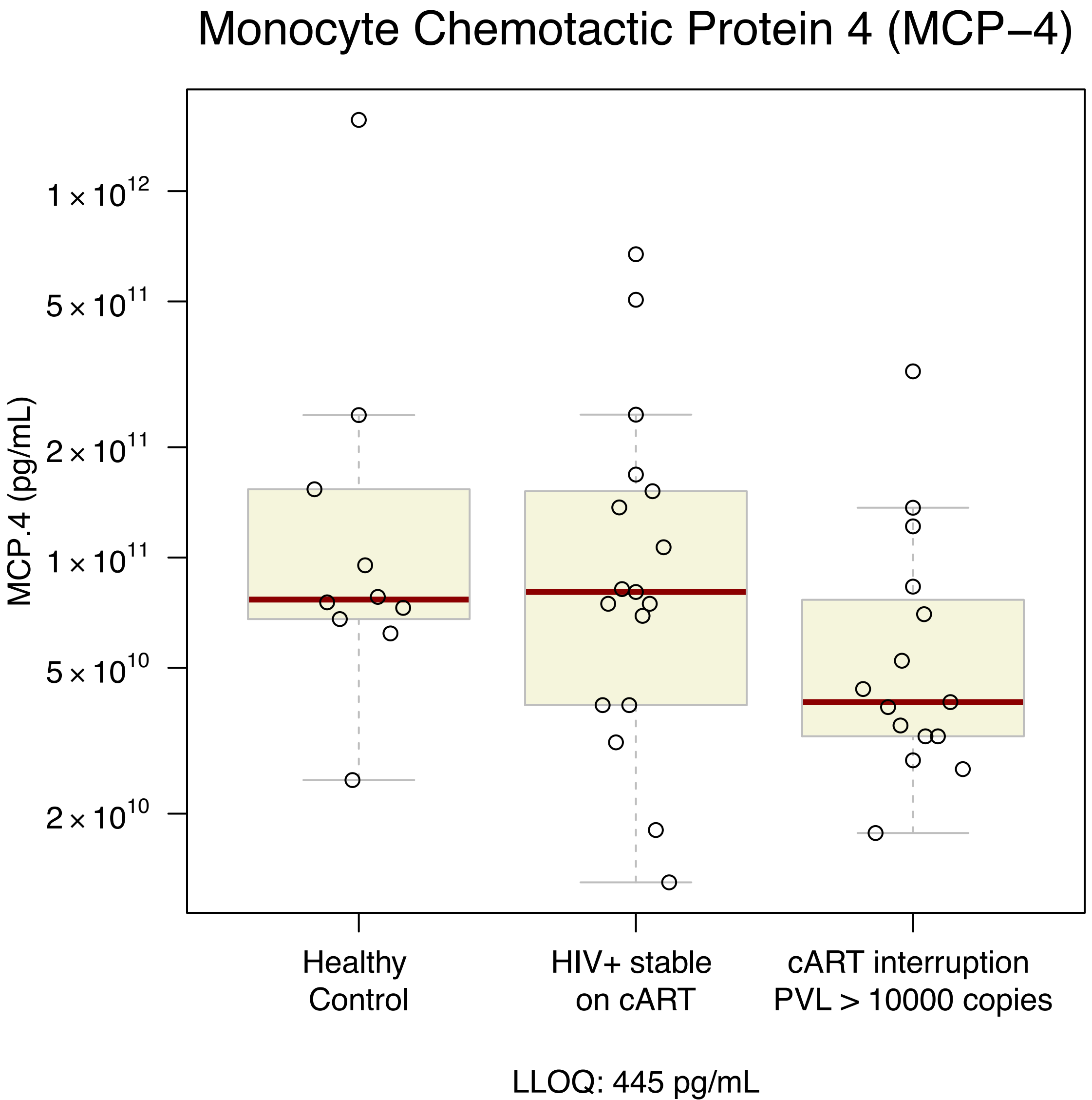
Monocyte Chemotactic Protein 4 (MCP−4)
MCP.4 (pg/mL)
1×10^{12}
5×10^{11}
2×10^{11}
1×10^{11}
5×10^{10}
2×10^{10}
Healthy Control
HIV+ stable on cART
cART interruption PVL > 10000 copies
LLOQ: 445 pg/mL

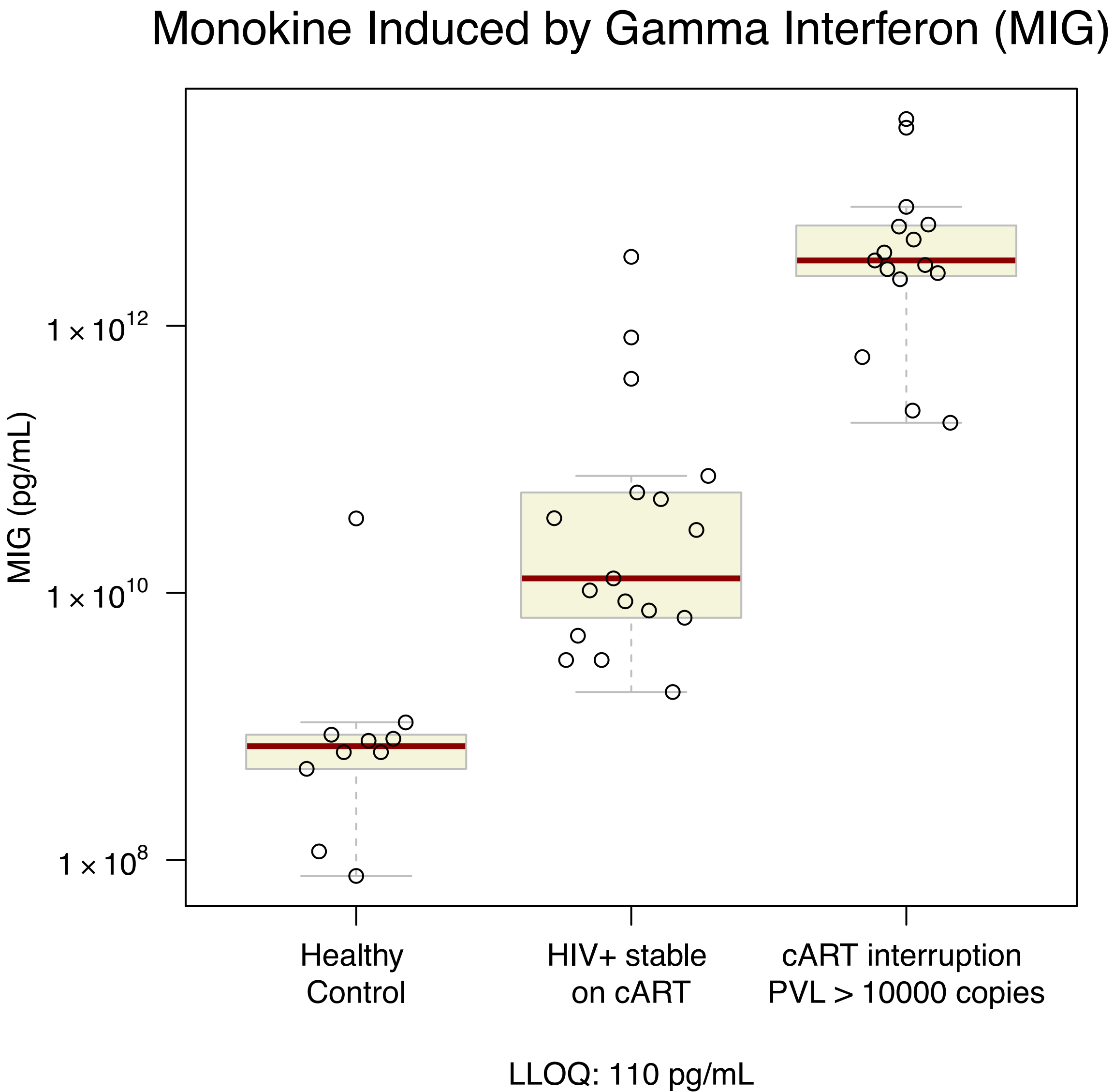

Monokine Induced by Gamma Interferon (MIG)
MIG (pg/mL)
1×10^{12}
1×10^{10}
1×10^{8}
Healthy Control
HIV+ stable on cART
cART interruption PVL > 10000 copies
LLOQ: 110 pg/mL

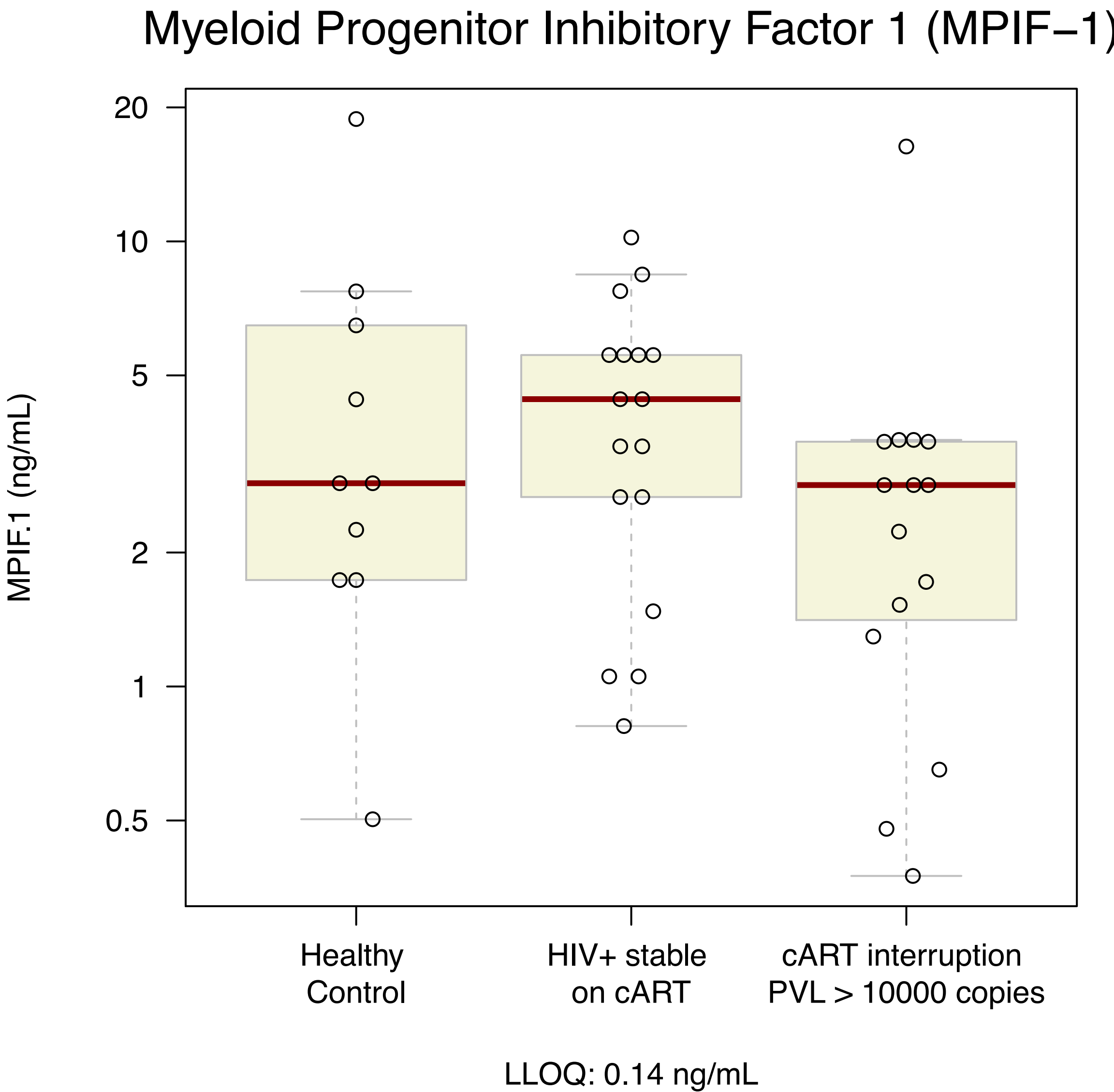
Myeloid Progenitor Inhibitory Factor 1 (MPIF−1)
MPIF.1 (ng/mL)
20
10
5
2
1
0.5
Healthy Control
HIV+ stable on cART
cART interruption PVL > 10000 copies
LLOQ: 0.14 ng/mL

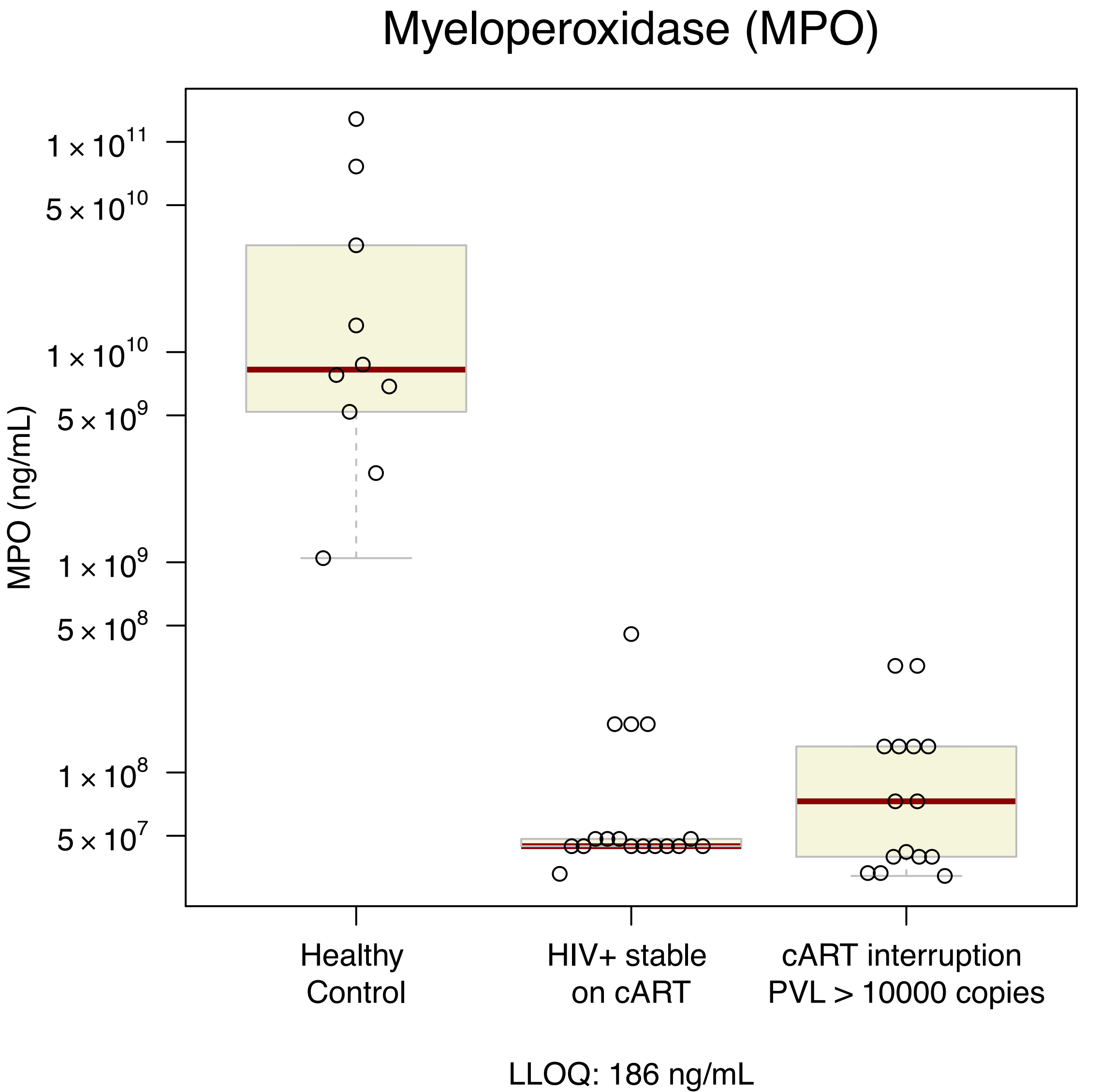
Myeloperoxidase (MPO)
MPO (ng/mL)
1×10^{11}
5×10^{10}
1×10^{10}
5×10^{9}
1×10^{9}
5×10^{8}
1×10^{8}
5×10^{7}
Healthy Control
HIV+ stable on cART
cART interruption PVL > 10000 copies
LLOQ: 186 ng/mL

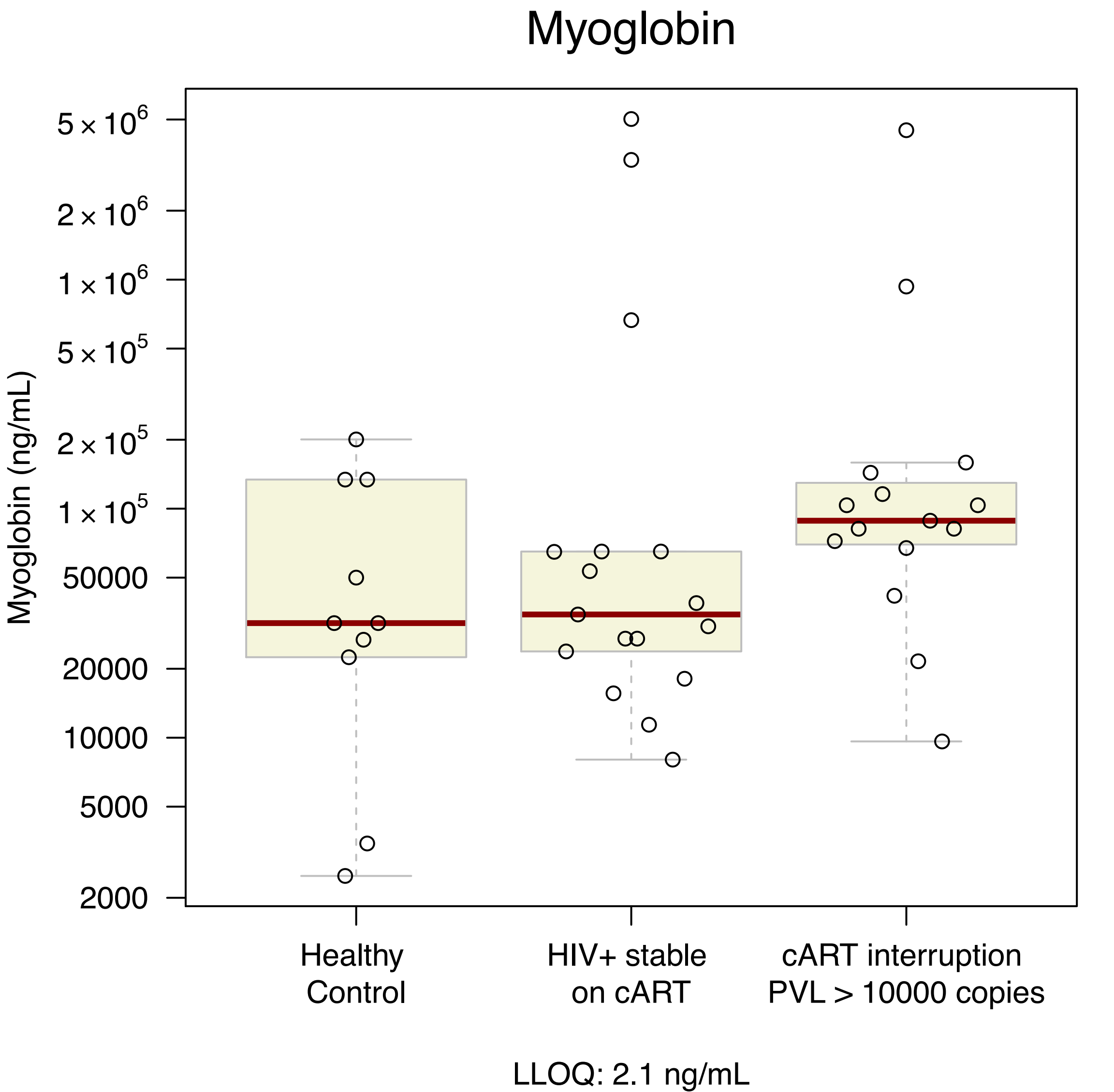

Myoglobin
Myoglobin (ng/mL)
5 × 10^6
2 × 10^6
1 × 10^6
5 × 10^5
2 × 10^5
1 × 10^5
50000
20000
10000
5000
2000
Healthy Control
HIV+ stable on cART
cART interruption PVL > 10000 copies
LLOQ: 2.1 ng/mL

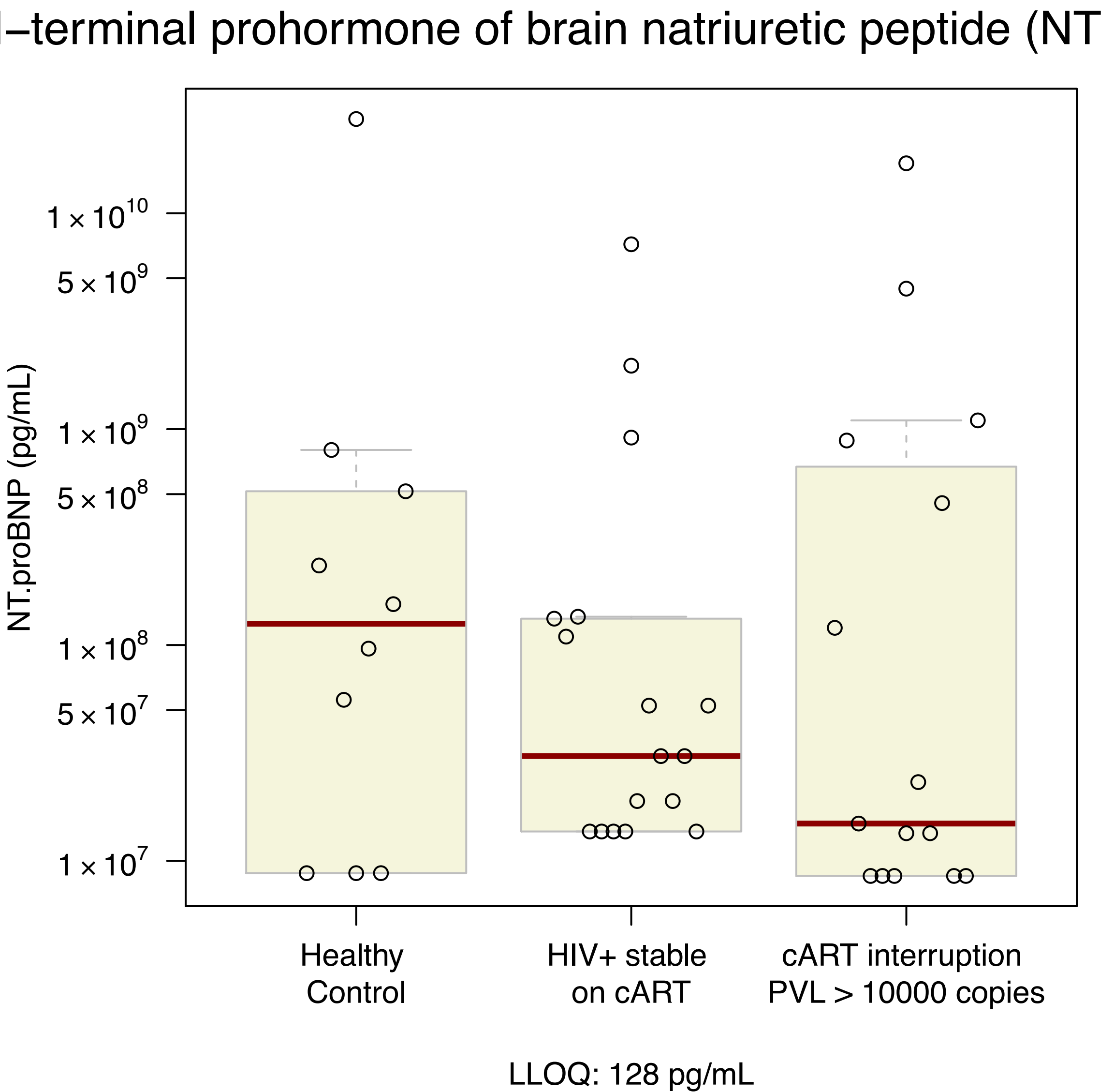

N–terminal prohormone of brain natriuretic peptide (NT
NT.proBNP (pg/mL)
1×10^{10}
5×10^{9}
1×10^{9}
5×10^{8}
1×10^{8}
5×10^{7}
1×10^{7}
Healthy Control
HIV+ stable on cART
cART interruption PVL > 10000 copies
LLOQ: 128 pg/mL

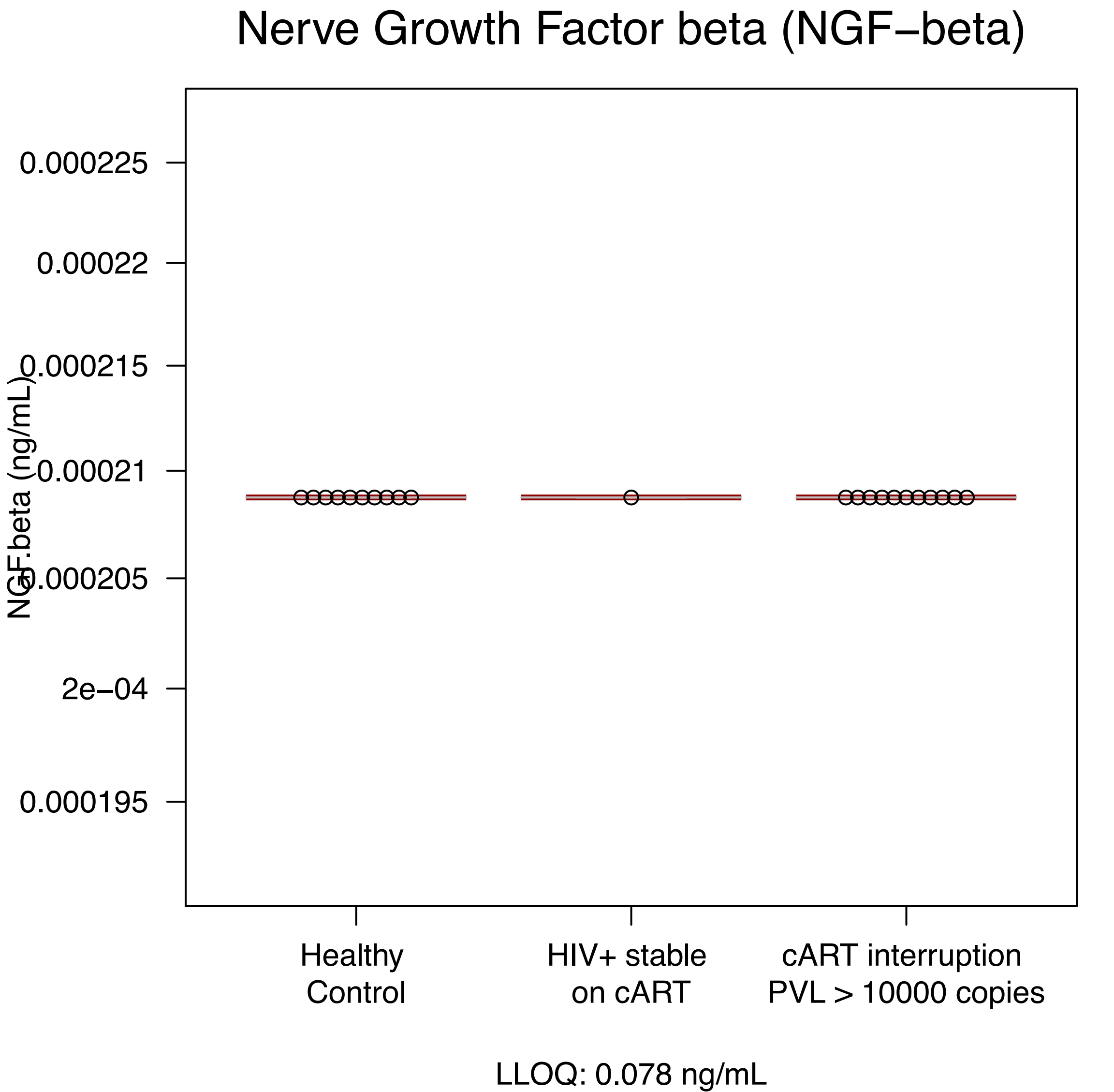
Nerve Growth Factor beta (NGF−beta)
NGF.beta (ng/mL)
0.000225
0.00022
0.000215
0.00021
0.000205
2e−04
0.000195
Healthy Control
HIV+ stable on cART
cART interruption PVL > 10000 copies
LLOQ: 0.078 ng/mL

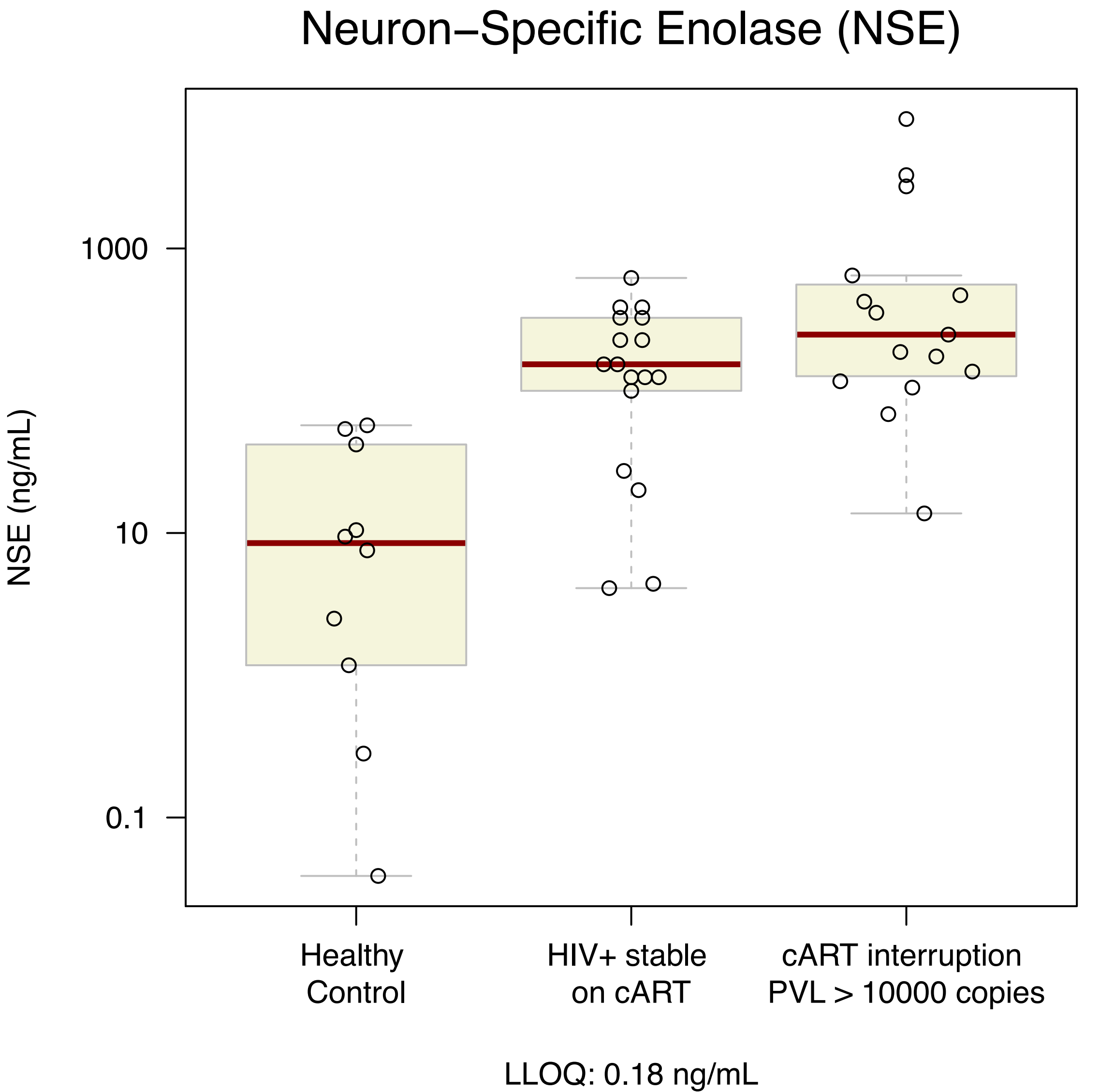

Neuron–Specific Enolase (NSE)
NSE (ng/mL)
1000
10
0.1
Healthy Control
HIV+ stable on cART
cART interruption PVL > 10000 copies
LLOQ: 0.18 ng/mL

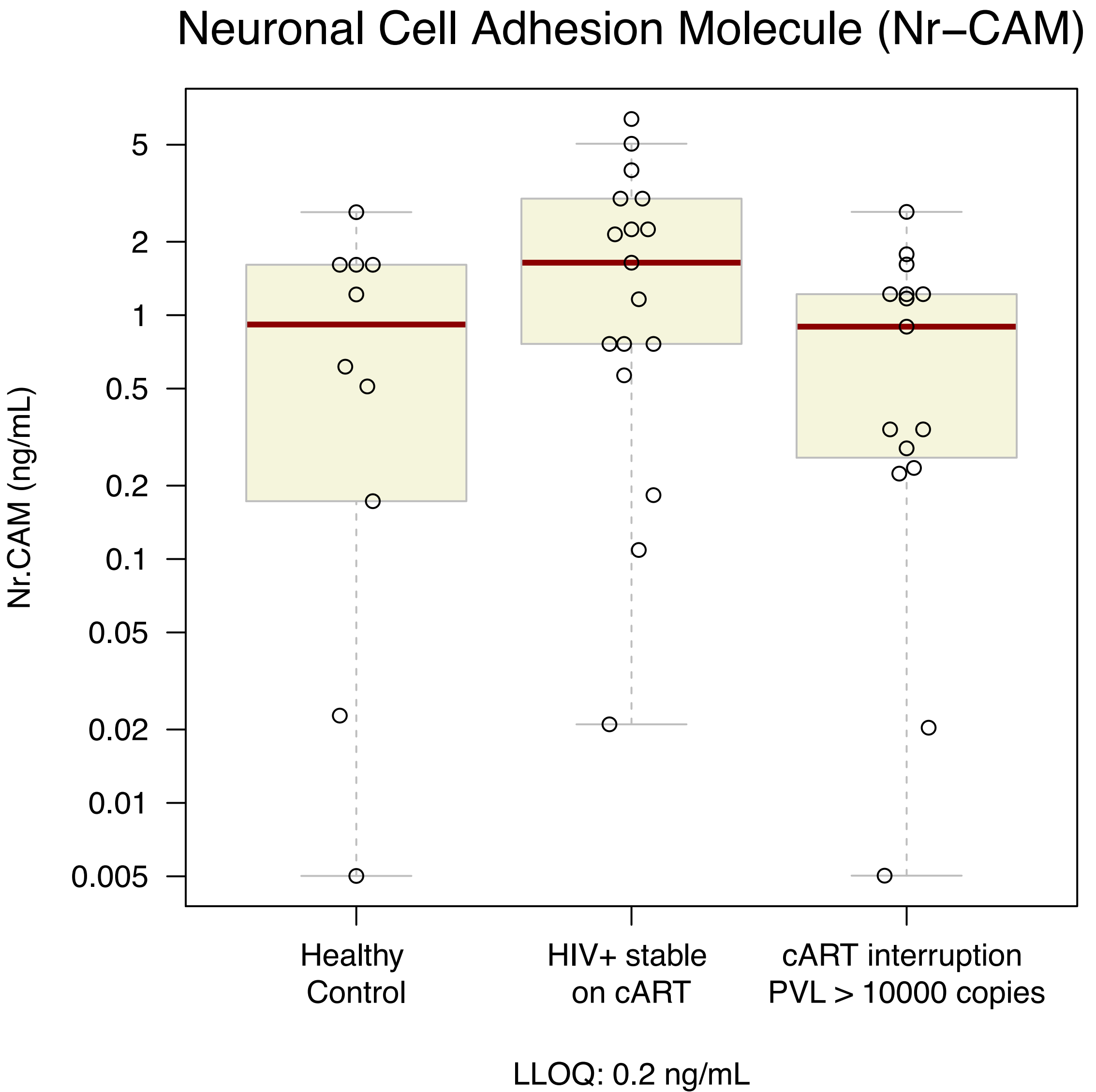

Neuronal Cell Adhesion Molecule (Nr−CAM)
Nr.CAM (ng/mL)
5
2
1
0.5
0.2
0.1
0.05
0.02
0.01
0.005
Healthy Control
HIV+ stable on cART
cART interruption PVL > 10000 copies
LLOQ: 0.2 ng/mL

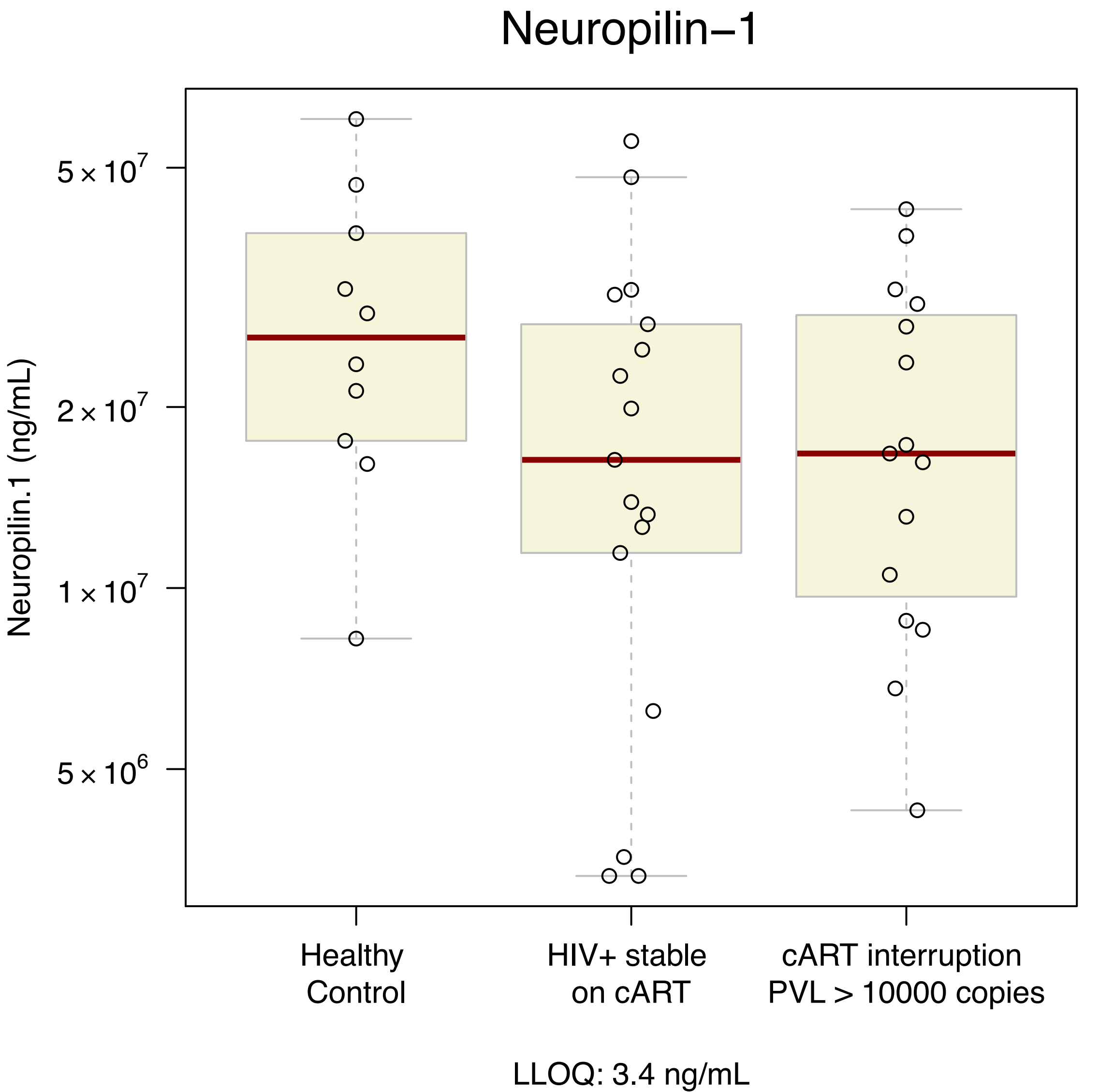

Neuropilin−1
Neuropilin.1 (ng/mL)
5 × 10^7
2 × 10^7
1 × 10^7
5 × 10^6
Healthy Control
HIV+ stable on cART
cART interruption PVL > 10000 copies
LLOQ: 3.4 ng/mL

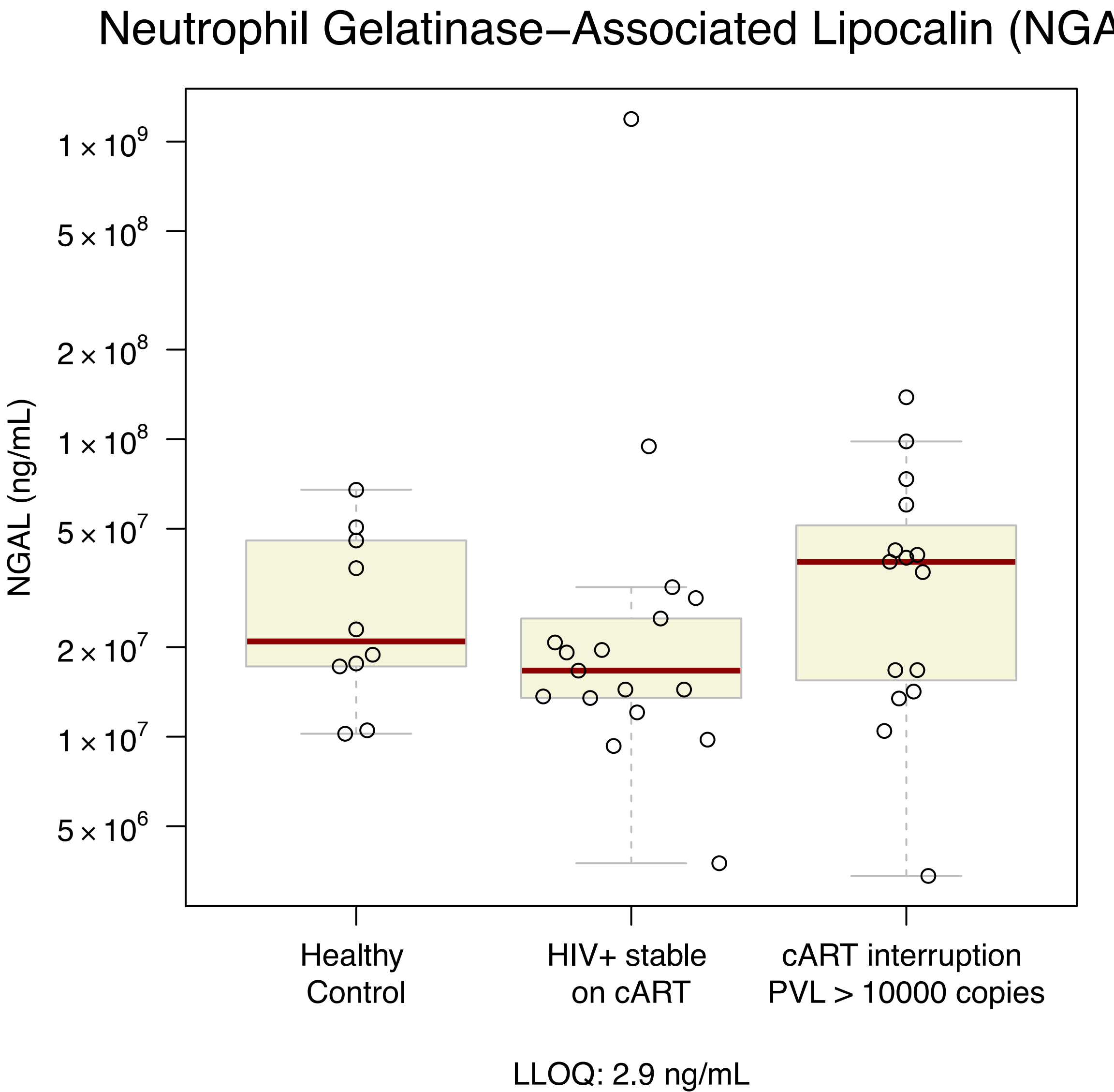

Neutrophil Gelatinase−Associated Lipocalin (NGA
NGAL (ng/mL)
1×10^9
5×10^8
2×10^8
1×10^8
5×10^7
2×10^7
1×10^7
5×10^6
Healthy Control
HIV+ stable on cART
cART interruption PVL > 10000 copies
LLOQ: 2.9 ng/mL

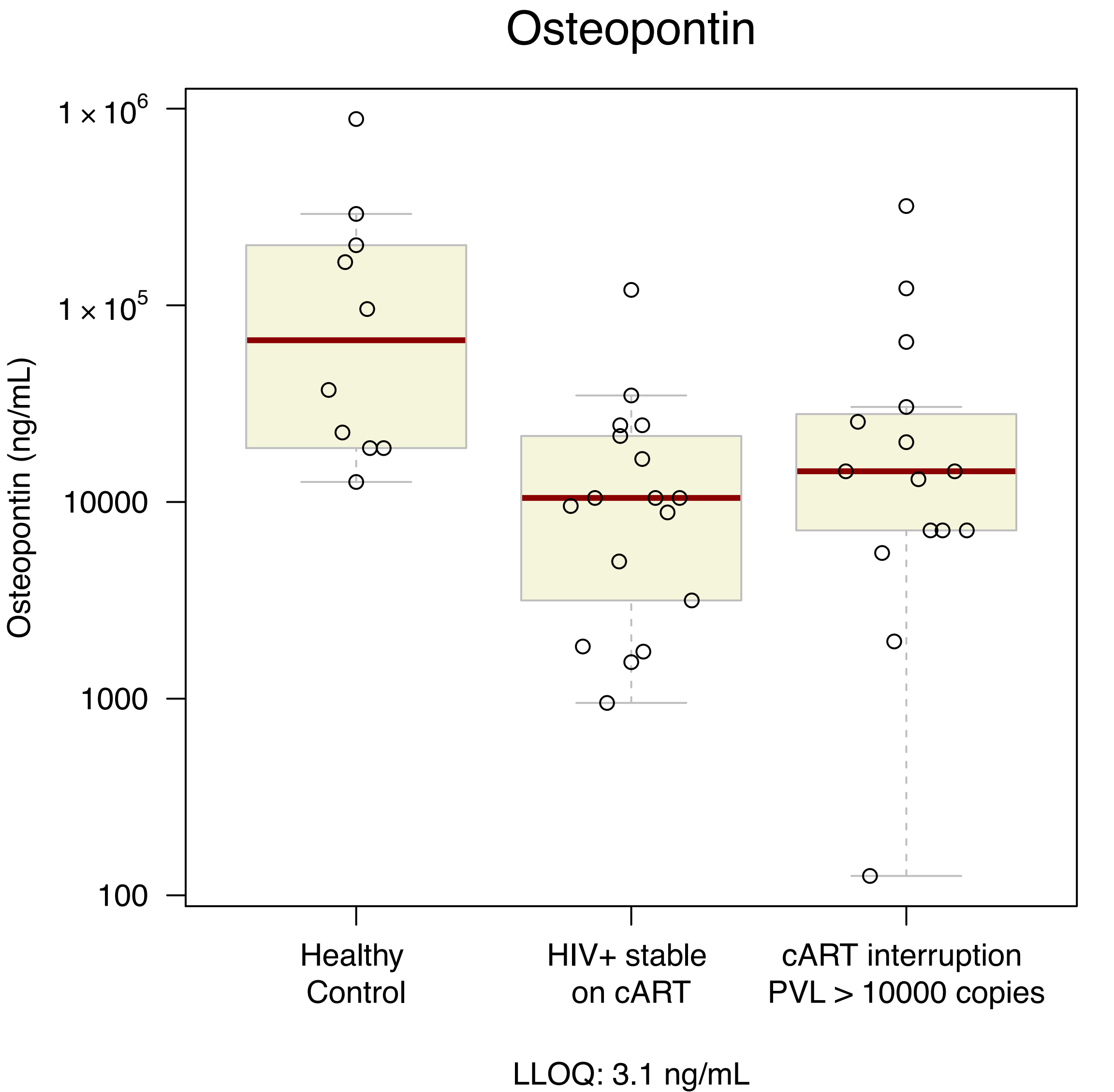
Osteopontin
Osteopontin (ng/mL)
1×10^6
1×10^5
10000
1000
100
Healthy Control
HIV+ stable on cART
cART interruption PVL > 10000 copies
LLOQ: 3.1 ng/mL

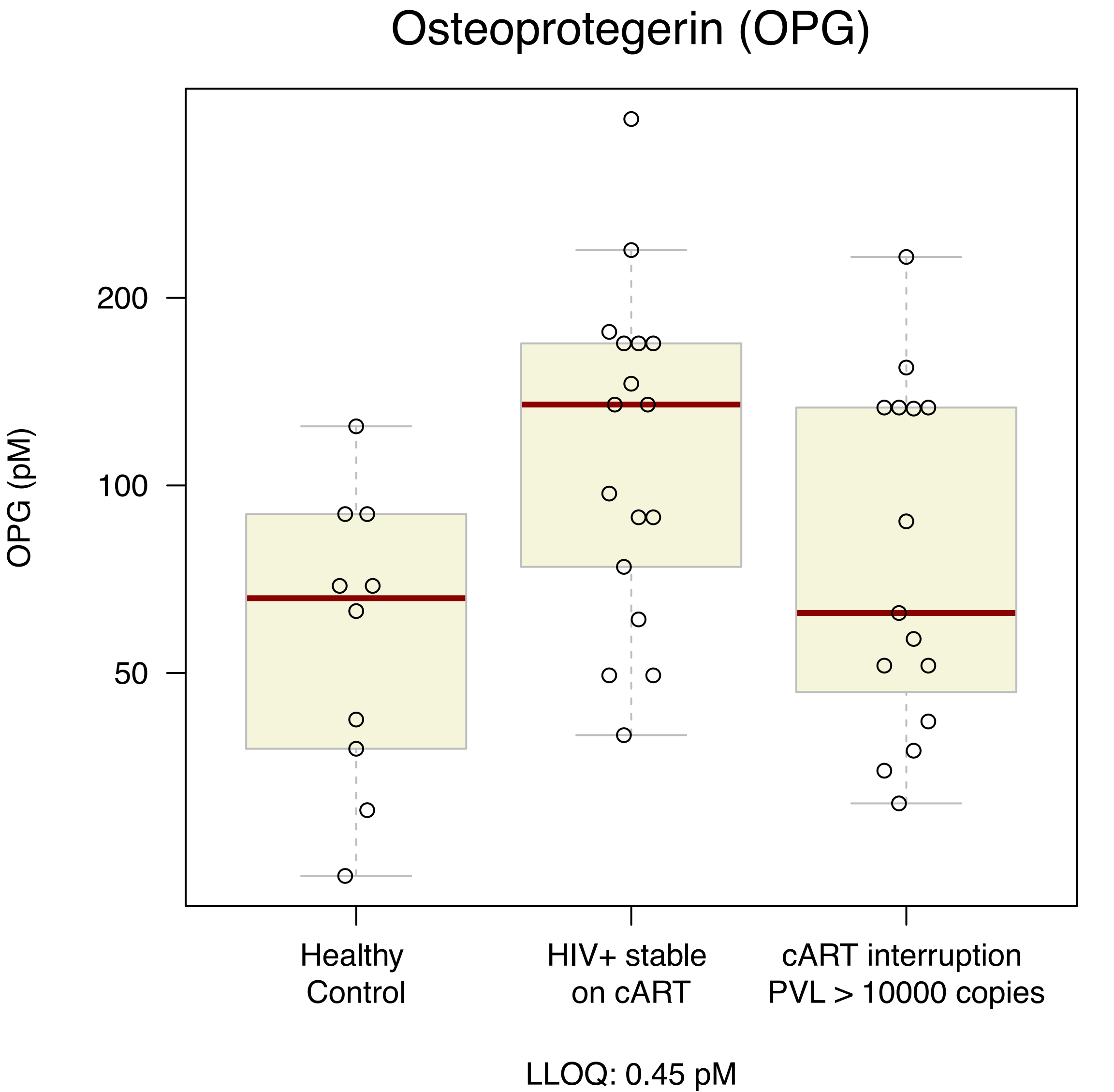
Osteoprotegerin (OPG)
OPG (pM)
200
100
50
Healthy Control
HIV+ stable on cART
cART interruption PVL > 10000 copies
LLOQ: 0.45 pM

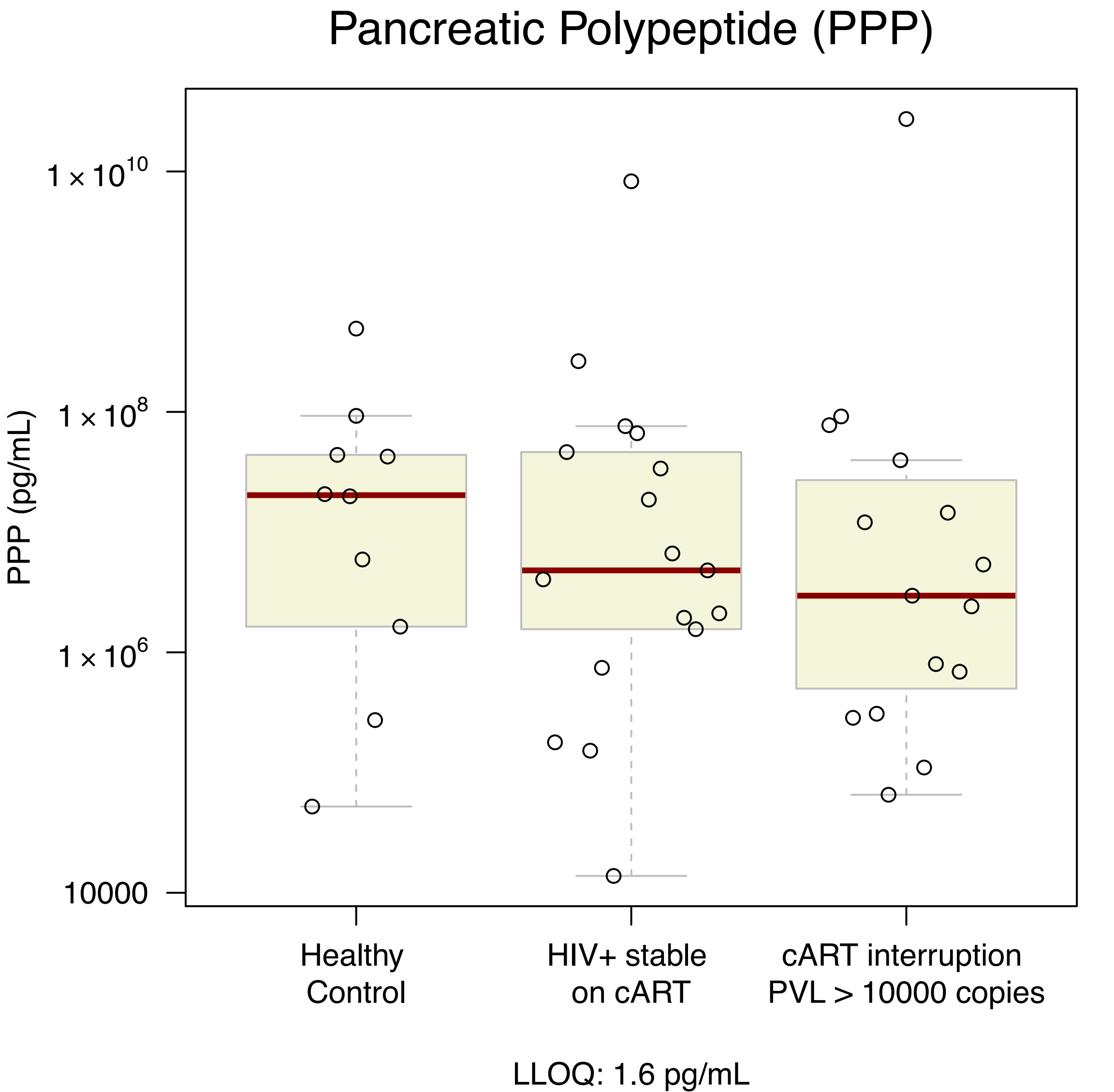

Pancreatic Polypeptide (PPP)
PPP (pg/mL)
1 × 10^10
1 × 10^8
1 × 10^6
10000
Healthy Control
HIV+ stable on cART
cART interruption PVL > 10000 copies
LLOQ: 1.6 pg/mL

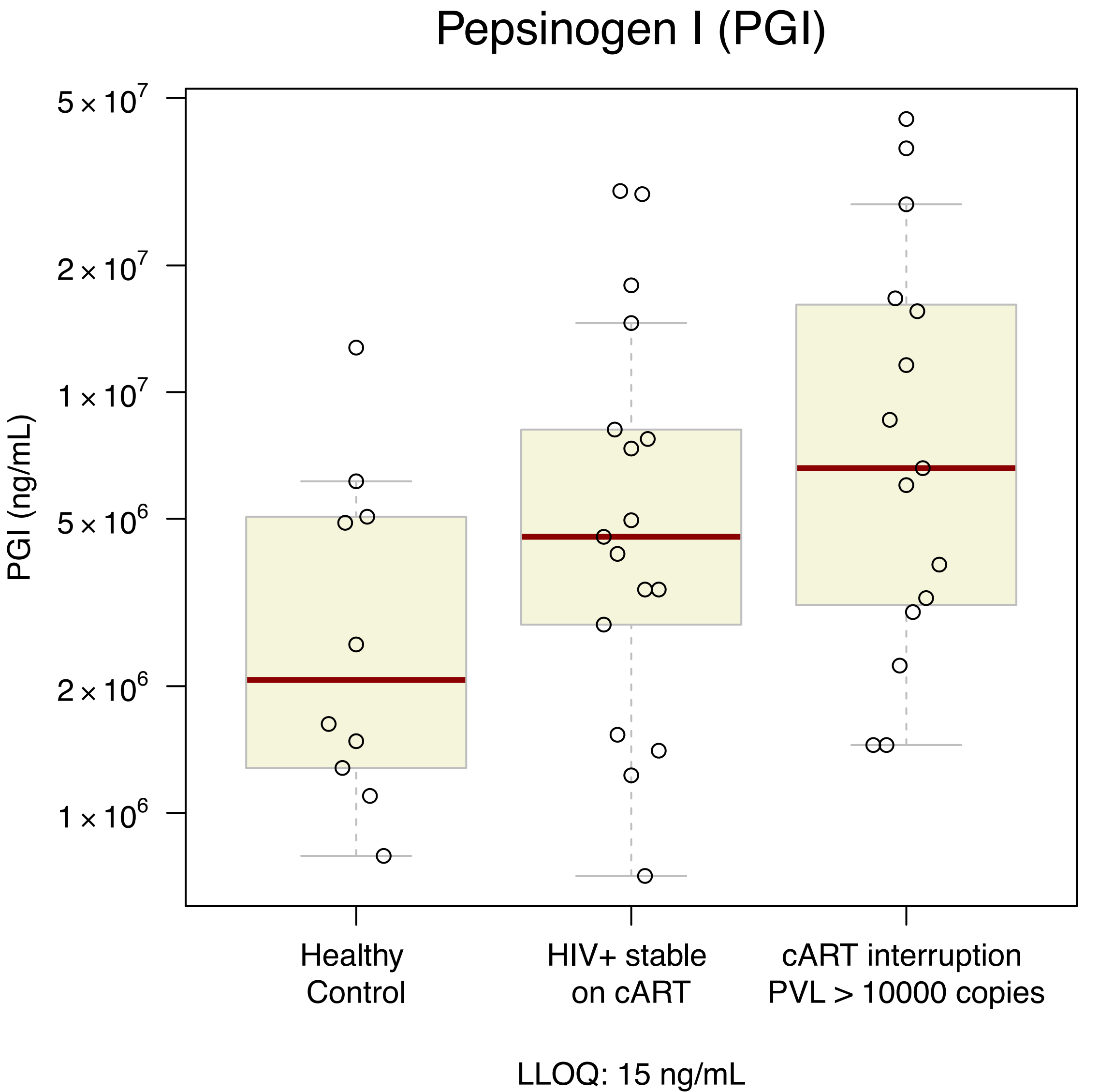

Pepsinogen I (PGI)
PGI (ng/mL)
5 × 10^7
2 × 10^7
1 × 10^7
5 × 10^6
2 × 10^6
1 × 10^6
Healthy Control
HIV+ stable on cART
cART interruption PVL > 10000 copies
LLOQ: 15 ng/mL

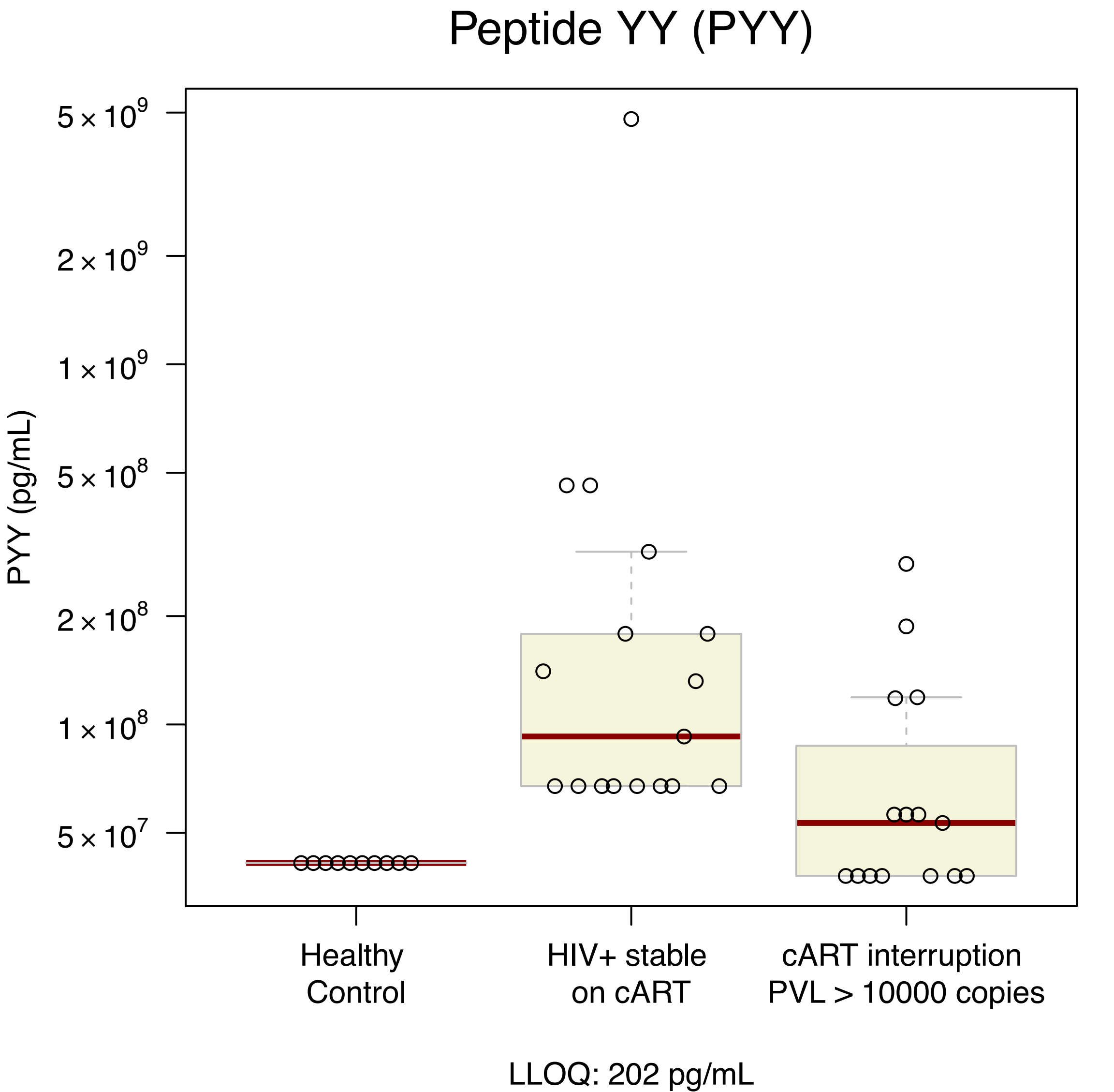

Peptide YY (PYY)
PYY (pg/mL)
5 × 10^9
2 × 10^9
1 × 10^9
5 × 10^8
2 × 10^8
1 × 10^8
5 × 10^7
Healthy Control
HIV+ stable on cART
cART interruption PVL > 10000 copies
LLOQ: 202 pg/mL

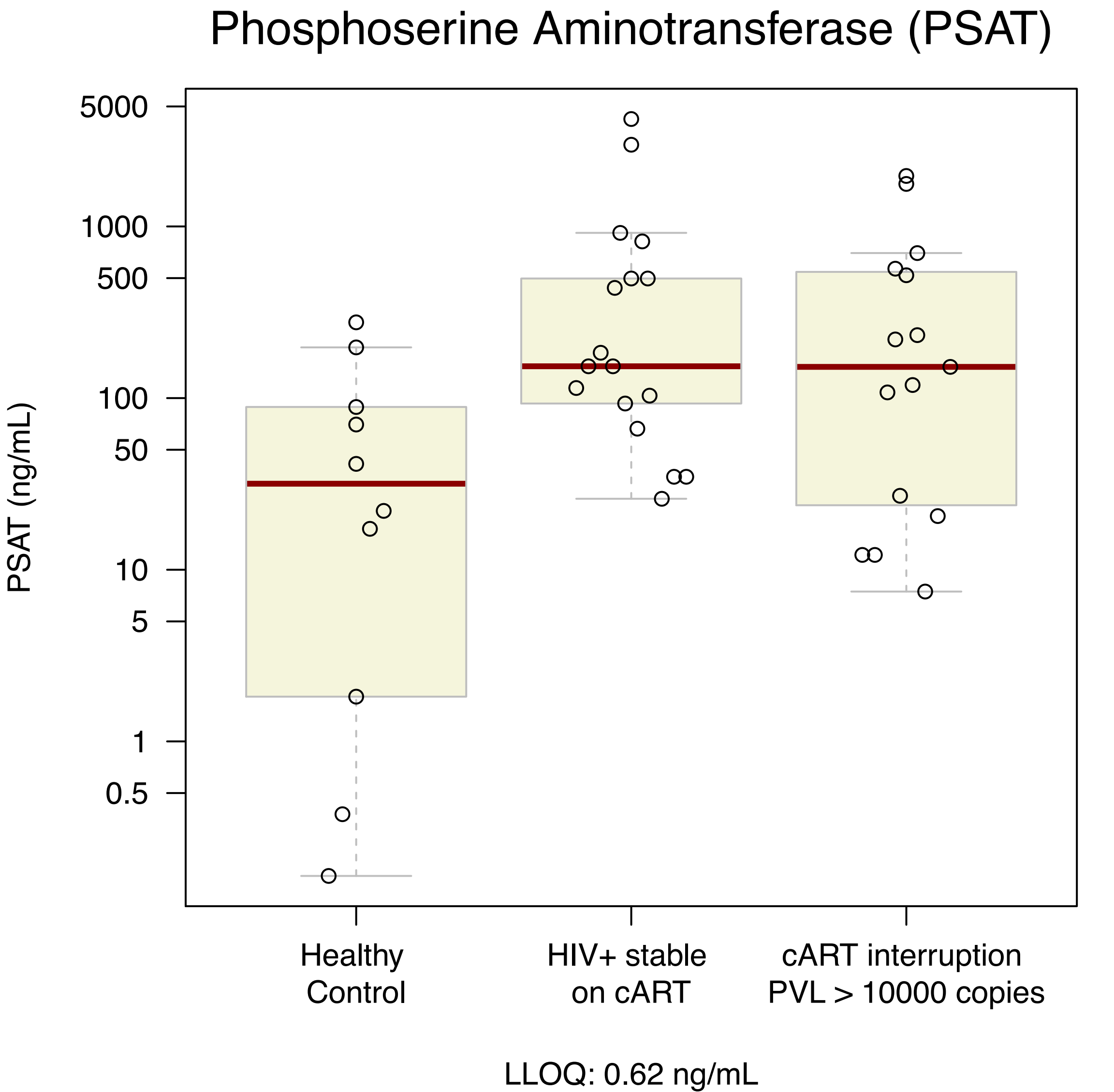
Phosphoserine Aminotransferase (PSAT)
PSAT (ng/mL)
5000
1000
500
100
50
10
5
1
0.5
Healthy Control
HIV+ stable on cART
cART interruption PVL > 10000 copies
LLOQ: 0.62 ng/mL

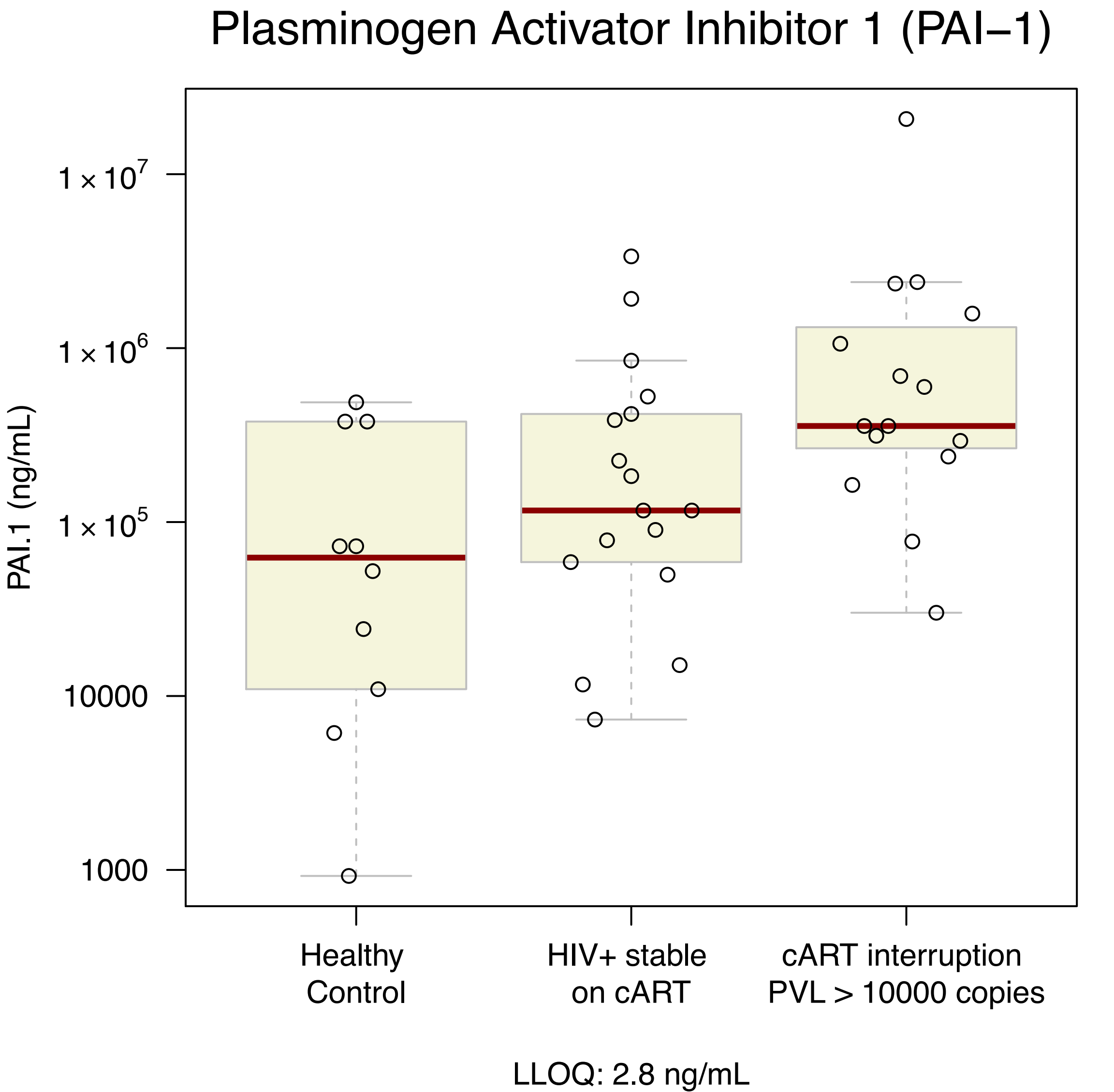

Plasminogen Activator Inhibitor 1 (PAI–1)
PAI.1 (ng/mL)
1 × 10^7
1 × 10^6
1 × 10^5
10000
1000
Healthy Control
HIV+ stable on cART
cART interruption PVL > 10000 copies
LLOQ: 2.8 ng/mL

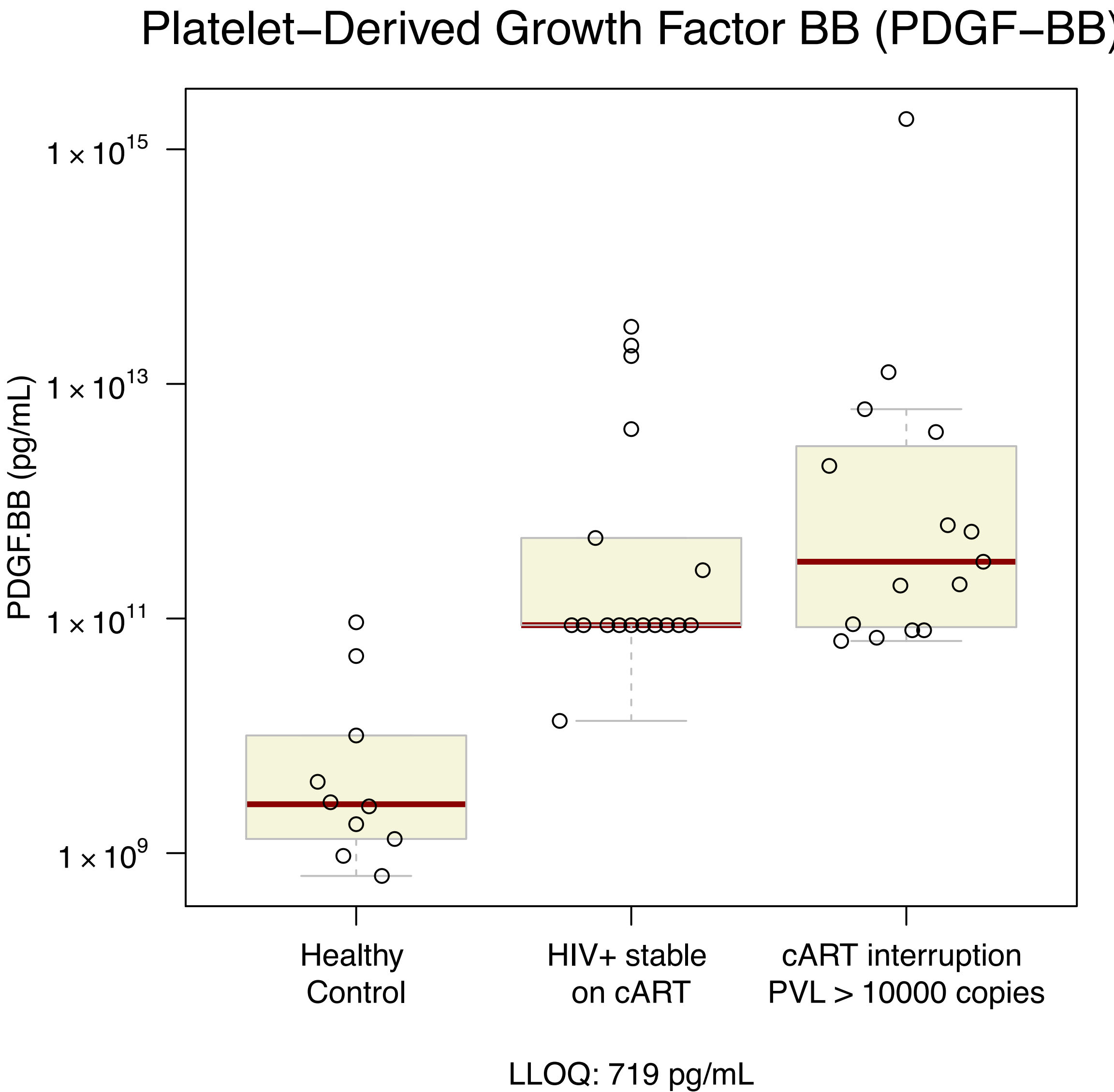

Platelet−Derived Growth Factor BB (PDGF−BB)
PDGF.BB (pg/mL)
1×10^{15}
1×10^{13}
1×10^{11}
1×10^{9}
Healthy Control
HIV+ stable on cART
cART interruption PVL > 10000 copies
LLOQ: 719 pg/mL

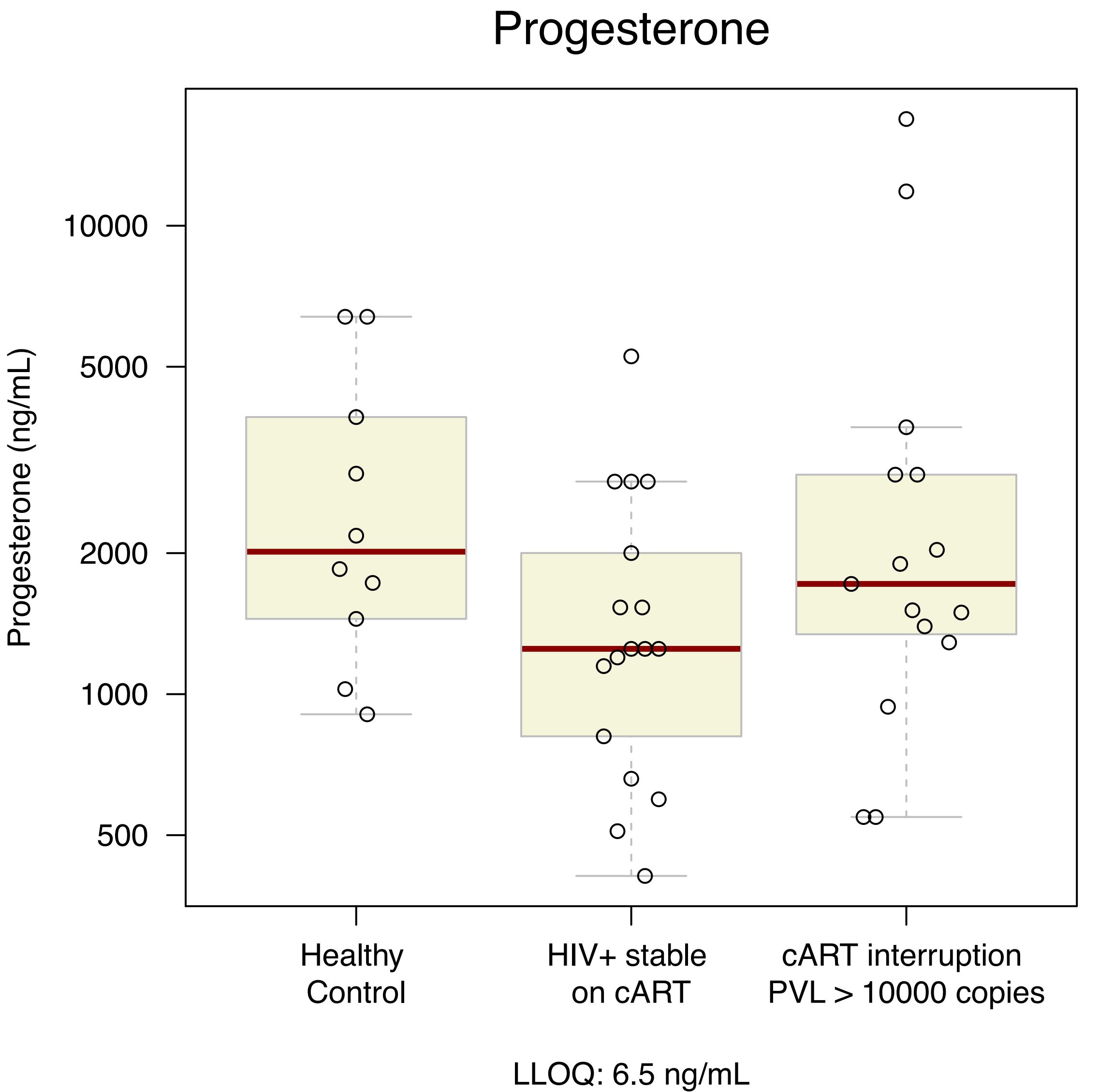
Progesterone
Progesterone (ng/mL)
10000
5000
2000
1000
500
Healthy
Control
HIV+ stable
on cART
cART interruption
PVL > 10000 copies
LLOQ: 6.5 ng/mL

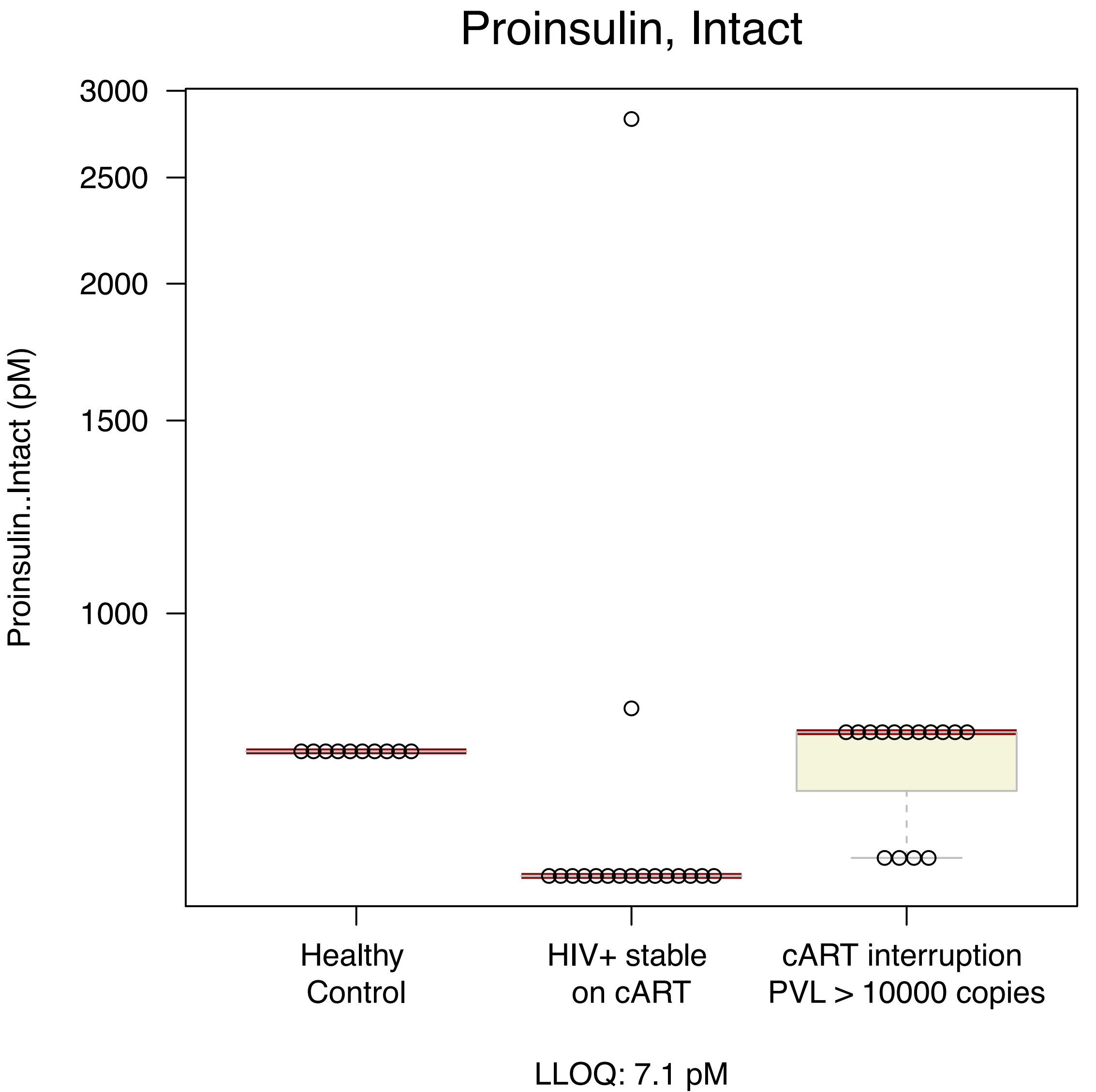
Proinsulin, Intact
Proinsulin..Intact (pM)
3000
2500
2000
1500
1000
Healthy Control
HIV+ stable on cART
cART interruption PVL > 10000 copies
LLOQ: 7.1 pM

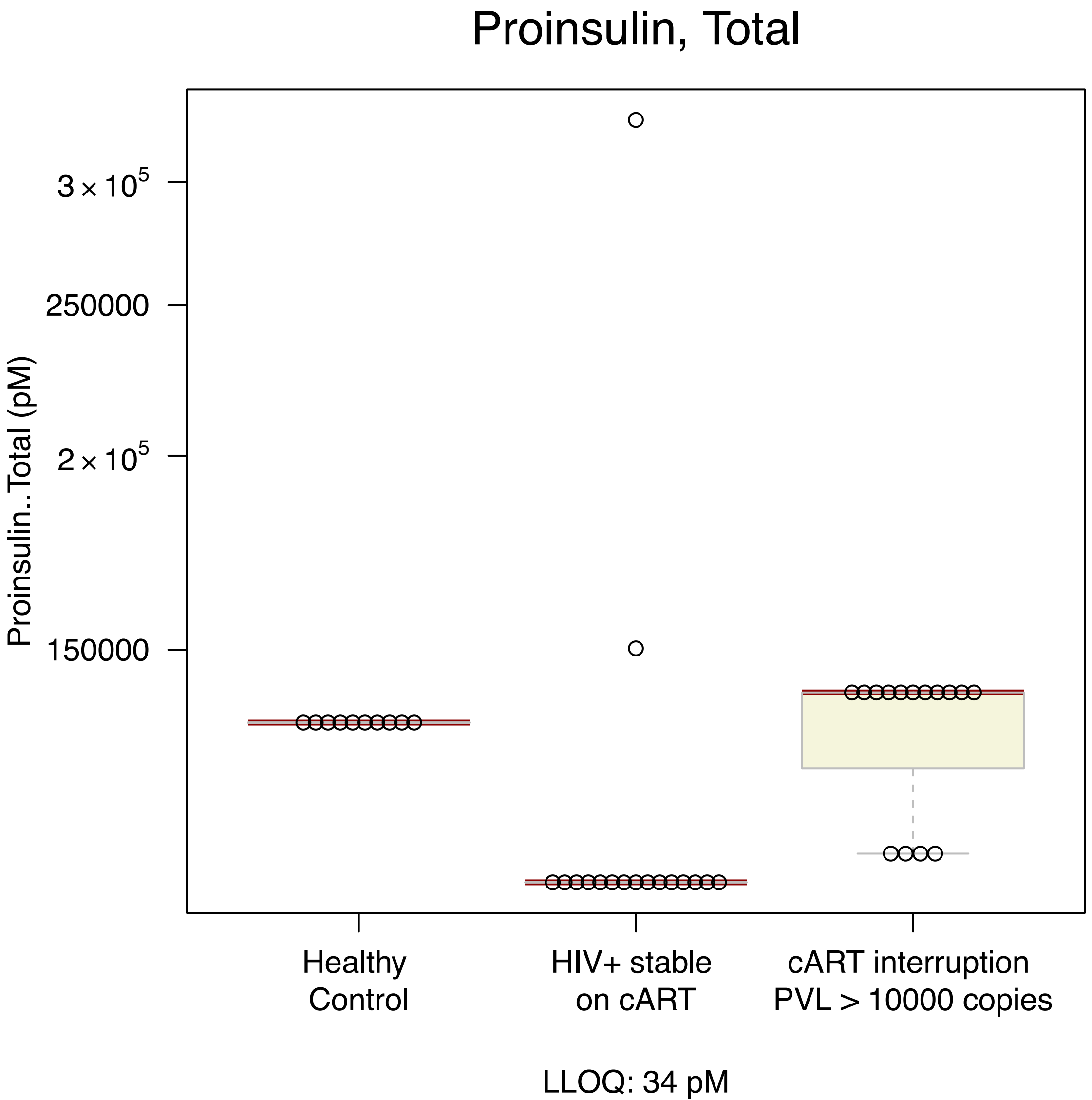

Proinsulin, Total
Proinsulin..Total (pM)
3×10^5
250000
2×10^5
150000
Healthy Control
HIV+ stable on cART
cART interruption PVL > 10000 copies
LLOQ: 34 pM

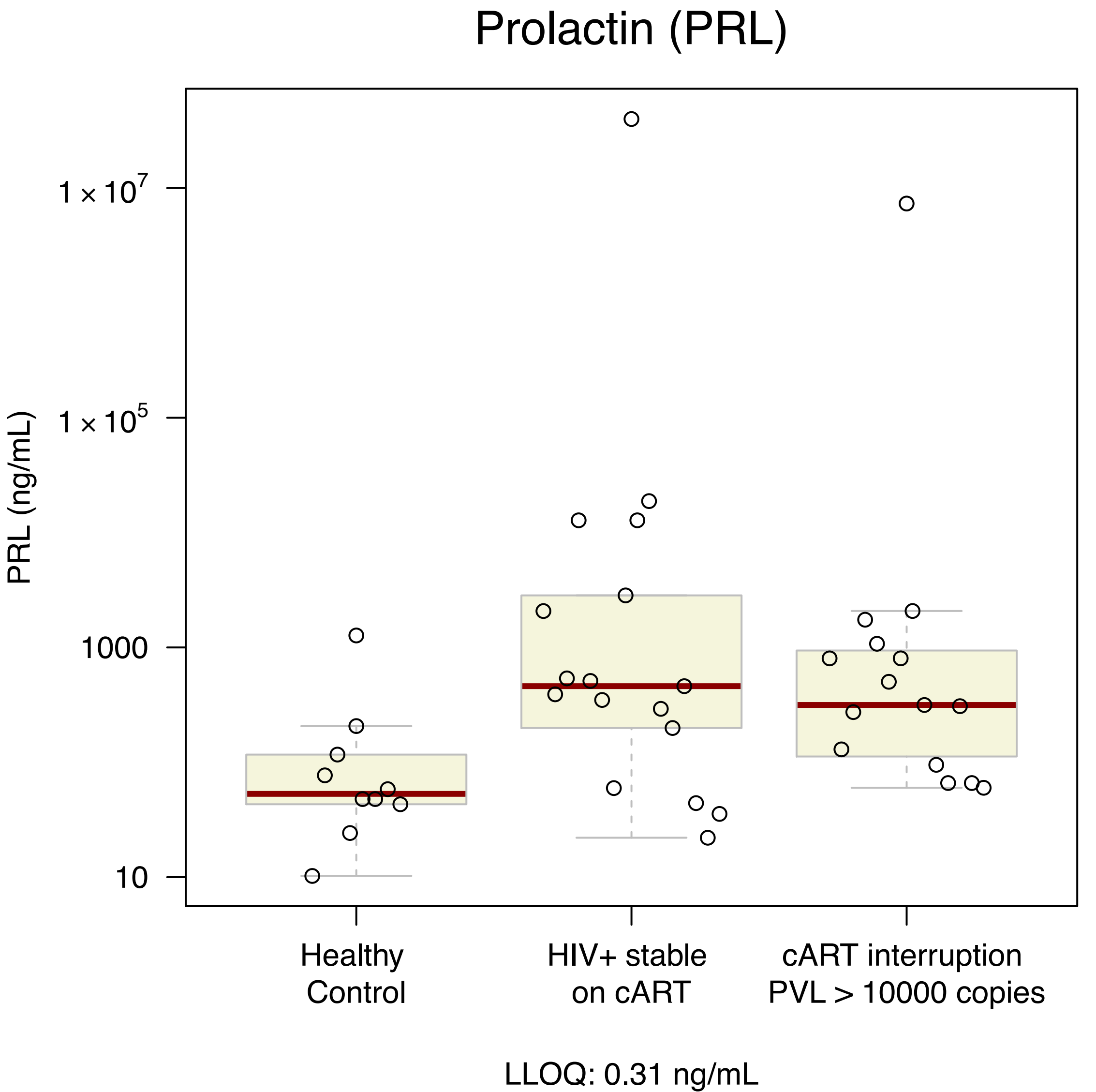

Prolactin (PRL)
PRL (ng/mL)
1 × 10^7
1 × 10^5
1000
10
Healthy Control
HIV+ stable on cART
cART interruption PVL > 10000 copies
LLOQ: 0.31 ng/mL

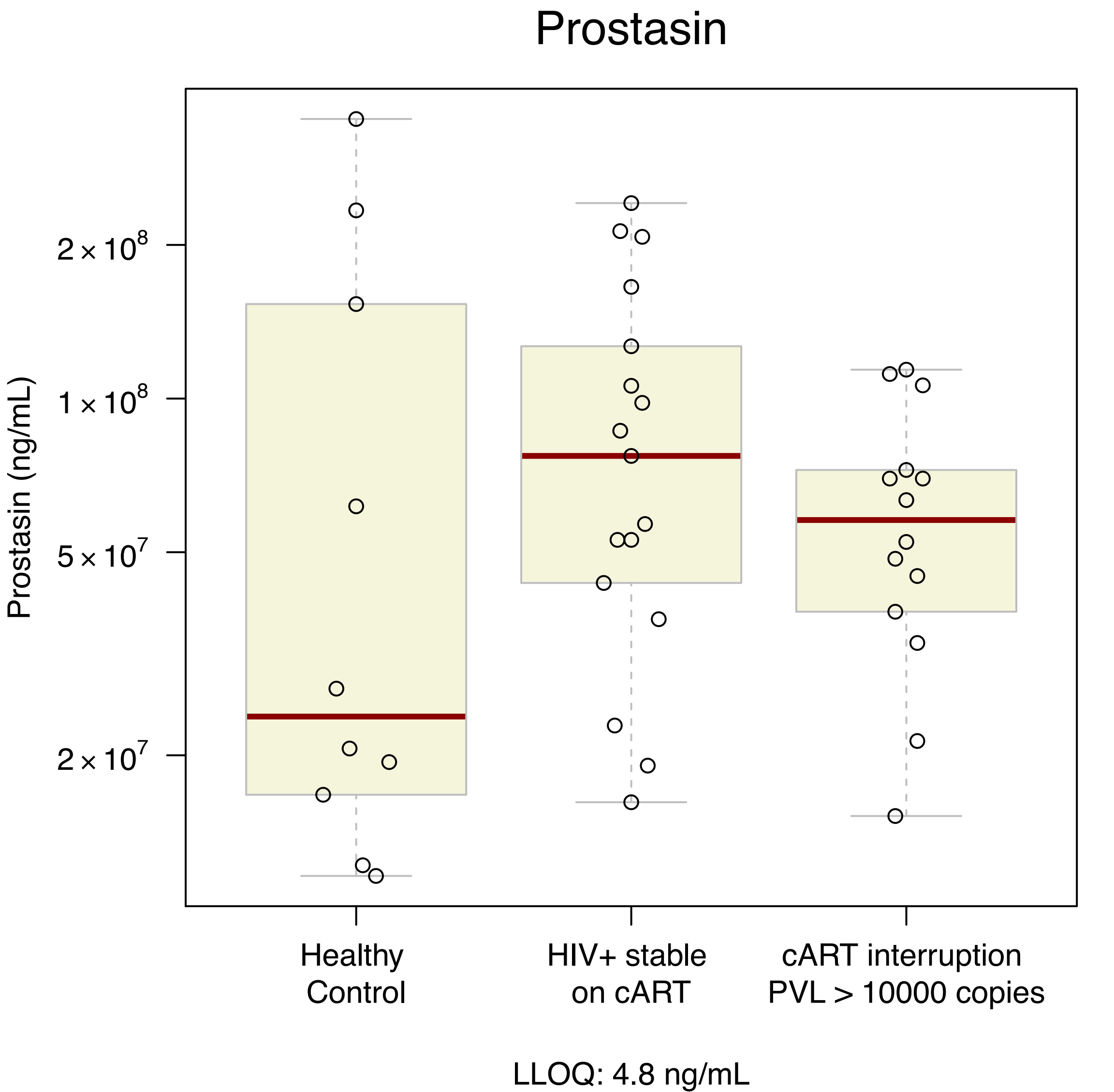
Prostasin
Prostasin (ng/mL)
2×10^8
1×10^8
5×10^7
2×10^7
Healthy Control
HIV+ stable on cART
cART interruption PVL > 10000 copies
LLOQ: 4.8 ng/mL

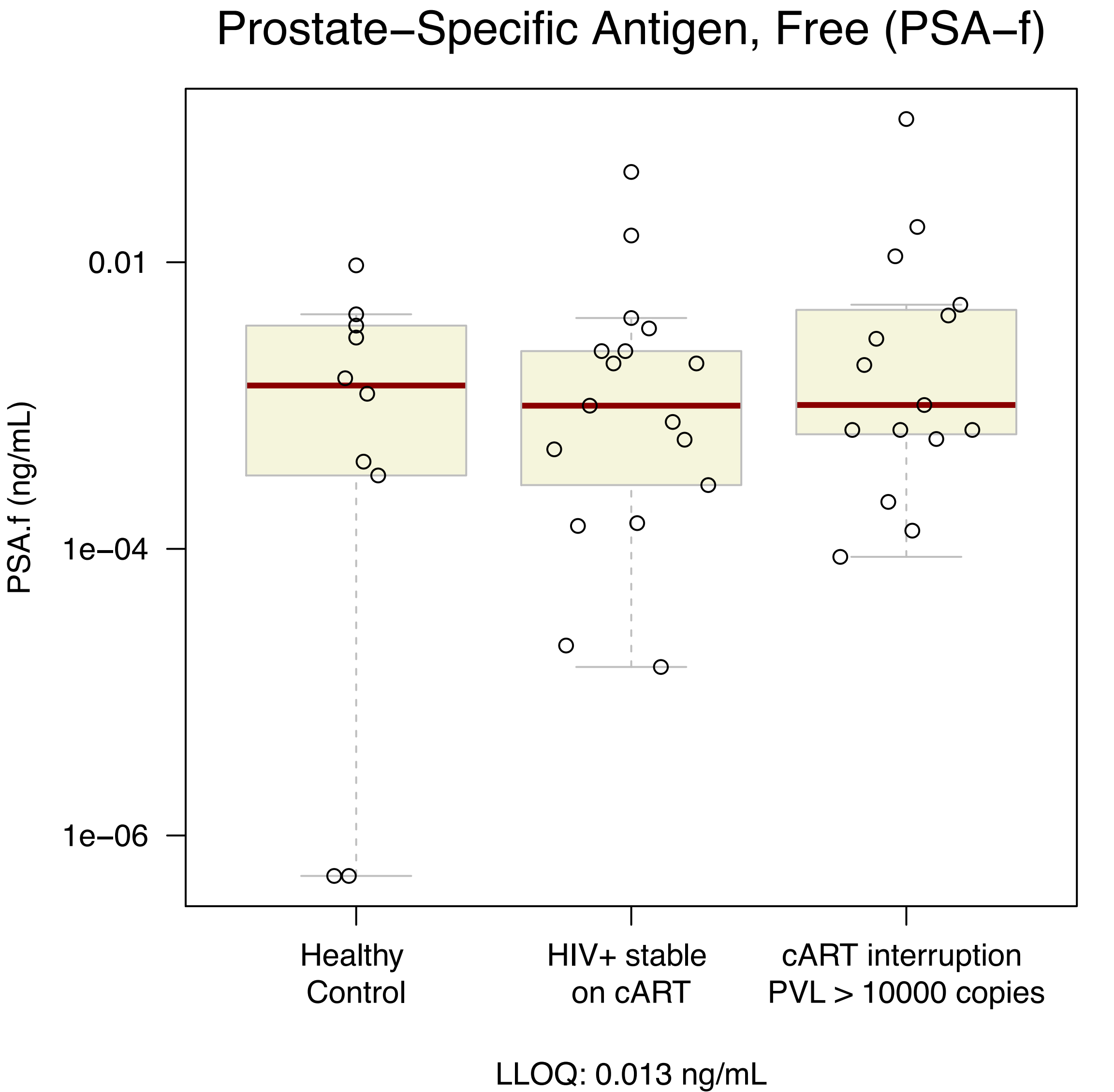

Prostate−Specific Antigen, Free (PSA−f)
PSA.f (ng/mL)
0.01
1e−04
1e−06
Healthy Control
HIV+ stable on cART
cART interruption PVL > 10000 copies
LLOQ: 0.013 ng/mL

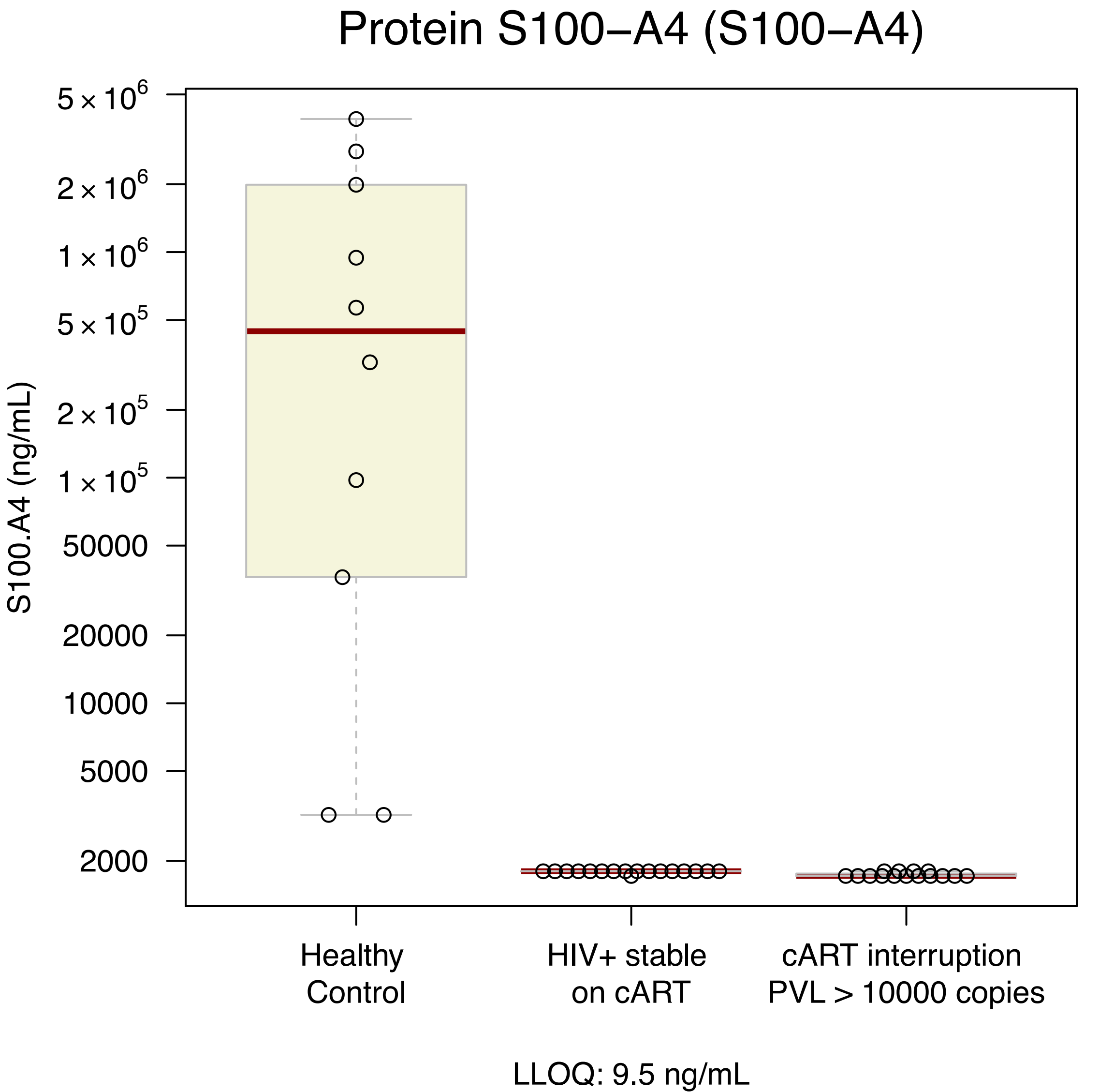
Protein S100−A4 (S100−A4)
S100.A4 (ng/mL)
5 × 10^6
2 × 10^6
1 × 10^6
5 × 10^5
2 × 10^5
1 × 10^5
50000
20000
10000
5000
2000
Healthy Control
HIV+ stable on cART
cART interruption PVL > 10000 copies
LLOQ: 9.5 ng/mL

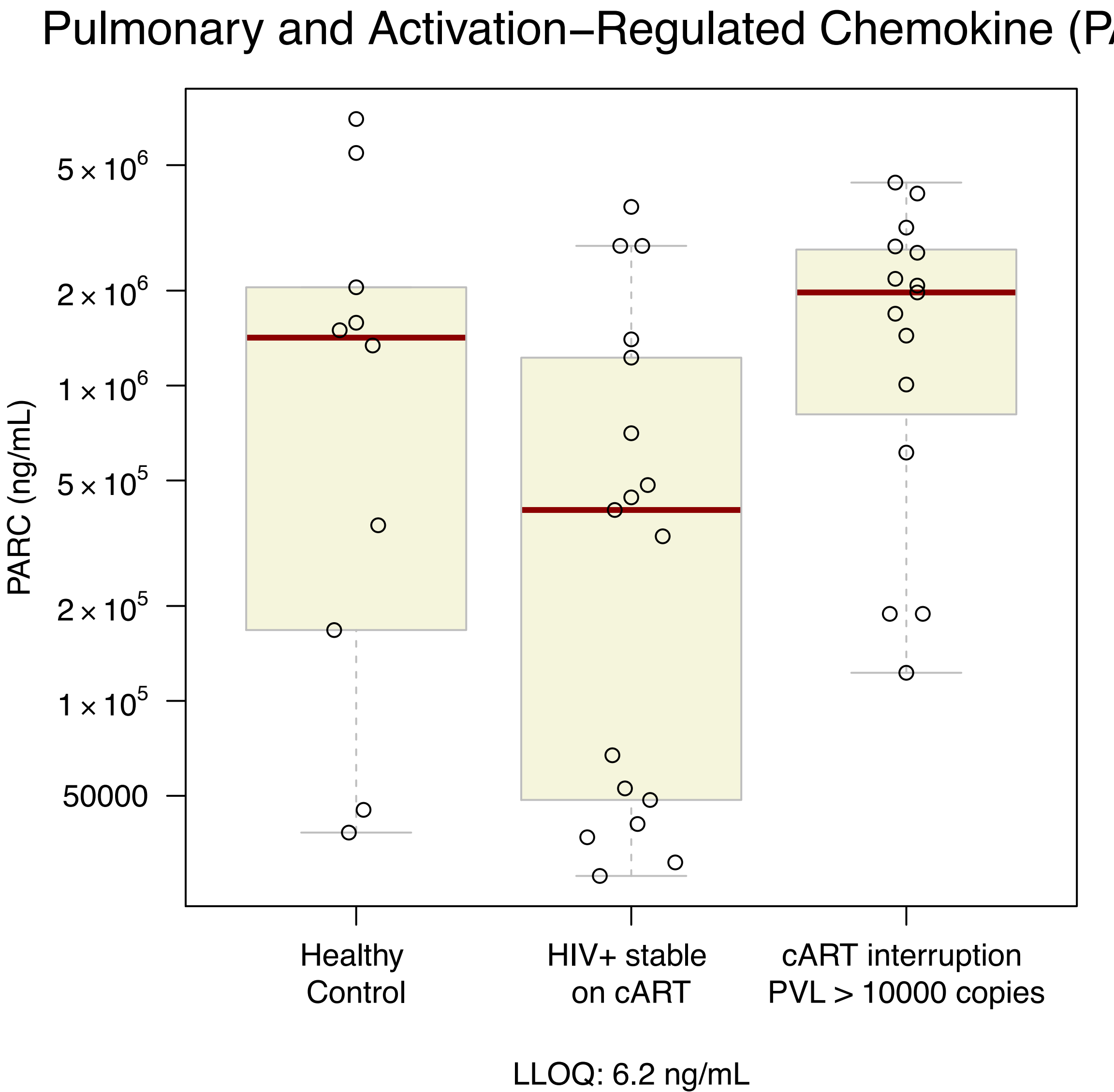

Pulmonary and Activation−Regulated Chemokine (PA
PARC (ng/mL)
5×10^6
2×10^6
1×10^6
5×10^5
2×10^5
1×10^5
50000
Healthy Control
HIV+ stable on cART
cART interruption PVL > 10000 copies
LLOQ: 6.2 ng/mL

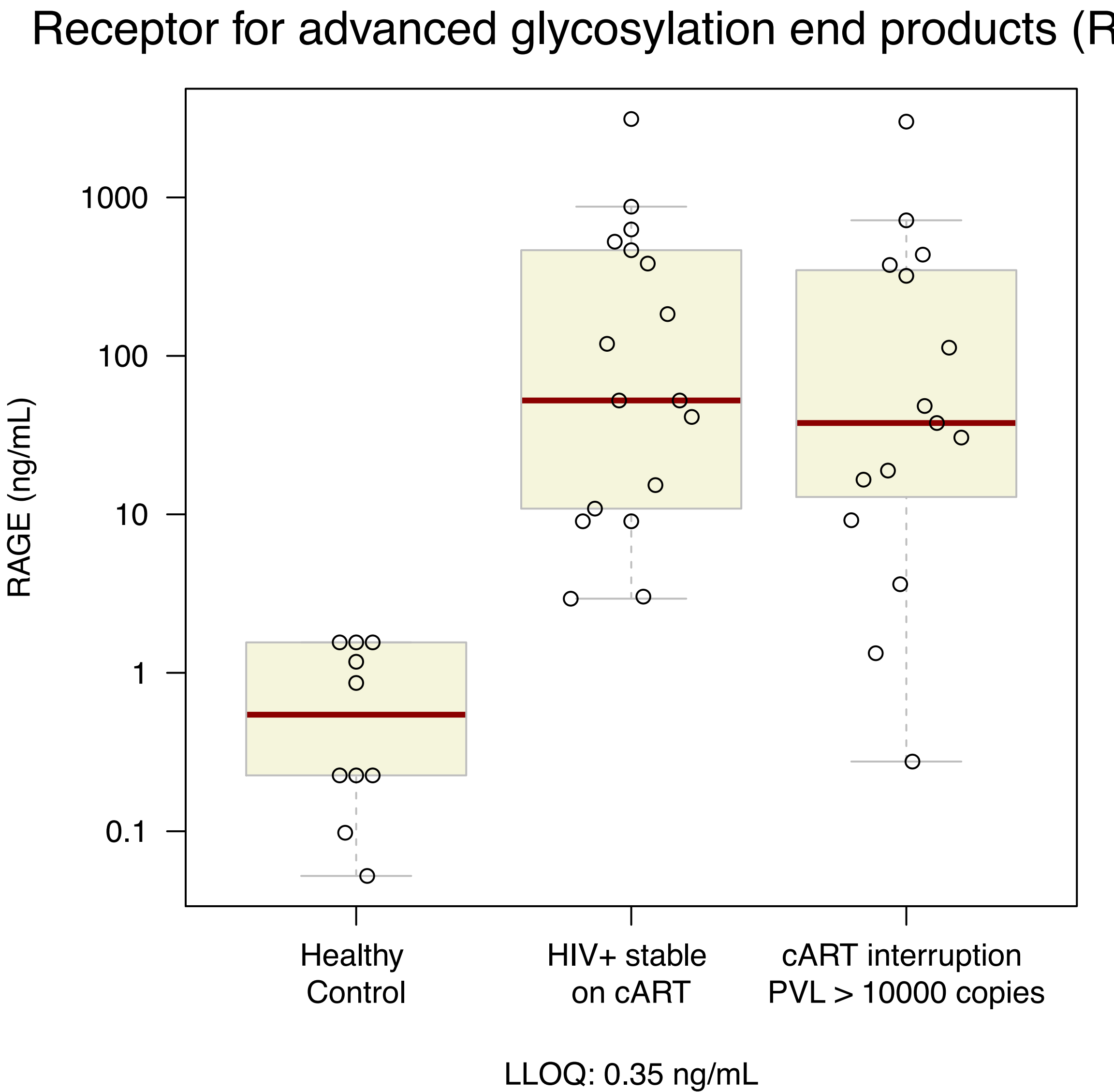

Receptor for advanced glycosylation end products (R
RAGE (ng/mL)
1000
100
10
1
0.1
Healthy Control
HIV+ stable on cART
cART interruption PVL > 10000 copies
LLOQ: 0.35 ng/mL

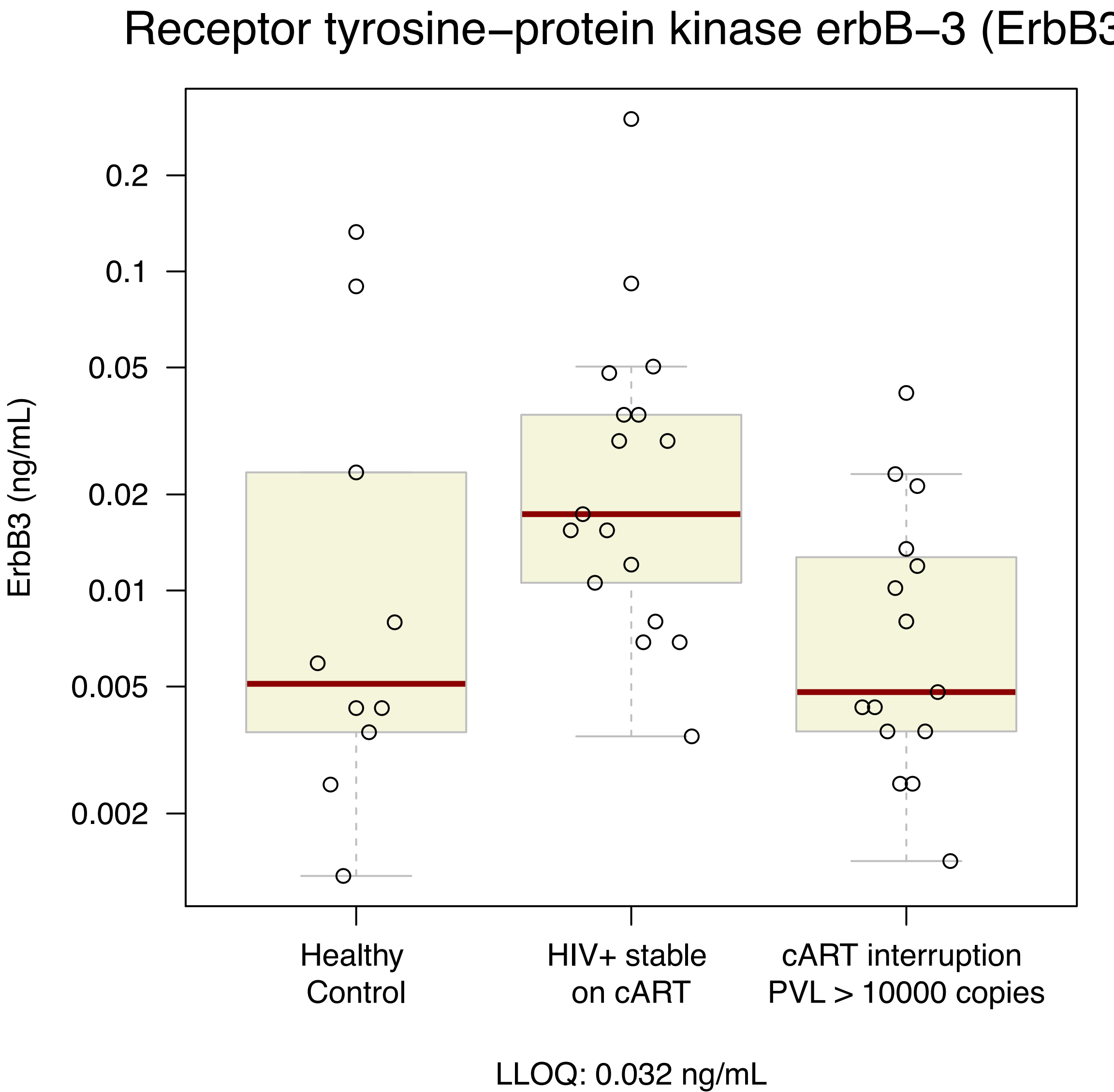

Receptor tyrosine−protein kinase erbB−3 (ErbB3
ErbB3 (ng/mL)
0.2
0.1
0.05
0.02
0.01
0.005
0.002
Healthy Control
HIV+ stable on cART
cART interruption PVL > 10000 copies
LLOQ: 0.032 ng/mL

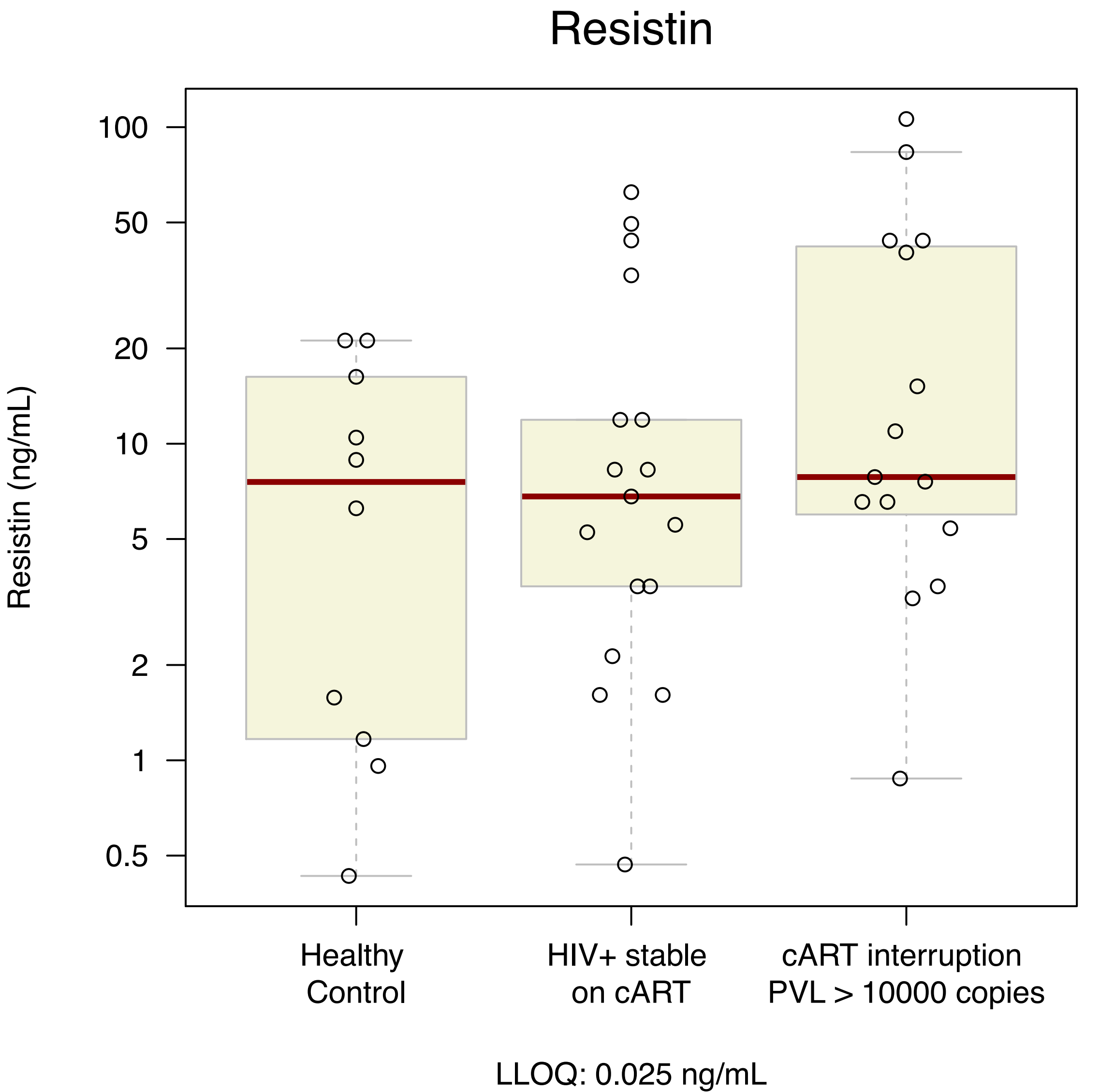
Resistin
100
50
20
10
5
2
1
0.5
Resistin (ng/mL)
Healthy
Control
HIV+ stable
on cART
cART interruption
PVL > 10000 copies
LLOQ: 0.025 ng/mL

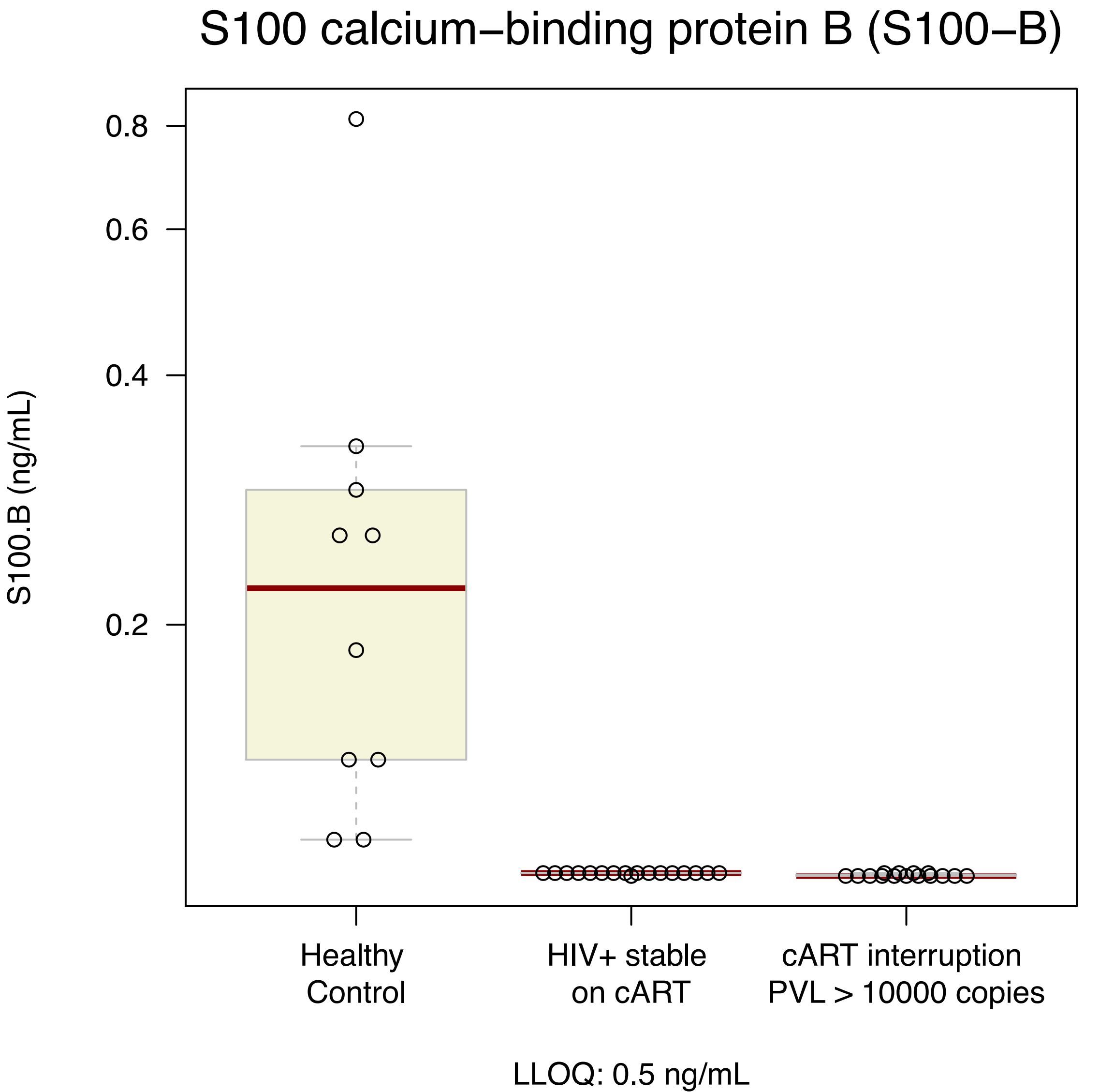

S100 calcium−binding protein B (S100−B)
S100.B (ng/mL)
0.8
0.6
0.4
0.2
Healthy Control
HIV+ stable on cART
cART interruption PVL > 10000 copies
LLOQ: 0.5 ng/mL

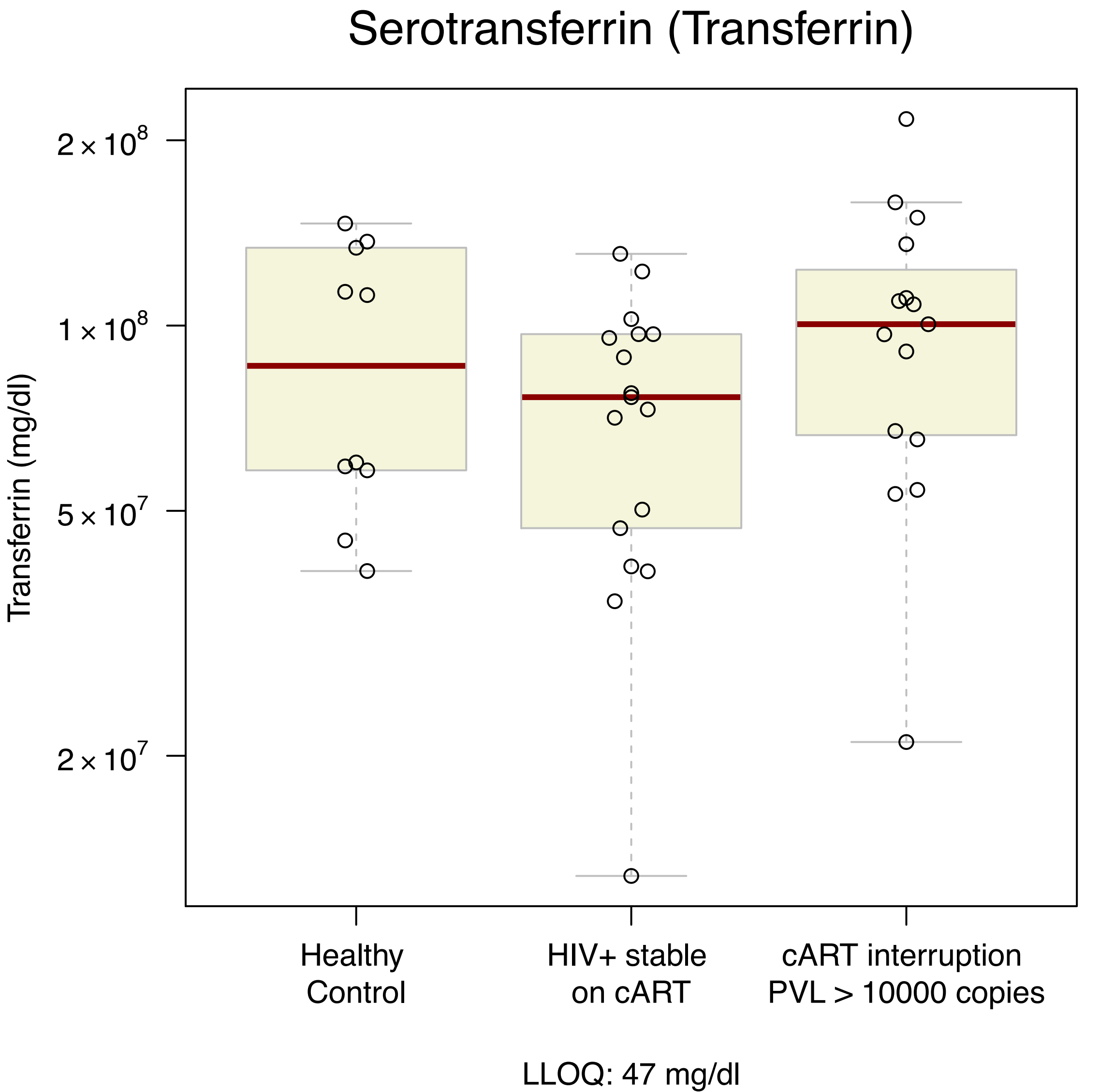

Serotransferrin (Transferrin)
Transferrin (mg/dl)
2×10^8
1×10^8
5×10^7
2×10^7
Healthy Control
HIV+ stable on cART
cART interruption PVL > 10000 copies
LLOQ: 47 mg/dl

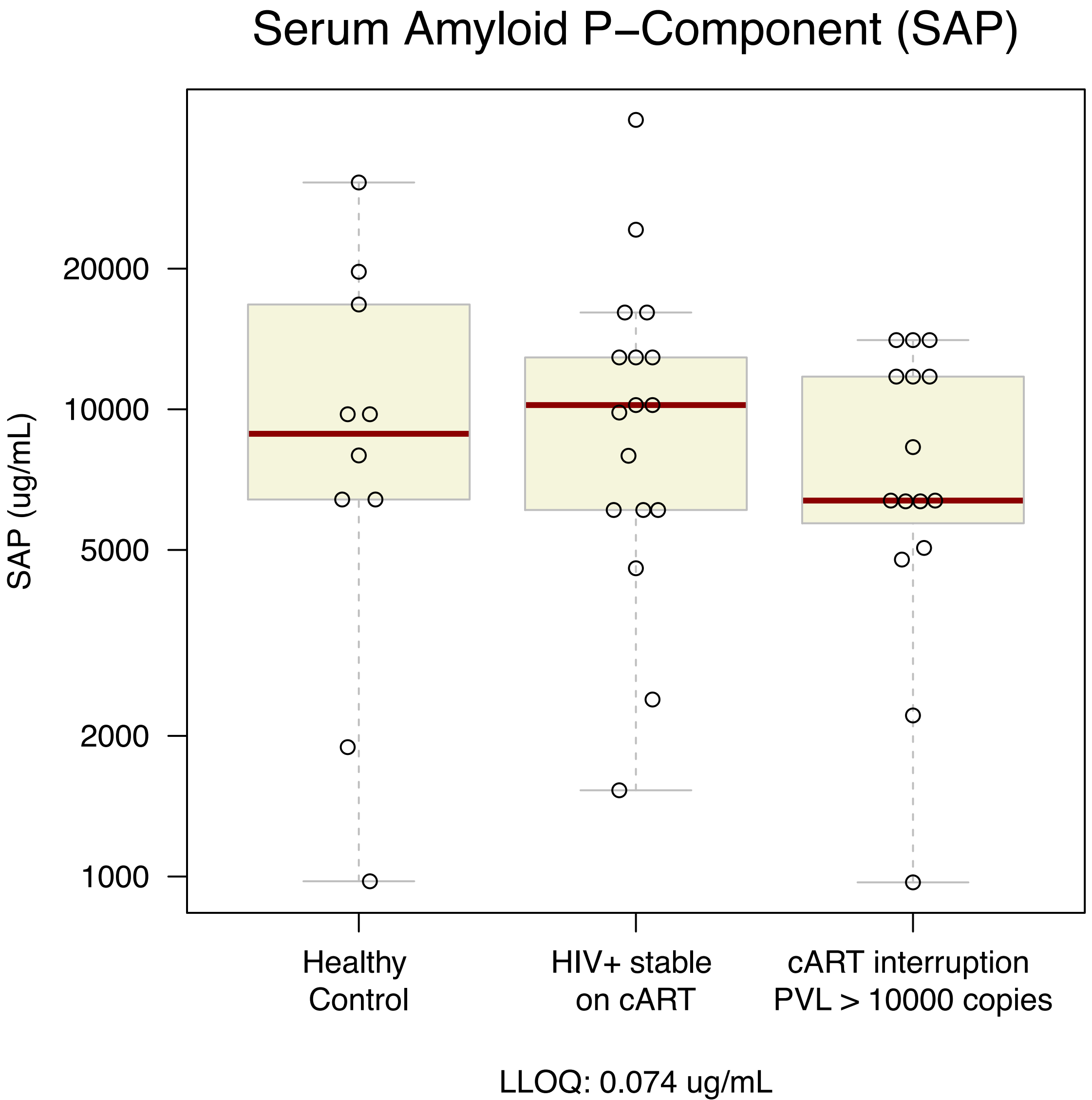
Serum Amyloid P−Component (SAP)
SAP (ug/mL)
20000
10000
5000
2000
1000
Healthy Control
HIV+ stable on cART
cART interruption PVL > 10000 copies
LLOQ: 0.074 ug/mL

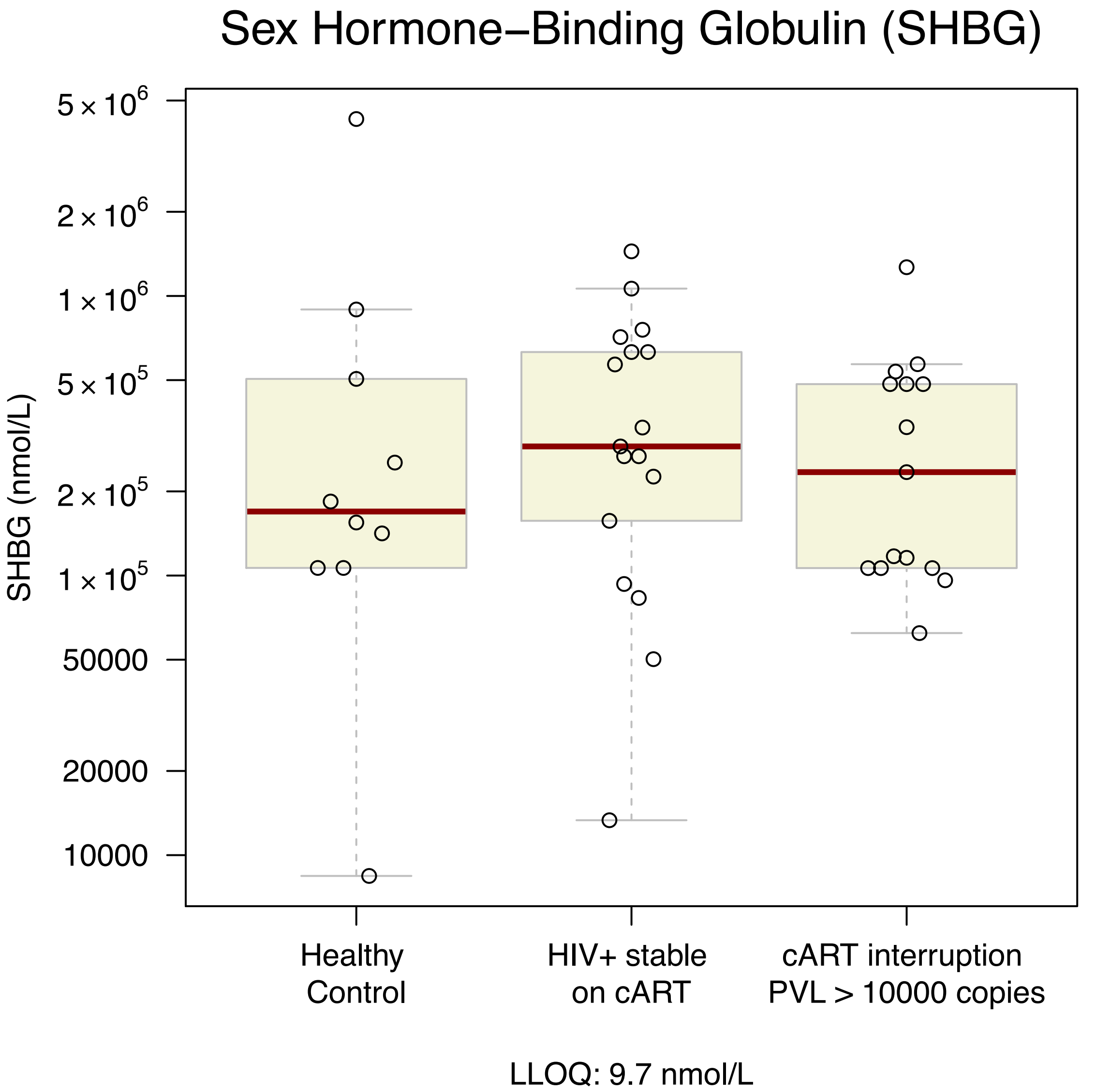

Sex Hormone–Binding Globulin (SHBG)
SHBG (nmol/L)
5 × 10^6
2 × 10^6
1 × 10^6
5 × 10^5
2 × 10^5
1 × 10^5
50000
20000
10000
Healthy Control
HIV+ stable on cART
cART interruption PVL > 10000 copies
LLOQ: 9.7 nmol/L

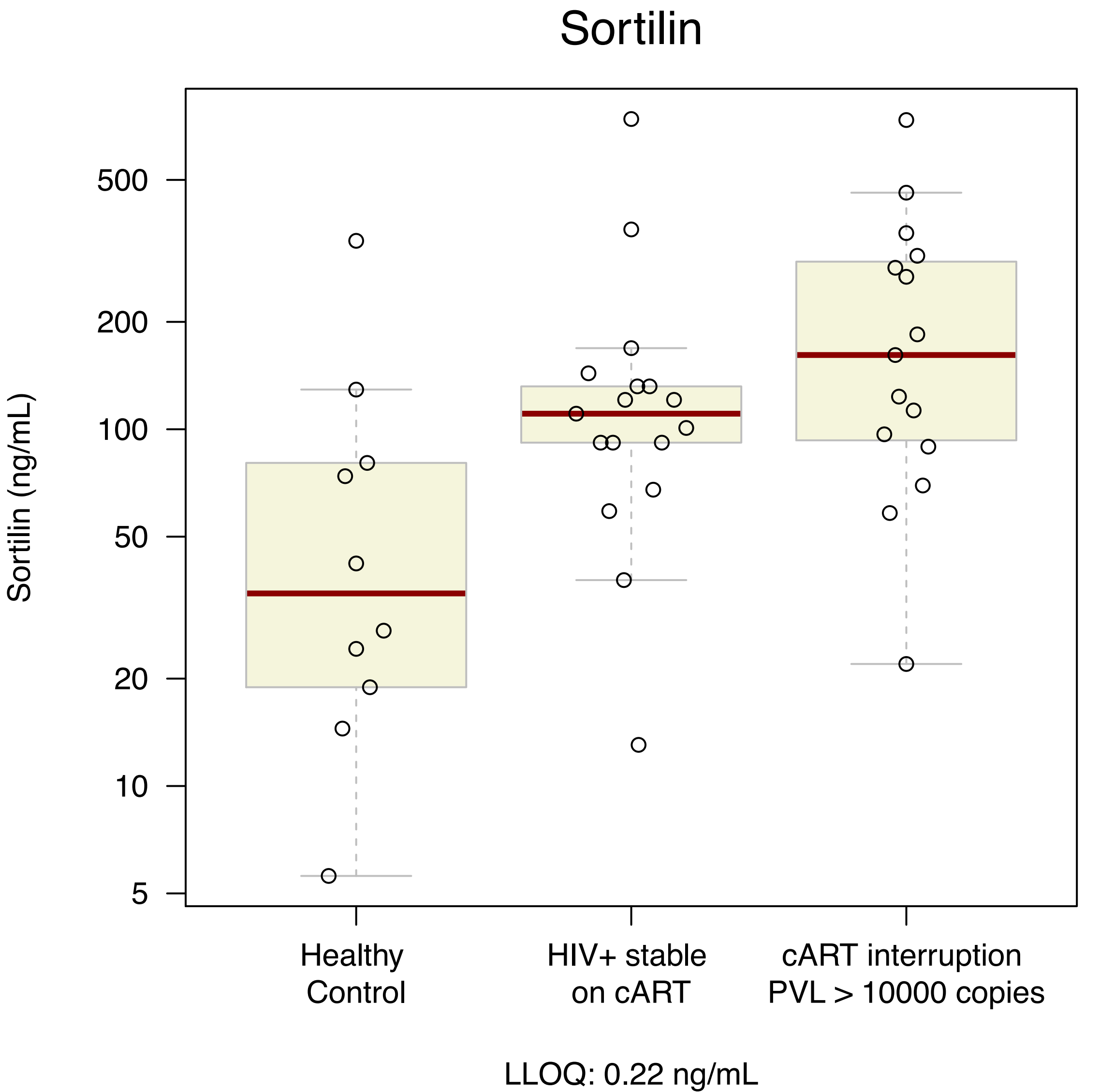
Sortilin
Sortilin (ng/mL)
500
200
100
50
20
10
5
Healthy Control
HIV+ stable on cART
cART interruption PVL > 10000 copies
LLOQ: 0.22 ng/mL

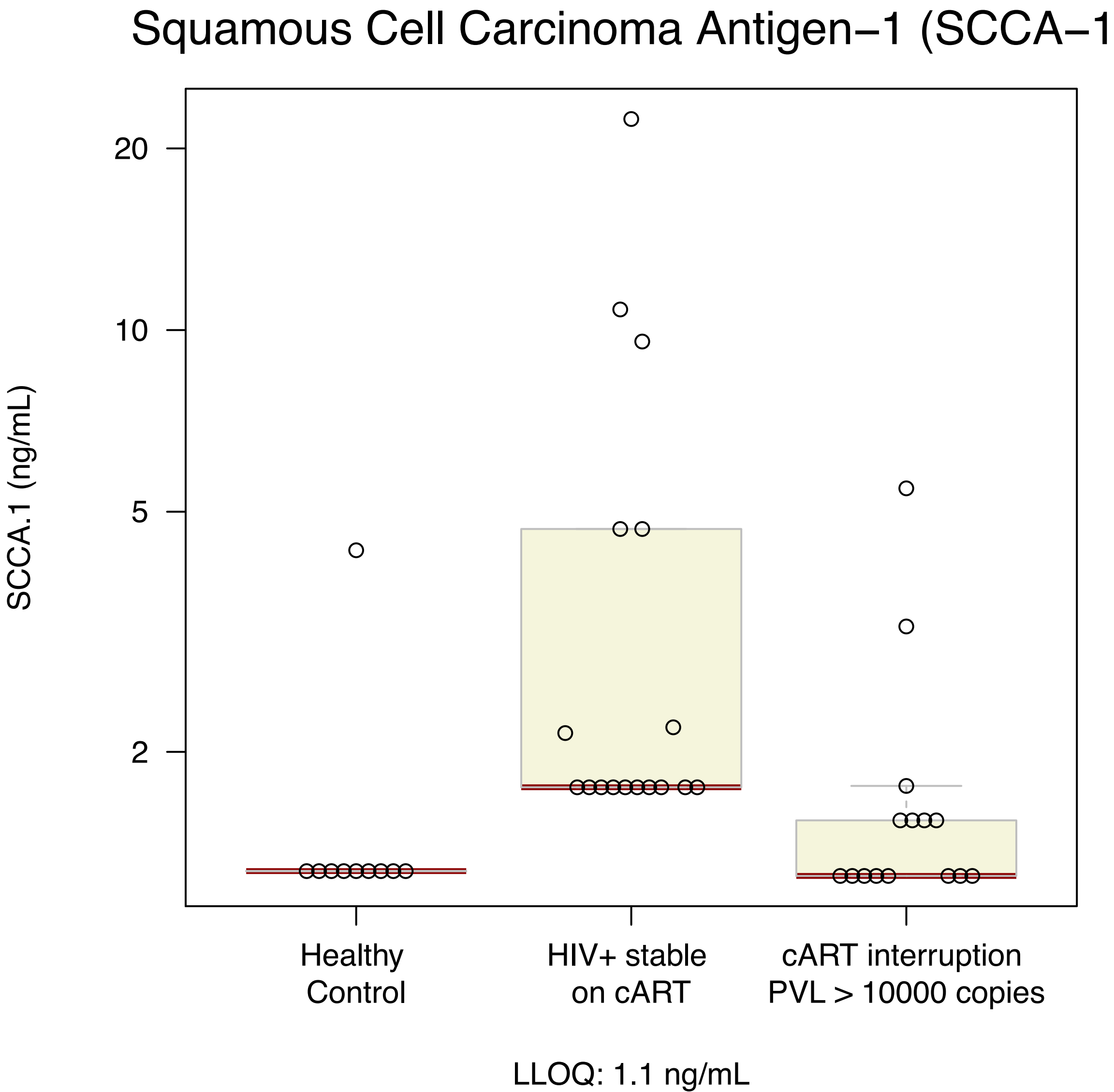

Squamous Cell Carcinoma Antigen−1 (SCCA−1
SCCA.1 (ng/mL)
20
10
5
2
Healthy Control
HIV+ stable on cART
cART interruption PVL > 10000 copies
LLOQ: 1.1 ng/mL

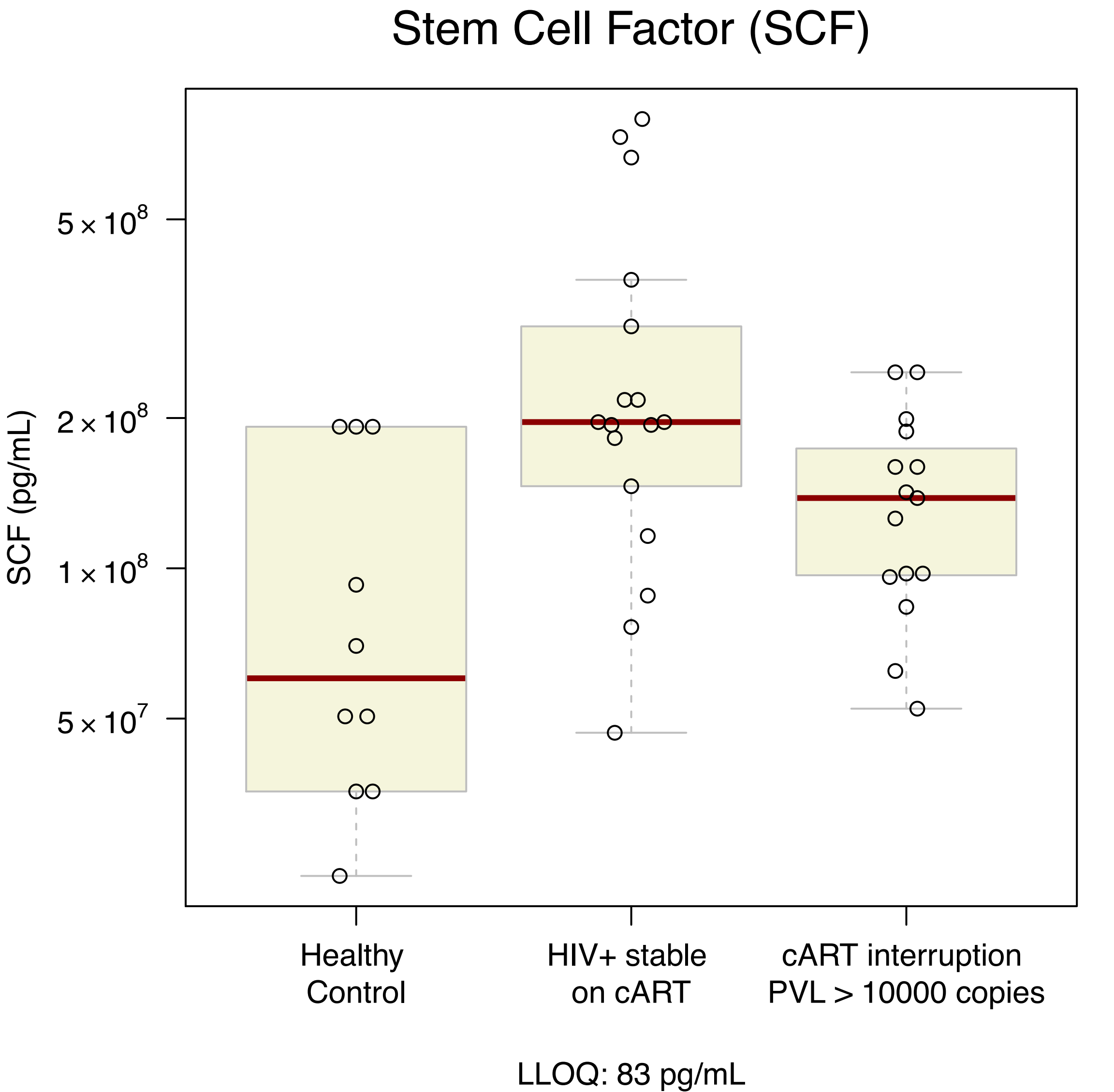
Stem Cell Factor (SCF)
SCF (pg/mL)
5 × 10^8
2 × 10^8
1 × 10^8
5 × 10^7
Healthy Control
HIV+ stable on cART
cART interruption PVL > 10000 copies
LLOQ: 83 pg/mL

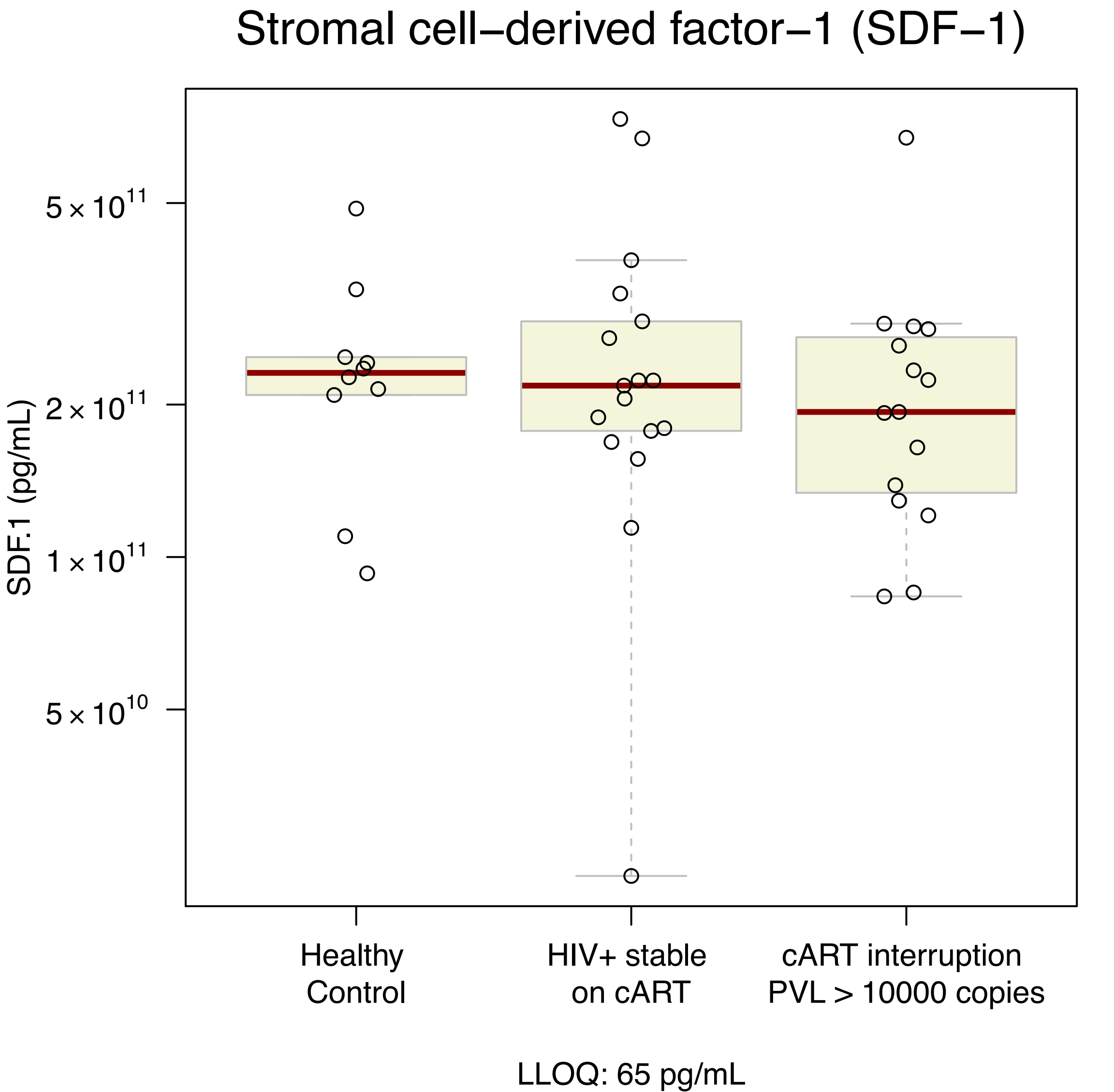

Stromal cell−derived factor−1 (SDF−1)
SDF.1 (pg/mL)
5×10^{11}
2×10^{11}
1×10^{11}
5×10^{10}
Healthy Control
HIV+ stable on cART
cART interruption PVL > 10000 copies
LLOQ: 65 pg/mL

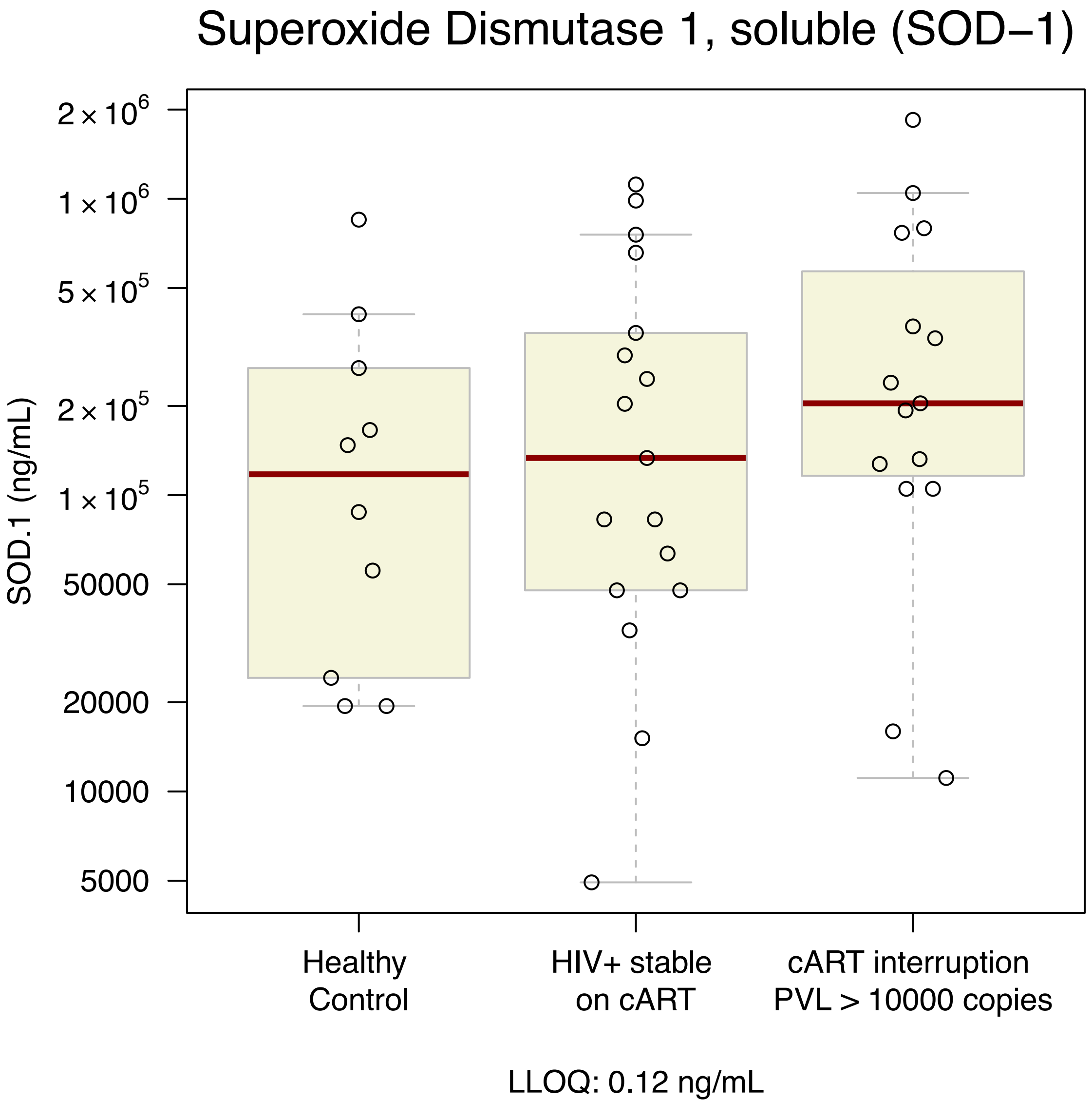
Superoxide Dismutase 1, soluble (SOD−1)
SOD.1 (ng/mL)
2 × 10^6
1 × 10^6
5 × 10^5
2 × 10^5
1 × 10^5
50000
20000
10000
5000
Healthy Control
HIV+ stable on cART
cART interruption PVL > 10000 copies
LLOQ: 0.12 ng/mL

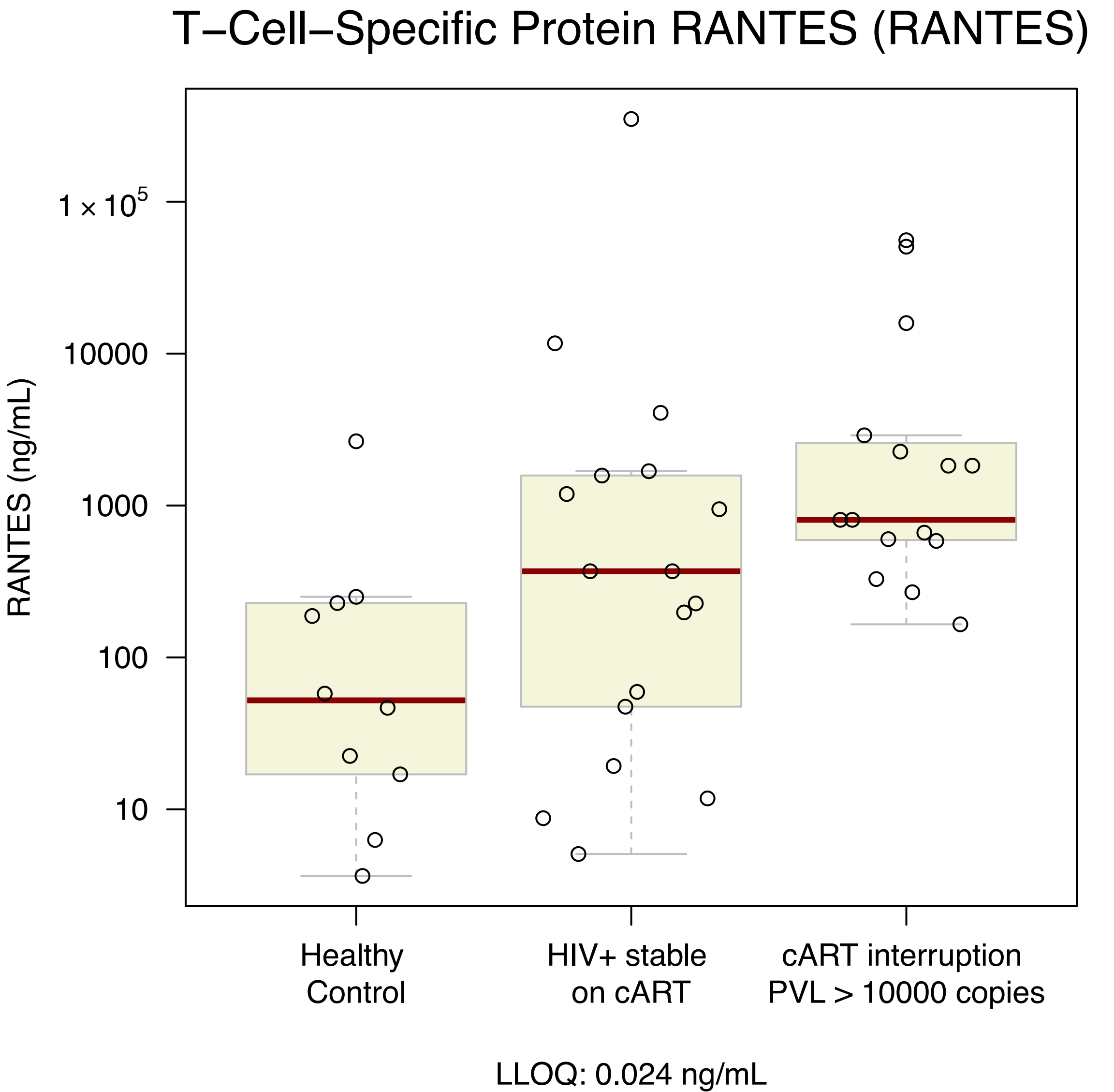
T−Cell−Specific Protein RANTES (RANTES)
RANTES (ng/mL)
1 × 10^5
10000
1000
100
10
Healthy Control
HIV+ stable on cART
cART interruption PVL > 10000 copies
LLOQ: 0.024 ng/mL

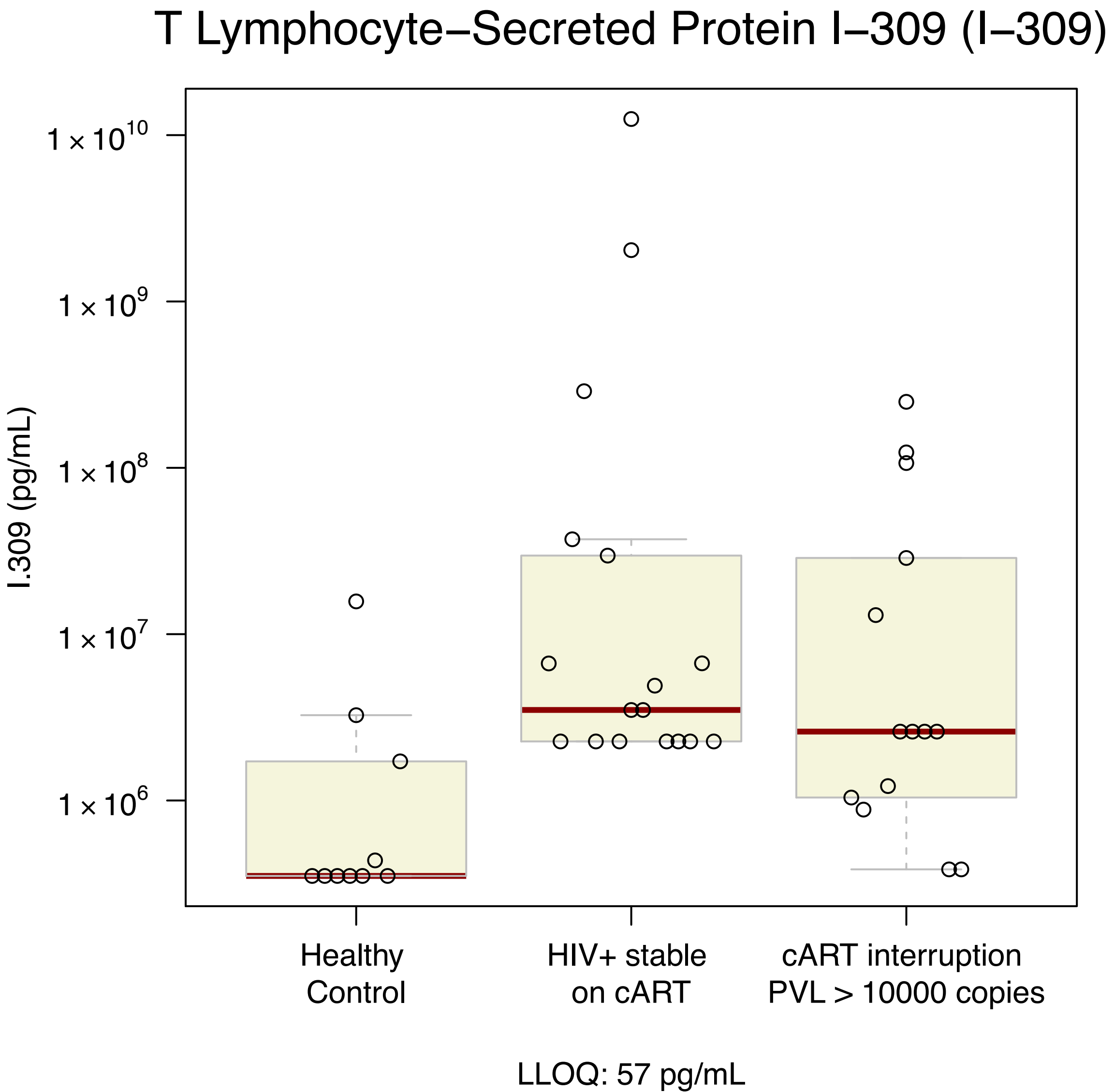
T Lymphocyte−Secreted Protein I−309 (I−309)
I.309 (pg/mL)
1 × 10^10
1 × 10^9
1 × 10^8
1 × 10^7
1 × 10^6
Healthy Control
HIV+ stable on cART
cART interruption PVL > 10000 copies
LLOQ: 57 pg/mL

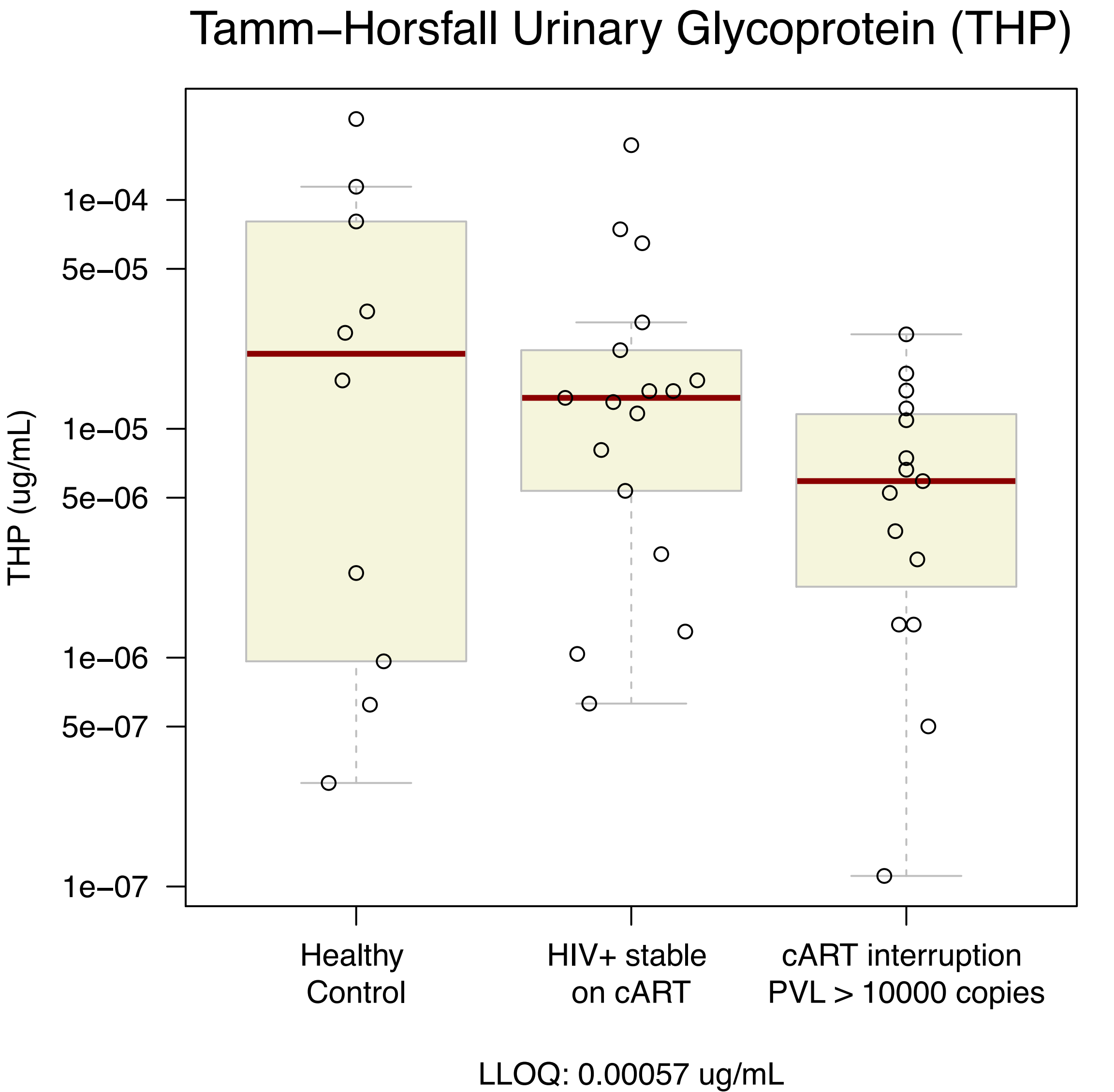

Tamm–Horsfall Urinary Glycoprotein (THP)
THP (ug/mL)
1e−04
5e−05
1e−05
5e−06
1e−06
5e−07
1e−07
Healthy Control
HIV+ stable on cART
cART interruption PVL > 10000 copies
LLOQ: 0.00057 ug/mL

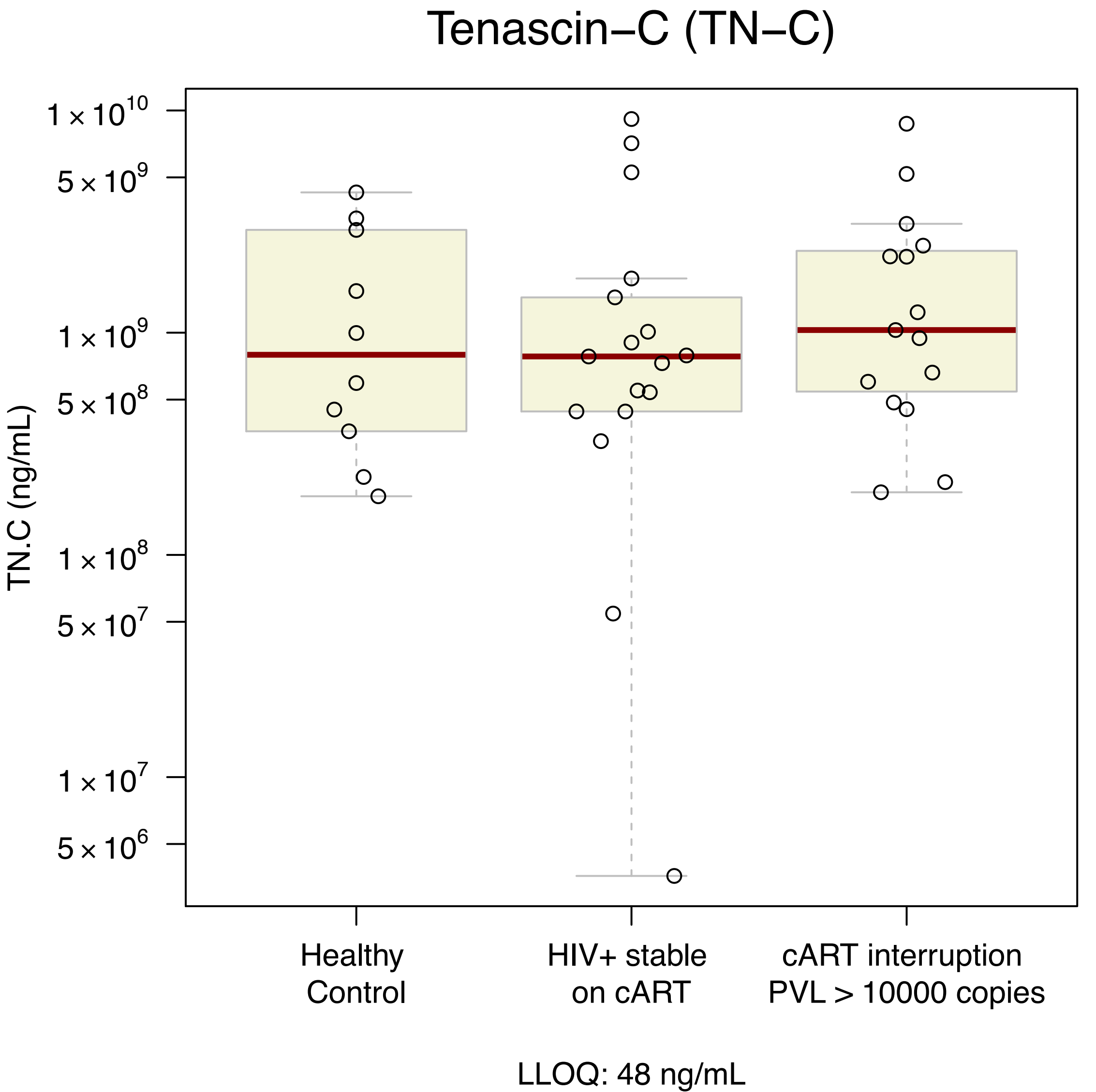

Tenascin−C (TN−C)
TN.C (ng/mL)
1 × 10^10
5 × 10^9
1 × 10^9
5 × 10^8
1 × 10^8
5 × 10^7
1 × 10^7
5 × 10^6
Healthy Control
HIV+ stable on cART
cART interruption PVL > 10000 copies
LLOQ: 48 ng/mL

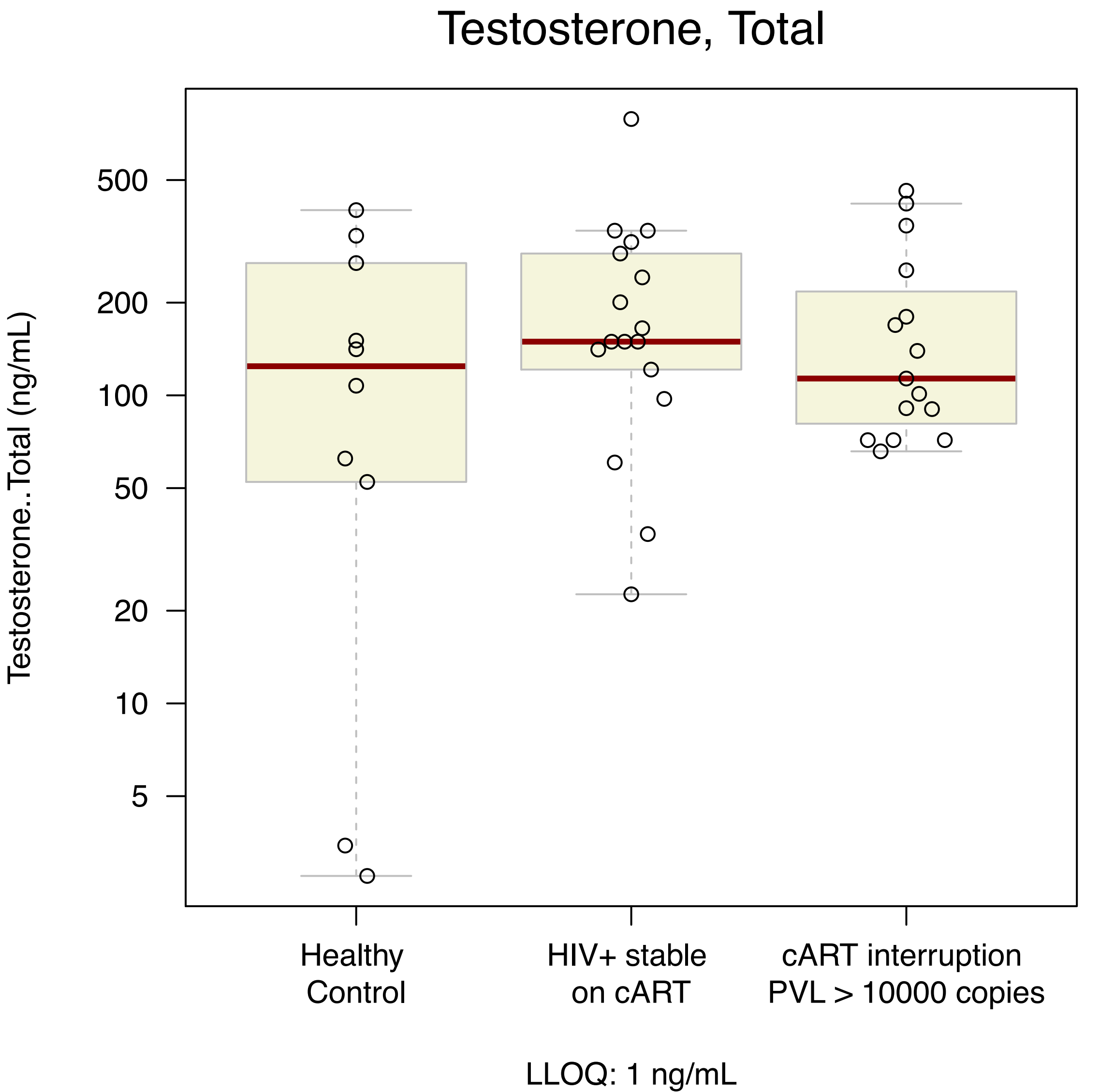
Testosterone, Total
Testosterone..Total (ng/mL)
500
200
100
50
20
10
5
Healthy Control
HIV+ stable on cART
cART interruption PVL > 10000 copies
LLOQ: 1 ng/mL

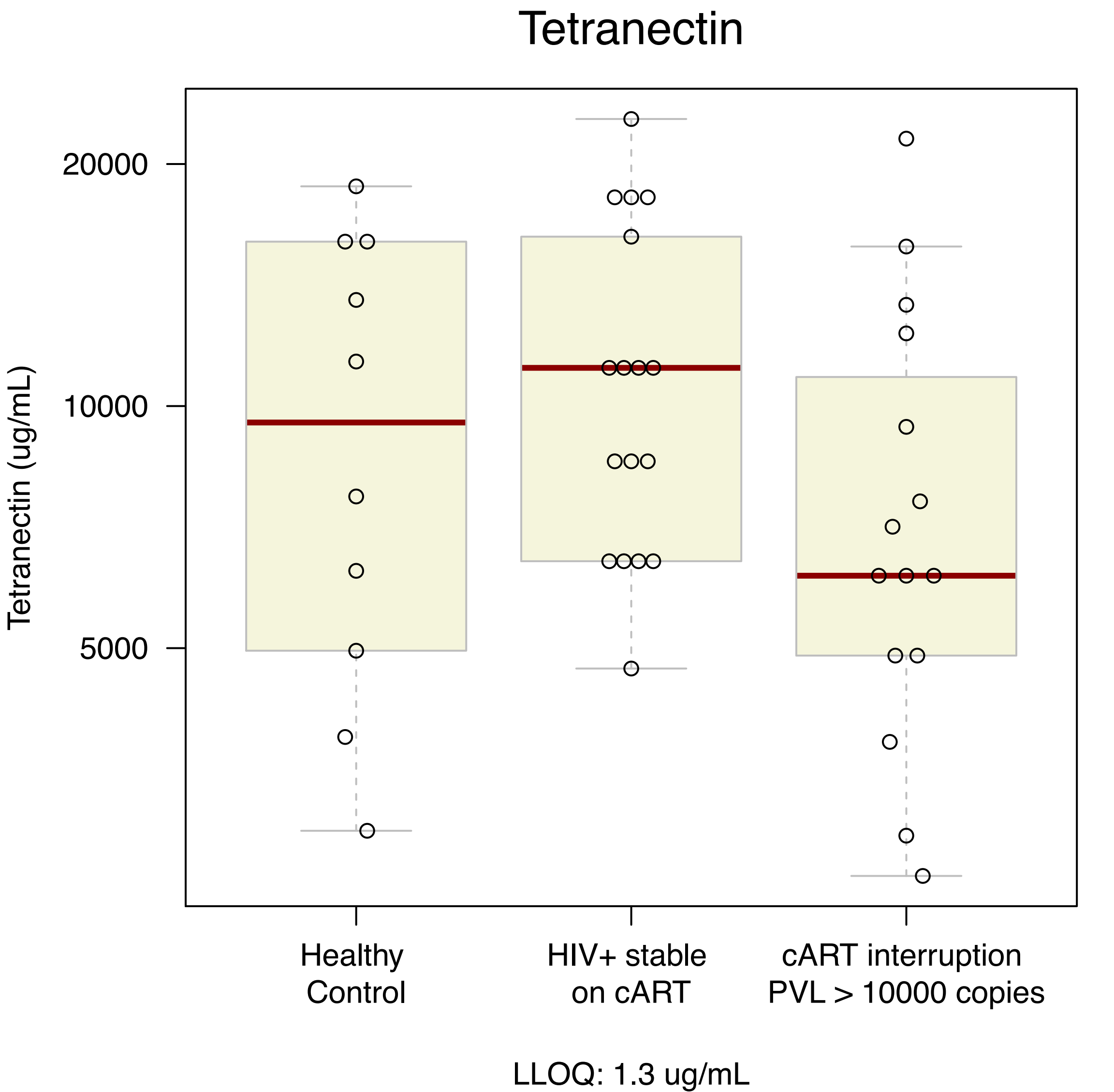
Tetranectin
Tetranectin (ug/mL)
20000
10000
5000
Healthy Control
HIV+ stable on cART
cART interruption PVL > 10000 copies
LLOQ: 1.3 ug/mL

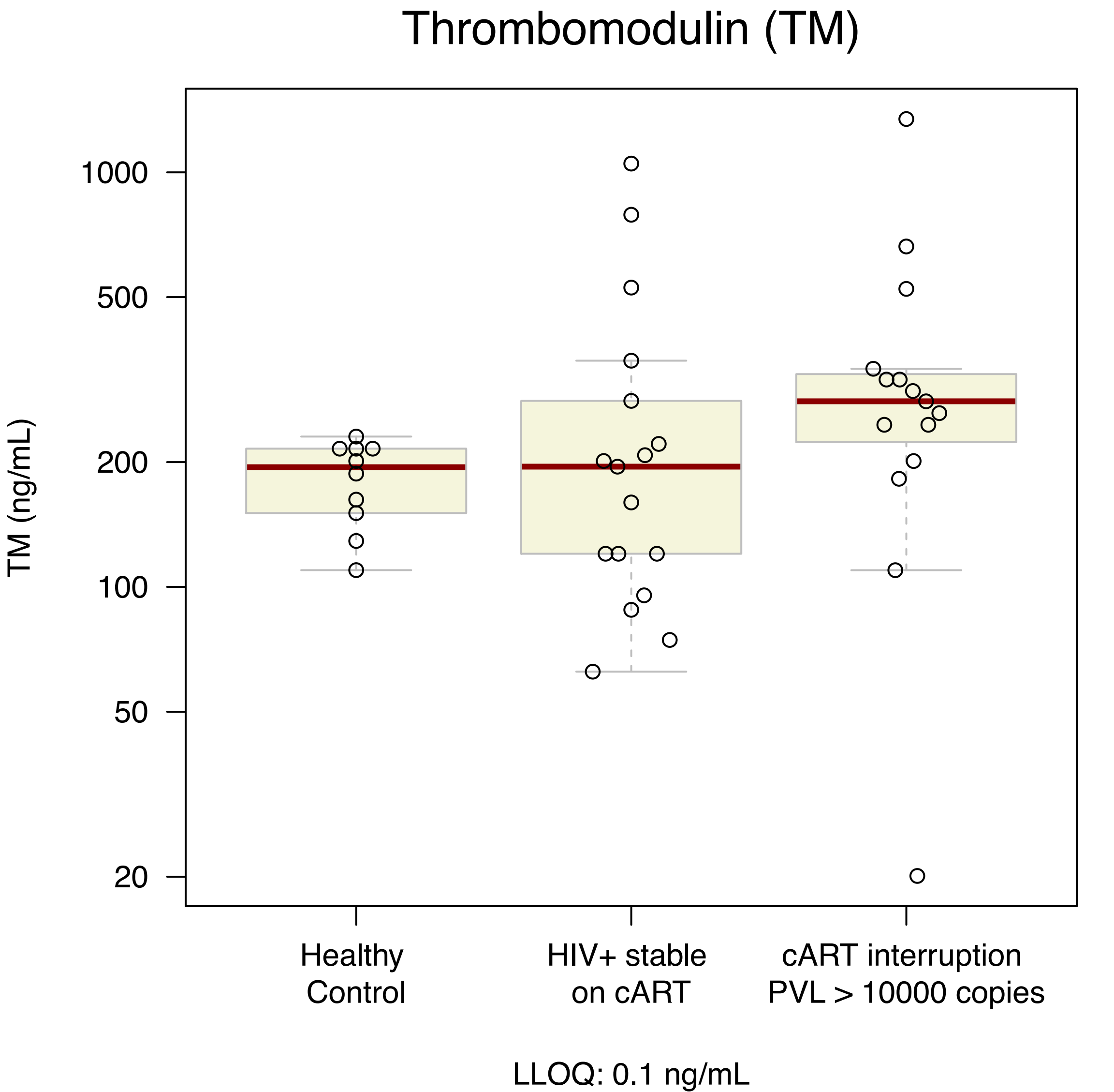
Thrombomodulin (TM)
TM (ng/mL)
1000
500
200
100
50
20
Healthy
Control
HIV+ stable
on cART
cART interruption
PVL > 10000 copies
LLOQ: 0.1 ng/mL

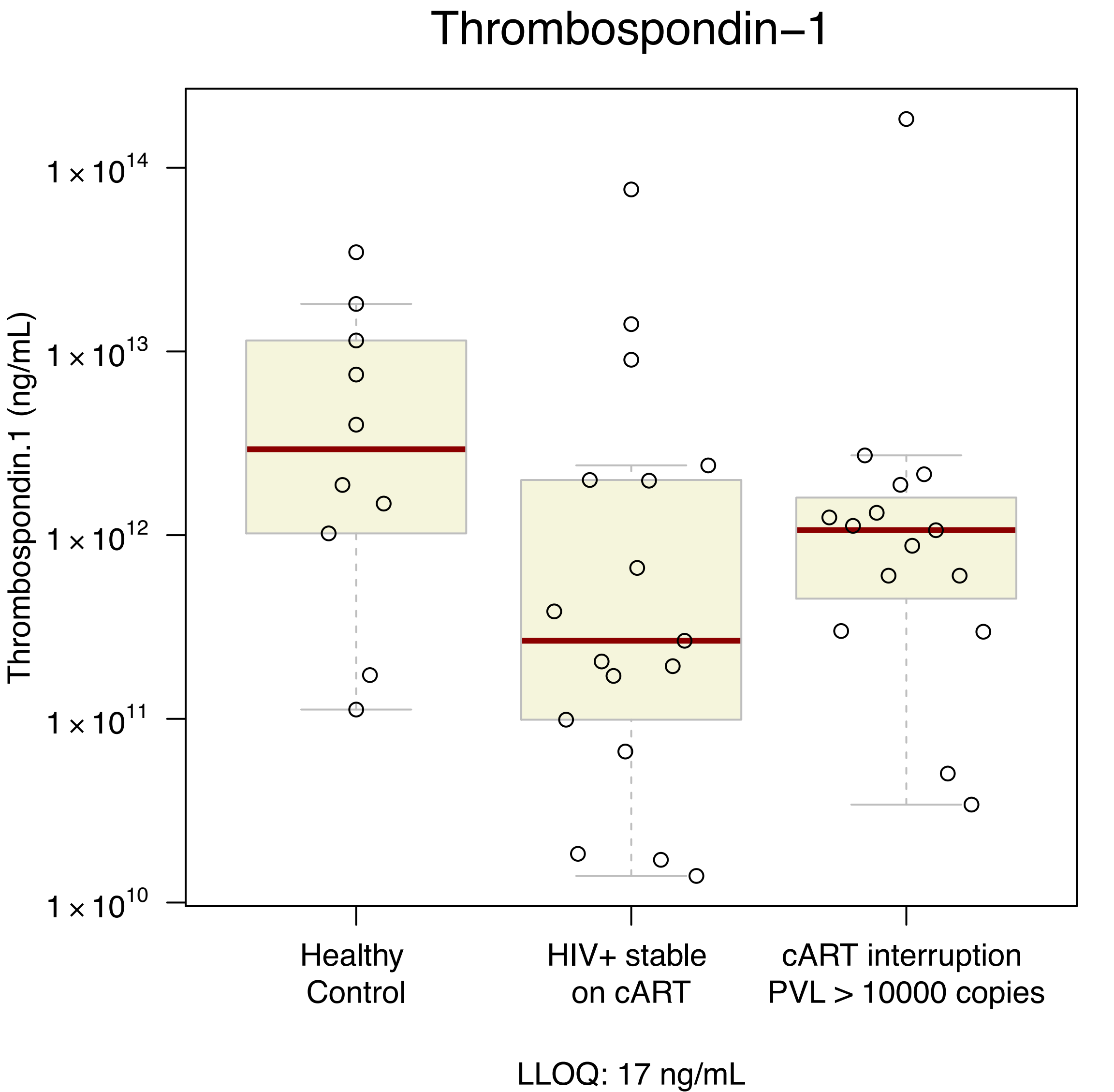

Thrombospondin−1
Thrombospondin.1 (ng/mL)
1×10^{14}
1×10^{13}
1×10^{12}
1×10^{11}
1×10^{10}
Healthy Control
HIV+ stable on cART
cART interruption PVL > 10000 copies
LLOQ: 17 ng/mL

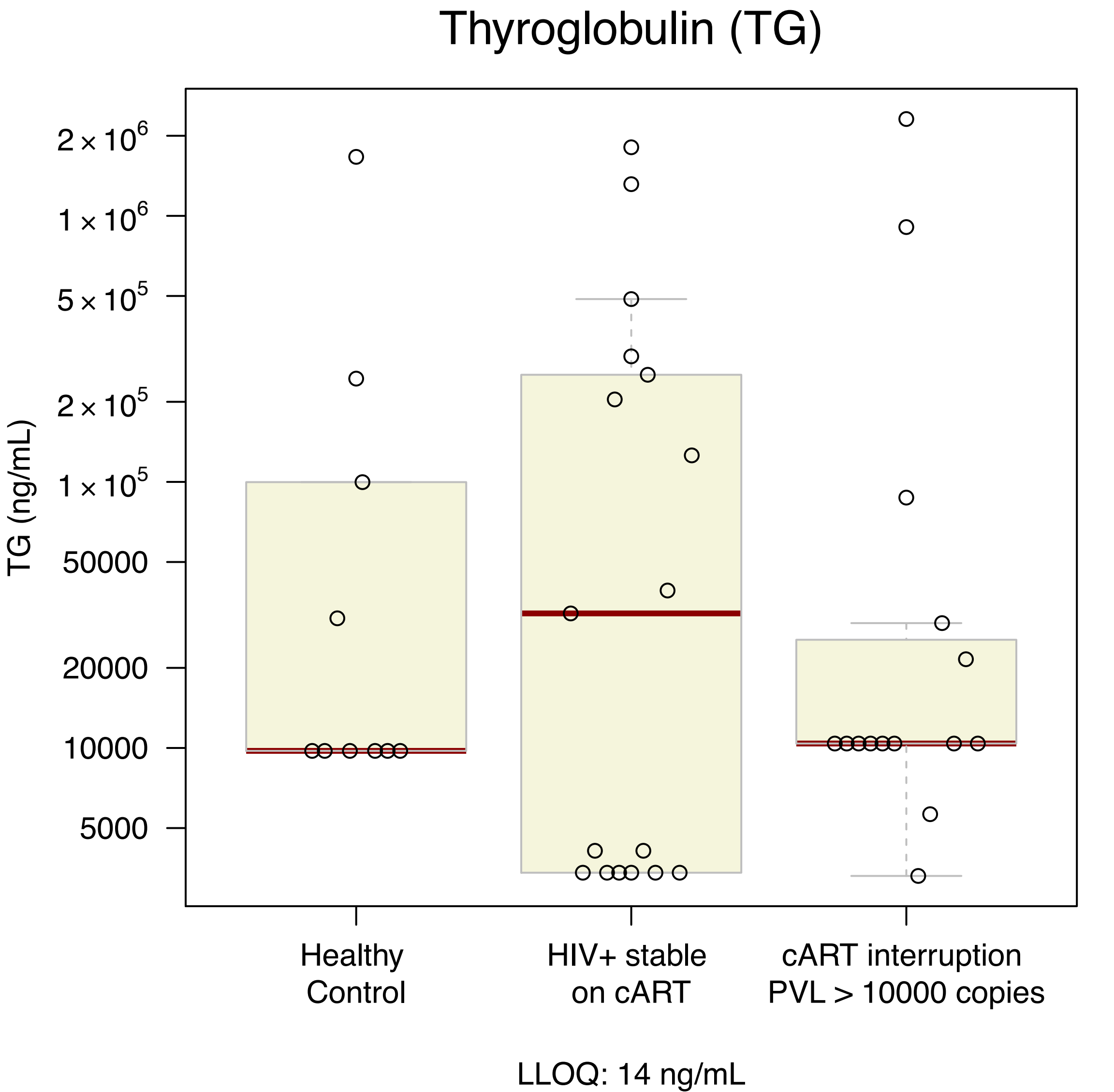
Thyroglobulin (TG)
TG (ng/mL)
2 × 10^6
1 × 10^6
5 × 10^5
2 × 10^5
1 × 10^5
50000
20000
10000
5000
Healthy Control
HIV+ stable on cART
cART interruption PVL > 10000 copies
LLOQ: 14 ng/mL

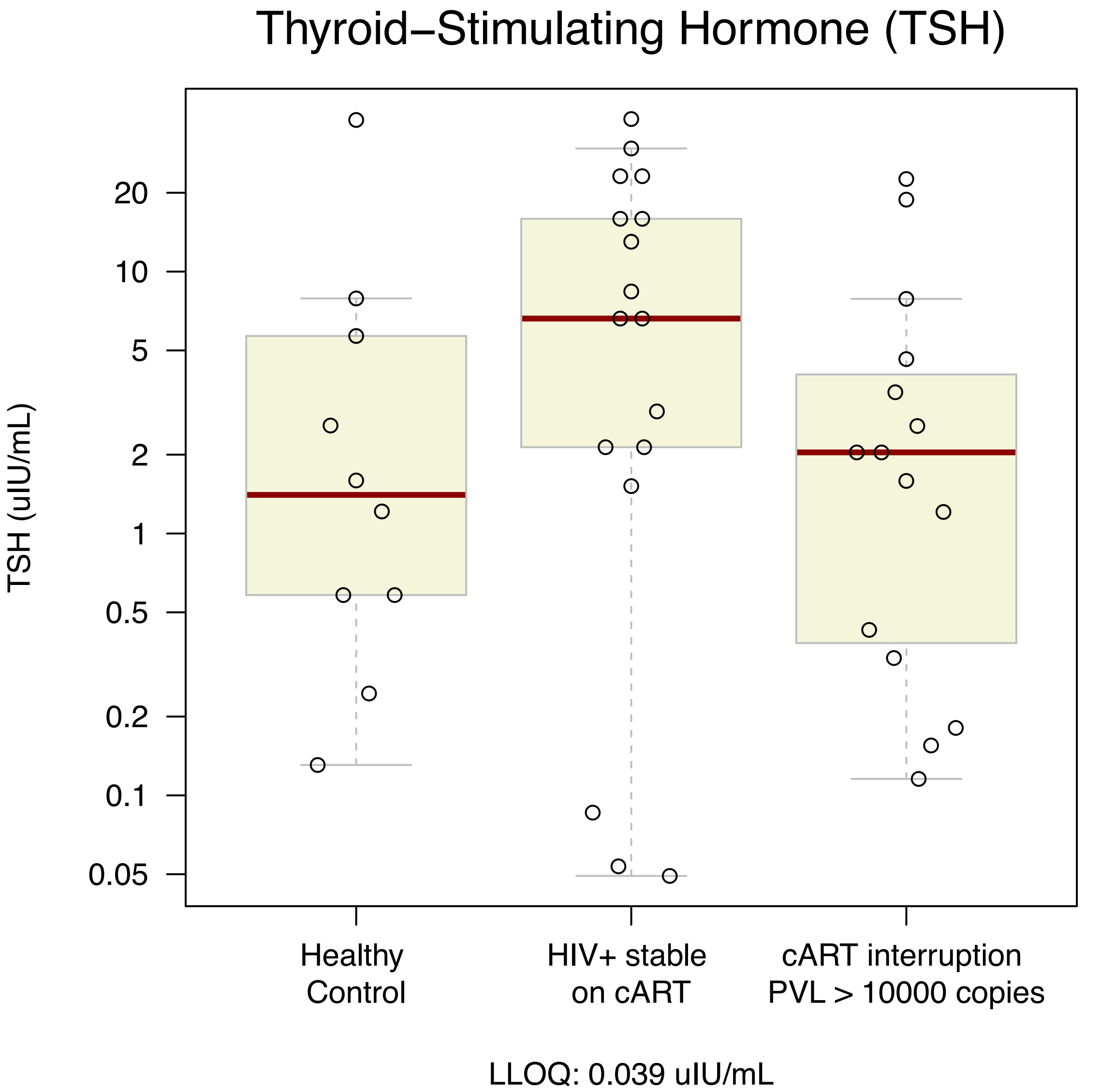

Thyroid–Stimulating Hormone (TSH)
TSH (uIU/mL)
20
10
5
2
1
0.5
0.2
0.1
0.05
Healthy Control
HIV+ stable on cART
cART interruption PVL > 10000 copies
LLOQ: 0.039 uIU/mL

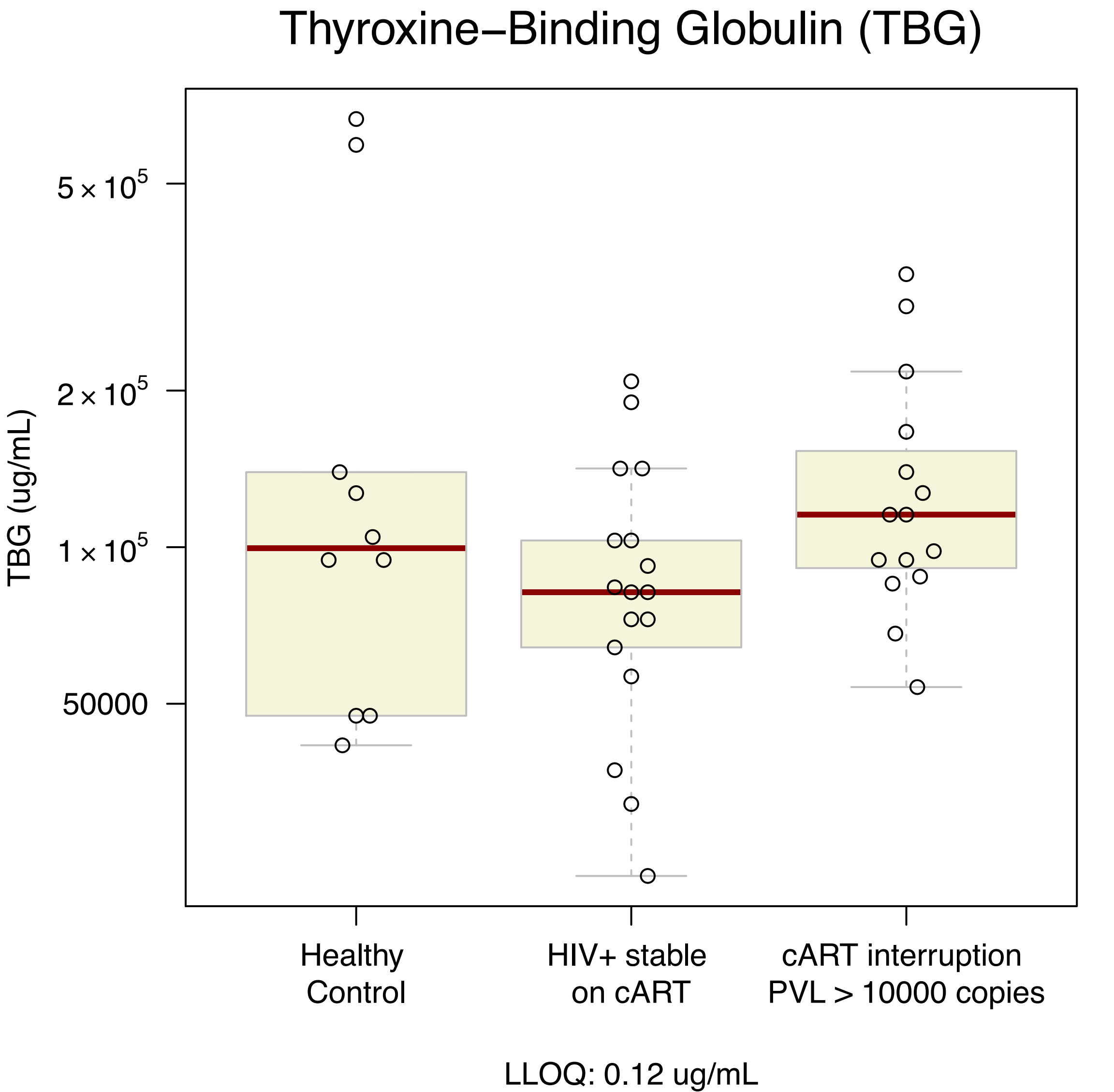
Thyroxine–Binding Globulin (TBG)
TBG (ug/mL)
5 × 10^5
2 × 10^5
1 × 10^5
50000
Healthy Control
HIV+ stable on cART
cART interruption PVL > 10000 copies
LLOQ: 0.12 ug/mL

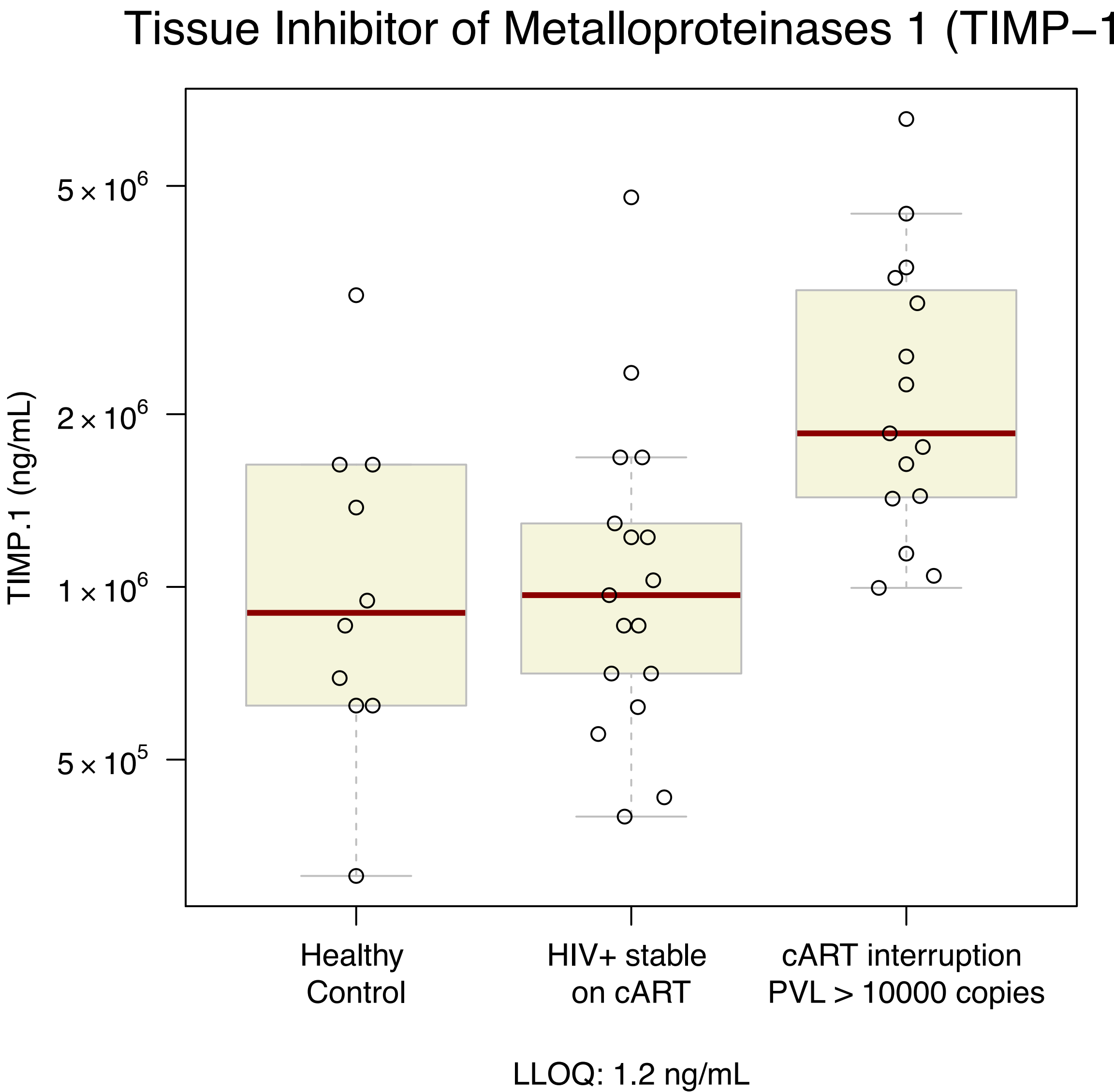

Tissue Inhibitor of Metalloproteinases 1 (TIMP−1
TIMP.1 (ng/mL)
5 × 10^6
2 × 10^6
1 × 10^6
5 × 10^5
Healthy Control
HIV+ stable on cART
cART interruption PVL > 10000 copies
LLOQ: 1.2 ng/mL

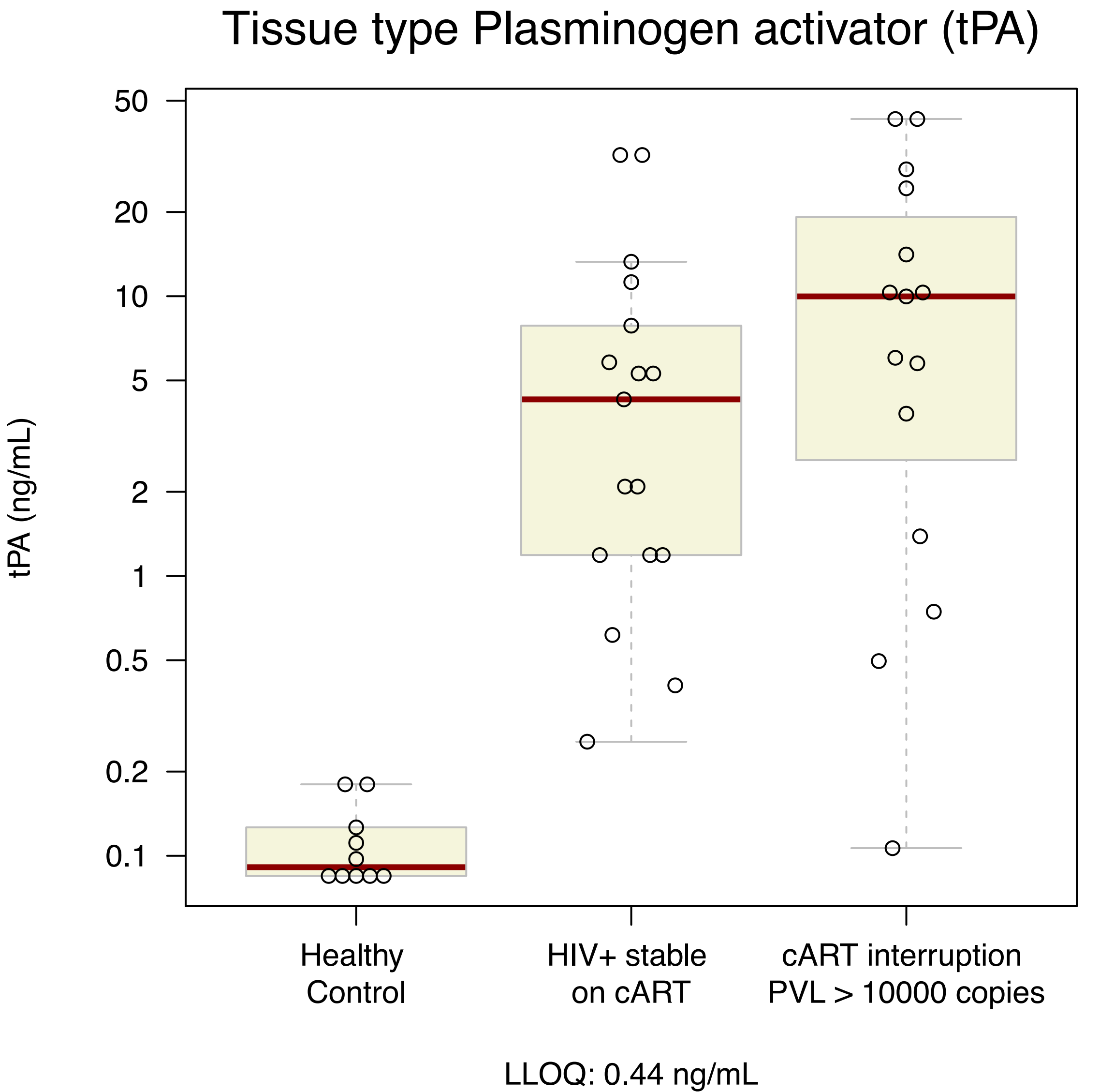
Tissue type Plasminogen activator (tPA)
tPA (ng/mL)
50
20
10
5
2
1
0.5
0.2
0.1
Healthy Control
HIV+ stable on cART
cART interruption PVL > 10000 copies
LLOQ: 0.44 ng/mL

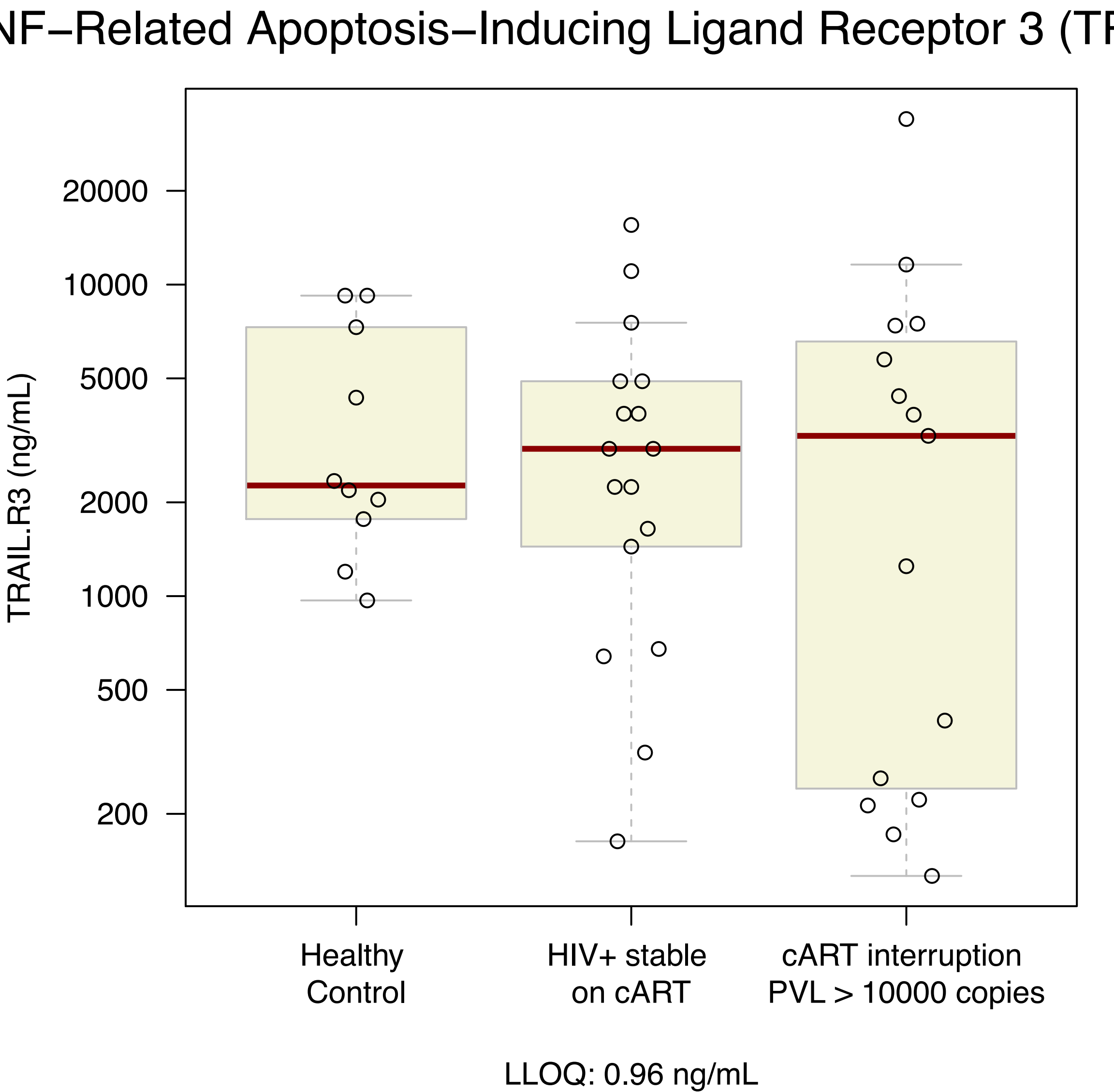

NF−Related Apoptosis−Inducing Ligand Receptor 3 (TF
TRAIL.R3 (ng/mL)
20000
10000
5000
2000
1000
500
200
Healthy Control
HIV+ stable on cART
cART interruption PVL > 10000 copies
LLOQ: 0.96 ng/mL

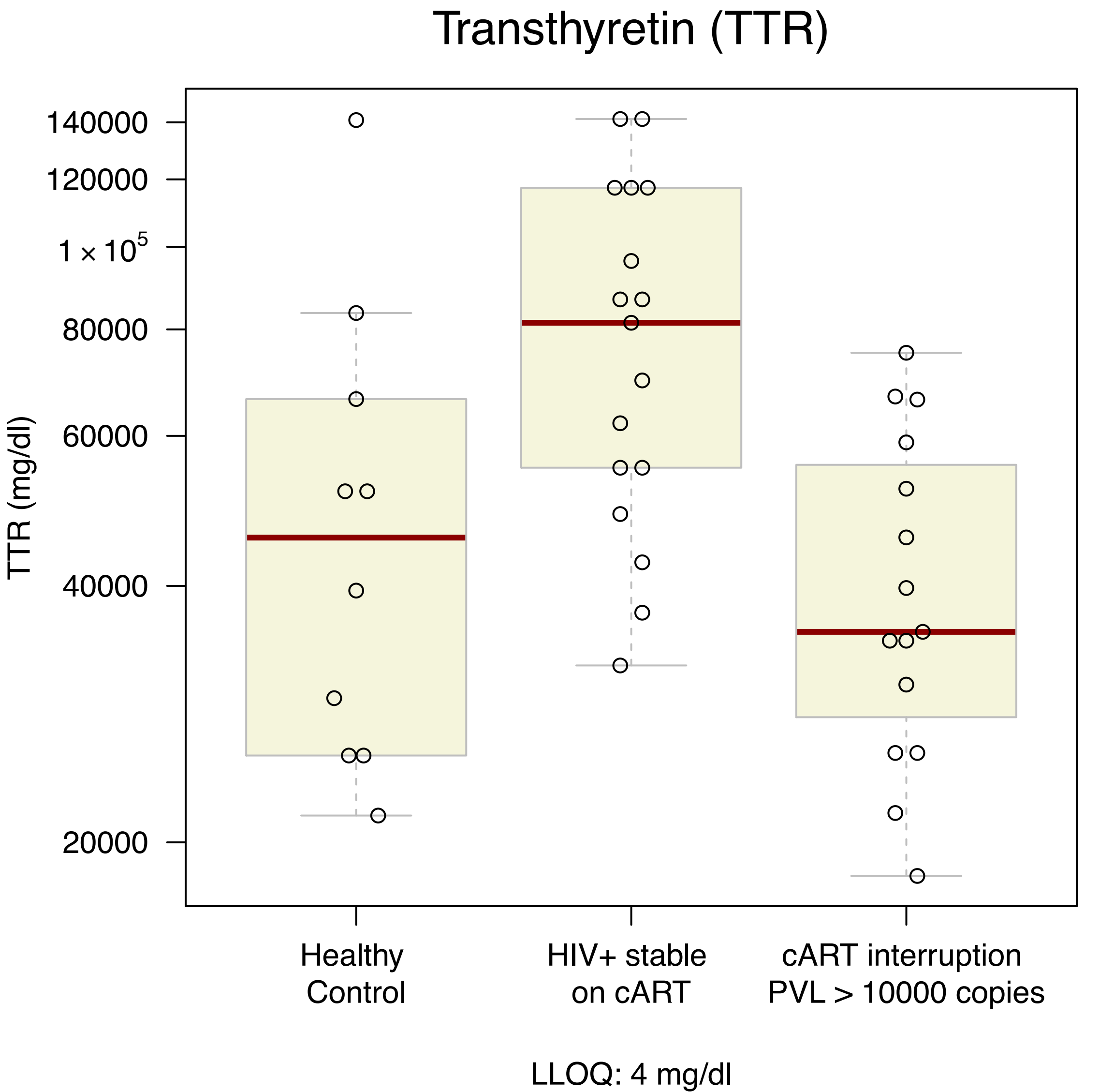
Transthyretin (TTR)
TTR (mg/dl)
140000
120000
1×10^5
80000
60000
40000
20000
Healthy Control
HIV+ stable on cART
cART interruption PVL > 10000 copies
LLOQ: 4 mg/dl

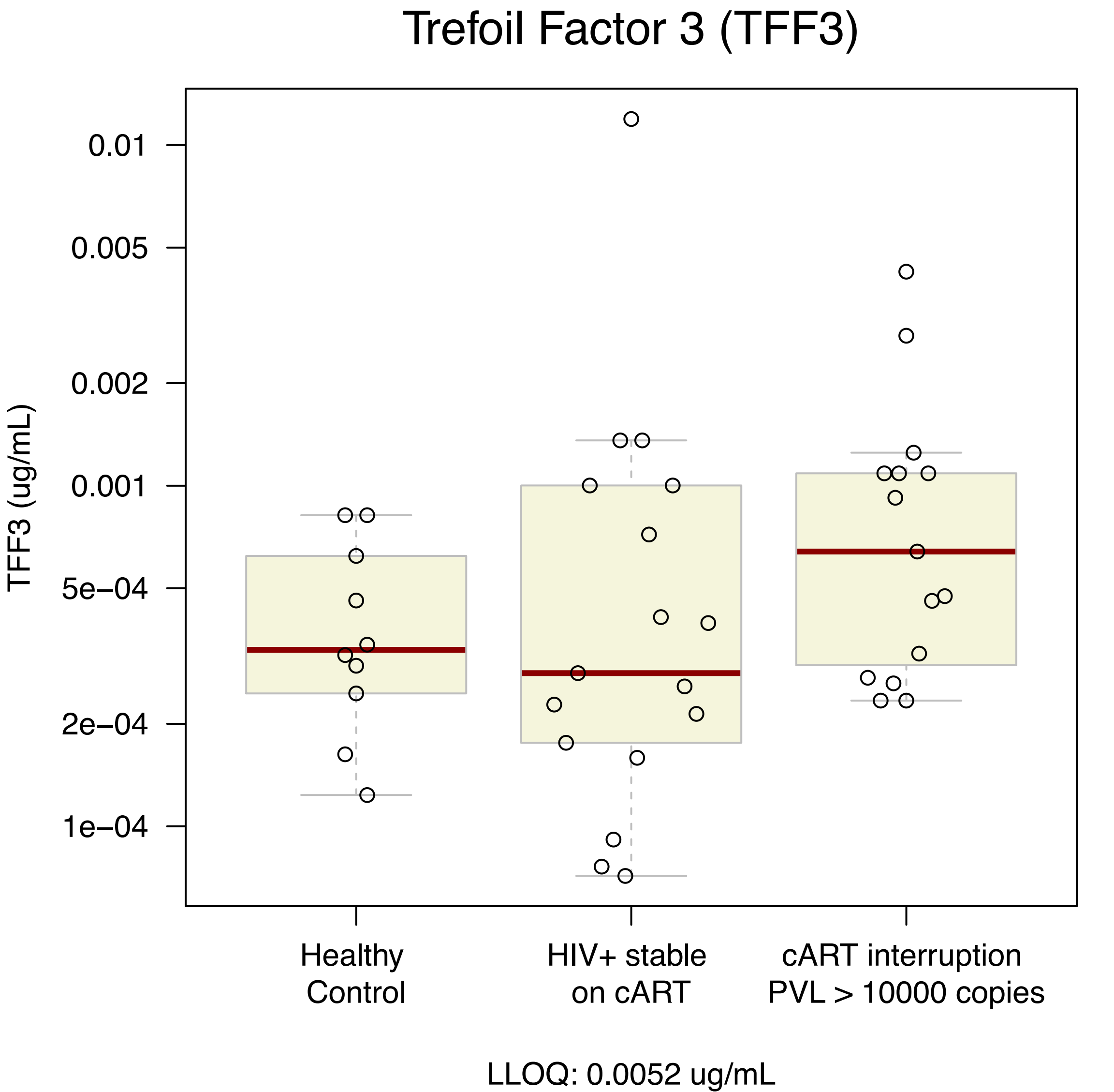

Trefoil Factor 3 (TFF3)
TFF3 (ug/mL)
0.01
0.005
0.002
0.001
5e−04
2e−04
1e−04
Healthy
Control
HIV+ stable
on cART
cART interruption
PVL > 10000 copies
LLOQ: 0.0052 ug/mL

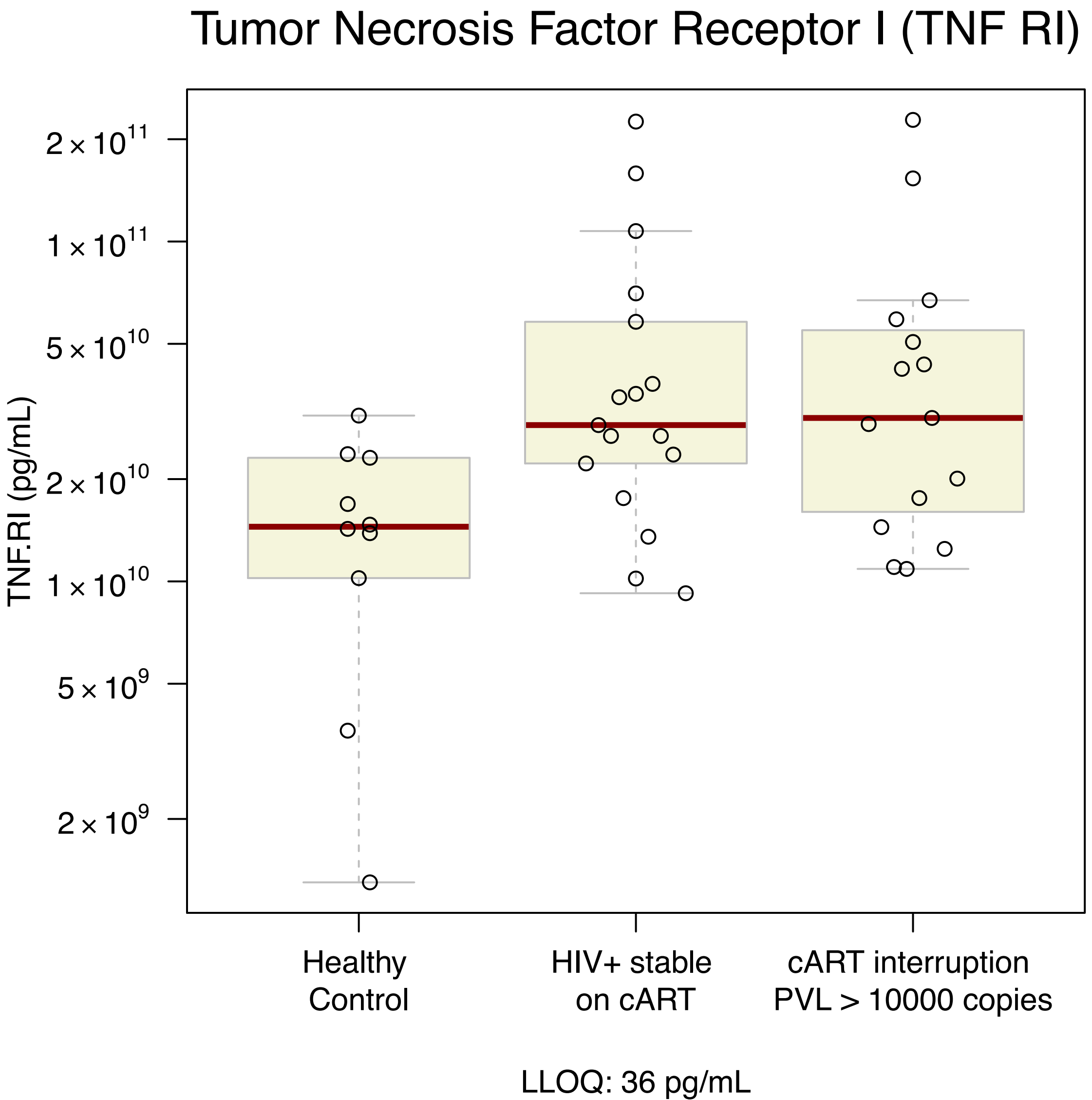
Tumor Necrosis Factor Receptor I (TNF RI)
TNF.RI (pg/mL)
2 × 10^11
1 × 10^11
5 × 10^10
2 × 10^10
1 × 10^10
5 × 10^9
2 × 10^9
Healthy Control
HIV+ stable on cART
cART interruption PVL > 10000 copies
LLOQ: 36 pg/mL

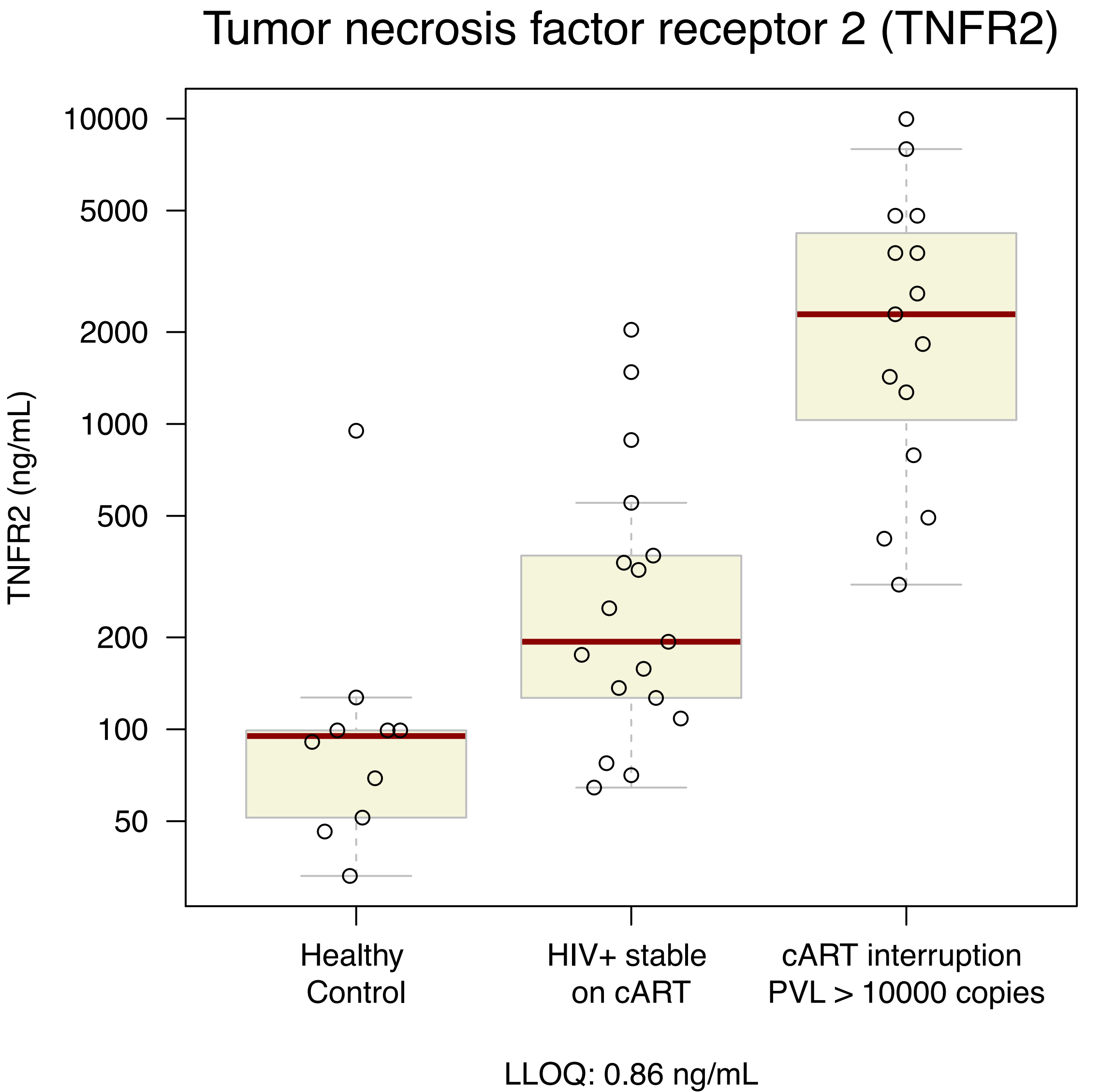

Tumor necrosis factor receptor 2 (TNFR2)
TNFR2 (ng/mL)
10000
5000
2000
1000
500
200
100
50
Healthy Control
HIV+ stable on cART
cART interruption PVL > 10000 copies
LLOQ: 0.86 ng/mL

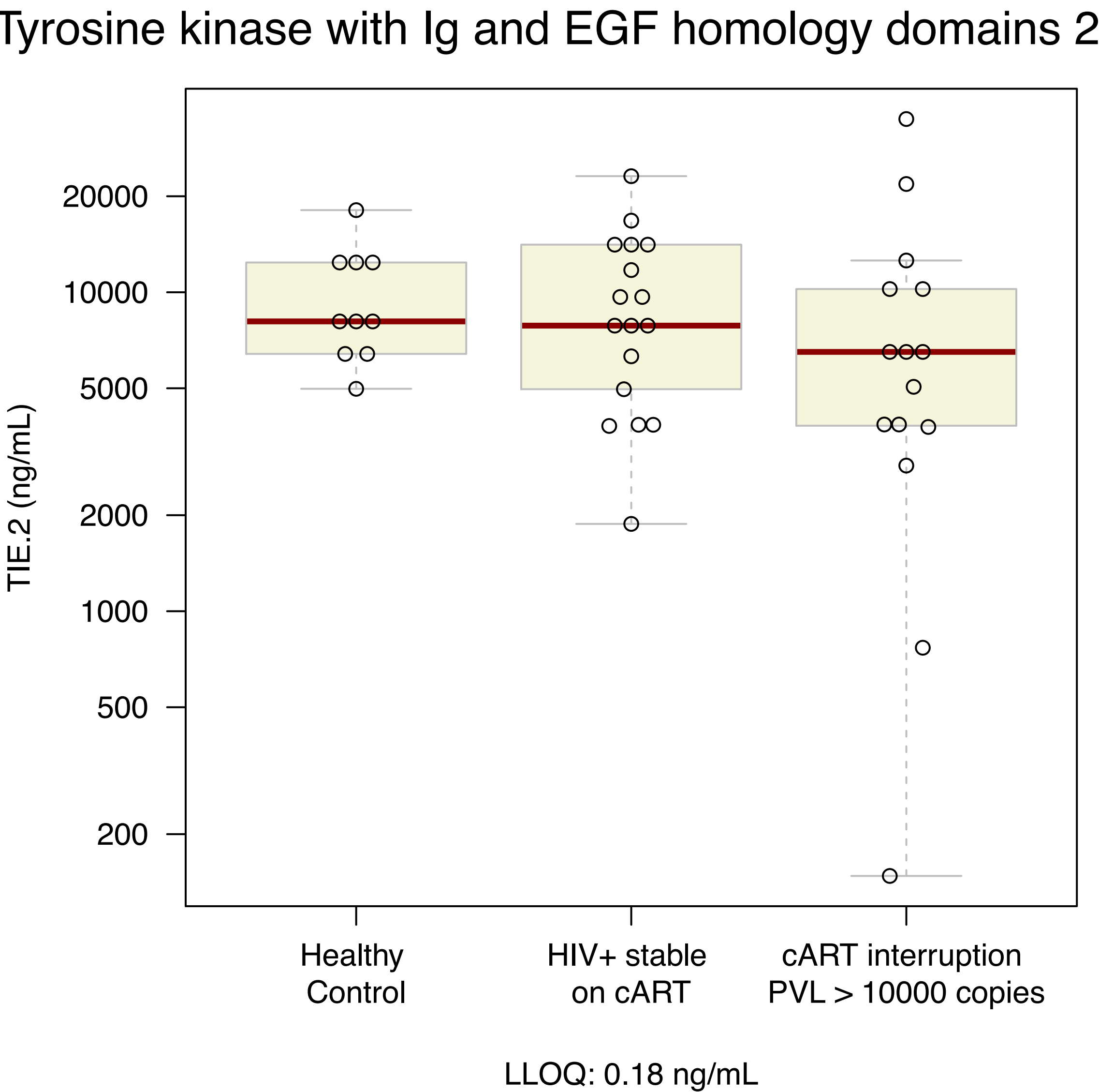
Tyrosine kinase with Ig and EGF homology domains 2
TIE.2 (ng/mL)
20000
10000
5000
2000
1000
500
200
Healthy Control
HIV+ stable on cART
cART interruption PVL > 10000 copies
LLOQ: 0.18 ng/mL

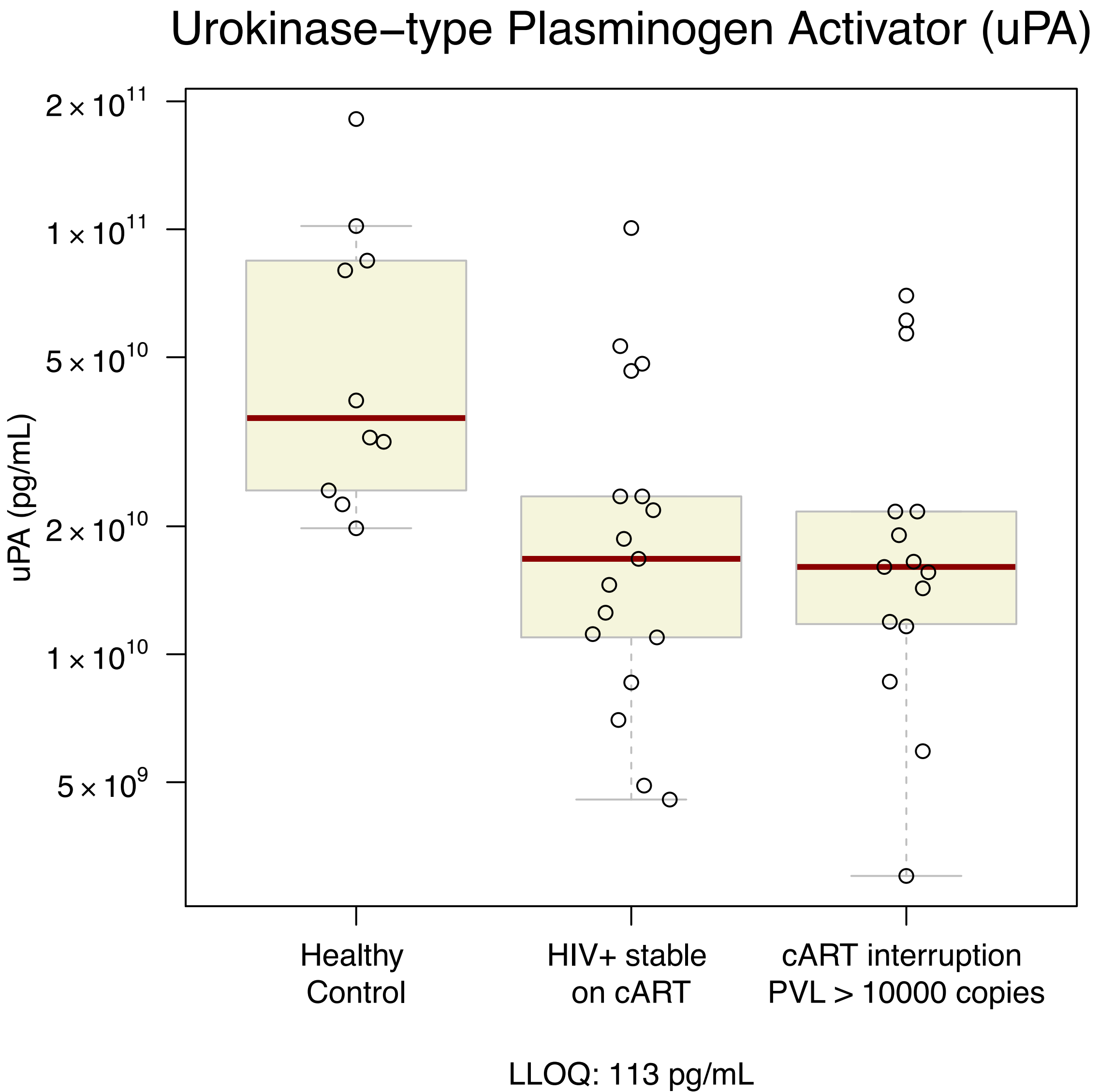

Urokinase–type Plasminogen Activator (uPA)
uPA (pg/mL)
2×10^{11}
1×10^{11}
5×10^{10}
2×10^{10}
1×10^{10}
5×10^{9}
Healthy Control
HIV+ stable on cART
cART interruption PVL > 10000 copies
LLOQ: 113 pg/mL

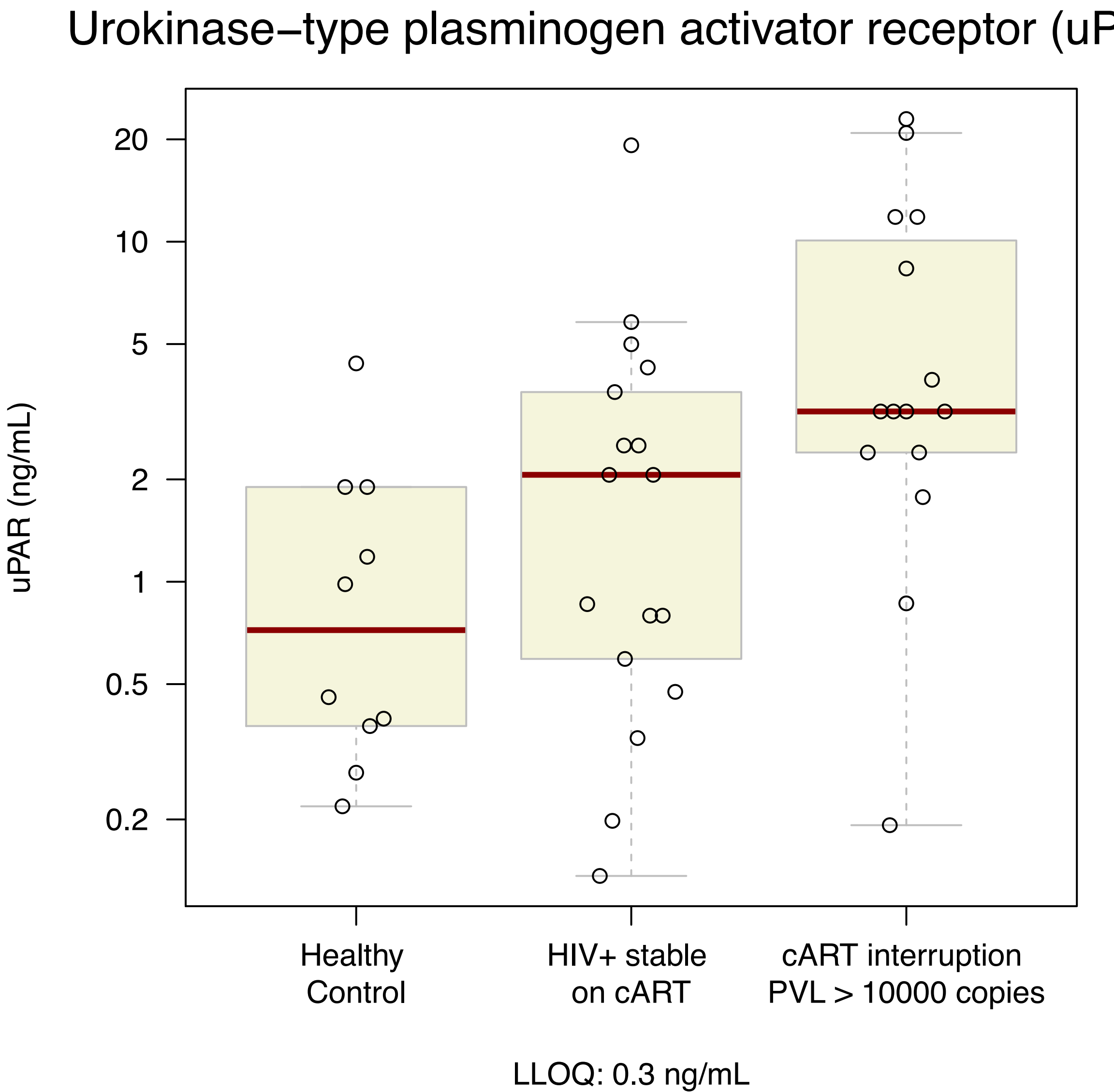

Urokinase−type plasminogen activator receptor (uP
uPAR (ng/mL)
20
10
5
2
1
0.5
0.2
Healthy
Control
HIV+ stable
on cART
cART interruption
PVL > 10000 copies
LLOQ: 0.3 ng/mL

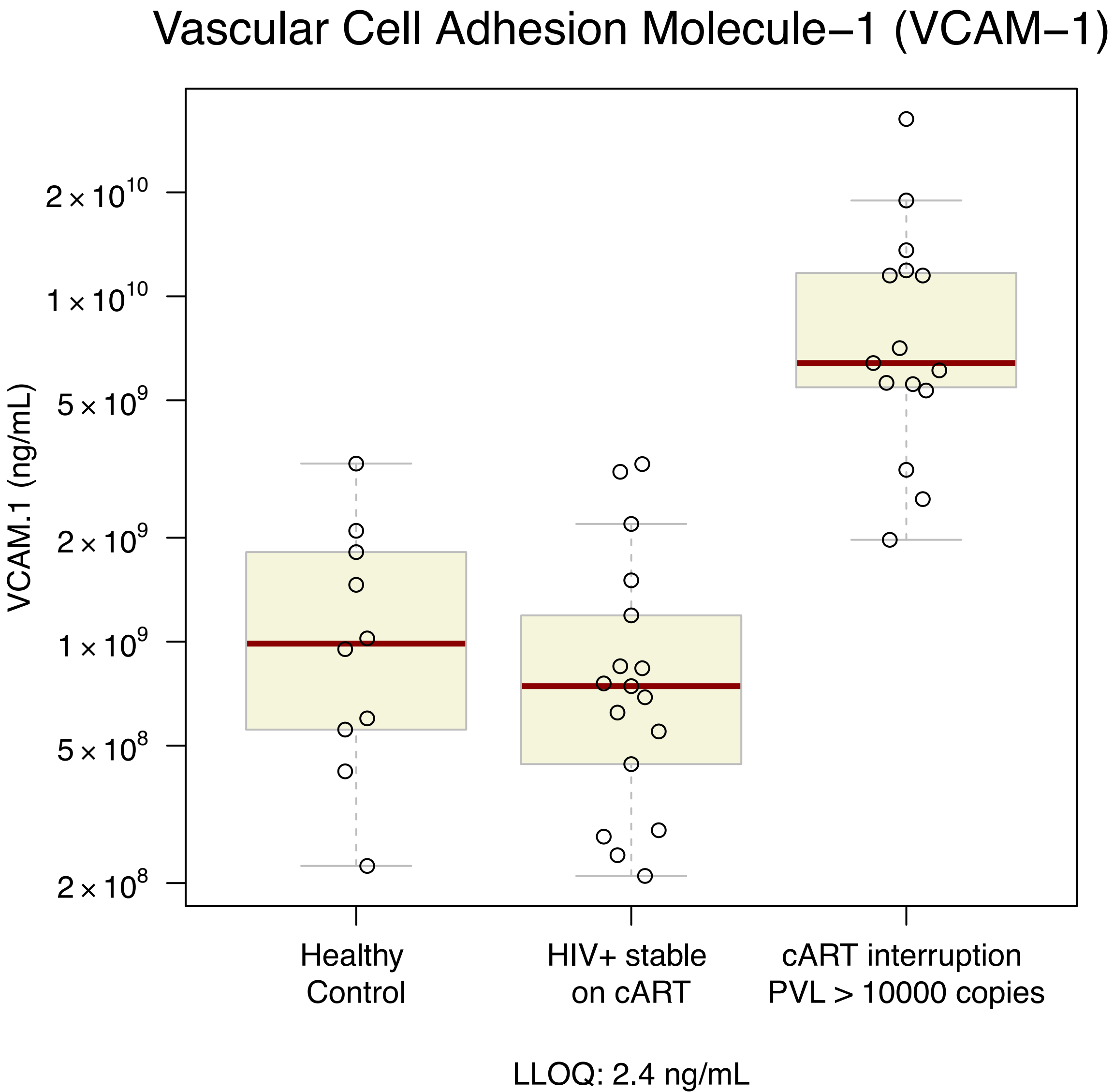

Vascular Cell Adhesion Molecule−1 (VCAM−1)
VCAM.1 (ng/mL)
2×10^{10}
1×10^{10}
5×10^{9}
2×10^{9}
1×10^{9}
5×10^{8}
2×10^{8}
Healthy Control
HIV+ stable on cART
cART interruption PVL > 10000 copies
LLOQ: 2.4 ng/mL

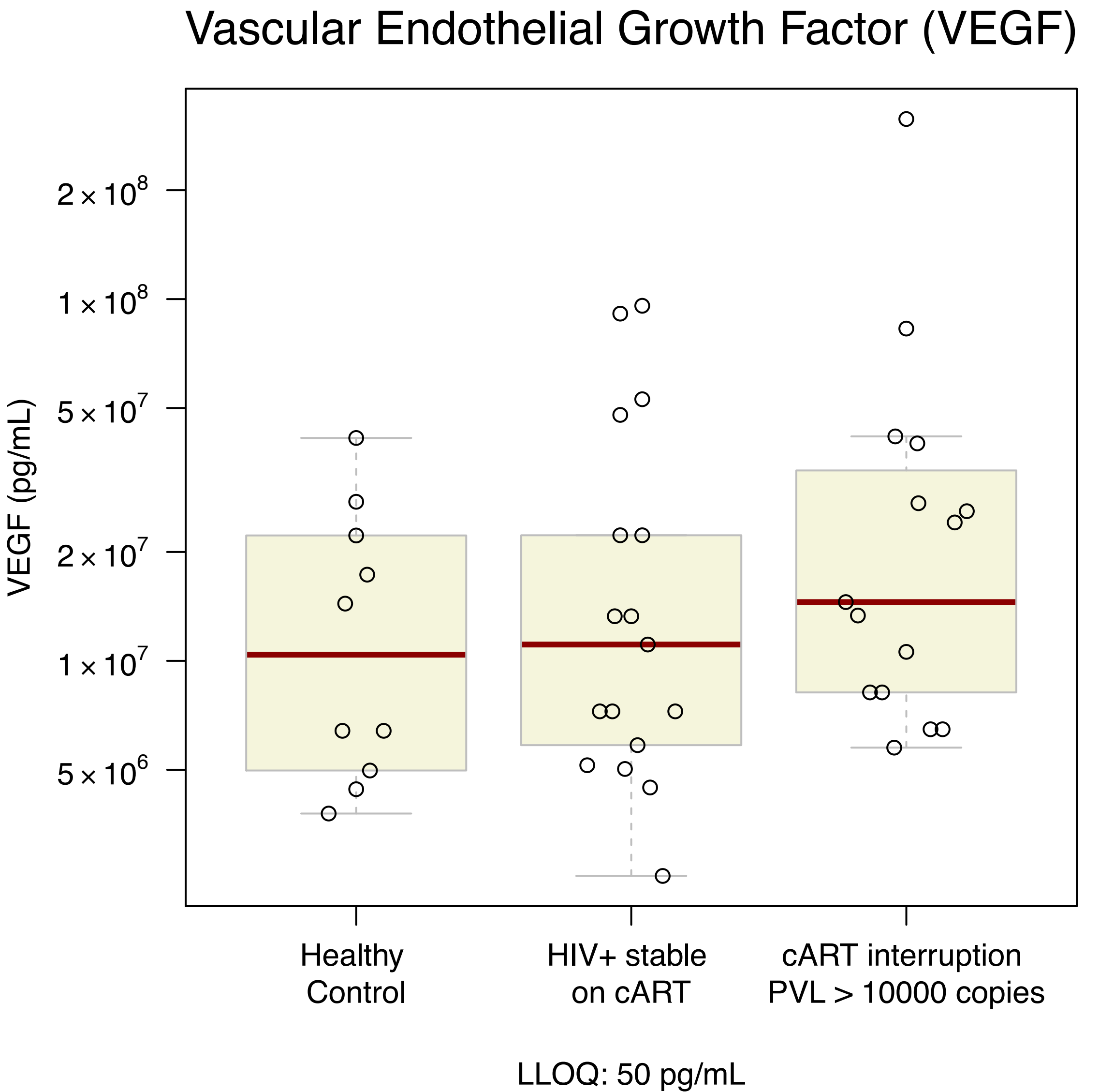

Vascular Endothelial Growth Factor (VEGF)
VEGF (pg/mL)
2×10^8
1×10^8
5×10^7
2×10^7
1×10^7
5×10^6
Healthy Control
HIV+ stable on cART
cART interruption PVL > 10000 copies
LLOQ: 50 pg/mL

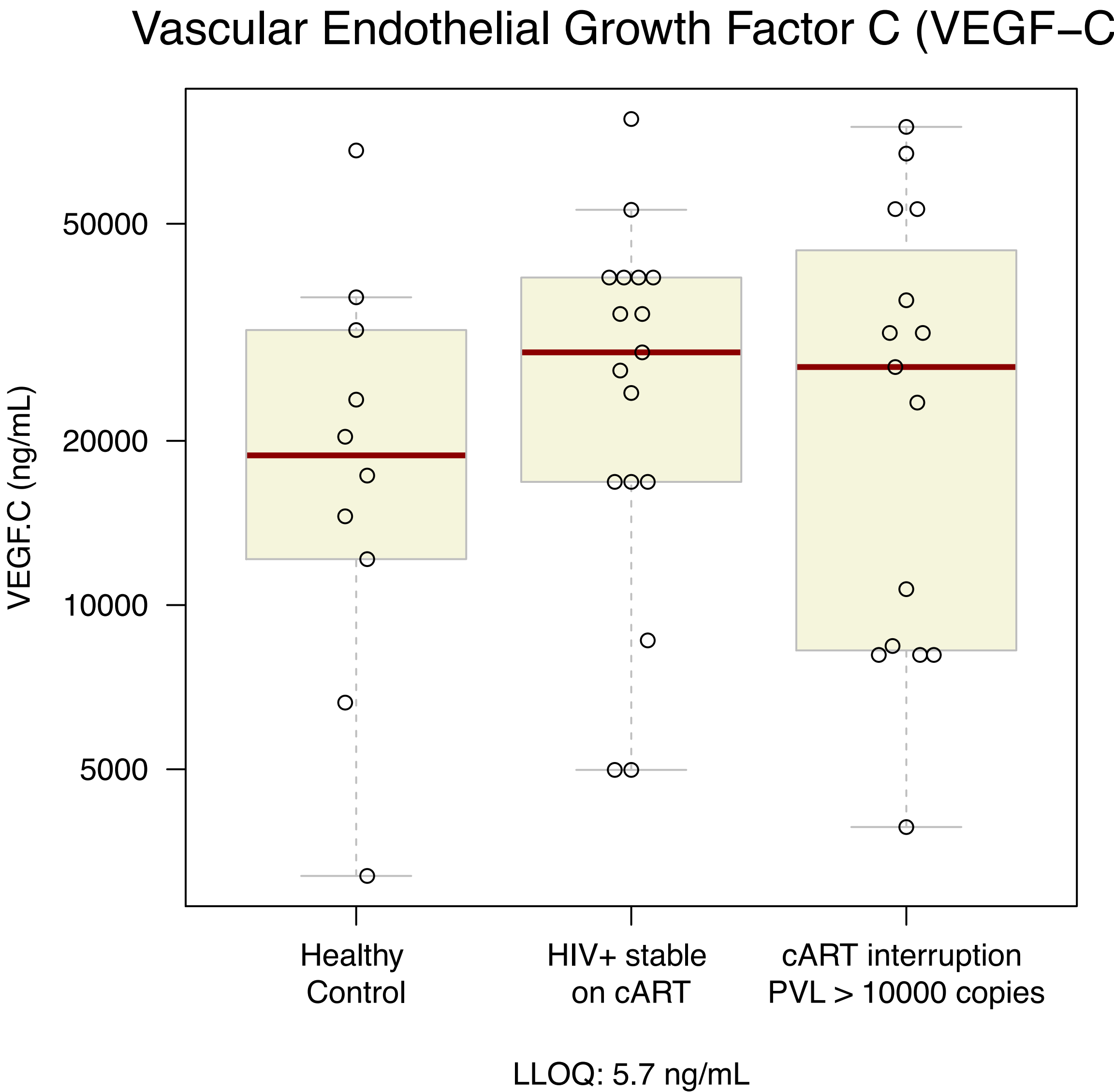

Vascular Endothelial Growth Factor C (VEGF−C
VEGF.C (ng/mL)
50000
20000
10000
5000
Healthy Control
HIV+ stable on cART
cART interruption PVL > 10000 copies
LLOQ: 5.7 ng/mL

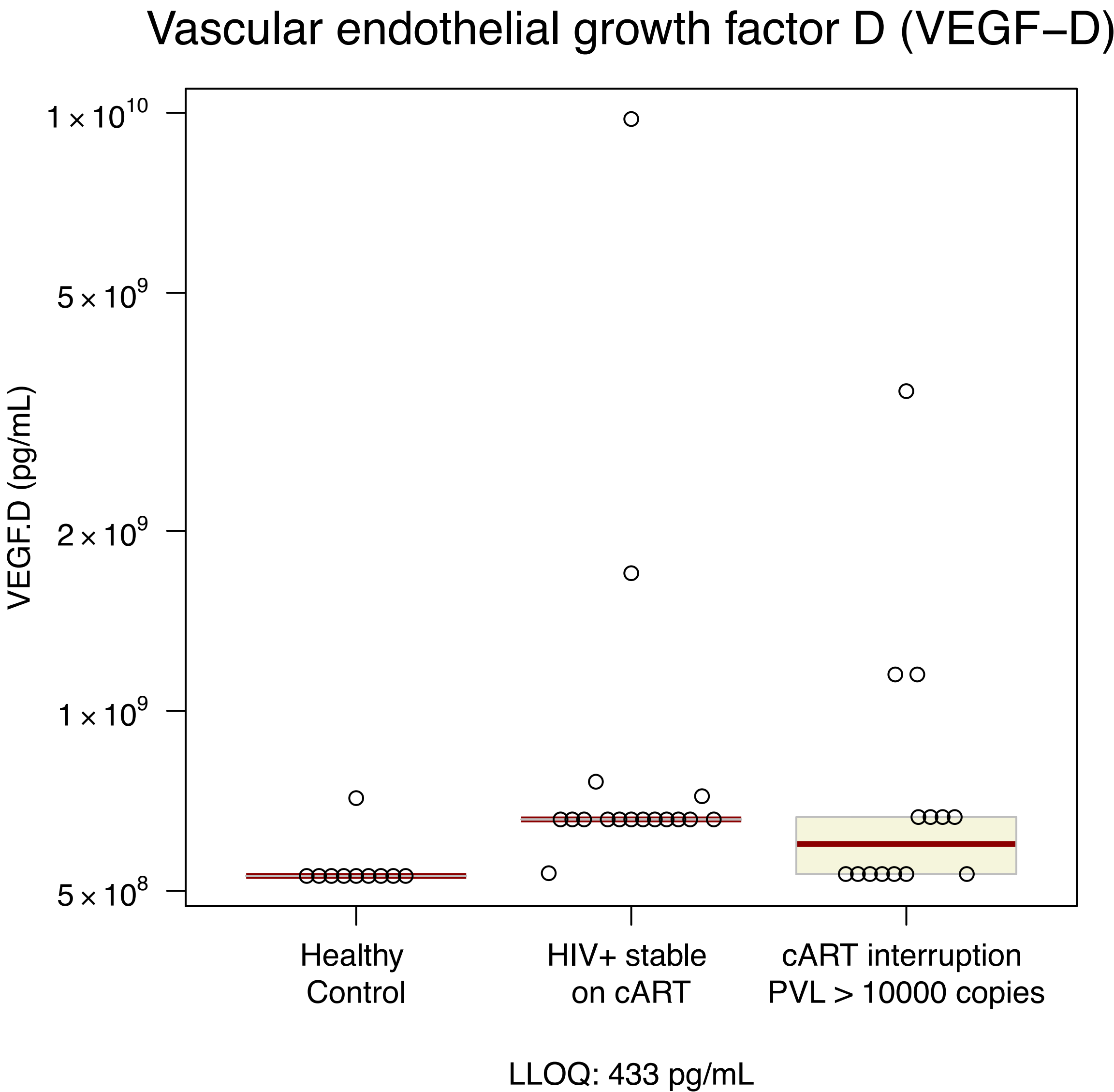
Vascular endothelial growth factor D (VEGF−D)
VEGF.D (pg/mL)
1 × 10^10
5 × 10^9
2 × 10^9
1 × 10^9
5 × 10^8
Healthy Control
HIV+ stable on cART
cART interruption PVL > 10000 copies
LLOQ: 433 pg/mL

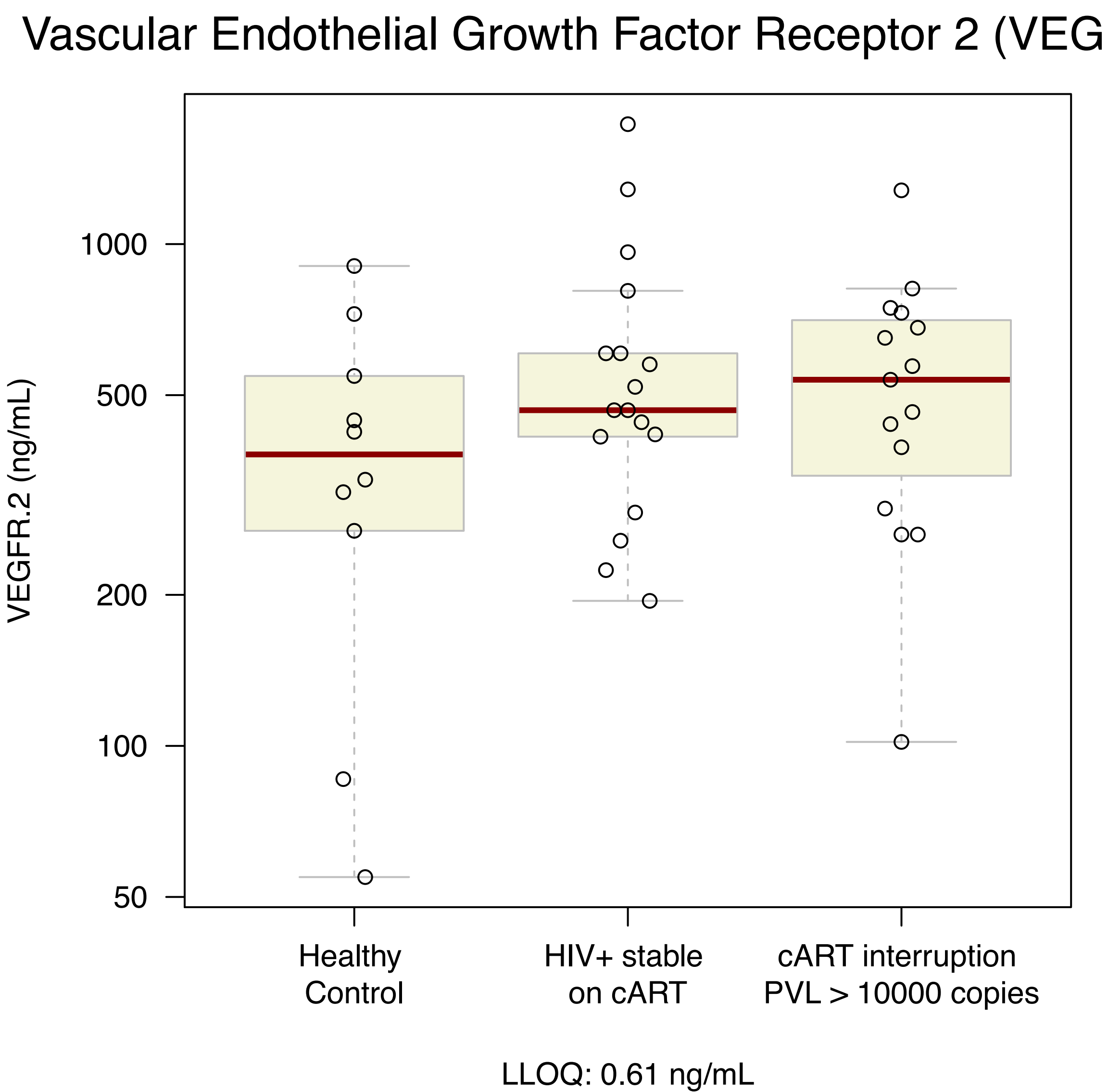

Vascular Endothelial Growth Factor Receptor 2 (VEG
VEGFR.2 (ng/mL)
1000
500
200
100
50
Healthy
Control
HIV+ stable
on cART
cART interruption
PVL > 10000 copies
LLOQ: 0.61 ng/mL

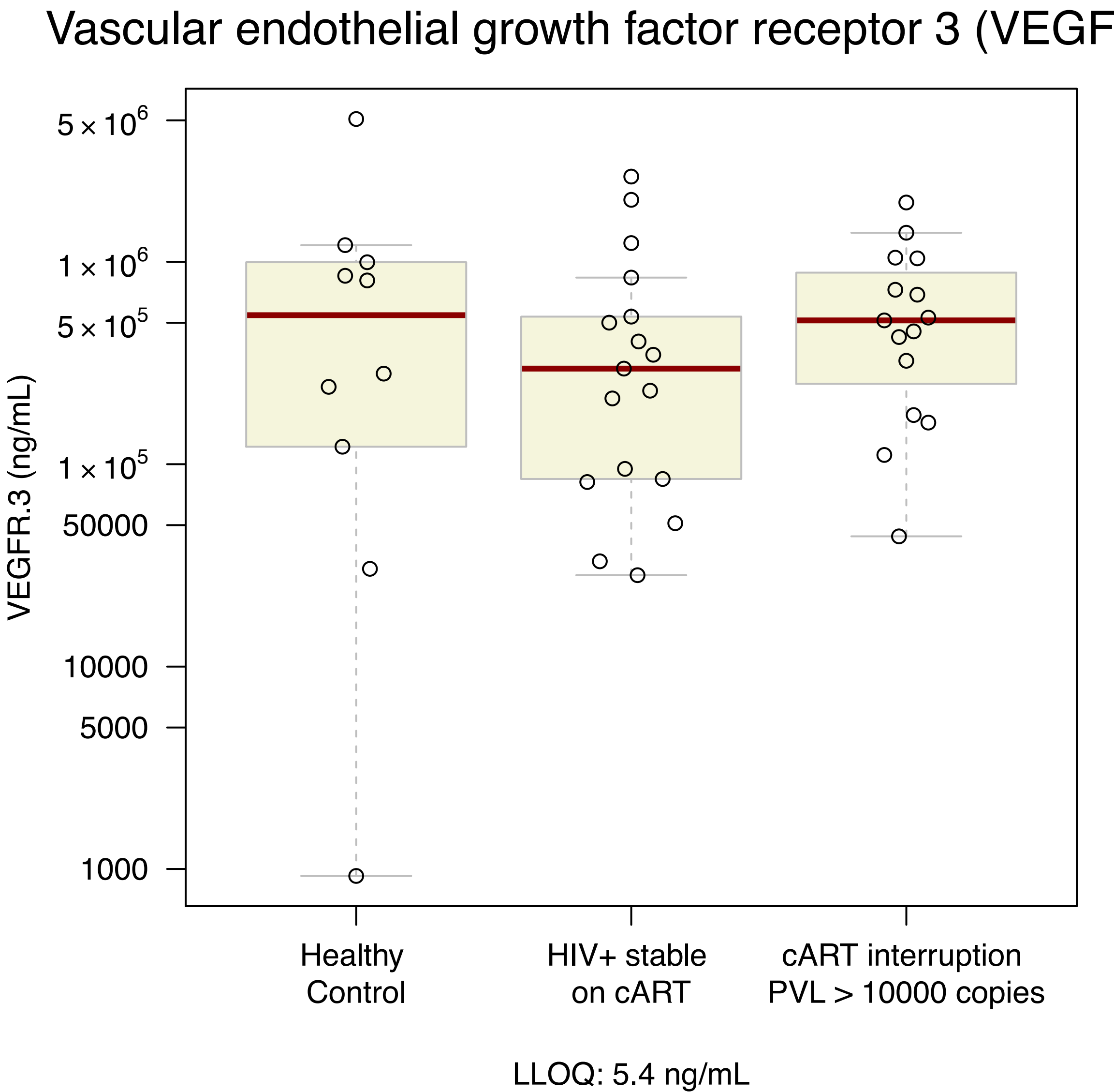
Vascular endothelial growth factor receptor 3 (VEGF
VEGFR.3 (ng/mL)
5 × 10^6
1 × 10^6
5 × 10^5
1 × 10^5
50000
10000
5000
1000
Healthy Control
HIV+ stable on cART
cART interruption PVL > 10000 copies
LLOQ: 5.4 ng/mL

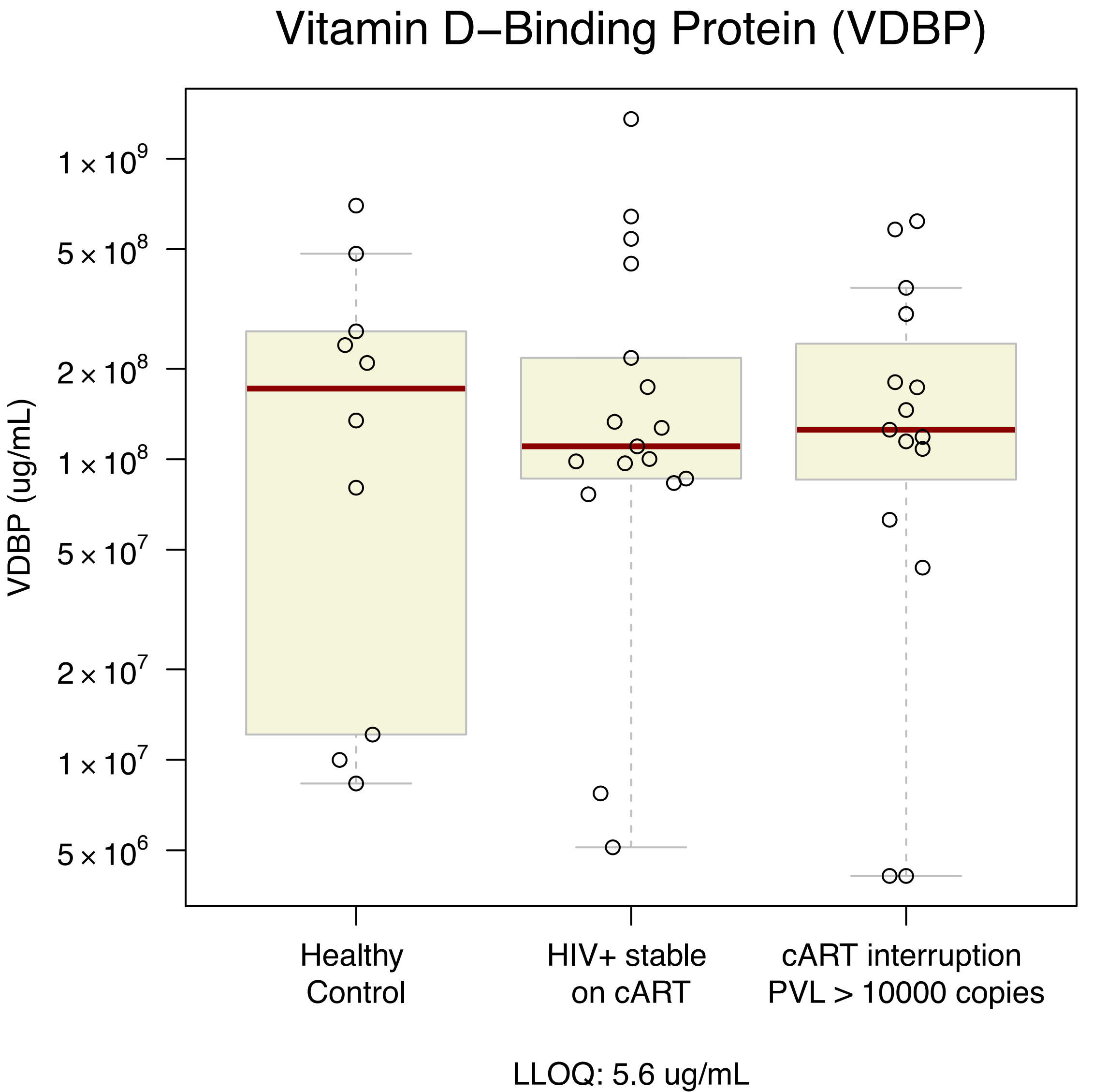

Vitamin D−Binding Protein (VDBP)
VDBP (ug/mL)
1 × 10^9
5 × 10^8
2 × 10^8
1 × 10^8
5 × 10^7
2 × 10^7
1 × 10^7
5 × 10^6
Healthy Control
HIV+ stable on cART
cART interruption PVL > 10000 copies
LLOQ: 5.6 ug/mL

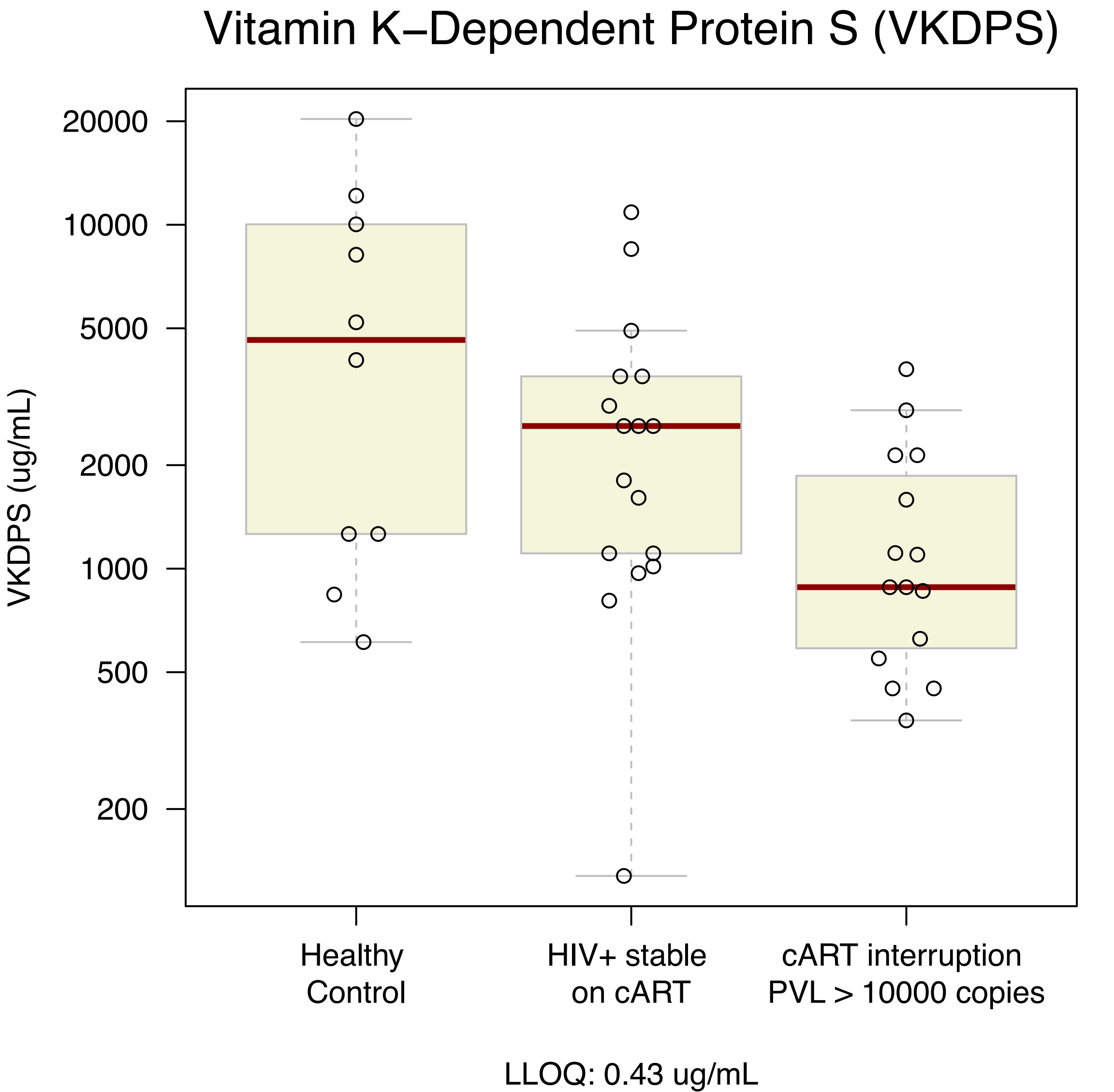
Vitamin K–Dependent Protein S (VKDPS)
VKDPS (ug/mL)
20000
10000
5000
2000
1000
500
200
Healthy Control
HIV+ stable on cART
cART interruption PVL > 10000 copies
LLOQ: 0.43 ug/mL

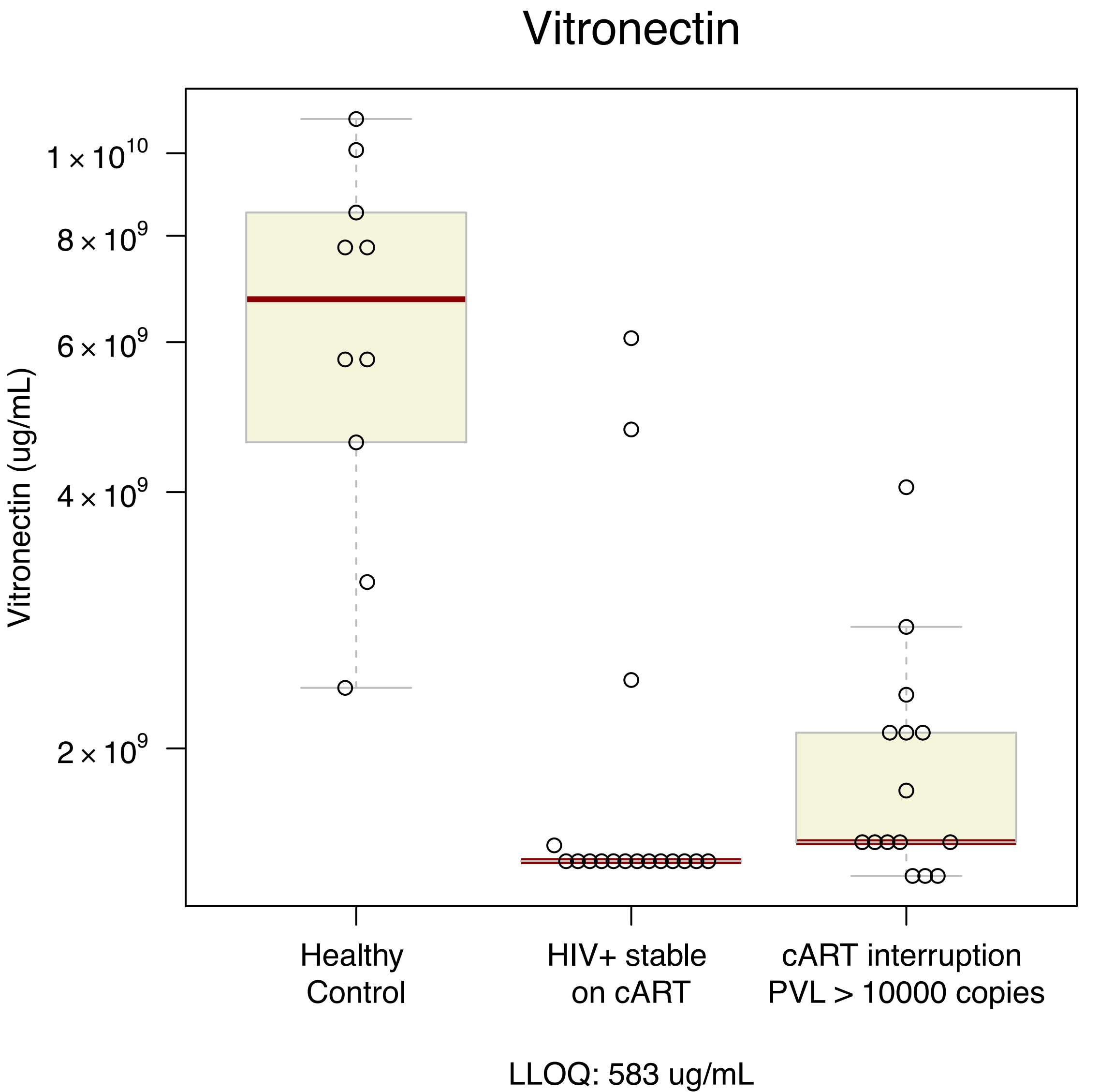

Vitronectin
Vitronectin (ug/mL)
1×10^{10}
8×10^{9}
6×10^{9}
4×10^{9}
2×10^{9}
Healthy Control
HIV+ stable on cART
cART interruption PVL > 10000 copies
LLOQ: 583 ug/mL

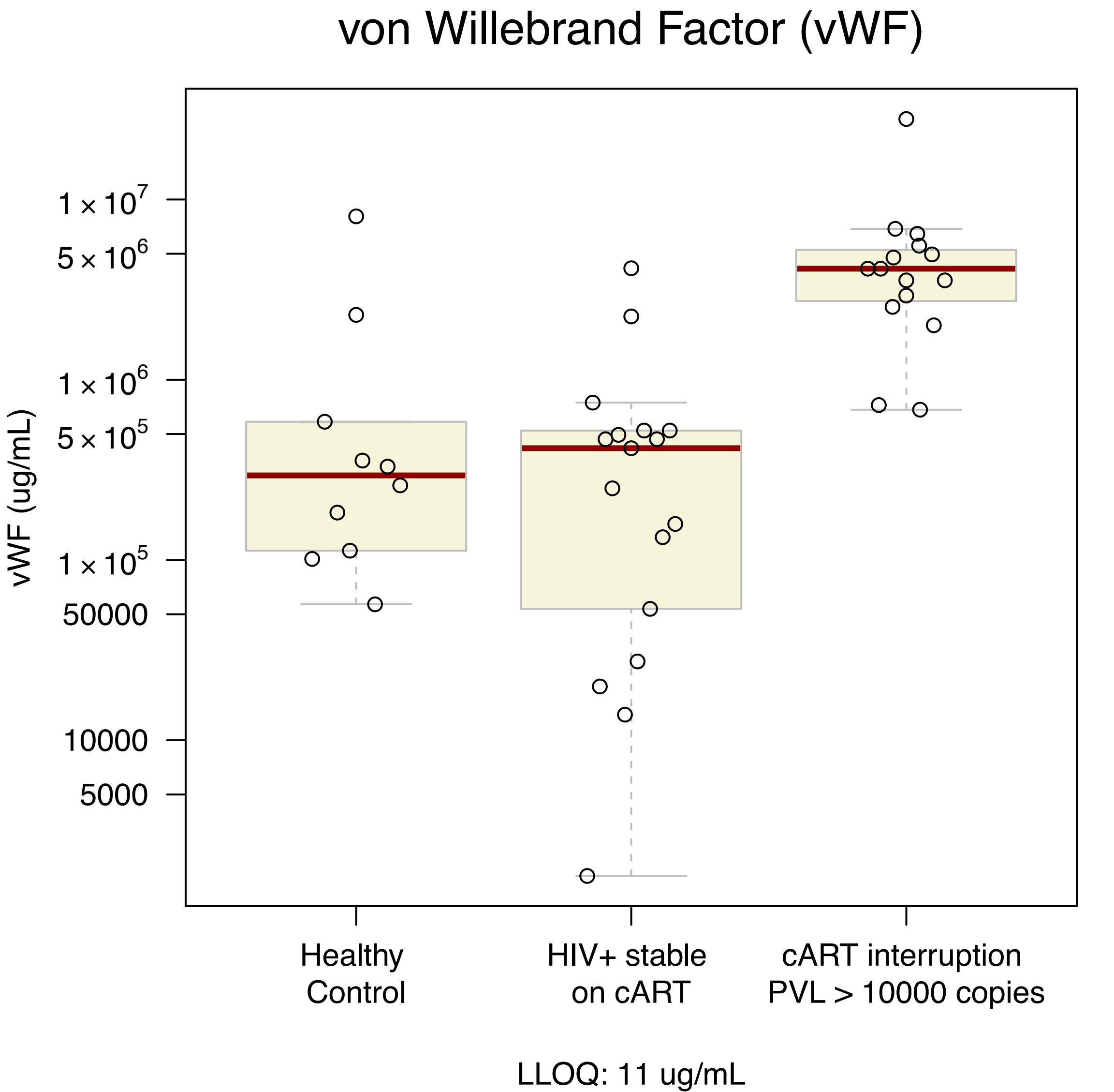

von Willebrand Factor (vWF)
vWF (ug/mL)
1 × 10^7
5 × 10^6
1 × 10^6
5 × 10^5
1 × 10^5
50000
10000
5000
Healthy Control
HIV+ stable on cART
cART interruption PVL > 10000 copies
LLOQ: 11 ug/mL

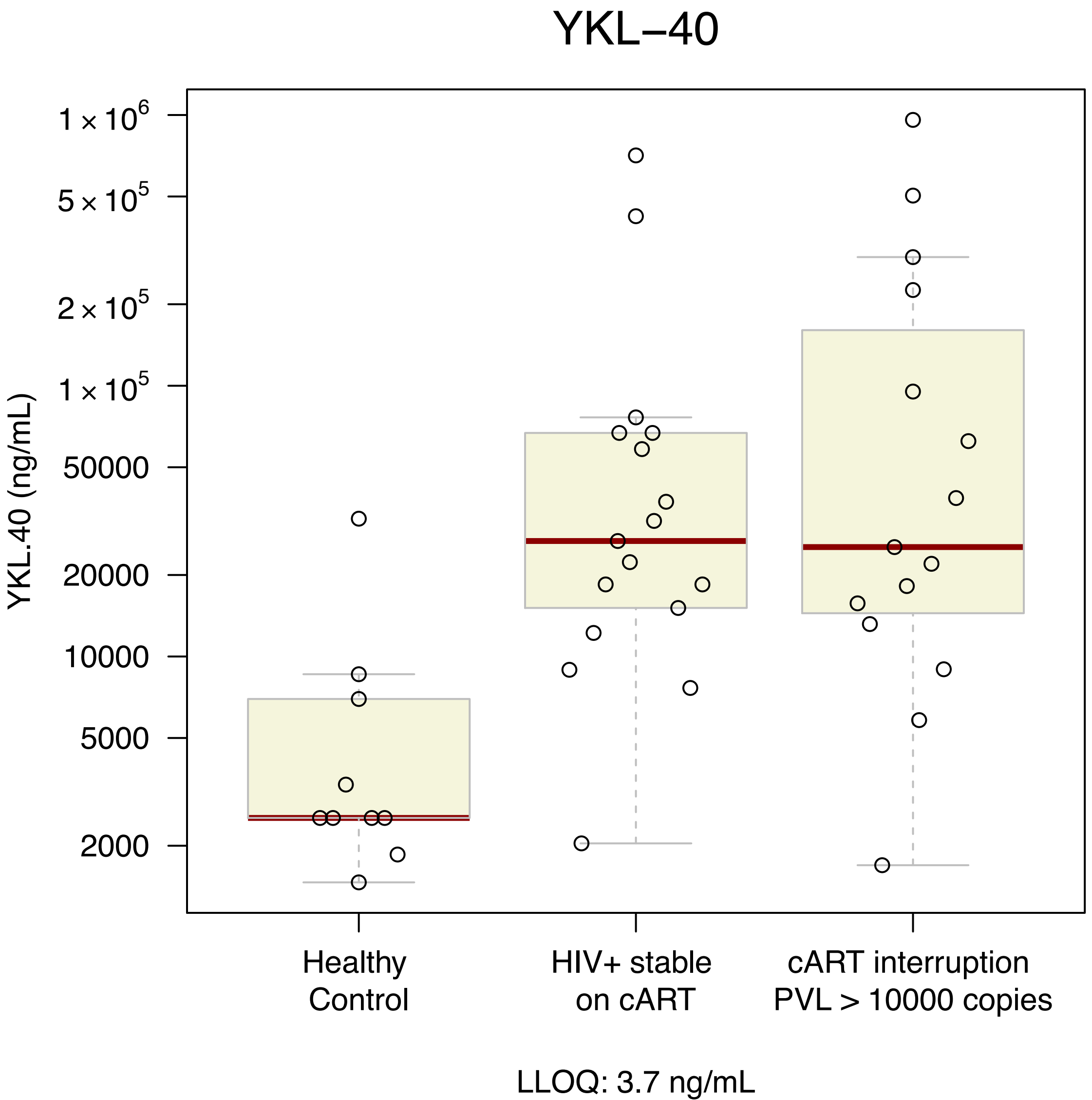

YKL–40
1×10^6
5×10^5
2×10^5
1×10^5
50000
20000
10000
5000
2000
YKL.40 (ng/mL)
Healthy Control
HIV+ stable on cART
cART interruption PVL > 10000 copies
LLOQ: 3.7 ng/mL
